# Short-term effects of extreme air pollutant concentrations on coronary heart disease hospitalization in Henan province: a time-stratified case-crossover study

**DOI:** 10.3389/fcvm.2025.1538788

**Published:** 2025-04-24

**Authors:** Shuming Liu, Yongbin Wang, Lujie Wang, Xuefang Li, Menghui Fei, Pingshuan Dong, Kan Yang, Hui Liu, Na Xie, Hengwen Chen, Guang Chen, Huan Li, Xiayan Zang, Jun Li, Zhigang Chen, Fei Lin, Guoan Zhao

**Affiliations:** ^1^ Department of Cardiology, The First Affiliated Hospital of Xinxiang Medical University, Weihui, China; ^2^ Department of Epidemiology and Health Statistics, Xinxiang Medical University, Xinxiang, China; ^3^ Henan Engineering Technology Research Center of Environmental Meteorological Medicine, The First Affiliated Hospital of Xinxiang Medical University, Weihui, China; ^4^ Department of Cardiology, The First Affiliated Hospital of Henan University of Science and Technology, Luoyang, China; ^5^ Department of Cardiology, Nanyang Central Hospital, Nanyang, China; ^6^ Department of Cardiology, Anyang District Hospital, Anyang, China; ^7^ Department of Cardiology, The Third Affiliated Hospital of Xinxiang Medical University, Xinxiang, China; ^8^ Guang'anmen Hospital, China Academy of Chinese Medical Sciences, Beijing, China; ^9^ Li Ka Shing Faculty of Medicine, The University of Hong Kong, Hong Kong, Hong Kong SAR, China; ^10^ Department of traditional Chinese medicine, The First Affliated Hospital of Xinxiang Medical University, Weihui, China

**Keywords:** air pollution, coronary heart disease, distributed lag nonlinear model, time-stratified case-crossover design, interaction, Henan province

## Abstract

**Introduction:**

Coronary heart disease (CHD) is a leading cause of cardiovascular mortality, with air pollution serving as a significant risk factor. Henan Province, characterized by both a high incidence of CHD and severe air pollution, faces substantial health and economic challenges. However, limited research has explored the relationship between air pollution and CHD in this region.

**Methods:**

This study employs a case-crossover design combined with a distributed lag non-linear model (DLNM) to examine the short-term effects of extreme concentrations of air pollutants (PM₂.₅, PM₁₀, NO₂, SO₂, CO, and O₃) on CHD hospitalizations in Henan. Data on 133,294 confirmed CHD patients from seven large hospitals across five cities (2016–2021) were collected, with patients' addresses linked to nearby air quality monitoring stations to assess exposure to air pollutants and meteorological factors. The time-stratified case-crossover design and DLNM were used to calculate relative risks (RRs) for pollutant exposure on CHD hospitalizations, and subgroup analyses were conducted to identify sensitive groups.

**Results:**

Significant increases in CHD hospitalizations were associated with extremely high concentrations of NO₂, SO₂, and PM₁₀, with maximum RRs of 1.768 for NO₂, 2.821 for SO₂, and 1.728 for PM₁₀ on the 7th cumulative day, while high O₃ levels showed a protective effect. Younger individuals (≤64y) and males were more sensitive to these effects, and high CO concentrations only increase the risk of CHD incidence in the younger (≤64y) subgroup. Synergistic interactions were observed between certain pollutants, such as CO and NO₂/SO₂/PM₁₀, suggesting that the negative impact of CO on CHD is amplified in a multi-pollutant environment due to interactions with other pollutants.

**Discussion:**

These findings highlight the significant public health impact of air pollution on CHD in Henan Province.

## 1 Introduction

Coronary heart disease (CHD), also known as coronary artery atherosclerotic heart disease, is a common cardiovascular condition primarily caused by the narrowing or blockage of coronary arteries. Between 1990 and 2019, the global prevalence of cardiovascular diseases nearly doubled, increasing from 271 million to 523 million cases, while the number of deaths rose from 12.1 million to 18.6 million (1). In addition to posing a significant threat to human health, CHD is expected to impose a substantial economic burden on healthcare systems, particularly in China, where the economic impact of CHD is more severe than in developed countries (2). CHD is a multifactorial disease, with risk factors that include age (3), gender (4), family history (5), environmental influences, genetic predispositions (6), and obesity (7). Notably, exposure to secondhand smoke, in addition to active smoking (8), significantly increases the risk of developing CHD (9). Our team's previous research also indicates a close association between birth month and coronary artery disease (CAD) risk (10), with individuals born in winter exhibiting a higher CAD risk. Furthermore, the incidence of CHD tends to increase with elevated fasting blood glucose levels (11).

Recent research increasingly indicates that air pollutants are significant risk factors for CHD-related hospitalizations and fatal events. For example, a time series analysis in Lanzhou found that each 10 μg/m^3^ increase in NO₂, SO₂, PM₂.₅, and PM₁₀ concentrations was associated with a rise in CHD hospitalization risk by 0.20%, 0.53%, 0.14%, and 0.03%, respectively (12). CO had the greatest impact, with each 1 mg/m^3^ increase raising hospitalization risk by 10.76%, peaking on the third day. In contrast, O₃ concentrations showed a protective effect, with each 10 μg/m^3^ increase reducing hospitalization risk by 0.09%. Additionally, elevated concentrations of PM₂.₅, PM₁₀, SO₂, CO, and NO₂ were associated with an increased risk of hospitalization for CHD in Beijing (13). These pollutants also exhibited a time-lag effect, with their impact being more pronounced under low-temperature conditions. A study in Wuhan (14) similarly demonstrated a significant positive correlation between the concentrations of PM₂.₅, PM₁₀, NO₂, and SO₂ and hospitalizations for ischemic heart disease (IHD), with the impact of air pollution being especially noticeable during the cold season and among elderly individuals (≥76y). Similarly, air pollution in coastal cities of southern China imposes a substantial burden on IHD, with SO₂ and NO₂ exerting a more pronounced influence than PM₂.₅ (15). Although studies on the relationship between air pollution and CHD have originated from diverse geographic locations and used different statistical methodologies, findings consistently highlight the close association between air pollutants and the development and progression of CHD.

Henan Province, located in central China, is one of the most populous provinces in the country, with an estimated 12–16 million heart disease patients. Cardiovascular disease-related deaths rank second nationwide, underscoring substantial public health challenges within the province. In addition to the prevalent dietary habits characterized by high fat, salt, and oil intake, the impact of air pollution on the health of CHD patients in Henan cannot be overlooked. Henan is among the provinces with the most severe air pollution in China, accounting for half of the regions within the bottom 20 rankings nationwide for air quality. According to *Air Pollution Characteristics and Health Risks in Henan Province, China* (16), calculations of the Health Air Quality Index (HAQI) indicate that Henan residents are consistently exposed to polluted air, with approximately 28% of the population living in air conditions harmful to health and 31% exposed to extremely unhealthy air environments. The study emphasizes that PM₂.₅ and PM₁₀ are the predominant pollutants, with air quality particularly deteriorating in winter due to coal heating and unfavorable weather conditions. Additionally, Henan has a high rate of vehicle ownership, with 25.88 million vehicles contributing significantly to air pollution through vehicle emissions. Given these factors, understanding the relationship between air pollution and CHD incidence in Henan Province is of critical importance.

Currently, research on the correlation between air pollution and CHD in Henan Province remains limited. Therefore, this study utilizes a time-stratified case-crossover design combined with DLNM to assess the short-term effects of six air pollutants (PM₂.₅, PM₁₀, NO₂, SO₂, CO, and O₃) at extreme concentrations on CHD hospitalization risk. Additionally, it investigates the interactive effects between different pollutants on CHD hospitalizations, aiming to provide new insights into the relationship between air pollution and CHD incidence in Henan Province.

## 2 Methods

### 2.1 Study population

Between 2016 and 2021, the research team collected data on 133,294 CHD patients from seven large general hospitals across five cities in Henan Province: Xinxiang (The First Affiliated Hospital of Xinxiang Medical University: longitude 114.059, latitude 35.408; The Third Affiliated Hospital of Xinxiang Medical University: longitude 113.925, latitude 35.281; The Affiliated People's Hospital of Xinxiang Medical University: longitude 113.869, latitude 35.306), Nanyang (Nanyang Central Hospital: longitude 112.529, latitude 33.008), Anyang (Anyang First People's Hospital: longitude 114.412, latitude 36.076), Kaifeng (Hospital of Traditional Chinese Medicine Affiliated to Henan University: longitude 114.368, latitude 34.805), and Luoyang (The First Affiliated Hospital of Henan University of Science and Technology: longitude 112.427, latitude 34.599).

All CHD patients included in the study were clinically diagnosed and confirmed, classified under the 10th edition of the International Classification of Diseases (ICD-10: I25). Collected patient data included hospitalization numbers, medical records, gender, age, residential address, and information regarding the first visit. This study did not involve the use of personally identifiable data; therefore, explicit informed consent was not deemed necessary.

### 2.2 Air pollution and weather data

**Figure 1:**
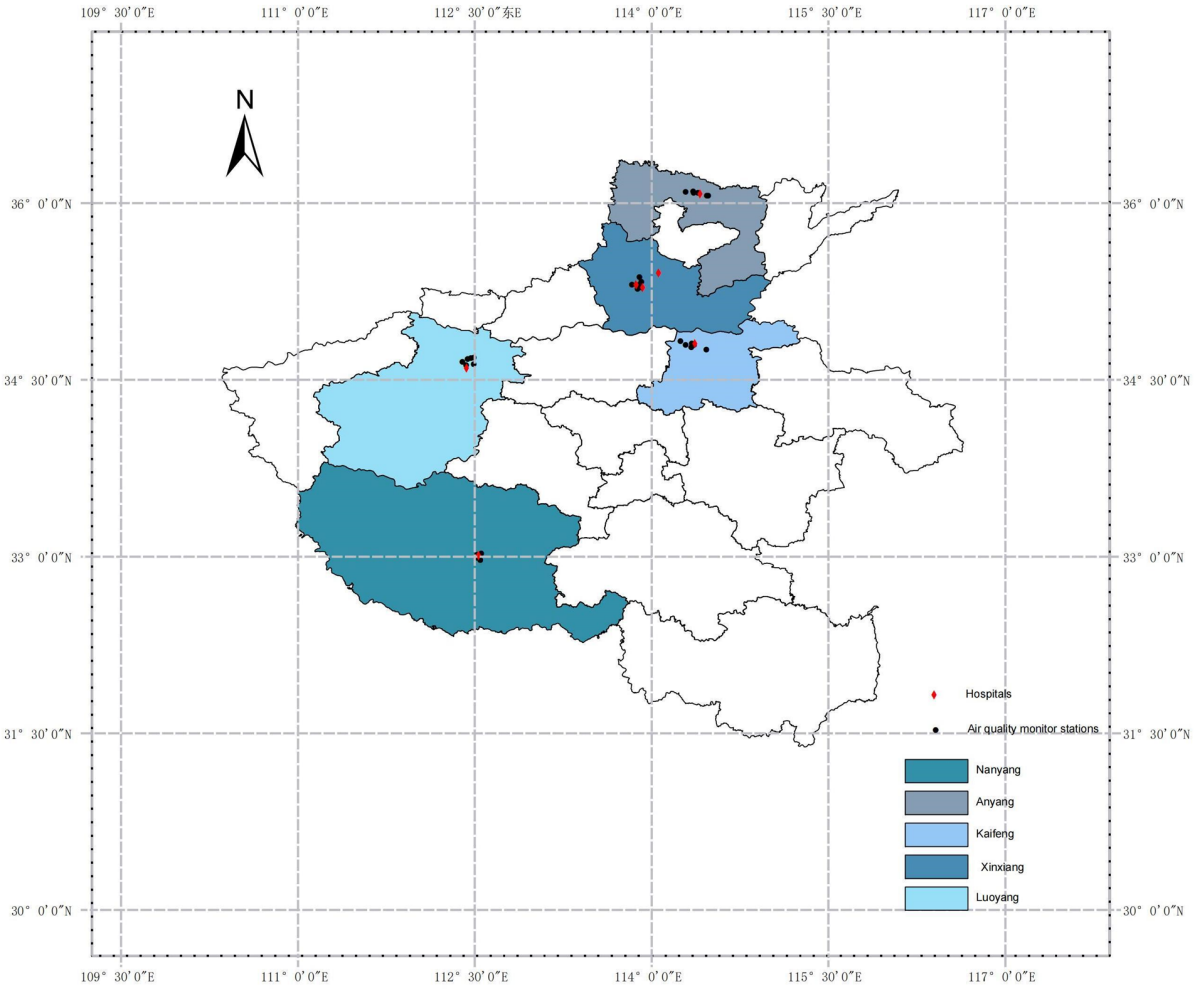
Locations of air pollution monitoring stations and hospitals in Henan province.

The data sources for this study include the China National Environmental Monitoring Center (http://www.cnemc.cn/) and the China National Meteorological Information Center (http://data.cma.cn/). Air pollution data were collected from 28 fixed monitoring stations (see Figure 1; Supplementary Table S1 for details), geocoded on regional maps using ArcGIS 10.5 software. These monitoring stations were strategically located away from roads, industrial areas, and factories to minimize interference from local pollution sources, thereby ensuring that the data accurately reflected the overall air quality in the cities. The primary air pollutants monitored included ozone (O₃), particulate matter (PM₂.₅ and PM₁₀), carbon monoxide (CO), nitrogen dioxide (NO₂), and sulfur dioxide (SO₂). Each participant's home address was geocoded into latitude and longitude coordinates and matched to the nearest air quality monitoring station, enabling a more precise assessment of individual air pollution exposure levels. Additionally, meteorological data, including daily average humidity and daily average temperature, were incorporated into the model as covariates to adjust for the influence of potential confounding factors.

All air pollution and meteorological data underwent processes of data cleaning, transformation, and reshaping, during which missing values and outliers were addressed. Data types were standardized, and variables were renamed to create a comprehensive database for further analysis. This study was approved by the Ethics Committee of the First Affiliated Hospital of Xinxiang Medical University and granted a waiver of informed consent (Approval No: EC-024-573; Xinxiang, China).

### 2.3 Statistical analysis

The time-stratified case-crossover design (TSCC) is a widely employed research methodology, particularly suitable for investigating the effects of environmental exposures on human health. By comparing an individual's exposure levels on days when health events occur (case days) with those on control days within the same month, this design effectively controls for unmeasured long-term trends, seasonal effects, and other potential confounders (17, 18). In this study, we aim to compare CHD hospitalization events on the day of occurrence (case days) with corresponding days of the same week in other weeks within the same month (control days). For instance, if the case day falls on a Wednesday in the first week of a given month, we would select the Wednesdays of the second, third, and fourth weeks of the same month as control days for comparison. Thus, each case has 3–4 control days, which represent the individual's exposure levels to air pollutants on days without the occurrence of a CHD event. Since each CHD patient serves as their own control, potential confounding factors such as age, gender, ethnicity, and lifestyle habits—factors that are unlikely to change substantially in a short period—are effectively controlled.

Regarding the TSCC, previous studies commonly utilize conditional logistic regression and quasi-Poisson regression models, with the choice of model dependent on the characteristics of the research data. Conditional logistic regression is suited for individual-matched case-control studies (19, 20), where each case is compared with multiple control days from the same period and location. This model does not require adjustment for long-term trends or seasonal variables; however, it is less efficient when dealing with aggregated exposure data, especially when exposure data is summarized over large geographic areas, and may also be subject to the risk of overlap bias (21). In contrast, this study employs quasi-Poisson regression, a model well-suited for research involving count data, particularly in time-series studies (22–25). When health events occur frequently, this model not only effectively addresses issues of overdispersion and autocorrelation inherent in count data but also directly applies to time-series data, avoiding the need to transform the data into case-control format. This simplification of the computational process makes the quasi-Poisson regression model especially suitable for analyzing large datasets.

The distributed lag nonlinear model (DLNM) is a versatile and powerful statistical tool for analyzing the health effects of environmental exposures, as it effectively captures both lagged effects and nonlinear exposure-response relationships. By incorporating multidimensional lag parameters, DLNM dynamically characterizes the intricate influence of air pollution exposure on health risks over different lag periods, making it particularly suitable for pollutants with delayed effects. In this study, we employed a quasi-Poisson regression model that integrates the TSCC with DLNM. This approach mitigates potential biases associated with model selection, effectively controls for seasonality, temperature, and other time-dependent confounders, and reduces the reliance on extensive statistical adjustments (26). The quasi-Poisson regression model developed in this study is as follows:

$$Y_{t}∼quasiPoisson(\mu_{t})$$

$$log(\mu_{t})=\alpha +\beta P_{t,l}+ns(Temp_{t},3)+ns(RH_{t},3)+\eta DOW_{t}+\nu Holiday_{t}+\lambda Strata_{t}$$

t:The date of the observation; $Y_{t}$:The number of CHD admissions recorded on day *t*;α:The intercept term; $P_{t,l}$:Cross-basis function related to the daily average concentration of air pollutants on day *t*;β:A vector of associated coefficients; l:The number of lag days; ns:Natural cubic spline function; $DOW_{t}$:The day of the week corresponding to day *t*;η:A vector of coefficients; $Holiday_{t}$:A binary variable indicating whether day *t* is a public holiday (1 if true); ν:Coefficient associated with the variable; $Strata_{t}$:the complete set of stratifications within the study, comprising multiple stratum subsets. Each stratum represents a group of four to five dates that share identical attributes in terms of location, year, month, and day of the week, with one designated as the case day and the remaining three to four as control days (e.g., Anyang, Henan Province—January 2016—Friday). The purpose of this stratification is to ensure that case and control days fall within the same temporal window and geographical setting, thereby enabling valid comparisons; λ:A vector of coefficients. Based on previous studies (12, 27) and the minimum value of the Akaike Information Criterion (AIC) in the quasi-Poisson model, a natural spline function (ns) with three degrees of freedom (df) was applied to smooth the nonlinear effects of daily average temperature and humidity. Given that the cardiovascular impacts of air pollution are often acute, the association between CHD onset and air pollution exposure tends to be short-term. To better capture these acute effects, a 7-day lag period was considered, with lag effects analyzed as both single-day and cumulative lags. In this study, Spearman correlation analysis indicated that the correlation coefficients between PM₂.₅ and PM₁₀, NO₂, CO, as well as between NO₂ and PM₁₀, SO₂, and between average temperature and ozone, all exceeded 0.6. In contrast, the correlation coefficients among the remaining pollutants (excluding O₃) were generally slightly below 0.6. Given these high correlations, the application of a multi-pollutant model may not be appropriate, as it could introduce multicollinearity issues, leading to unstable parameter estimation and consequently undermining the interpretability and predictive capacity of the model (22). Moreover, employing a multi-pollutant model complicates the precise delineation of each pollutant's independent contribution to health outcomes, as their effects may overlap or interact. Additionally, the incorporation of multiple pollutant variables would significantly increase model complexity, heightening the risk of over-fitting. To mitigate these potential sources of bias and ensure robust risk estimation, we opted for a single-pollutant model approach to independently assess the effects of each air pollutant. Relative risk (RR) estimates and 95% confidence intervals (CI) were used to quantify the association between extremely high and low pollutant concentrations and CHD hospitalizations in a single-pollutant model. Extremely high air pollution concentration is defined as the pollutant concentration at the 99th percentile, while extremely low concentration is defined at the 1st percentile, the effects of these concentrations are assessed using the relative risk at the 99th and 1st percentiles compared to the median. Age was stratified into groups below 65 years (≤64y) and 65 years or older (≥65y), while gender was analyzed separately to identify potentially vulnerable subgroups related to exposure to extreme air pollution concentrations.

As single-pollutant models struggle to elucidate the complex interactions among air pollutants in relation to CHD hospitalizations, we incorporated interaction terms into the model to quantify the synergistic or attenuating effects among independent variables under co-exposure scenarios (28). The interaction effects of air pollutants factors on CHD were further quantified. Air pollution levels were categorized as “low” or “high” relative to the median, and one pollutant was chosen as a reference to examine the combined effect on CHD hospitalizations. RR_11_ represents the relative risk when both pollutants are “high”; RR_01_ and RR_10_ represent the relative risks when one pollutant is “high” and the other is “low”; RR_00_ represents the relative risk when both pollutants are “low,” serving as the control group with a baseline value of 1. Interaction Relative Risk (IRR) and Relative Excess Risk due to Interaction (RERI) were used to evaluate the interaction effects. The specific calculation formulas are as follows:

$$IRR\;=\frac{RR_{11}}{\left(RR_{01}\times RR_{10}\right)}$$

$$RERI\;=\;RR_{11}\;-RR_{10}\;-RR_{01}\;+1$$

An interaction was considered significant only if IRR were not equal to 1, RERI was not equal to 0, and the *p*-value was <0.05. If IRR >1 or RERI >0, it indicates a synergistic interaction; if IRR <1 or RERI <0, it indicates an antagonistic interaction.

To test the robustness of the results, a sensitivity analysis was conducted by modifying the maximum lag period of the DLNM from 7 days to 5, 6, 8, or 9 days and adjusting the degrees of freedom for temperature and humidity variables from 3 to 2 or 4. All data analyses were performed using R software (version 4.3.2), values of *p* < 0.05 were considered statistically significant.

## 3 Results

**Figure 2:**
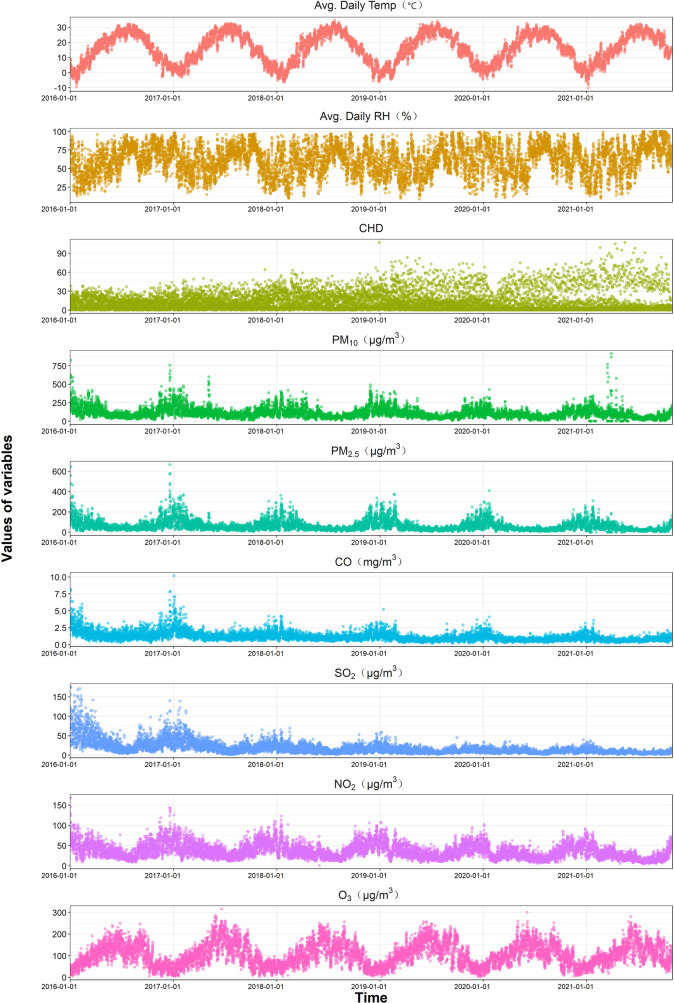
Time series plot of daily air pollutants and CHD hospitalizations in Henan from 2016 to 2021.

The scatter plot of air pollution concentration and the distribution of CHD hospitalizations in Henan Province from November 1, 2016, to December 31, 2021 (Figure 2) shows that the number of CHD hospital admissions was higher in the colder seasons and generally showed an upward trend during the study period. O₃ concentration was higher in the summer due to the influence of temperature and sunlight duration. Meanwhile, concentirations of PM₂.₅, PM₁₀, NO₂, SO₂, and CO remain relatively stable, with higher levels generally observed in colder seasons. But a notable exception was the period around January 1, 2016, and 2017, when the concentration of pollutants (except O₃) was significantly higher, and subsequently, pollution levels were somewhat controlled. The characteristics of study population, air pollutant concentrations, and meteorological factors are presented in Table 1. During the study period, a total of 133,294 CHD hospitalizations were recorded, with males accounting for 53.72% (71,600 cases), females 43.70% (58,249 cases), and cases with missing gender data comprising 2.58% (3,445 cases). Patients aged over 65 years (≥65y) represented 56.13% (74,813 cases), while those aged 0–64 years (≤64y) made up 43.87% (58,479 cases), with only 0.0015% (2 cases) missing age data. The average concentrations of air pollutants during this period were as follows: PM₂.₅, 61.84 µg/m^3^; PM₁₀, 109.18 µg/m^3^; SO₂, 17.97 µg/m^3^; NO₂, 36.53 µg/m^3^; CO, 1.18 mg/m^3^; and O₃, 104.29 µg/m^3^. The average daily temperature was 16.06°C, and the average daily relative humidity was 61.61%. Sunshine duration data were missing for a total of 304 days from January 1 to October 31, 2021. Therefore, “sunshine duration” was not included as a covariate in the model.

**Table 1:** Summary statistics of CHD hospitalizations, air pollutant, and meteorological factors in henan province from 2016 to 2021.

Variables	$\bar{\text{\textbackslash{},}x}\pm \text{\textbackslash{},}S$	Minimum	P_25_	P_50_	P_75_	Maximum
Hospital admissions						
Total	12.51 ± 14.78	0.00	2.00	7.00	18.00	107.00
Male	6.72 ± 8.44	0.00	1.00	3.00	10.00	71.00
Female	5.47 ± 6.61	0.00	1.00	3.00	8.00	57.00
Young	5.49 ± 6.19	0.00	1.00	3.00	9.00	46.00
Old	7.02 ± 9.19	0.00	1.00	3.00	10.00	66.00
Air pollutants						
PM_2.5_(µg/m^3^)	61.84 ± 50.93	0.00	30.00	45.00	75.00	665.00
PM_10_(µg/m^3^)	109.18 ± 71.30	0.00	63.00	92.00	134.00	915.00
CO(mg/m^3^)	1.18 ± 0.69	0.20	0.80	1.00	1.40	10.20
SO_2_(µg/m^3^)	17.97 ± 16.60	2.00	8.00	13.00	22.00	176.00
NO_2_(µg/m^3^)	36.53 ± 18.27	1.00	22.00	33.00	48.00	168.00
O_3_(µg/m^3^)	104.29 ± 52.44	4.00	62.00	99.00	142.00	316.00
Average daily meteorological factors						
Temp (°C)	16.06 ± 9.78	−10.20	7.60	16.90	25.00	34.60
RH (%)	61.61 ± 19.20	9.00	47.00	63.00	77.00	100.00

PM_2.5_, particulate matter with an aerodynamic diameter ≤2.5 µm; PM_10_, particulate matter with an aerodynamic diameter ≤10 µm; NO_2_, nitrogen dioxide; CO, carbon monoxide; SO_2_, sulfur dioxide; O_3_, ozone; Temp, average daily temperature; RH, average daily relative humidity; SD, standard deviation; Px, percentile of the data; CHD, coronary heart disease.

**Figure 3:**
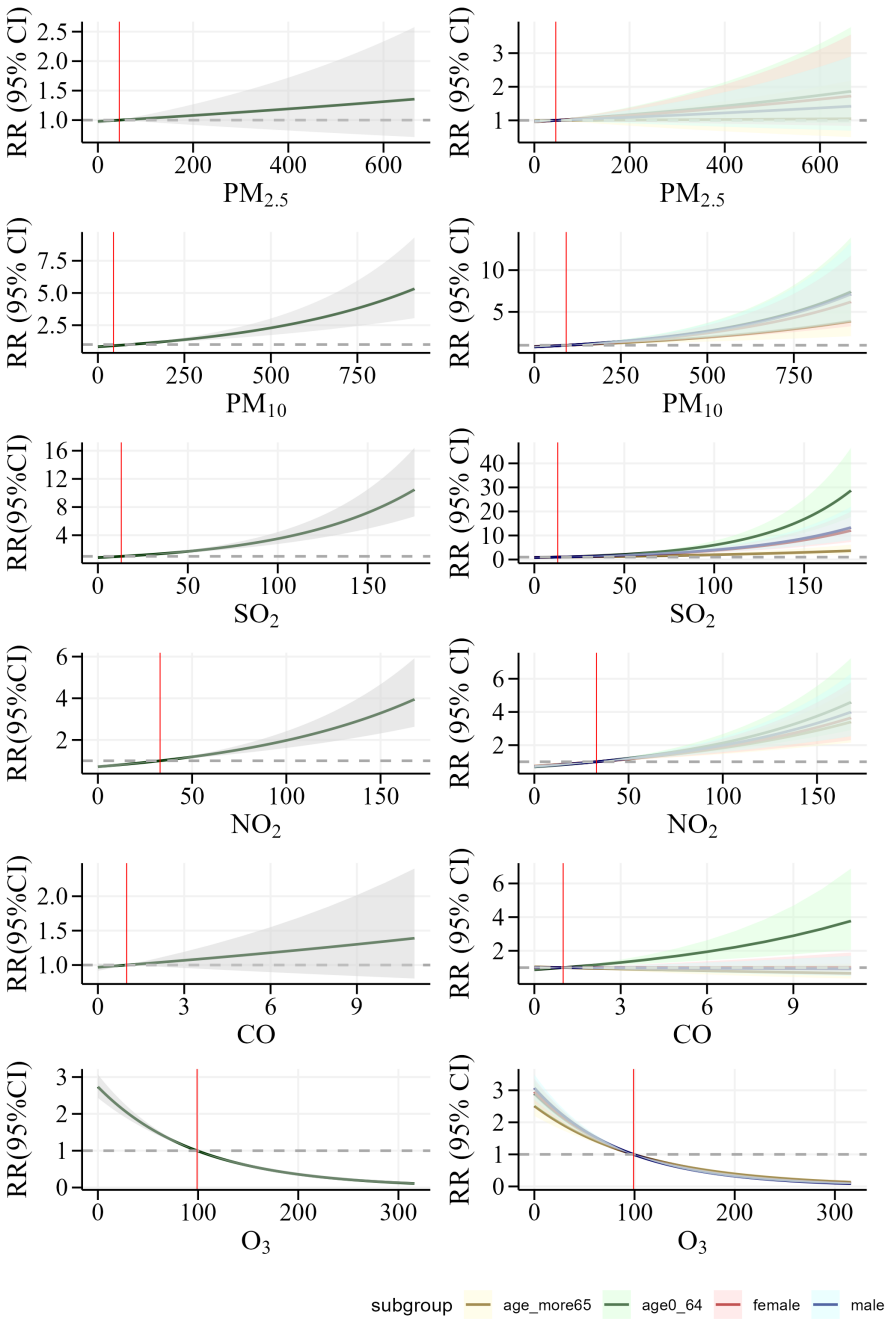
Cumulative exposure-response curves of Air pollutant concentrations and CHD hospitalizations, overall and stratified by Age and gender, in Henan province, 2016–2021.

**Figure 4:**
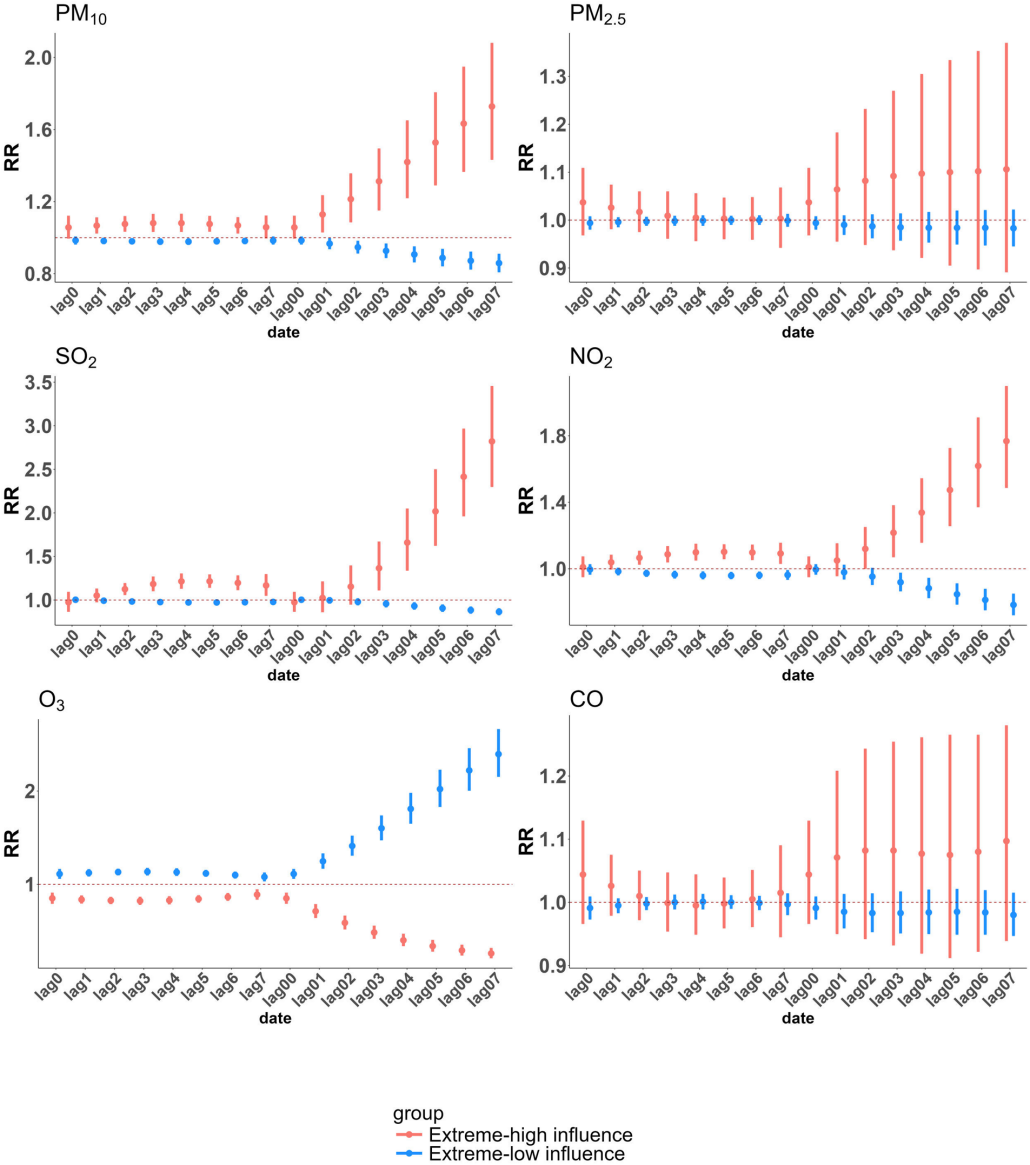
Relative risks (RRs) and 95% confidence intervals (CIs) of CHD hospitalizations associated with extreme concentrations of each pollutant in Henan province from 2016 to 2021. Extremely high air pollutant concentration is defined as the concentration at the 99th percentile, while extremely low concentration is defined at the 1st percentile. The effects of extremely high concentration are assessed by comparing the relative risk at the 99th percentile with the median, and the effects of extremely low concentration are assessed by comparing the relative risk at the 1st percentile with the median.

We employed cumulative exposure-response curves to illustrate the impact of varying concentrations of different air pollutants on CHD hospitalizations across distinct exposure subgroups, stratified by age and gender (Figure 3). The exposure-response relationships for PM₁₀, SO₂, and NO₂ with CHD hospitalization rates exhibited a nonlinear, no-threshold pattern (*P* < 0.05), whereas the trend for O₃ was precisely the opposite of that observed for the other pollutants. Notably, no significant association was detected for PM₂.₅ and CO in the exposure-response curves. Subgroup analyses revealed that younger individuals and males were more susceptible to increased CHD hospitalization risks associated with PM₁₀, SO₂, and NO₂ exposure. However, across all concentration levels and subgroups, no definitive link was established between PM₂.₅ exposure and elevated CHD hospitalization risk. In contrast, a significant association between elevated CO concentrations and increased CHD hospitalization risk was observed exclusively among individuals under the age of 65, underscoring a distinct vulnerability unique to this demographic group. Figure 4 illustrates the relative risk and 95% confidence interval of CHD hospitalizations associated with extremely high and low concentrations of air pollutants in a single-pollutant model. At extremely high concentrations, NO₂, SO₂, and PM₁₀ show a significant association with increased CHD hospitalizations compared to median levels, while extremely low concentrations exhibit a protective effect. The lag effect is strongest on the 4th single day and the 7th cumulative day. Across cumulative lag days, the impact of extremely high and low concentrations of NO₂, SO₂, and PM₁₀ on CHD hospitalizations follows a linear pattern, with the cumulative effect of extremely high concentrations intensifying with extended lag periods, the maximum RR values are 1.768 (95% CI: 1.495, 2.091) for NO₂ (99th vs. median), 2.821 (95% CI: 2.314, 3.440) for SO₂ (99th vs. median), and 1.728 (95% CI: 1.440, 2.073) for PM₁₀ (99th vs. median). In contrast to other air pollutants, O₃ demonstrates a protective effect at extremely high concentrations compared to the median. No significant association is observed between either extremely high or low concentrations of CO and PM₂.₅ and CHD incidence across any lag days.

In the subgroup analysis based on age and gender, Supplementary Tables S2, S3 present the cumulative lagged effects of extreme pollutant concentrations on CHD hospitalizations in different subgroups, with single-day lagged effects detailed in Supplementary Tables S4, S5. In this study, extremely high concentrations of NO₂, SO₂, and PM₁₀ as well as extremely low concentrations of O₃, are associated with a higher relative risk in young (≤64y) and males. Specifically, for SO₂ and NO₂, subgroup analyses by age and gender revealed no significant differences in relative risk during the initial lag period (lag0–lag2); however, from lag3 onwards, a pronounced increase in risk emerged among younger males. This suggests that the detrimental effects of high concentrations of SO₂ and NO₂ on younger male populations are not immediate but rather exhibit a delayed onset, likely requiring cumulative exposure to manifest their full impact. During the early lag period (lag0–lag1), elderly females exhibited a higher risk of CHD hospitalization following PM₁₀ exposure, indicating greater susceptibility to its immediate effects. This heightened vulnerability may be attributed to their diminished immune tolerance and increased sensitivity, rendering them more prone to adverse cardiovascular outcomes in the initial phase of exposure. However, from lag2 onward, younger males began to exhibit a markedly higher risk than elderly females. A plausible explanation for this shift could be the increased likelihood of prolonged outdoor exposure among younger males, particularly those engaged in outdoor occupations. Coupled with lower awareness or adherence to protective measures, this sustained exposure may lead to a “threshold breakthrough” effect. Of particular note, SO₂ demonstrated the highest relative risk among the pollutants studied, with the risk among younger individuals exceeding that of the elderly by more than twofold.

Conversely, extremely low concentrations of NO₂, PM₁₀, and SO₂, as well as extremely high concentrations of O₃, demonstrated a protective effect against CHD hospitalizations when compared to median levels, with significant variations observed across different subgroups. Notably, the potential protective effect of O₃ was particularly pronounced among younger males throughout the entire lag period, warranting further investigation to elucidate the underlying mechanisms driving these disparities. Furthermore, extreme concentrations of PM₂.₅ did not exhibit any significant association with CHD hospitalizations across all subgroups, suggesting a comparatively weaker impact relative to other pollutants. Similarly, extreme CO concentrations showed no significant effects in gender-stratified analyses. However, high CO levels were associated with detrimental effects among younger individuals (≤64y) on specific single lag days (lag2–lag6) and over cumulative lag periods (lag04 to lag07), with the highest relative risk observed at lag07 (RR = 1.450; 95% CI: 1.225, 1.716). In contrast, among elderly individuals, high CO concentrations were linked to significant adverse effects on the day of exposure, indicating an immediate health impact in this population.

**Figure 5:**
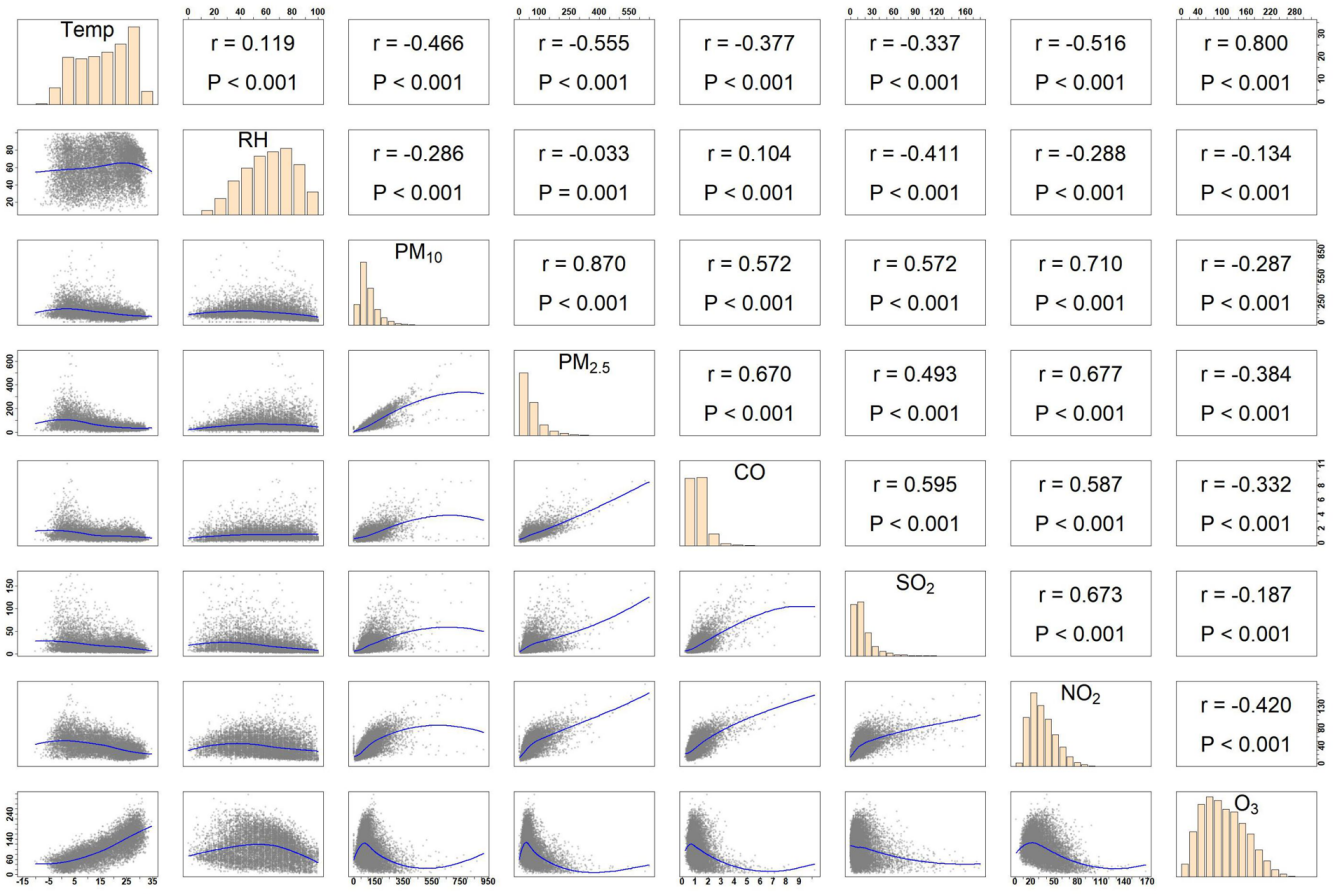
Spearman's correlation coefficients between daily air pollutants and meteorological data in Henan province, 2016–2021.

Spearman rank correlation analysis can measure the monotonic relationship between meteorological factors and air pollutants. As shown in Figure 5, the correlation between PM₁₀ and PM₂.₅ was the strongest, with a coefficient of 0.87, followed by the correlation between PM₁₀ and NO₂, which was 0.71. Notably, O₃ exhibited a negative correlation with other pollutants, while the remaining five air pollutants demonstrated positive correlations to varying extents. In particular, the correlation coefficients between PM₂.₅ and PM₁₀, NO₂, and CO, as well as between NO₂ and PM₁₀, SO₂, and between average temperature and O₃, all exceeded 0.6, indicating a relatively high level of association. Table 2 presents the interaction analysis of air pollutants on CHD hospitalizations. Significant synergistic interactions (*P* < 0.05, IRR >1, RERI >0) were observed between CO and the pollutants NO₂, SO₂, and PM₁₀. In interactions involving O₃, when PM₁₀ or CO was used as the reference pollutant, antagonistic effects were observed (IRR <1, RERI <0), with the RERI for CO and O₃ remaining non-significant. For PM₂.₅ and PM₁₀, or PM₁₀ and SO₂ (with PM₁₀ as the reference), only RERI was statistically significant (*P* < 0.05), while IRR demonstrated no notable effects. Among all pollutant pairs, SO₂ and CO exhibited the strongest interaction, with an IRR of 1.247 (95% CI: 1.178, 1.317) and an RERI of 0.198 (95% CI: 0.155, 0.241).

**Table 2:** Interaction analysis between various air pollutants on coronary heart disease hospitalizations in Henan province from 2016 to 2021.

Pollu-tant	CO	NO_2_	SO_2_	O_3_	PM_10_	PM_2.5_
CO		IRR: 1.115 (1.053, 1.177)*	IRR: 1.247 (1.178, 1.317)*	IRR: 0.962 (0.912, 1.012)	IRR: 1.065 (1.007, 1.123)*	IRR: 1.007 (0.950, 1.063)
		RERI: 0.096 (0.051, 0.141)*	RERI: 0.198 (0.155, 0.241)*	RERI: −0.033 (−0.085, 0.019)	RERI: 0.060 (0.019, 0.100)*	RERI: 0.005 (−0.036,0.046)
NO_2_	IRR: 1.116 (1.054, 1.178)*		IRR: 1.024 (0.967, 1.081)	IRR: 0.985 (0.932, 1.038)	IRR: 1.032 (0.973, 1.091)	IRR: 1.034 (0.974, 1.094)
	RERI: 0.093 (0.050, 0.137)*		RERI: 0.013 (−0.030, 0.056)	RERI: −0.016 (−0.067, 0.036)	RERI: 0.029 (−0.011, 0.069)	RERI: 0.033 (−0.008, 0.073)
SO_2_	IRR: 1.243 (1.174, 1.312)*	IRR: 1.037 (0.979, 1.094)		IRR: 0.998 (0.946, 1.051)	IRR: 1.051 (0.996, 1.105)	IRR: 1.023 (0.971, 1.075)
	RERI: 0.196 (0.152, 0.241)*	RERI: 0.028 (−0.018, 0.074)		RERI: 0.002 (−0.050, 0.055)	RERI: 0.049 (0.005, 0.093)*	RERI: 0.021 (−0.024, 0.066)
O_3_	IRR: 0.938 (0.888, 0.987)*	IRR: 0.956 (0.904, 1.007)	IRR: 0.976 (0.924, 1.028)		IRR: 0.934 (0.885, 0.983)*	IRR: 0.950 (0.900, 1.001)
	RERI: −0.046 (−0.098, 0.006)	RERI: −0.045 (−0.098, 0.008)	RERI: −0.008 (−0.059, 0.043)		RERI: −0.062 (−0.111, −0.012)*	RERI: −0.048 (−0.097, 0.002)
PM_10_	IRR: 1.063 (1.005, 1.121)*	IRR: 1.024 (0.966, 1.083)	IRR: 1.040 (0.987, 1.094)	IRR: 0.955 (0.905, 1.004)		IRR: 1.068 (0.994, 1.142)
	RERI: 0.055 (0.014, 0.097)*	RERI: 0.023 (−0.020, 0.066)	RERI: 0.036 (−0.006, 0.079)	RERI: −0.045 (−0.095, 0.005)		RERI: 0.063 (0.027, 0.099)*
PM_2.5_	IRR: 1.008 (0.951, 1.065)	IRR: 1.028 (0.968, 1.087)	IRR: 1.021 (0.969, 1.073)	IRR: 0.972 (0.921, 1.023)	IRR: 1.072 (0.998, 1.147)	
	RERI: 0.004 (−0.037, 0.046)	RERI: 0.027 (−0.016, 0.070)	RERI: 0.017 (−0.028, 0.061)	RERI: −0.028 (−0.078, 0.023)	RERI: 0.067 (0.030, 0.103)*	

Statistically significant values are bolded and marked with “*”.

The detailed results of the sensitivity analysis are provided in the supplementary file (Supplementary Tables S6–S64). Adjusting the degrees of freedom (df) for temperature and humidity from 3 to 2 or 4, as well as modifying the maximum lag days from 7 days to 5, 6, 8, or 9, yields results consistent with the primary findings.

## 4 Discussion

This study focuses on Henan Province and employs a single pollutant model in conjunction with a DLNM based on a case-crossover design to investigate the impact of individual air pollutants on CHD hospitalizations. The findings indicate that extremely high concentrations of NO₂, SO₂, and PM₁₀ significantly increased the risk of CHD hospitalizations when compared to median levels, while extremely high concentrations of O₃ demonstrated a protective effect. The observed associations between elevated concentrations of NO₂, SO₂, and PM₁₀ and CHD hospitalizations are largely consistent with previous studies (29–31).

However, the effects of O₃ are complex and variable. In most cardiovascular studies (32, 33), elevated O₃ concentrations have been shown to significantly increase the incidence of various cardiovascular diseases, particularly when O₃ levels exceed the World Health Organization's recommended threshold of 100 µg/m^3^ (34). A study in Sweden found that a 10 µg/m^3^ rise in the lag2 and lag7 average O₃ concentration led to a 0.7% and 2.7% increase in cardiovascular mortality, respectively (35). Similarly, a 2024 nationwide cohort study in China reported (36) that for every 10 µg/m^3^ increase in long-term O₃ exposure, the risk of CHD increased by 15%. Additionally, short-term O₃ exposure has been linked to an elevated risk of atrial fibrillation episodes (37). Nevertheless, not all studies have found a consistent association between O₃ and cardiovascular disease. An Australian time-stratified case-crossover study on air pollution and acute myocardial infarction (AMI) emergency department visits found no strong correlation between O₃ exposure and AMI risk (38). A 2023 meta-analysis further highlighted that (39) short-term exposure to PM₂.₅, PM₁₀, NO₂, and CO was significantly associated with increased hospitalization and mortality risk from heart failure, while O₃ demonstrated no significant short-term effects.

Interestingly, a 2024 case-crossover study (40) on acute cardiovascular events in New York City found that summer O₃ concentrations were negatively correlated with cardiovascular risk, potentially due to the chemical scavenging effect of O₃. Similarly, several other studies (12, 27, 41) have also documented an inverse association between O₃ levels and CHD incidence, these ozone-related findings are consistent with those of our study, while the underlying mechanisms remain incompletely understood. Previous research has suggested that O₃ may induce oxidative preconditioning, enhancing the activity of key antioxidant enzymes. This, in turn, reduces lipid peroxidation and protein oxidation, thereby mitigating oxidative stress-induced cardiac injury (42). Moreover, ozone preconditioning has been shown to activate the Nrf2 pathway, upregulating the expression of antioxidant proteins such as Slc7a11 and Gpx4 (43). This mechanism plays a crucial role in inhibiting ferroptosis triggered by ischemia-reperfusion injury, thereby attenuating myocardial damage associated with ischemic episodes. Additionally, O₃ facilitates the release of nitric oxide, a potent vasodilator that enhances endothelial function, promotes myocardial perfusion, and increases oxygen delivery to the heart. As a result, O₃ alleviates myocardial ischemia and reduces cardiomyocyte injury (44). Beyond these effects, ozone has demonstrated the capacity to improve hemorheological properties by reducing blood viscosity, thereby enhancing circulation, optimizing myocardial oxygenation, and facilitating cardiac metabolism (45). While these mechanistic insights provide compelling evidence supporting the potential cardioprotective effects of ozone, further research is required to fully elucidate the precise physiological pathways underlying these benefits.

Numerous prior studies have firmly established a strong association between CO, PM₂.₅, and cardiovascular risk. For instance, a Shanghai study (46) linked a 10 μg/m^3^ pollutant increase (lag0) to 0.68% and 0.08% rises in out-of-hospital CHD mortality for PM₂.₅ and CO, respectively. Similarly, In Hubei Province (47), acute exposure to PM₂.₅, CO, and other pollutants was linked to longer hospital stays for IHD patients. Furthermore, two meta-analyses (39, 48) revealed a significant increase in the risk of hospitalization and mortality due to heart failure following exposure to PM₂.₅ and CO, even at low concentrations (49). Additionally, elevated CO levels have been strongly associated with a heightened risk of heart failure rehospitalization in patients with unstable angina (50). Regarding PM₂.₅, an extensive body of literature has unequivocally demonstrated its role in elevating CHD risk (51–55). Long-term exposure to PM₂.₅ is particularly concerning, as every 10 μg/m^3^ increase in PM₂.₅ concentration has been linked to a 43% increase in total CHD risk (HR 1.43), a 45% increase in non-fatal CHD risk (HR 1.45), and a 38% increase in fatal CHD risk (HR 1.38) (56). A similar time-stratified case-crossover study conducted across nine cities in Sichuan Province further corroborated these findings (57), showing that for every 10 μg/m^3^ rise in PM₁₀ and PM₂.₅ concentrations, CHD hospitalization risk increased by 0.46% and 0.57%, respectively. Beyond that, PM₂.₅ exposure has also been linked to increased mortality and economic burden from ischemic heart disease (58–60), alongside a potential heightened risk of ventricular arrhythmias (61, 62). Strikingly, an umbrella review (63) integrating previous meta-analyses concluded that PM₂.₅ is significantly associated with nearly all major cardiovascular diseases, including mortality, myocardial infarction, stroke, hypertension, and atherosclerosis.

However, the non-significant associations between CO/PM₂.₅ exposure and CHD observed in this study diverge from previous epidemiological findings. Potential explanations include: First, our analysis utilized a maximum 7-day lag period, whereas PM₂.₅ or CO effects might manifest over longer lags (>10 days). Second, differences in study regions can also impact the experimental results. For example, Beijing reported null associations between SO₂, CO, or O₃ and out-of-hospital cardiac arrest (64), whereas Italian studies identified significant CO-OHCA risks (65). Similarly, PM₂.₅ and PM₁₀ exhibited stronger associations with acute cardiovascular events in Taiwan's central/southern regions than in northern/eastern areas (66). Furthermore, COVID-19 pandemic restrictions during the study period likely increased indoor residence time, potentially decoupling indoor pollutant exposures from outdoor monitoring data and introducing exposure misclassification. Finally, single-pollutant models were employed to avoid multicollinearity, though atmospheric pollutants typically co-occur, which may have led to the introduction of bias. Notably, synergistic effects between CO and NO₂/SO₂/PM₁₀ emerged in interaction analyses, suggesting CO's cardiovascular impacts may be amplified in multi-pollutant contexts through oxygen transport impairment, oxidative stress, and inflammatory pathways (67, 68).

In interaction analyses centered on PM₁₀, dual assessment via additive (RERI) and multiplicative (IRR) interaction indices constructed with DLNM revealed synergistic and antagonistic effects between CO/O₃ and PM₁₀ in additive and multiplicative models, respectively. In contrast, PM₁₀-PM₂.₅ and PM₁₀-SO₂ interactions showed statistical significance only in additive models, with IRR values remaining non-significant. From an epidemiological modeling perspective, this phenomenon—additive interaction reaching significance while multiplicative interaction remains null—suggests that PM₂.₅ and SO₂ primarily exert independent dose-additive effects on CHD risk rather than true biological synergy mediated by receptor colocalization or metabolic competition. For instance, both PM₂.₅ and PM₁₀ are potent inducers of oxidative stress, driving the excessive generation of free radicals that inflict damage upon cell membranes, DNA, and proteins, while simultaneously provoking inflammatory cascades within the body (69). However, due to its relatively larger aerodynamic diameter, PM₁₀ is more effectively cleared via mucociliary mechanisms, resulting in prolonged retention within the upper respiratory tract. This extended residence time not only amplifies opportunities for co-exposure with other airborne pollutants but also renders its cardiovascular effects more indirect in nature. In contrast, PM₂.₅, with its finer particulate size and significantly larger reactive surface area, possesses the capacity to penetrate deep into the alveolar regions of the lungs and even translocate into systemic circulation (70). Once within the bloodstream, PM₂.₅ exerts profound cardiotoxic effects through the induction of chronic inflammation (71), oxidative stress (72, 73), and autonomic dysregulation (74, 75), culminating in a heightened risk of cardiovascular morbidity. From a policy and regulatory perspective, for pollutant combinations displaying simple additive interactions, risk mitigation strategies may be effectively implemented through precise source tracing and independent emission control, enabling a linear reduction in health risks.

In this study, the Spearman correlation analysis of pollutants revealed a notably high correlation of 0.87 between PM₁₀ and PM₂.₅, while the lowest correlation was observed between SO₂ and PM₂.₅ at 0.47. The correlations among other air pollutants hovered around 0.6, indicating a moderately elevated interdependence. It is worth noting that the Spearman correlation coefficient is best suited for describing monotonic relationships (76). However, only specific pollutant pairings exhibited statistically significant interactive effects. For instance, a global multicenter study demonstrated that (77), under high exposure levels, the synergy index of PM₂.₅ and O₃ in relation to cardiovascular mortality reached 1.37, synergistically increasing the relative risk of CHD hospitalization.

Subgroup analysis further indicated that younger individuals and males exhibited heightened susceptibility to the detrimental effects of NO₂, SO₂, and PM₁₀, as well as an intriguing protective effect of O₃ against CHD risk. Previous studies have suggested several plausible explanations for this observation. Firstly, younger individuals and men tend to engage in outdoor activities more frequently or work in highly polluted environments, leading to increased exposure and inadequate protective measures, thereby rendering them more vulnerable to acute effects during pollution surges (78). Secondly, lifestyle factors such as smoking, alcohol consumption, and irregular sleep patterns prevalent among these subgroups may exacerbate the cardiovascular impact of air pollution (79). Additionally, stress-induced activation of the sympathetic nervous system may further heighten cardiovascular strain (80). Furthermore, males may possess relatively weaker antioxidant capacities, rendering them more susceptible to oxidative stress induced by air pollution. Experimental evidence has demonstrated that (81), following identical infusions of angiotensin II, oxidative stress levels in male mice were significantly higher than in their female counterparts. Given that younger individuals and men typically exhibit higher levels of physical activity and respiratory rates, their inhalation of airborne pollutants is correspondingly greater, potentially predisposing them to both short-term and long-term health risks (82). Notably, this study also revealed that high concentrations of CO predominantly increased the risk of CHD among younger individuals (≤64y), underscoring the necessity of stringent air pollution control measures—particularly targeting CO emissions—in major metropolitan areas where young populations predominantly reside.

This study focuses on six major air pollutants in the Central Plains region of China, an area whose air pollution levels are emblematic of broader global patterns. The findings of this research offer insights that can be extrapolated to other nations or regions facing similar challenges. For instance, according to the World Health Organization, the air pollution levels in Henan Province—an industrial and traffic-intensive hub—are comparable to those found in emerging economies such as Bangladesh and India, yet notably higher than those in many developed nations. In contrast, rural areas or western regions of China exhibit relatively lower pollution levels, more akin to the suburban air quality found in developed countries. China's air pollution, distinct from that of other countries, is primarily driven by coal combustion, industrial emissions, and vehicle exhaust, with air quality management relying heavily on national policies and exhibiting a clear urban-rural divide.

The limitations of this study include: (1) In matching patients' residential addresses with air quality monitoring stations, we used the “nearest neighbor principle,” directly linking patients’ addresses to the geographically closest monitoring station without setting a 10-km radius or other distance threshold for screening. This may lead to limitations in exposure assessment due to the spatial coverage of monitoring stations, especially when patients live far from the nearest station, potentially causing bias in exposure estimates. (2) We used hospital admission dates as a proxy for symptom onset, which may result in temporal misalignment between pollution exposure and CHD onset, leading to estimation bias. (3) Although we adjusted for covariates such as temperature, humidity, day of the week, and holidays in the model, data limitations prevented us from including other potential confounders (e.g., sunshine duration, socioeconomic status, healthcare accessibility, comorbidities) as covariates to control for bias, which may affect the results to some extent.

## 5 Conclusions

The findings demonstrated that short-term exposure to elevated concentrations of NO₂, SO₂, and PM₁₀ in Henan Province significantly increased the risk of CHD hospitalizations, with cumulative effects peaking at a 7-day lag. Conversely, higher O₃ concentrations exhibited a negative correlation with CHD risk. Subgroup analyses revealed greater susceptibility among younger populations and males to air pollutants, while PM₂.₅ showed no significant associations across subgroups and CO only elevated CHD risk in younger demographics. Some pollutants interact significantly, with CO showing additive and multiplicative synergy with NO₂, SO₂, and PM₁₀, suggesting its adverse impact on CHD is amplified in a multi-pollutant environment. Future large-scale studies are warranted to validate these pollutant interaction mechanisms and strengthen causal inferences.

## Data Availability

The data analyzed in this study is subject to the following licenses/restrictions: the dataset used in this study is restricted to personnel from the Cardiac Center of The First Affiliated Hospital of Xinxiang Medical University, and its use requires the consent of the corresponding author. Access to the data is strictly controlled and requires appropriate authorization. In order to protect patient privacy, all data have been de-identified prior to analysis. The dataset is solely for research purposes and cannot be used for commercial or other non-research purposes. Additionally, the data cannot be shared with external parties without explicit permission, in accordance with institutional privacy policies. Requests to access these datasets should be directed to Shuming Liu, 3091929205@qq.com.
