# Supplementary material for: Short-term effects of extreme air pollutant concentrations on coronary heart disease hospitalization in Henan province: a time-stratified case-crossover study
Source: Front Cardiovasc Med. 2025 Apr 24;12:1538788. doi: 10.3389/fcvm.2025.1538788 (PMC12058883; doi:10.3389/fcvm.2025.1538788)
Supplement: Supplementary file 1 [file Datasheet1.pdf]

Supplementary Material

Contents

Supplementary Table 1: Coordinates of each city air monitoring station in the study area (P2)

Supplementary Table 2: Subgroup analysis by gender and age of cumulative lagged relative risks (RRs) and 95% confidence intervals (CIs) for the impact of extremely low air pollution concentrations on CHD hospitalizations in Henan Province, 2016–2021(P3)

Supplementary Table 3: Subgroup analysis by gender and age of cumulative lagged relative risks (RRs) and 95% confidence intervals (CIs) for the impact of extremely high air pollution concentrations on CHD hospitalizations in Henan Province, 2016–2021(P4)

Supplementary Table 4: Subgroup analysis by gender and age of single-day lagged relative risks (RRs) and 95% confidence intervals (CIs) for the impact of extremely low air pollution concentrations on CHD hospitalizations in Henan Province, 2016–2021 (P5)

Supplementary Table 5: Subgroup analysis by gender and age of single-day lagged relative risks (RRs) and 95% confidence intervals (CIs) for the impact of extremely high air pollution concentrations on CHD hospitalizations in Henan Province, 2016–2021 (P6)

Supplementary Table 6-Table 37: Sensitivity analysis of subgroup analysis by gender and age of cumulative lagged relative risks (RRs) and 95% confidence intervals (CIs) for the impact of extreme air pollution concentrations on CHD hospitalizations in Henan Province, 2016–2021 (P7-P46)

Supplementary Table 38-Table 55: Sensitivity analysis of relative risks (RRs) and 95% confidence intervals (CIs) of coronary heart disease hospitalizations associated with extreme air pollution concentrations of each pollutant in Henan Province from 2016 to 2021 (P47-P64)

Supplementary Table 56-Table 64: Sensitivity analysis of interaction analysis between various air pollutants and coronary heart disease hospitalizations in Henan Province from 2016 to 2021 (P65-P73)

Supplementary Table 1 Coordinates of each city air monitoring station in the study area

City	City code	Station code	Longitude, N	Latitude, E
Xinxiang	410700	2389A	113.836	35.310
		2390A	113.884	35.272
		2391A	113.883	35.303
		3054A	113.919	35.285
		3476A	113.914	35.333
		3689A	113.900	35.372
Nanyang	411300	2403A	112.519	32.992
		2404A	112.522	33.012
		2406A	112.557	33.027
		2407A	112.550	32.968
Anyang	410500	1818A	114.484	36.062
		1819A	114.355	36.102
		1820A	114.358	36.087
		3141A	114.392	36.088
		3669A	114.471	36.064
		3703A	114.292	36.095
Kaifeng	410200	1824A	114.339	34.778
		1826A	114.289	34.797
		3147A	114.344	34.811
		3210A	114.364	34.796
		3473A	114.249	34.831
		3592A	114.464	34.756
Luoyang	410300	1812A	112.394	34.651
		1814A	112.466	34.687
		1815A	112.494	34.689
		1817A	112.428	34.626
		3341A	112.495	34.633
		3636A	112.439	34.678

Supplementary Table 2 Subgroup analysis by gender and age of cumulative lagged relative risks (RRs) and 95% confidence intervals (CIs) for the impact of extremely low air pollution concentrations on CHD hospitalizations in Henan Province, 2016–2021.

pollutant	group	Lag00	Lag01	Lag02	Lag03	Lag04	Lag05	Lag06	Lag07
		RR 95% CI	RR 95% CI	RR 95% CI	RR 95% CI	RR 95% CI	RR 95% CI	RR 95% CI	RR 95% CI
**CO**	Male	0.995(0.977,1.014)	0.993(0.965,1.021)	0.991(0.959,1.024)	0.992(0.957,1.027)	0.993(0.957,1.031)	0.996(0.958,1.035)	1.000(0.963,1.038)	1.005(0.969,1.042)
	Female	0.988(0.970,1.007)	0.985(0.957,1.014)	0.989(0.957,1.023)	0.997(0.962,1.033)	1.006(0.968,1.045)	1.010(0.972,1.051)	1.009(0.971,1.048)	0.999(0.962,1.037)
	Young	1.001(0.983,1.020)	0.996(0.968,1.024)	0.984(0.953,1.017)	0.969(0.936,1.003)	**0.953(0.918,0.989)**	**0.939(0.904,0.975)**	**0.929(0.895,0.964)**	**0.923(0.891,0.957)**
	Old	**0.981(0.963,0.999)**	0.974(0.947,1.002)	0.979(0.947,1.012)	0.991(0.957,1.027)	1.007(0.969,1.046)	1.020(0.980,1.060)	1.026(0.988,1.066)	1.025(0.988,1.064)
**$PM_{2.5}$**	Male	0.995(0.983,1.007)	0.991(0.971,1.011)	0.987(0.964,1.012)	0.985(0.957,1.013)	0.983(0.951,1.015)	0.981(0.946,1.018)	0.980(0.943,1.019)	0.980(0.942,1.021)
	Female	0.989(0.977,1.002)	0.982(0.963,1.002)	0.978(0.954,1.003)	0.976(0.948,1.004)	0.975(0.943,1.008)	0.974(0.939,1.011)	0.972(0.935,1.011)	0.970(0.931,1.010)
	Young	1.000(0.988,1.013)	0.997(0.977,1.018)	0.991(0.967,1.016)	0.983(0.956,1.012)	0.975(0.944,1.008)	0.969(0.935,1.005)	0.966(0.930,1.003)	0.965(0.928,1.005)
	Old	0.989(0.977,1.001)	0.984(0.965,1.003)	0.984(0.960,1.008)	0.987(0.959,1.015)	0.992(0.960,1.025)	0.996(0.960,1.034)	0.998(0.960,1.038)	0.997(0.957,1.039)
**PM_{10}**	Male	**0.983(0.968,0.999)**	**0.962(0.938,0.988)**	**0.938(0.908,0.969)**	**0.912(0.878,0.948)**	**0.887(0.848,0.929)**	**0.866(0.823,0.911)**	**0.848(0.804,0.896)**	**0.836(0.790,0.885)**
	Female	**0.980(0.964,0.996)**	**0.960(0.935,0.986)**	**0.941(0.910,0.973)**	**0.922(0.886,0.959)**	**0.903(0.863,0.946)**	**0.885(0.840,0.932)**	**0.866(0.819,0.915)**	**0.847(0.799,0.897)**
	Young	0.993(0.976,1.010)	0.976(0.949,1.003)	**0.950(0.919,0.983)**	**0.921(0.885,0.958)**	**0.891(0.850,0.933)**	**0.865(0.821,0.911)**	**0.845(0.800,0.893)**	**0.833(0.787,0.882)**
	Old	**0.979(0.964,0.994)**	**0.961(0.937,0.986)**	**0.947(0.917,0.977)**	**0.935(0.900,0.971)**	**0.923(0.883,0.965)**	**0.911(0.867,0.958)**	**0.898(0.852,0.948)**	**0.884(0.835,0.935)**
**SO_2**	Male	1.004(0.988,1.019)	0.995(0.972,1.019)	0.976(0.950,1.004)	**0.950(0.923,0.979)**	**0.922(0.893,0.951)**	**0.895(0.866,0.924)**	**0.871(0.845,0.899)**	**0.853(0.827,0.879)**
	Female	1.000(0.984,1.016)	0.991(0.967,1.015)	0.974(0.947,1.002)	**0.952(0.924,0.981)**	**0.927(0.898,0.957)**	**0.902(0.873,0.932)**	**0.879(0.852,0.907)**	**0.858(0.832,0.885)**
	Young	0.999(0.984,1.014)	0.984(0.961,1.007)	**0.959(0.933,0.985)**	**0.926(0.900,0.953)**	**0.892(0.865,0.919)**	**0.860(0.834,0.887)**	**0.834(0.809,0.859)**	**0.814(0.790,0.838)**
	Old	1.009(0.993,1.025)	1.010(0.985,1.035)	1.004(0.975,1.033)	0.992(0.962,1.023)	0.976(0.945,1.009)	**0.959(0.927,0.991)**	**0.941(0.911,0.972)**	**0.924(0.894,0.954)**
**NO_2**	Male	0.996(0.970,1.022)	0.978(0.939,1.020)	**0.950(0.904,1.000)**	**0.915(0.864,0.970)**	**0.878(0.821,0.938)**	**0.842(0.782,0.906)**	**0.809(0.749,0.875)**	**0.782(0.721,0.847)**
	Female	0.994(0.968,1.020)	0.978(0.937,1.020)	0.954(0.906,1.004)	**0.924(0.871,0.980)**	**0.891(0.833,0.953)**	**0.858(0.796,0.924)**	**0.825(0.763,0.892)**	**0.795(0.732,0.862)**
	Young	1.002(0.976,1.029)	0.987(0.946,1.029)	0.957(0.909,1.007)	**0.917(0.865,0.973)**	**0.874(0.817,0.934)**	**0.832(0.773,0.896)**	**0.795(0.735,0.859)**	**0.763(0.703,0.827)**
	Old	0.991(0.966,1.016)	0.974(0.935,1.014)	**0.950(0.905,0.999)**	**0.922(0.871,0.977)**	**0.892(0.835,0.953)**	**0.862(0.801,0.927)**	**0.832(0.771,0.899)**	**0.805(0.742,0.872)**
**O_3**	Male	**1.140(1.098,1.184)**	**1.303(1.227,1.384)**	**1.492(1.388,1.604)**	**1.706(1.572,1.852)**	**1.940(1.769,2.127)**	**2.183(1.973,2.415)**	**2.423(2.179,2.695)**	**2.651(2.371,2.963)**
	Female	**1.103(1.061,1.147)**	**1.242(1.168,1.321)**	**1.421(1.320,1.531)**	**1.641(1.508,1.784)**	**1.888(1.717,2.075)**	**2.140(1.929,2.374)**	**2.372(2.127,2.645)**	**2.560(2.285,2.869)**
	Young	**1.130(1.086,1.175)**	**1.282(1.204,1.364)**	**1.458(1.354,1.571)**	**1.658(1.525,1.803)**	**1.875(1.707,2.059)**	**2.098(1.893,2.324)**	**2.315(2.079,2.577)**	**2.515(2.248,2.813)**
	Old	**1.092(1.052,1.133)**	**1.209(1.140,1.283)**	**1.355(1.261,1.455)**	**1.526(1.407,1.656)**	**1.715(1.563,1.881)**	**1.904(1.720,2.108)**	**2.079(1.867,2.315)**	**2.224(1.987,2.489)**

Supplementary Table 3 Subgroup analysis by gender and age of cumulative lagged relative risks (RRs) and 95% confidence intervals (CIs) for the impact of extremely high air pollution concentrations on CHD hospitalizations in Henan Province, 2016–2021.

pollutant	group	Lag00	Lag01	Lag02	Lag03	Lag04	Lag05	Lag06	Lag07
		RR 95% CI	RR 95% CI	RR 95% CI	RR 95% CI	RR 95% CI	RR 95% CI	RR 95% CI	RR 95% CI
**CO**	Male	1.022(0.938,1.112)	1.036(0.907,1.182)	1.042(0.894,1.214)	1.040(0.883,1.225)	1.033(0.867,1.230)	1.019(0.851,1.220)	1.001(0.841,1.191)	0.978(0.824,1.160)
	Female	1.056(0.968,1.151)	1.071(0.937,1.225)	1.052(0.901,1.228)	1.013(0.858,1.195)	0.973(0.815,1.162)	0.953(0.793,1.144)	0.961(0.805,1.147)	1.006(0.846,1.196)
	Young	0.993(0.913,1.081)	1.019(0.894,1.162)	1.076(0.925,1.253)	1.158(0.985,1.362)	**1.252(1.053,1.488)**	**1.342(1.123,1.603)**	**1.411(1.188,1.675)**	**1.450(1.225,1.716)**
	Old	**1.096(1.006,1.193)**	1.130(0.990,1.289)	1.104(0.948,1.287)	1.041(0.883,1.227)	0.969(0.812,1.156)	0.913(0.761,1.097)	0.885(0.742,1.057)	0.890(0.748,1.060)
**$PM_{2.5}$**	Male	1.030(0.958,1.107)	1.055(0.940,1.185)	1.077(0.934,1.243)	1.095(0.928,1.293)	1.109(0.915,1.343)	1.118(0.903,1.383)	1.123(0.896,1.407)	1.123(0.886,1.423)
	Female	1.064(0.989,1.145)	1.110(0.987,1.249)	1.139(0.985,1.316)	1.153(0.975,1.364)	1.160(0.956,1.407)	1.166(0.940,1.447)	1.178(0.938,1.480)	1.197(0.943,1.520)
	Young	0.998(0.926,1.074)	1.016(0.903,1.144)	1.054(0.912,1.218)	1.104(0.935,1.305)	1.158(0.957,1.402)	1.202(0.973,1.485)	1.228(0.982,1.534)	1.229(0.973,1.551)
	Old	1.067(0.994,1.145)	1.100(0.982,1.234)	1.102(0.956,1.270)	1.080(0.914,1.276)	1.049(0.865,1.273)	1.024(0.824,1.271)	1.011(0.803,1.273)	1.016(0.798,1.293)
**PM_{10}**	Male	**1.062(1.002,1.125)**	**1.148(1.045,1.260)**	**1.258(1.119,1.414)**	**1.390(1.210,1.597)**	**1.535(1.305,1.806)**	**1.677(1.398,2.013)**	**1.803(1.485,2.188)**	**1.900(1.549,2.330)**
	Female	**1.076(1.014,1.142)**	**1.158(1.052,1.274)**	**1.245(1.104,1.402)**	**1.338(1.161,1.542)**	**1.439(1.219,1.700)**	**1.551(1.287,1.869)**	**1.676(1.375,2.044)**	**1.816(1.474,2.237)**
	Young	1.026(0.965,1.091)	1.092(0.989,1.205)	**1.200(1.062,1.355)**	**1.346(1.166,1.553)**	**1.515(1.283,1.789)**	**1.684(1.399,2.026)**	**1.827(1.502,2.222)**	**1.924(1.566,2.364)**
	Old	**1.080(1.021,1.142)**	**1.152(1.052,1.261)**	**1.216(1.085,1.362)**	**1.274(1.113,1.460)**	**1.332(1.135,1.564)**	**1.395(1.166,1.671)**	**1.469(1.213,1.780)**	**1.557(1.273,1.906)**
**SO_2**	Male	0.974(0.871,1.089)	1.035(0.871,1.231)	1.188(0.973,1.451)	**1.443(1.166,1.786)**	**1.798(1.435,2.253)**	**2.229(1.770,2.807)**	**2.694(2.159,3.362)**	**3.139(2.521,3.910)**
	Female	1.001(0.894,1.121)	1.068(0.896,1.273)	1.207(0.985,1.478)	**1.426(1.148,1.770)**	**1.726(1.373,2.169)**	**2.099(1.661,2.652)**	**2.531(2.022,3.168)**	**3.004(2.406,3.752)**
	Young	1.009(0.906,1.125)	1.121(0.948,1.327)	**1.355(1.116,1.645)**	**1.736(1.412,2.135)**	**2.279(1.831,2.837)**	**2.956(2.362,3.698)**	**3.697(2.982,4.583)**	**4.405(3.562,5.448)**
	Old	0.938(0.836,1.520)	0.931(0.779,1.114)	0.973(0.791,1.197)	1.061(0.850,1.325)	1.190(0.941,1.506)	**1.354(1.064,1.724)**	**1.549(1.227,1.956)**	**1.773(1.406,2.236)**
**NO_2**	Male	1.010(0.951,1.073)	1.052(0.956,1.159)	**1.126(1.001,1.267)**	**1.229(1.073,1.408)**	**1.355(1.160,1.583)**	**1.494(1.259,1.774)**	**1.637(1.367,1.962)**	**1.777(1.471,2.146)**
	Female	1.015(0.955,1.080)	1.054(0.955,1.163)	1.117(0.991,1.258)	**1.202(1.048,1.380)**	**1.308(1.118,1.531)**	**1.430(1.202,1.702)**	**1.565(1.304,1.879)**	**1.710(1.414,2.070)**
	Young	0.996(0.936,1.059)	1.032(0.935,1.139)	1.108(0.983,1.249)	**1.224(1.066,1.404)**	**1.370(1.171,1.603)**	**1.536(1.293,1.826)**	**1.710(1.427,2.050)**	**1.882(1.558,2.272)**
	Old	1.022(0.964,1.084)	1.064(0.968,1.169)	**1.126(1.003,1.263)**	**1.207(1.056,1.380)**	**1.305(1.119,1.521)**	**1.415(1.194,1.677)**	**1.535(1.283,1.836)**	**1.661(1.377,2.003)**
**O_3**	Male	**0.817(0.771,0.866)**	**0.664(0.605,0.729)**	**0.538(0.481,0.602)**	**0.438(0.386,0.497)**	**0.359(0.311,0.414)**	**0.299(0.256,0.350)**	**0.254(0.216,0.300)**	**0.221(0.186,0.263)**
	Female	**0.859(0.809,0.912)**	**0.715(0.650,0.787)**	**0.581(0.518,0.651)**	**0.465(0.408,0.530)**	**0.374(0.323,0.433)**	**0.308(0.263,0.362)**	**0.263(0.222,0.311)**	**0.234(0.196,0.279)**
	Young	**0.828(0.779,0.880)**	**0.681(0.619,0.750)**	**0.558(0.497,0.626)**	**0.457(0.402,0.521)**	**0.378(0.327,0.437)**	**0.318(0.271,0.373)**	**0.273(0.231,0.322)**	**0.240(0.202,0.286)**
	Old	**0.873(0.825,0.924)**	**0.746(0.681,0.817)**	**0.625(0.560,0.699)**	**0.520(0.458,0.590)**	**0.434(0.377,0.501)**	**0.369(0.316,0.432)**	**0.322(0.273-0.381)**	**0.291(0.244,0.346)**

Supplementary Table 4 Subgroup analysis by gender and age of single-day lagged relative risks (RRs) and 95% confidence intervals (CIs) for the impact of extremely low air pollution concentrations on CHD hospitalizations in Henan Province, 2016–2021

pollutant	group	Lag0	Lag1	Lag2	Lag3	Lag4	Lag5	Lag6	Lag7
		RR 95% CI	RR 95% CI	RR 95% CI	RR 95% CI	RR 95% CI	RR 95% CI	RR 95% CI	RR 95% CI
**CO**	Male	0.995(0.977,1.014)	0.997(0.986,1.008)	0.999(0.990,1.007)	1.000(0.990,1.011)	1.002(0.991,1.013)	1.003(0.994,1.012)	1.004(0.994,1.014)	1.005(0.988,1.022)
	Female	0.988(0.970,1.007)	0.997(0.986,1.008)	1.004(0.995,1.013)	1.008(0.997,1.019)	1.008(0.997,1.020)	1.005(0.995,1.014)	0.998(0.988,1.009)	0.990(0.974,1.007)
	Young	1.001(0.983,1.020)	0.994(0.984,1.005)	**0.988(0.980,0.997)**	**0.984(0.974,0.995)**	**0.983(0.973,0.994)**	**0.985(0.976,0.994)**	**0.989(0.979,0.999)**	0.994(0.978,1.011)
	Old	**0.981(0.963,0.999)**	0.994(0.983,1.004)	1.005(0.996,1.014)	**1.013(1.002,1.024)**	**1.015(1.004,1.027)**	**1.013(1.004,1.022)**	1.007(0.996,1.017)	0.999(0.982,1.016)
**$PM_{2.5}$**	Male	0.995(0.983,1.007)	0.996(0.988,1.004)	0.996(0.989,1.004)	0.997(0.989,1.006)	0.998(0.989,1.007)	0.999(0.991,1.006)	0.999(0.991,1.007)	1.000(0.989,1.011)
	Female	0.989(0.977,1.002)	0.993(0.985,1.001)	0.996(0.988,1.003)	0.998(0.989,1.007)	0.999(0.990,1.008)	0.999(0.991,1.007)	0.998(0.99,1.006)	0.997(0.986,1.009)
	Young	1.000(0.988,1.013)	0.997(0.989,1.005)	0.994(0.987,1.001)	0.992(0.983,1.001)	0.992(0.983,1.001)	0.994(0.986,1.001)	0.996(0.989,1.004)	1.000(0.988,1.011)
	Old	0.989(0.977,1.001)	0.995(0.987,1.003)	1.000(0.992,1.007)	1.003(0.995,1.012)	1.005(0.996,1.014)	1.004(0.996,1.012)	1.002(0.994,1.010)	0.999(0.988,1.010)
**PM_{10}**	Male	**0.983(0.968,0.999)**	**0.979(0.968,0.989)**	**0.975(0.965,0.985)**	**0.973(0.961,0.984)**	**0.973(0.961,0.985)**	**0.976(0.966,0.986)**	**0.980(0.969,0.991)**	0.985(0.970,1.002)
	Female	**0.980(0.964,0.996)**	**0.980(0.969,0.991)**	**0.980(0.970,0.990)**	**0.980(0.968,0.992)**	**0.980(0.968,0.992)**	**0.979(0.969,0.990)**	**0.979(0.968,0.990)**	**0.978(0.962,0.994)**
	Young	0.993(0.976,1.010)	**0.983(0.972,0.994)**	**0.974(0.964,0.984)**	**0.969(0.957,0.981)**	**0.968(0.956,0.980)**	**0.971(0.961,0.981)**	**0.977(0.967,0.989)**	0.986(0.969,1.002)
	Old	**0.979(0.964,0.994)**	**0.982(0.972,0.993)**	**0.985(0.975,0.995)**	**0.987(0.975,0.999)**	**0.988(0.976,1.000)**	**0.987(0.977,0.997)**	**0.986(0.975,0.996)**	**0.984(0.968,1.000)**
**SO_2**	Male	1.004(0.988,1.019)	0.992(0.983,1.001)	**0.981(0.974,0.988)**	**0.973(0.965,0.982)**	**0.970(0.961,0.979)**	**0.971(0.963,0.978)**	**0.974(0.965,0.983)**	**0.979(0.965,0.993)**
	Female	1.000(0.984,1.016)	**0.991(0.982,1.000)**	**0.983(0.976,0.990)**	**0.977(0.968,0.986)**	**0.974(0.965,0.983)**	**0.973(0.966,0.981)**	**0.974(0.966,0.983)**	**0.976(0.962,0.991)**
	Young	0.999(0.984,1.014)	**0.985(0.977,0.994)**	**0.974(0.967,0.981)**	**0.966(0.958,0.975)**	**0.963(0.954,0.972)**	**0.965(0.957,0.972)**	**0.969(0.961,0.978)**	**0.976(0.962,0.990)**
	Old	1.009(0.993,1.025)	1.001(0.992,1.011)	0.994(0.986,1.001)	**0.988(0.979,0.997)**	**0.984(0.975,0.993)**	**0.982(0.974,0.990)**	**0.981(0.973,0.991)**	**0.981(0.967,0.996)**
**NO_2**	Male	0.996(0.970,1.022)	**0.983(0.966,0.999)**	**0.972(0.957,0.986)**	**0.963(0.946,0.981)**	**0.959(0.942,0.977)**	**0.959(0.944,0.974)**	**0.962(0.946,0.978)**	**0.966(0.943,0.989)**
	Female	0.994(0.968,1.020)	0.984(0.968,1.001)	**0.976(0.961,0.991)**	**0.969(0.951,0.986)**	**0.964(0.947,0.982)**	**0.962(0.947,0.978)**	**0.962(0.946,0.978)**	**0.963(0.939,0.987)**
	Young	1.002(0.976,1.029)	0.985(0.968,1.002)	**0.970(0.955,0.985)**	**0.959(0.941,0.976)**	**0.953(0.935,0.970)**	**0.952(0.937,0.967)**	**0.955(0.939,0.971)**	**0.960(0.936,0.984)**
	Old	0.991(0.966,1.016)	**0.983(0.967,0.999)**	**0.976(0.962,0.991)**	**0.971(0.954,0.988)**	**0.967(0.950,0.985)**	**0.966(0.951,0.981)**	**0.966(0.950,0.982)**	**0.967(0.944,0.990)**
**O_3**	Male	**1.140(1.098,1.184)**	**1.143(1.116,1.171)**	**1.145(1.122,1.169)**	**1.144(1.116,1.172)**	**1.137(1.109,1.166)**	**1.125(1.101,1.150)**	**1.110(1.086,1.135)**	**1.094(1.059,1.130)**
	Female	**1.103(1.061,1.147)**	**1.126(1.099,1.154)**	**1.144(1.120,1.169)**	**1.154(1.126,1.183)**	**1.151(1.121,1.181)**	**1.134(1.109,1.159)**	**1.108(1.083,1.134)**	**1.079(1.044,1.116)**
	Young	**1.130(1.086,1.175)**	**1.135(1.107,1.163)**	**1.138(1.114,1.162)**	**1.137(1.109,1.166)**	**1.131(1.102,1.160)**	**1.119(1.094,1.144)**	**1.103(1.078,1.129)**	**1.086(1.050,1.124)**
	Old	**1.092(1.052,1.133)**	**1.108(1.082,1.134)**	**1.120(1.098,1.144)**	**1.127(1.100,1.154)**	**1.123(1.096,1.152)**	**1.111(1.087,1.135)**	**1.092(1.068,1.116)**	**1.070(1.036,1.105)**

Supplementary Table 5 Subgroup analysis by gender and age of single-day lagged relative risks (RRs) and 95% confidence intervals (CIs) for the impact of extremely high air pollution concentrations on CHD hospitalizations in Henan Province, 2016–2021

pollutant	group	Lag0	Lag1	Lag2	Lag3	Lag4	Lag5	Lag6	Lag7
		RR 95% CI	RR 95% CI	RR 95% CI	RR 95% CI	RR 95% CI	RR 95% CI	RR 95% CI	RR 95% CI
**CO**	Male	1.022(0.938,1.112)	1.014(0.964,1.066)	1.006(0.966,1.048)	0.999(0.950,1.050)	0.992(0.943,1.044)	0.987(0.946,1.030)	0.982(0.936,1.030)	0.977(0.904,1.056)
	Female	1.056(0.968,1.151)	1.015(0.964,1.068)	0.982(0.942,1.023)	0.963(0.915,1.013)	0.961(0.913,1.013)	0.978(0.937,1.022)	1.009(0.961,1.059)	1.047(0.967,1.132)
	Young	0.993(0.913,1.081)	1.026(0.977,1.079)	**1.056(1.014,1.099)**	**1.076(1.024,1.130)**	**1.081(1.028,1.137)**	**1.071(1.027,1.118)**	**1.052(1.003,1.103)**	1.028(0.952,1.110)
	Old	**1.096(1.006,1.193)**	1.031(0.980,1.084)	0.978(0.938,1.019)	0.943(0.896,0.991)	**0.931(0.884,0.980)**	**0.942(0.903,0.984)**	0.969(0.924,1.017)	1.005(0.930,1.087)
**$PM_{2.5}$**	Male	1.030(0.958,1.107)	1.025(0.978,1.075)	1.021(0.978,1.066)	1.017(0.966,1.070)	1.012(0.961,1.066)	1.008(0.964,1.055)	1.004(0.959,1.052)	1.000(0.936,1.069)
	Female	1.064(0.989,1.145)	1.043(0.995,1.095)	1.026(0.982,1.071)	1.013(0.962,1.066)	1.006(0.954,1.060)	1.006(0.961,1.053)	1.010(0.964,1.058)	1.016(0.950,1.087)
	Young	0.998(0.926,1.074)	1.019(0.971,1.069)	1.037(0.993,1.082)	1.048(0.996,1.103)	1.048(0.995,1.105)	1.038(0.992,1.086)	1.021(0.975,1.07)	1.001(0.935,1.071)
	Old	1.067(0.994,1.145)	1.031(0.984,1.081)	1.001(0.958,1.046)	0.980(0.931,1.032)	0.972(0.922,1.024)	0.976(0.932,1.021)	0.988(0.943,1.034)	1.004(0.941,1.072)
**PM_{10}**	Male	**1.062(1.002,1.125)**	**1.081(1.040,1.123)**	**1.096(1.056,1.138)**	**1.105(1.058,1.154)**	**1.104(1.057,1.153)**	**1.093(1.053,1.134)**	**1.075(1.034,1.118)**	1.054(0.994,1.117)
	Female	**1.076(1.014,1.142)**	**1.076(1.034,1.119)**	**1.075(1.035,1.116)**	**1.075(1.028,1.124)**	**1.076(1.029,1.125)**	**1.078(1.038,1.120)**	**1.080(1.038,1.124)**	**1.083(1.021,1.150)**
	Young	1.026(0.965,1.091)	**1.065(1.022,1.109)**	**1.099(1.058,1.141)**	**1.121(1.073,1.173)**	**1.126(1.077,1.177)**	**1.111(1.070,1.155)**	**1.085(1.042,1.130)**	1.053(0.992,1.119)
	Old	**1.080(1.021,1.142)**	**1.067(1.027,1.108)**	**1.056(1.018,1.095)**	**1.048(1.004,1.094)**	**1.045(1.001,1.091)**	**1.047(1.010,1.087)**	**1.053(1.013,1.094)**	**1.060(1.001,1.122)**
**SO_2**	Male	0.974(0.871,1.089)	1.063(0.995,1.135)	**1.148(1.090,1.209)**	**1.215(1.140,1.294)**	**1.246(1.167,1.330)**	**1.240(1.173,1.310)**	**1.208(1.134,1.288)**	**1.165(1.051,1.292)**
	Female	1.001(0.894,1.121)	1.067(0.998,1.141)	**1.130(1.072,1.192)**	**1.181(1.107,1.260)**	**1.211(1.133,1.293)**	**1.216(1.150,1.286)**	**1.206(1.130,1.286)**	**1.187(1.069,1.318)**
	Young	1.009(0.906,1.125)	**1.111(1.042,1.184)**	**1.208(1.148,1.271)**	**1.282(1.204,1.364)**	**1.313(1.231,1.399)**	**1.297(1.229,1.369)**	**1.251(1.175,1.331)**	**1.192(1.077,1.319)**
	Old	0.938(0.836,1.520)	0.993(0.927,1.063)	1.045(0.990,1.103)	**1.090(1.021,1.164)**	**1.122(1.049,1.200)**	**1.138(1.075,1.205)**	**1.144(1.071,1.222)**	**1.144(1.030,1.272)**
**NO_2**	Male	1.010(0.951,1.073)	**1.041(1.002,1.083)**	**1.070(1.033,1.108)**	**1.092(1.047,1.138)**	**1.103(1.057,1.150)**	**1.103(1.063,1.144)**	**1.096(1.054,1.139)**	**1.085(1.025,1.148)**
	Female	1.015(0.955,1.080)	1.038(0.998,1.080)	**1.059(1.022,1.098)**	**1.077(1.032,1.123)**	**1.088(1.042,1.136)**	**1.093(1.053,1.135)**	**1.094(1.052,1.138)**	**1.093(1.032,1.157)**
	Young	0.996(0.936,1.059)	1.036(0.996,1.078)	**1.074(1.037,1.113)**	**1.104(1.058,1.152)**	**1.120(1.073,1.169)**	**1.121(1.080,1.164)**	**1.113(1.071,1.157)**	**1.100(1.039,1.166)**
	Old	1.022(0.964,1.084)	**1.041(1.002,1.081)**	**1.058(1.022,1.096)**	**1.072(1.029,1.117)**	**1.081(1.037,1.127)**	**1.084(1.046,1.125)**	**1.084(1.044,1.126)**	**1.082(1.024,1.144)**
**O_3**	Male	**0.817(0.771,0.866)**	**0.813(0.783,0.844)**	**0.811(0.786,0.837)**	**0.813(0.783,0.844)**	**0.820(0.789,0.852)**	**0.833(0.800,0.862)**	**0.851(0.822,0.881)**	**0.870(0.828,0.915)**
	Female	**0.859(0.809,0.912)**	**0.833(0.802,0.865)**	**0.812(0.786,0.839)**	**0.801(0.771,0.833)**	**0.805(0.774,0.838)**	**0.824(0.796,0.852)**	**0.853(0.823,0.884)**	**0.888(0.843,0.936)**
	Young	**0.828(0.779,0.880)**	**0.823(0.792,0.854)**	**0.819(0.793,0.846)**	**0.820(0.789,0.852)**	**0.827(0.795,0.860)**	**0.841(0.812,0.870)**	**0.859(0.829,0.890)**	**0.880(0.835,0.927)**
	Old	**0.873(0.825,0.924)**	**0.854(0.823,0.886)**	**0.839(0.813,0.866)**	**0.832(0.801,0.863)**	**0.835(0.804,0.868)**	**0.850(0.822,0.879)**	**0.873(0.844,0.904)**	**0.901(0.857,0.947)**

Supplementary Table 6 Subgroup analysis by gender and age of cumulative lagged relative risks (RRs) and 95% confidence intervals (CIs) for the impact of extremely low air pollution concentrations on CHD hospitalizations in Henan Province, 2016–2021(the maximum lag period = 5)

pollutant	group	Lag00	Lag01	Lag02	Lag03	Lag04	Lag05
		RR 95% CI	RR 95% CI	RR 95% CI	RR 95% CI	RR 95% CI	RR 95% CI
**CO**	Male	0.994(0.976,1.012)	0.998(0.971,1.025)	1.007(0.976,1.040)	1.016(0.981,1.052)	1.016(0.981,1.053)	1.005(0.970,1.041)
	Female	0.984(0.967,1.003)	0.987(0.960,1.015)	1.002(0.970,1.035)	1.017(0.982,1.054)	1.021(0.985,1.058)	1.006(0.971,1.043)
	Young	0.996(0.978,1.014)	0.991(0.964,1.018)	0.983(0.953,1.015)	0.972(0.939,1.006)	0.956(0.923,0.990)	0.933(0.901,0.966)
	Old	0.980(0.962,0.997)	0.983(0.957,1.010)	1.003(0.972,1.036)	1.026(0.991,1.063)	1.036(0.999,1.074)	1.025(0.989,1.063)
**$PM_{2.5}$**	Male	0.994(0.982,1.007)	0.992(0.973,1.012)	0.992(0.967,1.018)	0.992(0.962,1.023)	0.990(0.957,1.025)	0.985(0.949,1.022)
	Female	0.988(0.976,1.000)	0.982(0.963,1.002)	0.982(0.957,1.007)	0.982(0.953,1.013)	0.981(0.948,1.016)	0.977(0.941,1.014)
	Young	0.999(0.986,1.011)	0.996(0.976,1.016)	0.992(0.967,1.018)	0.987(0.958,1.018)	0.981(0.948,1.015)	0.974(0.939,1.010)
	Old	0.989(0.977,1.001)	0.987(0.968,1.007)	0.992(0.967,1.017)	0.999(0.968,1.030)	1.001(0.967,1.037)	0.998(0.961,1.036)
**PM_{10}**	Male	0.981(0.965,0.997)	0.956(0.932,0.981)	0.928(0.897,0.961)	0.901(0.864,0.940)	0.879(0.838,0.923)	0.864(0.821,0.910)
	Female	0.977(0.961,0.993)	0.952(0.927,0.978)	0.929(0.897,0.962)	0.908(0.870,0.948)	0.892(0.849,0.937)	0.881(0.836,0.929)
	Young	0.989(0.972,1.006)	0.966(0.940,0.993)	0.936(0.904,0.970)	0.906(0.867,0.946)	0.881(0.839,0.925)	0.865(0.821,0.911)
	Old	0.978(0.962,0.993)	0.958(0.934,0.983)	0.942(0.911,0.974)	0.928(0.890,0.968)	0.917(0.874,0.961)	0.907(0.862,0.954)
**SO_2**	Male	0.998(0.983,1.014)	0.978(0.956,1.001)	0.946(0.921,0.971)	0.912(0.886,0.938)	0.885(0.859,0.911)	0.869(0.844,0.894)
	Female	0.996(0.980,1.012)	0.973(0.951,0.996)	0.940(0.915,0.965)	0.906(0.880,0.933)	0.883(0.857,0.909)	0.874(0.849,0.900)
	Young	0.993(0.978,1.008)	0.964(0.943,0.985)	0.921(0.898,0.945)	0.879(0.855,0.904)	0.848(0.824,0.872)	0.832(0.809,0.856)
	Old	1.005(0.990,1.021)	0.997(0.974,1.021)	0.980(0.953,1.007)	0.960(0.931,0.989)	0.944(0.915,0.973)	0.935(0.907,0.964)
**NO_2**	Male	0.987(0.962,1.013)	0.957(0.919,0.997)	0.917(0.871,0.967)	0.877(0.824,0.934)	0.846(0.790,0.907)	0.827(0.768,0.891)
	Female	0.983(0.957,1.009)	0.957(0.918,0.997)	0.925(0.878,0.976)	0.894(0.839,0.953)	0.867(0.808,0.930)	0.847(0.786,0.913)
	Young	0.990(0.964,1.017)	0.957(0.918,0.998)	0.911(0.864,0.960)	0.865(0.812,0.922)	0.833(0.777,0.893)	0.818(0.760,0.881)
	Old	0.983(0.959,1.008)	0.960(0.922,0.999)	0.933(0.886,0.982)	0.904(0.850,0.962)	0.876(0.818,0.938)	0.849(0.789,0.914)
**O_3**	Male	1.168(1.124,1.213)	1.363(1.285,1.445)	1.584(1.473,1.703)	1.823(1.673,1.985)	2.062(1.875,2.267)	2.285(2.064,2.531)
	Female	1.126(1.083,1.171)	1.291(1.215,1.371)	1.497(1.390,1.612)	1.737(1.591,1.895)	1.995(1.810,2.198)	2.255(2.031,2.503)
	Young	1.153(1.109,1.200)	1.337(1.259,1.421)	1.551(1.440,1.671)	1.781(1.633,1.944)	2.006(1.822,2.209)	2.204(1.988,2.443)
	Old	1.114(1.073,1.157)	1.247(1.177,1.321)	1.399(1.303,1.504)	1.573(1.444,1.712)	1.765(1.605,1.942)	1.978(1.785,2.192)

Supplementary Table 7 Subgroup analysis by gender and age of single-day lagged relative risks (RRs) and 95% confidence intervals (CIs) for the impact of extremely low air pollution concentrations on CHD hospitalizations in Henan Province, 2016–2021(the maximum lag period = 5)

pollutant	group	Lag0	Lag1	Lag2	Lag3	Lag4	Lag5
		RR 95% CI	RR 95% CI	RR 95% CI	RR 95% CI	RR 95% CI	RR 95% CI
**CO**	Male	0.994(0.976,1.012)	1.004(0.993,1.014)	1.010(0.999,1.020)	1.008(0.997,1.020)	1.000(0.990,1.011)	0.989(0.972,1.005)
	Female	0.984(0.967,1.003)	1.003(0.992,1.014)	1.015(1.004,1.027)	1.015(1.004,1.027)	1.003(0.993,1.014)	0.986(0.969,1.002)
	Young	0.996(0.978,1.014)	0.995(0.984,1.005)	0.993(0.982,1.003)	0.989(0.978,1.000)	0.983(0.973,0.993)	0.976(0.960,0.993)
	Old	0.980(0.962,0.997)	1.004(0.993,1.014)	1.021(1.009,1.032)	1.023(1.011,1.034)	1.010(0.999,1.020)	0.989(0.973,1.006)
**$PM_{2.5}$**	Male	0.994(0.982,1.007)	0.998(0.989,1.006)	1.000(0.991,1.009)	1.000(0.991,1.009)	0.998(0.989,1.006)	0.995(0.983,1.006)
	Female	0.988(0.976,1.000)	0.994(0.986,1.003)	0.999(0.990,1.009)	1.001(0.991,1.010)	0.999(0.990,1.008)	0.995(0.984,1.007)
	Young	0.999(0.986,1.011)	0.997(0.989,1.006)	0.996(0.987,1.006)	0.995(0.986,1.004)	0.994(0.985,1.002)	0.993(0.981,1.004)
	Old	0.989(0.977,1.001)	0.998(0.990,1.007)	1.005(0.996,1.014)	1.006(0.997,1.016)	1.003(0.994,1.011)	0.996(0.985,1.008)
**PM_{10}**	Male	0.981(0.965,0.997)	0.975(0.963,0.986)	0.971(0.958,0.983)	0.971(0.958,0.984)	0.976(0.964,0.987)	0.983(0.967,0.998)
	Female	0.997(0.961,0.993)	0.975(0.964,0.987)	0.975(0.962,0.989)	0.978(0.964,0.991)	0.982(0.970,0.994)	0.988(0.972,1.004)
	Young	0.989(0.972,1.006)	0.977(0.965,0.989)	0.969(0.956,0.982)	0.968(0.955,0.981)	0.973(0.961,0.984)	0.981(0.965,0.997)
	Old	0.978(0.962,0.993)	0.980(0.969,0.992)	0.983(0.970,0.996)	0.985(0.973,0.998)	0.987(0.976,0.999)	0.989(0.974,1.005)
**SO_2**	Male	0.998(0.983,1.014)	0.980(0.971,0.989)	0.967(0.958,0.976)	0.964(0.955,0.973)	0.970(0.962,0.979)	0.982(0.968,0.996)
	Female	0.996(0.980,1.012)	0.978(0.969,0.986)	0.965(0.956,0.974)	0.964(0.955,0.973)	0.974(0.966,0.983)	0.990(0.976,1.005)
	Young	0.993(0.978,1.008)	0.971(0.963,0.979)	0.956(0.948,0.965)	0.954(0.945,0.963)	0.965(0.956,0.973)	0.982(0.968,0.996)
	Old	1.005(0.990,1.021)	0.992(0.983,1.001)	0.982(0.973,0.992)	0.980(0.970,0.989)	0.983(0.975,0.992)	0.991(0.976,1.006)
**NO_2**	Male	0.987(0.962,1.013)	0.970(0.953,0.987)	0.958(0.940,0.977)	0.956(0.938,0.975)	0.964(0.948,0.982)	0.978(0.954,1.002)
	Female	0.983(0.957,1.009)	0.974(0.956,0.991)	0.967(0.949,0.986)	0.966(0.947,0.985)	0.970(0.953,0.988)	0.977(0.952,1.002)
	Young	0.990(0.964,1.017)	0.967(0.950,0.984)	0.951(0.933,0.970)	0.950(0.932,0.969)	0.963(0.946,0.980)	0.983(0.958,1.008)
	Old	0.983(0.959,1.008)	0.977(0.960,0.994)	0.972(0.954,0.991)	0.969(0.951,0.988)	0.969(0.952,0.986)	0.969(0.946,0.993)
**O_3**	Male	1.168(1.124,1.213)	1.167(1.139,1.196)	1.163(1.133,1.193)	1.151(1.120,1.182)	1.131(1.105,1.158)	1.108(1.073,1.146)
	Female	1.126(1.083,1.171)	1.146(1.118,1.175)	1.160(1.129,1.191)	1.160(1.129,1.193)	1.149(1.121,1.177)	1.130(1.093,1.169)
	Young	1.153(1.109,1.200)	1.160(1.131,1.189)	1.160(1.129,1.191)	1.148(1.117,1.181)	1.126(1.099,1.154)	1.099(1.062,1.137)
	Old	1.114(1.073,1.157)	1.119(1.092,1.146)	1.122(1.094,1.152)	1.124(1.094,1.154)	1.123(1.096,1.150)	1.120(1.085,1.157)

Supplementary Table 8 Subgroup analysis by gender and age of cumulative lagged relative risks (RRs) and 95% confidence intervals (CIs) for the impact of extremely high air pollution concentrations on CHD hospitalizations in Henan Province, 2016–2021(the maximum lag period = 5)

pollutant	group	Lag00	Lag01	Lag02	Lag03	Lag04	Lag05
		RR 95% CI	RR 95% CI	RR 95% CI	RR 95% CI	RR 95% CI	RR 95% CI
**CO**	Male	1.027(0.945,1.117)	1.010(0.890,1.146)	0.966(0.834,1.120)	0.929(0.790,1.093)	0.928(0.787,1.095)	0.979(0.830,1.154)
	Female	1.076(0.988,1.172)	1.062(0.935,1.207)	0.990(0.853,1.150)	0.922(0.782,1.088)	0.908(0.768,1.074)	0.972(0.822,1.149)
	Young	1.019(0.937,1.108)	1.045(0.922,1.185)	1.082(0.934,1.253)	1.141(0.971,1.341)	1.236(1.050,1.456)	1.382(1.175,1.625)
	Old	1.101(1.012,1.197)	1.082(0.954,1.228)	0.984(0.849,1.141)	0.887(0.753,1.045)	0.848(0.717,1.002)	0.891(0.753,1.053)
**$PM_{2.5}$**	Male	1.033(0.961,1.110)	1.047(0.933,1.174)	1.047(0.903,1.214)	1.047(0.876,1.253)	1.060(0.868,1.296)	1.094(0.882,1.356)
	Female	1.073(0.998,1.154)	1.109(0.987,1.246)	1.114(0.959,1.294)	1.109(0.925,1.329)	1.116(0.911,1.367)	1.146(0.922,1.424)
	Young	1.008(0.936,1.085)	1.023(0.910,1.150)	1.046(0.901,1.215)	1.078(0.901,1.289)	1.118(0.916,1.365)	1.168(0.944,1.445)
	Old	1.067(0.994,1.144)	1.078(0.962,1.207)	1.047(0.904,1.214)	1.009(0.843,1.208)	0.993(0.810,1.216)	1.014(0.815,1.261)
**PM_{10}**	Male	1.071(1.011,1.135)	1.174(1.069,1.289)	1.307(1.155,1.478)	1.453(1.248,1.691)	1.586(1.335,1.885)	1.688(1.403,2.031)
	Female	1.089(1.026,1.155)	1.191(1.082,1.310)	1.303(1.149,1.477)	1.413(1.210,1.650)	1.508(1.265,1.798)	1.576(1.305,1.905)
	Young	1.041(0.979,1.107)	1.131(1.025,1.247)	1.266(1.114,1.438)	1.425(1.219,1.666)	1.574(1.321,1.876)	1.685(1.397,2.032)
	Old	1.085(1.026,1.147)	1.165(1.064,1.275)	1.239(1.099,1.397)	1.306(1.125,1.516)	1.367(1.154,1.620)	1.421(1.184,1.706)
**SO_2**	Male	1.014(0.908,1.133)	1.174(0.996,1.383)	1.494(1.237,1.805)	1.947(1.584,2.393)	2.417(1.962,2.979)	2.756(2.235,3.399)
	Female	1.031(0.921,1.154)	1.213(1.027,1.434)	1.564(1.292,1.895)	2.033(1.650,2.505)	2.453(1.986,3.029)	2.633(2.129,3.256)
	Young	1.055(0.946,1.176)	1.305(1.112,1.531)	1.803(1.500,2.166)	2.530(2.071,3.091)	3.281(2.681,4.015)	3.744(3.057,4.586)
	Old	0.962(0.859,1.079)	1.020(0.861,1.208)	1.159(0.953,1.410)	1.345(1.085,1.668)	1.517(1.219,1.889)	1.621(1.299,2.022)
**NO_2**	Male	1.031(0.971,1.095)	1.107(1.006,1.218)	1.223(1.082,1.381)	1.357(1.172,1.570)	1.476(1.255,1.736)	1.556(1.310,1.849)
	Female	1.042(0.980,1.107)	1.109(1.006,1.222)	1.198(1.059,1.356)	1.299(1.120,1.506)	1.394(1.183,1.642)	1.473(1.237,1.753)
	Young	1.024(0.962,1.089)	1.108(1.004,1.221)	1.244(1.099,1.408)	1.402(1.210,1.625)	1.533(1.302,1.804)	1.596(1.343,1.897)
	Old	1.041(0.982,1.104)	1.100(1.002,1.207)	1.175(1.043,1.325)	1.264(1.095,1.460)	1.361(1.160,1.598)	1.464(1.234,1.736)
**O_3**	Male	0.787(0.742,0.835)	0.620(0.566,0.679)	0.491(0.439,0.549)	0.395(0.346,0.451)	0.327(0.282,0.378)	0.279(0.238,0.326)
	Female	0.832(0.783,0.884)	0.674(0.614,0.740)	0.536(0.478,0.601)	0.426(0.372,0.488)	0.344(0.296,0.399)	0.284(0.242,0.334)
	Young	0.802(0.754,0.853)	0.638(0.581,0.701)	0.507(0.452,0.569)	0.409(0.358,0.469)	0.341(0.294,0.395)	0.295(0.251,0.346)
	Old	0.846(0.798,0.896)	0.711(0.650,0.778)	0.595(0.532,0.664)	0.497(0.435,0.566)	0.415(0.358,0.481)	0.348(0.297,0.408)

Supplementary Table 9 Subgroup analysis by gender and age of single-day lagged relative risks (RRs) and 95% confidence intervals (CIs) for the impact of extremely high air pollution concentrations on CHD hospitalizations in Henan Province, 2016–2021(the maximum lag period = 5)

pollutant	group	Lag0	Lag1	Lag2	Lag3	Lag4	Lag5
		RR 95% CI	RR 95% CI	RR 95% CI	RR 95% CI	RR 95% CI	RR 95% CI
**CO**	Male	1.027(0.945,1.117)	0.983(0.935,1.033)	0.957(0.909,1.007)	0.962(0.913,1.013)	0.999(0.951,1.049)	1.055(0.975,1.140)
	Female	1.076(0.988,1.172)	0.987(0.939,1.038)	0.932(0.885,0.982)	0.931(0.883,0.982)	0.984(0.936,1.035)	1.070(0.989,1.158)
	Young	1.019(0.937,1.108)	1.025(0.976,1.077)	1.035(0.984,1.089)	1.055(1.002,1.111)	1.083(1.032,1.137)	1.118(1.035,1.208)
	Old	1.101(1.012,1.197)	0.983(0.935,1.033)	0.909(0.864,0.958)	0.901(0.855,0.950)	0.956(0.910,1.005)	1.051(0.972,1.136)
**$PM_{2.5}$**	Male	1.033(0.961,1.110)	1.013(0.964,1.066)	1.000(0.947,1.057)	1.000(0.947,1.057)	1.012(0.963,1.064)	1.031(0.964,1.103)
	Female	1.073(0.998,1.154)	1.033(0.982,1.087)	1.004(0.951,1.061)	0.996(0.942,1.053)	1.006(0.957,1.058)	1.027(0.960,1.099)
	Young	1.008(0.936,1.085)	1.015(0.965,1.068)	1.023(0.968,1.080)	1.030(0.975,1.088)	1.037(0.987,1.091)	1.045(0.976,1.119)
	Old	1.067(0.994,1.144)	1.010(0.961,1.062)	0.972(0.920,1.026)	0.963(0.912,1.018)	0.984(0.937,1.034)	1.021(0.956,1.091)
**PM_{10}**	Male	1.071(1.011,1.135)	1.096(1.051,1.143)	1.113(1.062,1.167)	1.112(1.060,1.165)	1.092(1.047,1.139)	1.064(1.006,1.126)
	Female	1.089(1.026,1.155)	1.094(1.048,1.142)	1.094(1.042,1.148)	1.085(1.034,1.139)	1.067(1.022,1.114)	1.045(0.986,1.108)
	Young	1.041(0.979,1.107)	1.086(1.040,1.135)	1.119(1.067,1.175)	1.126(1.073,1.181)	1.105(1.058,1.153)	1.070(1.009,1.136)
	Old	1.085(1.026,1.147)	1.074(1.031,1.119)	1.063(1.015,1.114)	1.054(1.007,1.105)	1.047(1.004,1.091)	1.039(0.984,1.098)
**SO_2**	Male	1.014(0.908,1.133)	1.157(1.086,1.233)	1.273(1.191,1.361)	1.303(1.218,1.395)	1.242(1.166,1.323)	1.140(1.027,1.266)
	Female	1.031(0.921,1.154)	1.177(1.104,1.256)	1.289(1.205,1.379)	1.300(1.213,1.392)	1.206(1.131,1.286)	1.073(0.966,1.193)
	Young	1.055(0.946,1.176)	1.237(1.163,1.316)	1.382(1.295,1.474)	1.404(1.313,1.500)	1.297(1.219,1.380)	1.141(1.030,1.264)
	Old	0.962(0.859,1.079)	1.059(0.992,1.131)	1.137(1.061,1.218)	1.161(1.082,1.245)	1.128(1.057,1.204)	1.068(0.960,1.188)
**NO_2**	Male	1.031(0.971,1.095)	1.074(1.030,1.119)	1.105(1.056,1.155)	1.109(1.061,1.161)	1.088(1.044,1.134)	1.054(0.995,1.117)
	Female	1.042(0.980,1.107)	1.064(1.021,1.110)	1.081(1.033,1.131)	1.084(1.035,1.135)	1.073(1.030,1.119)	1.056(0.996,1.120)
	Young	1.024(0.962,1.089)	1.082(1.037,1.128)	1.123(1.074,1.176)	1.127(1.077,1.180)	1.093(1.048,1.140)	1.041(0.981,1.105)
	Old	1.041(0.982,1.104)	1.056(1.014,1.100)	1.069(1.022,1.117)	1.076(1.029,1.124)	1.077(1.034,1.121)	1.075(1.016,1.137)
**O_3**	Male	0.787(0.742,0.835)	0.788(0.758,0.818)	0.792(0.761,0.825)	0.805(0.772,0.839)	0.826(0.797,0.857)	0.853(0.810,0.897)
	Female	0.832(0.783,0.884)	0.810(0.779,0.842)	0.795(0.763,0.829)	0.794(0.761,0.829)	0.807(0.777,0.838)	0.827(0.785,0.872)
	Young	0.802(0.754,0.853)	0.795(0.765,0.827)	0.795(0.763,0.829)	0.807(0.774,0.842)	0.832(0.801,0.864)	0.865(0.820,0.912)
	Old	0.846(0.798,0.896)	0.840(0.810,0.872)	0.836(0.803,0.871)	0.835(0.801,0.870)	0.836(0.806,0.867)	0.839(0.798,0.882)

Supplementary Table 10 Subgroup analysis by gender and age of cumulative lagged relative risks (RRs) and 95% confidence intervals (CIs) for the impact of extremely low air pollution concentrations on CHD hospitalizations in Henan Province, 2016–2021(the maximum lag period = 6)

pollutant	group	Lag00	Lag01	Lag02	Lag03	Lag04	Lag05	Lag06
		RR 95% CI	RR 95% CI	RR 95% CI	RR 95% CI	RR 95% CI	RR 95% CI	RR 95% CI
**CO**	Male	0.995(0.977,1.013)	0.996(0.969,1.024)	1.001(0.970,1.034)	1.008(0.973,1.044)	1.012(0.975,1.050)	1.011(0.974,1.048)	1.002(0.967,1.039)
	Female	0.986(0.968,1.004)	0.985(0.957,1.013)	0.993(0.961,1.026)	1.006(0.970,1.042)	1.015(0.977,1.054)	1.014(0.977,1.053)	1.001(0.966,1.039)
	Young	1.000(0.982,1.018)	0.996(0.969,1.024)	0.990(0.959,1.022)	0.979(0.946,1.014)	0.965(0.930,1.001)	0.946(0.913,0.981)	0.924(0.892,0.958)
	Old	0.979(0.961,0.997)	0.975(0.948,1.003)	0.985(0.954,1.017)	1.002(0.968,1.038)	1.019(0.981,1.058)	1.028(0.991,1.067)	1.026(0.989,1.064)
**$PM_{2.5}$**	Male	0.995(0.983,1.007)	0.992(0.973,1.012)	0.991(0.966,1.015)	0.990(0.961,1.019)	0.988(0.955,1.022)	0.985(0.949,1.022)	0.980(0.942,1.018)
	Female	0.989(0.976,1.001)	0.982(0.963,1.002)	0.979(0.955,1.004)	0.978(0.950,1.008)	0.978(0.945,1.012)	0.976(0.941,1.013)	0.973(0.936,1.012)
	Young	1.000(0.988,1.013)	0.998(0.979,1.019)	0.995(0.970,1.020)	0.989(0.960,1.019)	0.982(0.950,1.016)	0.974(0.939,1.010)	0.965(0.929,1.003)
	Old	0.988(0.977,1.000)	0.984(0.965,1.003)	0.984(0.961,1.009)	0.989(0.960,1.019)	0.994(0.961,1.029)	0.998(0.961,1.036)	0.999(0.960,1.039)
**PM_{10}**	Male	0.984(0.968,1.000)	0.962(0.937,0.987)	0.937(0.906,0.969)	0.910(0.874,0.948)	0.886(0.848,0.929)	0.864(0.821,0.910)	0.848(0.803,0.895)
	Female	0.979(0.963,0.995)	0.957(0.932,0.983)	0.935(0.904,0.968)	0.914(0.877,0.953)	0.895(0.853,0.940)	0.880(0.834,0.927)	0.967(0.820,0.916)
	Young	0.993(0.976,1.010)	0.975(0.949,1.003)	0.950(0.918,0.984)	0.920(0.883,0.960)	0.891(0.849,0.935)	0.865(0.821,0.912)	0.844(0.799,0.891)
	Old	0.978(0.963,0.994)	0.959(0.935,0.984)	0.942(0.912,0.973)	0.927(0.891,0.965)	0.915(0.874,0.959)	0.906(0.861,0.954)	0.900(0.853,0.949)
**SO_2**	Male	1.002(0.987,1.018)	0.990(0.967,1.014)	0.966(0.941,0.993)	0.936(0.909,0.964)	0.906(0.879,0.934)	0.880(0.854,0.907)	0.860(0.835,0.886)
	Female	0.998(0.983,1.014)	0.984(0.961,1.008)	0.959(0.933,0.986)	0.930(0.903,0.958)	0.902(0.875,0.931)	0.881(0.854,0.908)	0.867(0.841,0.893)
	Young	0.997(0.982,1.013)	0.979(0.957,1.002)	0.949(0.924,0.974)	0.912(0.886,0.938)	0.876(0.850,0.902)	0.845(0.821,0.870)	0.821(0.797,0.845)
	Old	1.007(0.991,1.024)	1.003(0.979,1.028)	0.991(0.963,1.019)	0.972(0.943,1.003)	0.955(0.924,0.986)	0.940(0.911,0.970)	0.931(0.902,0.961)
**NO_2**	Male	0.992(0.966,1.018)	0.967(0.929,1.008)	0.931(0.885,0.980)	0.891(0.839,0.946)	0.854(0.797,0.914)	0.826(0.767,0.889)	0.808(0.747,0.873)
	Female	0.988(0.963,1.015)	0.967(0.927,1.008)	0.937(0.891,0.987)	0.904(0.851,0.961)	0.872(0.814,0.935)	0.845(0.784,0.910)	0.823(0.760,0.890)
	Young	0.998(0.972,1.024)	0.976(0.936,1.017)	0.938(0.891,0.987)	0.894(0.841,0.949)	0.851(0.794,0.911)	0.815(0.757,0.878)	0.789(0.730,0.853)
	Old	0.986(0.961,1.011)	0.962(0.925,1.001)	0.932(0.887,0.979)	0.900(0.848,0.954)	0.871(0.814,0.931)	0.848(0.788,0.912)	0.833(0.771,0.900)
**O_3**	Male	1.149(1.106,1.194)	1.332(1.255,1.414)	1.551(1.441,1.668)	1.795(1.650,1.953)	2.043(1.858,2.246)	2.262(2.044,2.504)	2.427(2.181,2.701)
	Female	1.111(1.068,1.155)	1.270(1.194,1.350)	1.480(1.373,1.595)	1.733(1.589,1.889)	1.994(1.809,2.198)	2.220(2.000,2.645)	2.371(2.126,2.645)
	Young	1.137(1.093,1.183)	1.308(1.230,1.392)	1.514(1.405,1.632)	1.745(1.601,1.902)	1.978(1.796,2.178)	2.181(1.968,2.417)	2.328(2.090,2.593)
	Old	1.099(1.059,1.141)	1.232(1.161,1.306)	1.398(1.300,1.502)	1.589(1.460,1.728)	1.781(1.620,1.959)	1.949(1.759,2.159)	2.068(1.856,2.304)

Supplementary Table 11 Subgroup analysis by gender and age of single-day lagged relative risks (RRs) and 95% confidence intervals (CIs) for the impact of extremely low air pollution concentrations on CHD hospitalizations in Henan Province, 2016–2021(the maximum lag period = 5)

pollutant	group	Lag0 RR (95% CI)	Lag1 RR (95% CI)	Lag2 RR (95% CI)	Lag3 RR (95% CI)	Lag4 RR (95% CI)	Lag5 RR (95% CI)	Lag6 RR (95% CI)
**CO**	Male	0.995(0.977,1.013)	1.001(0.990,1.012)	1.005(0.995,1.015)	1.007(0.995,1.018)	1.004(0.994,1.014)	0.999(0.988,1.009)	0.992(0.975,1.009)
	Female	0.986(0.968,1.004)	0.999(0.988,1.009)	1.009(0.999,1.019)	1.013(1.001,1.024)	1.009(0.999,1.020)	1.000(0.989,1.010)	0.987(0.971,1.004)
	Young	1.000(0.982,1.018)	0.997(0.986,1.007)	0.993(0.984,1.003)	0.989(0.978,1.000)	0.985(0.975,0.995)	0.981(0.971,0.991)	0.977(0.961,0.993)
	Old	0.979(0.961,0.997)	0.996(0.985,1.007)	1.010(1.000,1.020)	1.018(1.006,1.027)	1.017(1.006,1.027)	1.009(0.998,1.019)	0.998(0.981,1.015)
**$PM_{2.5}$**	Male	0.995(0.983,1.007)	0.997(0.989,1.005)	0.999(0.990,1.007)	0.999(0.990,1.008)	0.998(0.990,1.007)	0.997(0.989,1.005)	0.995(0.983,1.006)
	Female	0.989(0.976,1.001)	0.993(0.985,1.002)	0.997(0.989,1.005)	0.999(0.990,1.009)	1.000(0.991,1.008)	0.999(0.990,1.007)	0.997(0.985,1.008)
	Young	1.000(0.988,1.013)	0.998(0.990,1.006)	0.996(0.988,1.004)	0.994(0.985,1.004)	0.993(0.984,1.001)	0.992(0.984,1.000)	0.991(0.979,1.002)
	Old	0.988(0.977,1.000)	0.995(0.987,1.003)	1.001(0.993,1.009)	1.004(0.995,1.014)	1.005(0.997,1.014)	1.004(0.996,1.012)	1.001(0.990,1.013)
**PM_{10}**	Male	0.984(0.968,1.000)	0.978(0.967,0.989)	0.974(0.962,0.985)	0.972(0.959,0.985)	0.973(0.961,0.984)	0.976(0.965,0.987)	0.981(0.965,0.996)
	Female	0.979(0.963,0.996)	0.978(0.967,0.989)	0.977(0.965,0.989)	0.977(0.964,0.991)	0.979(0.968,0.991)	0.982(0.971,0.994)	0.986(0.970,1.002)
	Young	0.993(0.976,1.010)	0.982(0.971,0.994)	0.974(0.962,0.986)	0.969(0.956,0.982)	0.968(0.957,0.980)	0.971(0.960,0.982)	0.975(0.959,0.992)
	Old	0.978(0.963,0.994)	0.980(0.970,0.991)	0.982(0.971,0.994)	0.984(0.972,0.997)	0.987(0.976,0.999)	0.990(0.979,1.001)	0.993(0.977,1.009)
**SO_2**	Male	1.002(0.987,1.018)	0.988(0.979,0.997)	0.976(0.968,0.984)	0.969(0.960,0.978)	0.968(0.960,0.976)	0.971(0.963,0.980)	0.977(0.963,0.991)
	Female	0.998(0.983,1.014)	0.985(0.977,0.994)	0.975(0.967,0.983)	0.969(0.960,0.979)	0.970(0.962,0.979)	0.976(0.967,0.985)	0.984(0.970,0.999)
	Young	0.997(0.982,1.013)	0.982(0.973,0.990)	0.969(0.961,0.977)	0.961(0.952,0.977)	0.960(0.952,0.968)	0.965(0.956,0.973)	0.971(0.958,0.985)
	Old	1.007(0.991,1.024)	0.996(0.987,1.006)	0.987(0.979,0.995)	0.982(0.972,0.991)	0.982(0.973,0.990)	0.985(0.976,0.994)	0.991(0.976,1.005)
**NO_2**	Male	0.992(0.966,1.018)	0.975(0.959,0.992)	0.963(0.947,0.979)	0.956(0.938,0.975)	0.959(0.942,0.975)	0.967(0.951,0.983)	0.979(0.955,1.003)
	Female	0.988(0.963,1.015)	0.978(0.961,0.995)	0.970(0.953,0.986)	0.965(0.946,0.984)	0.965(0.948,0.982)	0.968(0.952,0.985)	0.974(0.950,0.998)
	Young	0.998(0.972,1.024)	0.978(0.961,0.995)	0.962(0.945,0.978)	0.952(0.934,0.971)	0.952(0.935,0.969)	0.958(0.942,0.975)	0.968(0.944,0.993)
	Old	0.986(0.961,1.011)	0.976(0.960,0.992)	0.969(0.953,0.985)	0.965(0.947,0.984)	0.968(0.951,0.984)	0.974(0.958,0.990)	0.983(0.959,1.006)
**O_3**	Male	1.149(1.106,1.194)	1.159(1.132,1.188)	1.164(1.137,1.191)	1.158(1.128,1.189)	1.138(1.111,1.166)	1.107(1.082,1.133)	1.073(1.038,1.109)
	Female	1.111(1.068,1.155)	1.143(1.115,1.171)	1.166(1.139,1.194)	1.171(1.140,1.203)	1.151(1.122,1.180)	1.113(1.087,1.140)	1.068(1.033,1.105)
	Young	1.137(1.093,1.183)	1.150(1.122,1.179)	1.157(1.130,1.185)	1.153(1.122,1.184)	1.133(1.105,1.162)	1.103(1.077,1.129)	1.068(1.032,1.105)
	Old	1.099(1.059,1.141)	1.120(1.094,1.147)	1.135(1.109,1.161)	1.137(1.107,1.167)	1.121(1.095,1.149)	1.094(1.069,1.119)	1.061(1.027,1.096)

Supplementary Table 12 Subgroup analysis by gender and age of cumulative lagged relative risks (RRs) and 95% confidence intervals (CIs) for the impact of extremely high air pollution concentrations on CHD hospitalizations in Henan Province, 2016–2021(the maximum lag period = 6)

pollutant	group	Lag00	Lag01	Lag02	Lag03	Lag04	Lag05	Lag06
		RR 95% CI	RR 95% CI	RR 95% CI	RR 95% CI	RR 95% CI	RR 95% CI	RR 95% CI
**CO**	Male	1.023(0.940,1.113)	1.018(0.895,1.159)	0.994(0.856,1.155)	0.964(0.819,1.135)	0.946(0.796,1.12)	0.952(0.803,1.129)	0.990(0.837,1.171)
	Female	1.069(0.980,1.165)	1.076(0.943,1.227)	1.034(0.888,1.203)	0.974(0.825,1.150)	0.934(0.784,1.113)	0.936(0.787,1.113)	0.993(0.837,1.178)
	Young	1.000(0.919,1.089)	1.017(0.894,1.156)	1.050(0.905,1.219)	1.104(0.939,1.298)	1.183(0.997,1.404)	1.294(1.094,1.531)	1.445(1.225,1.706)
	Old	1.104(1.014,1.202)	1.125(0.988,1.281)	1.073(0.923,1.248)	0.989(0.839,1.166)	0.916(0.769,1.091)	0.879(0.740,1.046)	0.888(0.748,1.055)
**$PM_{2.5}$**	Male	1.029(0.958,1.106)	1.048(0.934,1.176)	1.057(0.915,1.221)	1.063(0.894,1.263)	1.073(0.880,1.308)	1.093(0.882,1.355)	1.128(0.899,1.416)
	Female	1.069(0.993,1.150)	1.112(0.990,1.250)	1.132(0.978,1.311)	1.137(0.955,1.354)	1.140(0.933,1.392)	1.150(0.925,1.428)	1.172(0.932,1.474)
	Young	0.998(0.927,1.075)	1.009(0.897,1.135)	1.032(0.892,1.194)	1.067(0.898,1.268)	1.112(0.913,1.354)	1.167(0.944,1.443)	1.232(0.985,1.541)
	Old	1.071(0.998,1.149)	1.102(0.983,1.235)	1.096(0.949,1.266)	1.067(0.897,1.270)	1.035(0.847,1.265)	1.013(0.814,1.260)	1.005(0.798,1.267)
**PM_{10}**	Male	1.061(1.001,1.125)	1.149(1.046,1.262)	1.264(1.121,1.425)	1.400(1.209,1.622)	1.546(1.304,1.833)	1.686(1.402,2.028)	1.809(1.490,2.197)
	Female	1.079(1.017,1.145)	1.170(1.063,1.287)	1.271(1.125,1.437)	1.379(1.188,1.602)	1.486(1.249,1.769)	1.585(1.312,1.914)	1.668(1.367,2.034)
	Young	1.027(0.965,1.092)	1.094(0.991,1.207)	1.202(1.061,1.362)	1.346(1.158,1.565)	1.511(1.270,1.798)	1.680(1.393,2.025)	1.838(1.510,2.236)
	Old	1.081(1.022,1.144)	1.161(1.061,1.272)	1.239(1.102,1.392)	1.310(1.135,1.512)	1.373(1.161,1.623)	1.423(1.186,1.708)	1.460(1.205,1.770)
**SO_2**	Male	0.983(0.879,1.099)	1.073(0.905,1.272)	1.278(1.051,1.554)	1.606(1.300,1.984)	2.034(1.630,2.538)	2.510(2.021,3.116)	2.966(2.393,3.677)
	Female	1.011(0.902,1.133)	1.124(0.945,1.336)	1.348(1.105,1.644)	1.685(1.360,2.087)	2.093(1.673,2.619)	2.495(2.004,3.106)	2.803(2.255,3.484)
	Young	1.019(0.914,1.136)	1.163(0.986,1.372)	1.460(1.208,1.765)	1.941(1.582,2.382)	2.596(2.095,3.218)	3.366(2.729,4.151)	4.149(3.370,5.108)
	Old	0.949(0.845,1.065)	0.975(0.818,1.163)	1.071(0.874,1.312)	1.223(0.981,1.524)	1.398(1.109,1.762)	1.558(1.241,1.956)	1.668(1.329,2.094)
**NO_2**	Male	1.020(0.960,1.083)	1.081(0.983,1.188)	1.181(1.049,1.328)	1.310(1.139,1.506)	1.446(1.233,1.696)	1.564(1.317,1.857)	1.645(1.372,1.973)
	Female	1.027(0.966,1.092)	1.082(0.982,1.192)	1.163(1.032,1.311)	1.265(1.098,1.457)	1.375(1.170,1.616)	1.483(1.246,1.764)	1.578(1.313,1.895)
	Young	1.005(0.945,1.069)	1.059(0.961,1.167)	1.161(1.030,1.308)	1.300(1.129,1.497)	1.458(1.242,1.712)	1.610(1.355,1.913)	1.736(1.447,2.082)
	Old	1.034(0.975,1.096)	1.094(0.997,1.201)	1.179(1.050,1.323)	1.280(1.115,1.469)	1.382(1.180,1.618)	1.470(1.239,1.743)	1.531(1.278,1.833)
**O_3**	Male	0.807(0.760,0.856)	0.642(0.585,0.704)	0.507(0.453,0.568)	0.405(0.355,0.461)	0.331(0.286,0.384)	0.283(0.242,0.331)	0.254(0.215,0.299)
	Female	0.850(0.800,0.903)	0.691(0.629,0.760)	0.545(0.486,0.612)	0.427(0.374,0.488)	0.344(0.296,0.400)	0.291(0.248,0.342)	0.263(0.222,0.312)
	Young	0.819(0.771,0.871)	0.660(0.600,0.726)	0.527(0.469,0.591)	0.423(0.370,0.483)	0.348(0.300,0.404)	0.299(0.255,0.351)	0.271(0.229,0.320)
	Old	0.864(0.815,0.915)	0.725(0.661,0.794)	0.596(0.533,0.666)	0.489(0.429,0.557)	0.409(0.353,0.474)	0.356(0.304,0.417)	0.325(0.275-0.384)

Supplementary Table 13 Subgroup analysis by gender and age of single-day lagged relative risks (RRs) and 95% confidence intervals (CIs) for the impact of extremely high air pollution concentrations on CHD hospitalizations in Henan Province, 2016–2021(the maximum lag period = 6)

pollutant	group	Lag0	Lag1	Lag2	Lag3	Lag4	Lag5	Lag6
		RR 95% CI	RR 95% CI	RR 95% CI	RR 95% CI	RR 95% CI	RR 95% CI	RR 95% CI
**CO**	Male	1.023(0.940,1.113)	0.996(0.948,1.046)	0.976(0.933,1.021)	0.970(0.920,1.022)	0.981(0.936,1.029)	1.006(0.959,1.057)	1.039(0.961,1.124)
	Female	1.069(0.980,1.165)	1.006(0.957,1.058)	0.961(0.918,1.006)	0.943(0.893,0.995)	0.959(0.913,1.006)	1.002(0.954,1.052)	1.061(0.981,1.149)
	Young	1.000(0.919,1.089)	1.016(0.968,1.067)	1.033(1.988,1.080)	1.051(0.998,1.108)	1.072(1.023,1.123)	1.094(1.043,1.147)	1.117(1.034,1.206)
	Old	1.104(1.014,1.202)	1.019(0.969,1.071)	0.954(0.911,0.999)	0.921(0.876,0.972)	0.926(0.883,0.972)	0.960(0.914,1.008)	1.010(0.934,1.093)
**$PM_{2.5}$**	Male	1.029(0.958,1.106)	1.018(0.970,1.068)	1.009(0.961,1.059)	1.005(0.952,1.062)	1.010(0.960,1.061)	1.019(0.971,1.070)	1.032(0.964,1.104)
	Female	1.069(0.993,1.150)	1.041(0.991,1.093)	1.018(0.969,1.069)	1.004(0.950,1.061)	1.002(0.953,1.054)	1.009(0.961,1.059)	1.019(0.952,1.092)
	Young	0.998(0.927,1.075)	1.011(0.963,1.061)	1.023(0.975,1.074)	1.034(0.979,1.092)	1.042(0.991,1.096)	1.049(1.000,1.101)	1.056(0.986,1.131)
	Old	1.071(0.998,1.149)	1.029(0.980,1.080)	0.995(0.947,1.045)	0.974(0.922,1.029)	0.970(0.922,1.020)	0.978(0.932,1.026)	0.993(0.928,1.061)
**PM_{10}**	Male	1.061(1.001,1.125)	1.083(1.040,1.127)	1.100(1.055,1.147)	1.108(1.057,1.161)	1.104(1.058,1.151)	1.091(1.048,1.135)	1.073(1.013,1.137)
	Female	1.079(1.017,1.145)	1.084(1.040,1.129)	1.087(1.041,1.135)	1.085(1.034,1.138)	1.078(1.032,1.125)	1.066(1.023,1.111)	1.052(0.992,1.117)
	Young	1.027(0.965,1.092)	1.065(1.022,1.111)	1.099(1.053,1.147)	1.120(1.067,1.175)	1.123(1.075,1.172)	1.112(1.067,1.158)	1.094(1.030,1.162)
	Old	1.081(1.022,1.144)	1.074(1.033,1.117)	1.066(1.023,1.111)	1.058(1.010,1.108)	1.048(1.005,1.092)	1.037(0.997,1.079)	1.026(0.970,1.086)
**SO_2**	Male	0.983(0.879,1.099)	1.091(1.023,1.164)	1.191(1.124,1.263)	1.257(1.174,1.345)	1.267(1.192,1.346)	1.234(1.158,1.315)	1.182(1.065,1.312)
	Female	1.011(0.902,1.133)	1.111(0.041,1.186)	1.200(1.131,1.273)	1.250(1.167,1.339)	1.242(1.168,1.322)	1.192(1.117,1.272)	1.123(1.011,1.249)
	Young	1.019(0.914,1.136)	1.142(1.072,1.215)	1.255(1.186,1.329)	1.329(1.243,1.421)	1.338(1.260,1.420)	1.296(1.218,1.379)	1.233(1.113,1.365)
	Old	0.949(0.845,1.065)	1.028(0.961,1.099)	1.098(1.034,1.167)	1.141(1.064,1.224)	1.144(1.074,1.218)	1.114(1.043,1.190)	1.071(0.962,1.192)
**NO_2**	Male	1.020(0.960,1.083)	1.060(1.019,1.102)	1.093(1.050,1.137)	1.109(1.061,1.160)	1.104(1.060,1.150)	1.082(1.040,1.125)	1.052(0.993,1.114)
	Female	1.027(0.966,1.092)	1.053(1.012,1.096)	1.075(1.033,1.119)	1.087(1.039,1.138)	1.087(1.043,1.133)	1.078(1.036,1.122)	1.064(1.004,1.128)
	Young	1.005(0.945,1.069)	1.054(1.013,1.097)	1.095(1.053,1.140)	1.120(1.071,1.173)	1.121(1.076,1.169)	1.104(1.061,1.149)	1.078(1.017,1.143)
	Old	1.034(0.975,1.096)	1.058(1.018,1.100)	1.077(1.036,1.120)	1.086(1.039,1.135)	1.080(1.037,1.124)	1.063(1.023,1.105)	1.042(0.985,1.102)
**O_3**	Male	0.807(0.760,0.856)	0.796(0.767,0.826)	0.791(0.763,0.819)	0.797(0.765,0.830)	0.819(0.789,0.850)	0.854(0.824,0.885)	0.897(0.852,0.943)
	Female	0.850(0.800-0.903)	0.814(0.783,0.845)	0.789(0.760,0.818)	0.784(0.752,0.817)	0.805(0.774,0.836)	0.847(0.817,0.879)	0.903(0.857,0.952)
	Young	0.819(0.771,0.871)	0.805(0.775,0.837)	0.798(0.769,0.824)	0.803(0.770,0.837)	0.824(0.793,0.856)	0.860(0.829,0.892)	0.904(0.857,0.953)
	Old	0.864(0.815,0.915)	0.839(0.809,0.871)	0.822(0.794,0.852)	0.820(0.788,0.854)	0.838(0.807,0.869)	0.870(0.840,0.902)	0.912(0.868,0.959)

Supplementary Table 14 Subgroup analysis by gender and age of cumulative lagged relative risks (RRs) and 95% confidence intervals (CIs) for the impact of extremely low air pollution concentrations on CHD hospitalizations in Henan Province, 2016–2021(the maximum lag period = 8)

pollutant	group	Lag00	Lag01	Lag02	Lag03	Lag04
		RR 95% CI	RR 95% CI	RR 95% CI	RR 95% CI	RR 95% CI
**CO**	Male	0.996(0.978,1.015)	0.995(0.966,1.024)	0.995(0.961,1.029)	0.996(0.960,1.033)	0.998(0.960,1.037)
	Female	0.992(0.973,1.010)	0.987(0.958,1.016)	0.985(0.951,1.020)	0.986(0.950,1.023)	0.988(0.951,1.028)
	Young	1.003(0.985,1.021)	1.000(0.971,1.029)	0.990(0.957,1.024)	0.977(0.942,1.013)	0.962(0.926,0.999)
	Old	0.983(0.965,1.001)	0.975(0.947,1.004)	0.975(0.943,1.009)	0.982(0.947,1.019)	0.993(0.956,1.033)
**$PM_{2.5}$**	Male	0.996(0.983,1.008)	0.992(0.972,1.012)	0.989(0.965,1.014)	0.987(0.959,1.015)	0.985(0.954,1.017)
	Female	0.990(0.978,1.003)	0.983(0.963,1.003)	0.978(0.954,1.003)	0.975(0.948,1.003)	0.973(0.942,1.005)
	Young	1.001(0.988,1.013)	0.999(0.979,1.020)	0.995(0.970,1.020)	0.988(0.961,1.017)	0.981(0.950,1.014)
	Old	0.990(0.978,1.002)	0.984(0.965,1.004)	0.982(0.959,1.007)	0.984(0.957,1.012)	0.988(0.956,1.020)
**PM_{10}**	Male	0.984(0.969,1.000)	0.965(0.940,0.991)	0.943(0.912,0.975)	0.919(0.885,0.955)	0.895(0.856,0.936)
	Female	0.982(0.966,0.998)	0.962(0.936,0.988)	0.941(0.910,0.973)	0.920(0.885,0.957)	0.900(0.860,0.941)
	Young	0.994(0.977,1.011)	0.979(0.952,1.006)	0.956(0.924,0.990)	0.929(0.893,0.966)	0.900(0.860,0.942)
	Old	0.981(0.965,0.996)	0.963(0.939,0.988)	0.947(0.917,0.978)	0.933(0.898,0.969)	0.920(0.881,0.961)
**SO_2**	Male	1.004(0.989,1.020)	0.994(0.971,1.019)	0.973(0.946,1.001)	0.943(0.916,0.972)	0.912(0.884,0.941)
	Female	1.000(0.985,1.016)	0.990(0.966,1.015)	0.972(0.944,1.000)	0.947(0.919,0.976)	0.920(0.891,0.950)
	Young	0.999(0.984,1.014)	0.984(0.961,1.007)	0.957(0.931,0.984)	0.923(0.896,0.950)	0.886(0.860,0.914)
	Old	1.010(0.994,1.026)	1.009(0.984,1.034)	0.999(0.970,1.029)	0.983(0.953,1.014)	0.964(0.933,0.966)
**NO_2**	Male	0.998(0.972,1.024)	0.981(0.940,1.023)	0.951(0.903,1.001)	0.913(0.861,0.968)	0.873(0.818,0.933)
	Female	0.997(0.971,1.024)	0.982(0.941,1.025)	0.956(0.908,1.008)	0.924(0.870,0.980)	0.888(0.831,0.949)
	Young	1.004(0.978,1.031)	0.989(0.947,1.032)	0.956(0.907,1.008)	0.912(0.860,0.968)	0.866(0.810,0.925)
	Old	0.994(0.970,1.020)	0.979(0.940,1.020)	0.956(0.909,1.006)	0.927(0.875,0.982)	0.895(0.839,0.955)
**O_3**	Male	1.131(1.090,1.174)	1.279(1.204,1.359)	1.447(1.345,1.557)	1.635(1.507,1.775)	1.845(1.685,2.020)
	Female	1.099(1.057,1.141)	1.222(1.148,1.300)	1.374(1.274,1.481)	1.557(1.432,1.693)	1.770(1.613,1.942)
	Young	1.122(1.080,1.166)	1.257(1.181,1.338)	1.408(1.305,1.518)	1.574(1.447,1.712)	1.759(1.604,1.929)
	Old	1.087(1.048,1.127)	1.193(1.125,1.266)	1.323(1.231,1.422)	1.476(1.361,1.600)	1.648(1.506,1.804)

pollutant	group	Lag05 RR 95% CI	Lag06 RR 95% CI	Lag07 RR 95% CI	Lag08 RR 95% CI
**CO**	Male	1.000(0.961,1.042)	1.003(0.963,1.044)	1.004(0.966,1.043)	1.005(0.968,1.042)
	Female	0.992(0.952,1.033)	0.995(0.955,1.037)	0.999(0.961,1.038)	1.001(0.964,1.039)
	Young	0.947(0.910,0.985)	0.935(0.898,0.973)	0.926(0.892,0.962)	0.922(0.889,0.956)
	Old	1.006(0.965,1.047)	1.016(0.975,1.059)	1.024(0.985,1.064)	1.027(0.989,1.067)
**$PM_{2.5}$**	Male	0.983(0.948,1.019)	0.981(0.944,1.020)	0.979(0.941,1.020)	0.977(0.938,1.018)
	Female	0.972(0.937,1.008)	0.971(0.934,1.010)	0.970(0.931,1.010)	0.968(0.928,1.009)
	Young	0.975(0.940,1.010)	0.969(0.932,1.007)	0.964(0.927,1.003)	0.961(0.923,1.001)
	Old	0.992(0.956,1.029)	0.995(0.957,1.036)	0.997(0.957,1.039)	0.997(0.956,1.040)
**PM_{10}**	Male	0.873(0.830,0.918)	0.854(0.809,0.901)	0.838(0.792,0.887)	0.827(0.780,0.876)
	Female	0.881(0.837,0.927)	0.865(0.818,0.914)	0.852(0.804,0.903)	0.843(0.794,0.895)
	Young	0.873(0.830,0.919)	0.851(0.805,0.899)	0.834(0.788,0.883)	0.825(0.777,0.875)
	Old	0.908(0.864,0.954)	0.898(0.851,0.947)	0.888(0.840,0.939)	0.880(0.830,0.932)
**SO_2**	Male	0.884(0.855,0.913)	0.864(0.836,0.893)	0.854(0.828,0.881)	0.856(0.830,0.883)
	Female	0.895(0.866,0.926)	0.876(0.847,0.906)	0.864(0.837,0.892)	0.859(0.833,0.887)
	Young	0.854(0.827,0.882)	0.830(0.804,0.857)	0.817(0.792,0.842)	0.815(0.791,0.840)
	Old	0.946(0.914,0.979)	0.933(0.901,0.965)	0.926(0.896,0.957)	0.927(0.897,0.958)
**NO_2**	Male	0.837(0.778,0.901)	0.808(0.747,0.874)	0.789(0.727,0.855)	0.779(0.718,0.847)
	Female	0.854(0.793,0.920)	0.825(0.762,0.894)	0.803(0.740,0.871)	0.788(0.725,0.857)
	Young	0.823(0.765,0.886)	0.790(0.731,0.855)	0.770(0.710,0.835)	0.762(0.702,0.828)
	Old	0.864(0.804,0.929)	0.836(0.774,0.904)	0.813(0.750,0.881)	0.794(0.731,0.862)
**O_3**	Male	2.076(1.879,2.294)	2.326(2.090,2.588)	2.594(2.323,2.897)	2.879(2.567,3.229)
	Female	2.008(1.813,2.224)	2.261(2.027,2.523)	2.521(2.252,2.823)	2.779(2.471,3.125)
	Young	1.965(1.086,1.175)	2.194(1.969,2.444)	2.450(2.192,2.738)	2.736(2.438,3.071)
	Old	1.834(1.659,2.027)	2.023(1.817,2.253)	2.206(1.973,2.468)	2.374(2.113,2.668)

Supplementary Table 15 Subgroup analysis by gender and age of single-day lagged relative risks (RRs) and 95% confidence intervals (CIs) for the impact of extremely low air pollution concentrations on CHD hospitalizations in Henan Province, 2016–2021(the maximum lag period = 8)

pollutant	group	Lag0	Lag1	Lag2	Lag3	Lag4
		RR 95% CI	RR 95% CI	RR 95% CI	RR 95% CI	RR 95% CI
**CO**	Male	0.996(0.978,1.015)	0.998(0.987,1.010)	1.000(0.992,1.008)	1.001(0.992,1.011)	1.002(0.991,1.013)
	Female	0.992(0.973,1.010)	0.995(0.9884,1.006)	0.998(0.990,1.007)	1.001(0.991,1.011)	1.003(0.992,1.014)
	Young	1.003(0.985,1.021)	0.997(0.986,1.008)	0.991(0.983,0.999)	0.987(0.977,0.996)	0.984(0.974,0.995)
	Old	0.983(0.963,1.001)	0.992(0.981,1.003)	1.000(0.992,1.009)	1.007(0.997,1.017)	1.011(1.000,1.022)
**$PM_{2.5}$**	Male	0.996(0.983,1.008)	0.996(0.988,1.005)	0.997(0.990,1.004)	0.998(0.990,1.006)	0.998(0.989,1.007)
	Female	0.990(0.978,1.003)	0.993(0.985,1.001)	0.995(0.988,1.002)	0.997(0.989,1.005)	0.998(0.989,1.007)
	Young	1.001(0.988,1.013)	0.998(0.990,1.006)	0.996(0.989,1.003)	0.994(0.986,1.002)	0.993(0.984,1.002)
	Old	0.990(0.978,1.002)	0.994(0.986,1.002)	0.998(0.992,1.005)	1.002(0.994,1.010)	1.004(0.995,1.013)
**PM_{10}**	Male	0.984(0.969,1.000)	0.980(0.970,0.991)	0.977(0.968,0.986)	0.975(0.964,0.985)	0.974(0.962,0.986)
	Female	0.982(0.966,0.998)	0.980(0.969,0.991)	0.978(0.969,0.988)	0.978(0.967,0.989)	0.978(0.966,0.990)
	Young	0.994(0.977,1.011)	0.985(0.974,0.996)	0.977(0.968,0.987)	0.971(0.961,0.982)	0.969(0.957,0.981)
	Old	0.981(0.965,0.996)	0.982(0.972,0.993)	0.984(0.974,0.993)	0.985(0.974,0.996)	0.986(0.975,0.998)
**SO_2**	Male	1.004(0.989,1.020)	0.990(0.981,1.000)	0.978(0.972,0.985)	0.970(0.962,0.977)	0.966(0.958,0.975)
	Female	1.000(0.985,1.016)	0.990(0.981,1.000)	0.981(0.974,0.988)	0.975(0.967,0.982)	0.972(0.963,0.981)
	Young	0.999(0.984,1.014)	0.985(0.976,0.994)	0.973(0.966,0.979)	0.964(0.957,0.972)	0.961(0.952,0.969)
	Old	1.010(0.994,1.026)	0.999(0.990,1.009)	0.990(0.983,0.997)	0.984(0.976,0.992)	0.981(0.971,0.990)
**NO_2**	Male	0.998(0.972,1.024)	0.983(0.966,1.000)	0.970(0.956,0.984)	0.960(0.945,0.976)	0.956(0.939,0.974)
	Female	0.997(0.971,1.024)	0.985(0.968,1.002)	0.974(0.960,0.988)	0.966(0.950,0.982)	0.961(0.944,0.979)
	Young	1.004(0.978,1.031)	0.984(0.967,1.002)	0.967(0.953,0.981)	0.954(0.939,0.970)	0.949(0.932,0.966)
	Old	0.994(0.970,1.020)	0.985(0.969,1.001)	0.976(0.963,0.990)	0.970(0.954,0.985)	0.966(0.949,0.983)
**O_3**	Male	1.131(1.090,1.174)	1.131(1.104,1.159)	1.131(1.110,1.153)	1.130(1.106,1.155)	1.128(1.101,1.156)
	Female	1.099(1.057,1.141)	1.112(1.085,1.140)	1.124(1.103,1.147)	1.133(1.108,1.159)	1.137(1.109,1.166)
	Young	1.122(1.080,1.166)	1.121(1.093,1.149)	1.119(1.098,1.141)	1.118(1.094,1.143)	1.117(1.090,1.146)
	Old	1.087(1.048,1.127)	1.098(1.072,1.125)	1.108(1.088,1.130)	1.115(1.091,1.140)	1.117(1.090,1.144)

pollutant	group	Lag5	Lag6	Lag7	Lag8
		RR 95% CI	RR 95% CI	RR 95% CI	RR 95% CI
**CO**	Male	1.002(0.993,1.012)	1.002(0.994,1.011)	1.001(0.991,1.012)	1.001(0.984,1.017)
	Female	1.004(0.994,1.014)	1.004(0.995,1.012)	1.003(0.992,1.014)	1.002(0.986,1.020)
	Young	0.985(0.975,0.994)	0.987(0.979,0.995)	0.991(0.980,1.001)	0.995(0.979,1.012)
	Old	1.012(1.002,1.022)	1.011(1.002,1.022)	1.007(0.997,1.018)	1.003(0.987,1.020)
**$PM_{2.5}$**	Male	0.998(0.990,1.006)	0.998(0.991,1.005)	0.998(0.990,1.006)	0.998(0.987,1.009)
	Female	0.999(0.991,1.007)	0.999(0.992,1.006)	0.999(0.991,1.006)	0.998(0.987,1.010)
	Young	0.993(0.985,1.001)	0.994(0.987,1.001)	0.995(0.988,1.003)	0.997(0.986,1.009)
	Old	1.004(0.996,1.012)	1.003(0.996,1.010)	1.002(0.994,1.009)	1.000(0.989,1.011)
**PM_{10}**	Male	0.975(0.965,0.986)	0.978(0.969,0.987)	0.982(0.971,0.992)	0.986(0.971,1.002)
	Female	0.979(0.968,0.990)	0.982(0.973,0.991)	0.985(0.975,0.996)	0.989(0.973,1.005)
	Young	0.970(0.959,0.981)	0.974(0.965,0.984)	0.981(0.970,0.992)	0.988(0.972,1.005)
	Old	0.987(0.977,0.998)	0.988(0.979,0.998)	0.989(0.979,1.000)	0.990(0.975,1.006)
**SO_2**	Male	0.969(0.961,0.977)	0.977(0.971,0.984)	0.989(0.980,0.998)	1.002(0.988,1.017)
	Female	0.973(0.965,0.981)	0.978(0.971,0.985)	0.986(0.977,0.995)	0.995(0.981,1.009)
	Young	0.964(0.956,0.972)	0.972(0.965,0.979)	0.984(0.975,0.992)	0.998(0.984,1.012)
	Old	0.982(0.973,0.990)	0.986(0.979,0.993)	0.993(0.984,1.002)	1.001(0.987,1.016)
**NO_2**	Male	0.958(0.942,0.974)	0.965(0.952,0.979)	0.976(0.960,0.992)	0.988(0.965,1.013)
	Female	0.962(0.946,0.978)	0.966(0.952,0.980)	0.973(0.957,0.990)	0.981(0.958,1.006)
	Young	0.951(0.935,0.967)	0.960(0.946,0.974)	0.974(0.958,0.990)	0.990(0.966,1.015)
	Old	0.965(0.950,0.981)	0.968(0.954,0.982)	0.972(0.956,0.988)	0.977(0.954,1.000)
**O_3**	Male	1.125(1.100,1.151)	1.121(1.099,1.143)	1.115(1.091,1.140)	1.110(1.075,1.146)
	Female	1.134(1.108,1.161)	1.126(1.104,1.149)	1.115(1.090,1.141)	1.102(1.066,1.140)
	Young	1.117(1.091,1.143)	1.117(1.095,1.139)	1.117(1.092,1.142)	1.117(1.080,1.155)
	Old	1.113(1.088,1.138)	1.103(1.082,1.125)	1.090(1.067,1.115)	1.076(1.043,1.111)

Supplementary Table 16 Subgroup analysis by gender and age of cumulative lagged relative risks (RRs) and 95% confidence intervals (CIs) for the impact of extremely high air pollution concentrations on CHD hospitalizations in Henan Province, 2016–2021(the maximum lag period = 8)

pollutant	group	Lag00	Lag01	Lag02	Lag03	Lag04
		RR 95% CI	RR 95% CI	RR 95% CI	RR 95% CI	RR 95% CI
**CO**	Male	1.017(0.935,1.107)	1.026(0.896,1.174)	1.026(0.875,1.203)	1.020(0.860,1.208)	1.009(0.844,1.207)
	Female	1.040(0.955,1.134)	1.065(0.929,1.221)	1.074(0.914,1.262)	1.070(0.900,1.271)	1.056(0.881,1.267)
	Young	0.986(0.907,1.073)	1.002(0.877,1.146)	1.046(0.893,1.225)	1.114(0.941,1.319)	1.199(1.004,1.432)
	Old	1.084(0.996,1.180)	1.125(0.983,1.288)	1.124(0.958,1.318)	1.087(0.916,1.290)	1.032(0.861,1.236)
**$PM_{2.5}$**	Male	1.026(0.955,1.103)	1.048(0.932,1.179)	1.066(0.923,1.232)	1.081(0.916,1.275)	1.093(0.905,1.319)
	Female	1.059(0.984,1.139)	1.105(0.981,1.245)	1.138(0.983,1.318)	1.159(0.981,1.370)	1.171(0.969,1.417)
	Young	0.995(0.924,1.072)	1.007(0.893,1.135)	1.032(0.891,1.196)	1.070(0.906,1.264)	1.116(0.924,1.347)
	Old	1.062(0.990,1.140)	1.099(0.979,1.234)	1.109(0.961,1.280)	1.098(0.931,1.296)	1.074(0.889,1.299)
**PM_{10}**	Male	1.058(0.999,1.121)	1.136(1.033,1.249)	1.234(1.097,1.389)	1.353(1.179,1.553)	1.487(1.269,1.743)
	Female	1.068(1.007,1.133)	1.149(1.043,1.266)	1.243(1.101,1.403)	1.349(1.1711,1.553)	1.462(1.242,1.720)
	Young	1.023(0.963,1.087)	1.081(0.978,1.194)	1.175(1.038,1.329)	1.303(1.130,1.503)	1.460(1.240,1.717)
	Old	1.073(1.015,1.134)	1.145(1.044,1.255)	1.215(1.083,1.363)	1.283(1.122,1.468)	1.349(1.154,1.577)
**SO_2**	Male	0.970(0.869,1.084)	1.041(0.875,1.240)	1.220(0.995,1.496)	1.522(1.227,1.888)	1.947(1.554,2.438)
	Female	0.997(0.891,1.115)	1.071(0.897,1.279)	1.229(0.999,1.512)	1.480(1.189,1.843)	1.820(1.448,2.289)
	Young	1.007(0.905,1.121)	1.124(0.948,1.332)	1.372(1.125,1.673)	1.786(1.448,2.202)	2.383(1.913,2.968)
	Old	0.934(0.833,1.046)	0.939(0.783,1.124)	1.007(0.815,1.244)	1.134(0.906,1.418)	1.306(1.003,1.652)
**NO_2**	Male	1.005(0.947,1.068)	1.047(0.949,1.155)	1.125(0.997,1.268)	1.236(1.078,1.416)	1.371(1.176,1.599)
	Female	1.007(0.947,1.070)	1.043(0.944,1.153)	1.110(0.982,1.254)	1.204(1.048,1.382)	1.319(1.129,1.541)
	Young	0.990(0.931,1.053)	1.027(0.929,1.135)	1.111(0.983,1.256)	1.239(1.078,1.423)	1.401(1.199,1.636)
	Old	1.013(0.955,1.074)	1.050(0.954,1.155)	1.111(0.987,1.249)	1.193(1.044,1.365)	1.294(1.112,1.506)
**O_3**	Male	0.827(0.781,0.876)	0.683(0.622,0.750)	0.565(0.504,0.632)	0.467(0.412,0.530)	0.388(0.337,0.446)
	Female	0.865(0.815,0.917)	0.734(0.667,0.807)	0.612(0.545,0.687)	0.504(0.443,0.574)	0.413(0.358,0.477)
	Young	0.837(0.788,0.888)	0.702(0.637,0.773)	0.589(0.524,0.662)	0.496(0.436,0.564)	0.418(0.362,0.482)
	Old	0.879(0.831,0.930)	0.761(0.694,0.834)	0.649(0.580,0.725)	0.548(0.483,0.621)	0.462(0.401,0.531)

pollutant	group	Lag05	Lag06	Lag07	Lag08
		RR 95% CI	RR 95% CI	RR 95% CI	RR 95% CI
**CO**	Male	0.998(0.827,1.204)	0.988(0.818,1.194)	0.982(0.822,1.173)	0.979(0.824,1.163)
	Female	1.039(0.858,1.257)	1.021(0.844,1.237)	1.007(0.840,1.206)	0.996(0.836,1.186)
	Young	1.289(1.071,1.552)	1.370(1.137,1.650)	1.431(1.200,1.705)	1.463(1.234,1.734)
	Old	0.975(0.806,1.179)	0.927(0.766,1.123)	0.896(0.747,1.074)	0.882(0.740,1.052)
**$PM_{2.5}$**	Male	1.104(0.894,1.364)	1.116(0.888,1.402)	1.129(0.892,1.430)	1.144(0.899,1.457)
	Female	1.179(0.953,1.460)	1.187(0.943,1.495)	1.197(0.943,1.518)	1.210(0.948,1.545)
	Young	1.163(0.942,1.435)	1.206(0.962,1.511)	1.239(0.982,1.563)	1.260(0.993,1.598)
	Old	1.048(0.846,1.298)	1.028(0.815,1.296)	1.017(0.080,1.294)	1.019(0.796,1.305)
**PM_{10}**	Male	1.628(1.360,1.948)	1.763(1.451,2.143)	1.884(1.538,2.306)	1.980(1.606,2.442)
	Female	1.576(1.311,1.894)	1.682(1.378,2.054)	1.775(1.443,2.183)	1.847(1.491,2.287)
	Young	1.627(1.355,1.954)	1.786(1.466,2.175)	1.915(1.561,2.349)	1.998(1.617,2.468)
	Old	1.412(1.182,1.686)	1.472(1.214,1.785)	1.529(1.252,1.869)	1.583(1.287,1.948)
**SO_2**	Male	2.436(1.925,3.084)	2.873(2.267,3.641)	3.114(2.486,3.901)	3.061(2.449,3.826)
	Female	2.214(1.742,2.813)	2.592(2.038,3.298)	2.871(2.284,3.608)	2.980(2.378,3.736)
	Young	3.109(2.470,3.912)	3.814(3.028,4.805)	4.289(3.446,5.338)	4.363(3.515,5.415)
	Old	1.493(1.167,1.910)	1.652(1.289,2.118)	1.738(1.371,2.205)	1.726(1.363,2.184)
**NO_2**	Male	1.515(1.277,1.797)	1.645(1.370,1.975)	1.741(1.442,2.101)	1.788(1.474,2.170)
	Female	1.444(1.215,1.717)	1.565(1.300,1.883)	1.667(1.379,2.016)	1.742(1.433,2.118)
	Young	1.575(0.326,1.871)	1.731(1.441,2.081)	1.841(1.525,2.222)	1.883(1.553,2.284)
	Old	1.405(1.187,1.664)	1.517(1.265,1.819)	1.621(1.345,1.954)	1.712(1.413,2.074)
**O_3**	Male	0.323(0.277,0.377)	0.271(0.230,0.320)	0.229(0.193,0.272)	0.195(0.163,0.233)
	Female	0.340(0.291,0.399)	0.283(0.239,0.335)	0.239(0.201,0.285)	0.206(0.172,0.247)
	Young	0.352(0.301,0.412)	0.297(0.251,0.351)	0.250(0.211,0.297)	0.211(0.176,0.252)
	Old	0.391(0.335,0.457)	0.336(0.285,0.397)	0.294(0.247,0.350)	0.263(0.219,0.314)

Supplementary Table 17 Subgroup analysis by gender and age of single-day lagged relative risks (RRs) and 95% confidence intervals (CIs) for the impact of extremely high air pollution concentrations on CHD hospitalizations in Henan Province, 2016–2021(the maximum lag period = 8)

pollutant	group	Lag0	Lag1	Lag2	Lag3	Lag4
		RR 95% CI	RR 95% CI	RR 95% CI	RR 95% CI	RR 95% CI
**CO**	Male	1.017(0.935,1.107)	1.008(0.957,1.063)	1.000(0.963,1.039)	0.994(0.950,1.039)	0.990(0.941,1.041)
	Female	1.040(0.955,1.134)	1.024(0.971,1.080)	1.008(0.970,1.048)	0.996(0.952,1.042)	0.987(0.938,1.040)
	Young	0.986(0.907,1.073)	1.016(0.965,1.070)	1.044(1.005,1.084)	1.065(1.019,1.113)	1.076(1.024,1.132)
	Old	1.084(0.996,1.180)	1.038(0.985,1.094)	0.998(0.961,1.038)	0.968(0.925,1.012)	0.949(0.902,0.999)
**$PM_{2.5}$**	Male	1.026(0.955,1.103)	1.021(0.974,1.072)	1.017(0.977,1.059)	1.014(0.967,1.062)	1.011(0.960,1.065)
	Female	1.059(0.984,1.139)	1.044(0.994,1.096)	1.030(0.989,1.072)	1.018(0.972,1.068)	1.011(0.959,1.065)
	Young	0.995(0.924,1.072)	1.011(0.963,1.062)	1.026(0.985,1.067)	1.037(0.989,1.086)	1.043(0.990,1.098)
	Old	1.062(0.990,1.140)	1.034(0.986,1.085)	1.009(0.969,1.051)	0.990(0.945,1.038)	0.978(0.929,1.030)
**PM_{10}**	Male	1.058(0.999,1.121)	1.073(1.032,1.116)	1.087(1.050,1.124)	1.096(1.054,1.140)	1.099(1.053,1.147)
	Female	1.068(1.007,1.133)	1.076(1.033,1.120)	1.082(1.045,1.120)	1.085(1.042,1.130)	1.084(1.038,1.133)
	Young	1.023(0.963,1.087)	1.056(1.014,1.101)	1.087(1.050,1.125)	1.109(1.066,1.155)	1.120(1.072,1.170)
	Old	1.073(1.015,1.134)	1.067(1.027,1.109)	1.061(1.026,1.098)	1.056(1.016,1.098)	1.051(1.008,1.096)
**SO_2**	Male	0.97(0.869,1.084)	1.073(1.003,1.148)	1.172(1.117,1.229)	1.248(1.178,1.321)	1.279(1.198,1.366)
	Female	0.997(0.891,1.115)	1.074(1.004,1.150)	1.148(1.093,1.205)	1.204(1.136,1.276)	1.230(1.151,1.315)
	Young	1.007(0.905,1.121)	1.116(1.045,1.191)	1.221(1.165,1.279)	1.301(1.231,1.376)	1.334(1.251,1.423)
	Old	0.934(0.833,1.046)	1.005(0.938,1.078)	1.073(1.021,1.127)	1.126(1.061,1.194)	1.152(1.077,1.233)
**NO_2**	Male	1.005(0.947,1.068)	1.041(1.001,1.084)	1.074(1.039,1.110)	1.099(1.058,1.141)	1.110(1.065,1.157)
	Female	1.007(0.947,1.070)	1.036(0.995,1.079)	1.064(1.029,1.099)	1.085(1.044,1.127)	1.096(1.050,1.143)
	Young	0.990(0.931,1.053)	1.038(0.996,1.081)	1.082(1.046,1.118)	1.115(1.073,1.158)	1.131(1.084,1.180)
	Old	1.013(0.955,1.074)	1.036(0.997,1.078)	1.058(1.024,1.082)	1.075(1.035,1.115)	1.085(1.041,1.130)
**O_3**	Male	0.827(0.781,0.876)	0.826(0.796,0.858)	0.827(0.803,0.851)	0.828(0.800,0.856)	0.830(0.799,0.861)
	Female	0.865(0.815,0.917)	0.848(0.816,0.881)	0.834(0.809,0.860)	0.824(0.796,0.853)	0.820(0.789,0.852)
	Young	0.837(0.788,0.888)	0.838(0.807,0.871)	0.840(0.815,0.865)	0.841(0.813,0.871)	0.842(0.810,0.875)
	Old	0.879(0.831,0.930)	0.865(0.834,0.897)	0.853(0.828,0.878)	0.845(0.817,0.873)	0.843(0.812,0.875)

pollutant	group	Lag5 RR 95% CI	Lag6 RR 95% CI	Lag7 RR 95% CI	Lag8 RR 95% CI
**CO**	Male	0.989(0.944,1.036)	0.990(0.952,1.030)	0.993(0.946,1.043)	0.997(0.922,1.078)
	Female	0.984(0.939,1.031)	0.983(0.945,1.023)	0.986(0.938,1.036)	0.989(0.914,1.070)
	Young	1.075(1.027,1.125)	1.063(1.023,1.105)	1.044(0.995,1.096)	1.023(0.947,1.105)
	Old	0.945(0.902,0.990)	0.951(0.915,0.990)	0.966(0.919,1.015)	0.985(0.911,1.065)
**$PM_{2.5}$**	Male	1.010(0.964,1.060)	1.011(0.971,1.052)	1.012(0.967,1.058)	1.013(0.949,1.082)
	Female	1.007(0.960,1.056)	1.006(0.966,1.048)	1.008(0.964,1.055)	1.011(0.946,1.081)
	Young	1.042(0.994,1.093)	1.037(0.996,1.079)	1.028(0.982,1.075)	1.017(0.951,1.088)
	Old	0.976(0.929,1.030)	0.980(0.941,1.021)	0.990(0.947,1.035)	1.002(0.939,1.069)
**PM_{10}**	Male	1.094(1.052,1.138)	1.083(1.048,1.120)	1.068(1.028,1.110)	1.051(0.993,1.113)
	Female	1.078(1.036,1.122)	1.068(1.032,1.105)	1.055(1.014,1.097)	1.041(0.982,1.103)
	Young	1.115(1.071,1.160)	1.097(1.060,1.136)	1.072(1.030,1.116)	1.043(0.983,1.107)
	Old	1.047(1.007,1.088)	1.043(1.009,1.1078)	1.039(1.000,1.079)	1.035(1.979,1.094)
**SO_2**	Male	1.252(1.179,1.329)	1.179(1.122,1.240)	1.084(1.017,1.155)	0.983(0.888,1.088)
	Female	1.216(1.145,1.292)	1.171(1.113,1.232)	1.107(1.038,1.181)	1.038(0.937,1.151)
	Young	1.305(1.231,1.383)	1.227(1.168,1.289)	1.124(1.056,1.197)	1.017(0.921,1.124)
	Old	1.143(1.075,1.216)	1.106(1.050,1.165)	1.052(0.986,1.123)	0.993(0.895,1.101)
**NO_2**	Male	1.105(1.063,1.148)	1.086(1.050,1.123)	1.058(1.018,1.100)	1.027(0.971,1.087)
	Female	1.095(1.053,1.139)	1.083(1.047,1.121)	1.066(1.025,1.108)	1.045(1.987,1.107)
	Young	1.124(1.081,1.169)	1.099(1.063,1.137)	1.063(1.023,1.106)	1.023(0.966,1.084)
	Old	1.086(1.045,1.128)	1.080(1.044,1.116)	1.069(1.029,1.110)	1.056(0.999,1.115)
**O_3**	Male	0.834(0.805,0.863)	0.839(0.814,0.864)	0.845(0.816,0.874)	0.851(0.810,0.895)
	Female	0.823(0.794,0.853)	0.832(0.806,0.858)	0.845(0.816,0.875)	0.860(0.817,0.906)
	Young	0.843(0.813,0.874)	0.843(0.817,0.870)	0.843(0.814,0.873)	0.843(0.800,0.888)
	Old	0.848(0.819,0.878)	0.859(0.834,0.886)	0.875(0.846,0.905)	0.893(0.850,0.938)

Supplementary Table 18 Subgroup analysis by gender and age of cumulative lagged relative risks (RRs) and 95% confidence intervals (CIs) for the impact of extremely low air pollution concentrations on CHD hospitalizations in Henan Province, 2016–2021(the maximum lag period = 9)

pollutant	group	Lag00	Lag01	Lag02	Lag03	Lag04
		RR 95% CI	RR 95% CI	RR 95% CI	RR 95% CI	RR 95% CI
**CO**	Male	0.996(0.978,1.014)	0.996(0.967,1.025)	0.998(0.964,1.033)	1.002(0.966,1.040)	1.008(0.970,1.047)
	Female	0.992(0.974,1.011)	0.988(0.960,1.018)	0.988(0.954,1.023)	0.990(0.954,1.028)	0.994(0.956,1.034)
	Young	1.002(0.984,1.020)	1.001(0.973,1.030)	0.997(0.963,1.032)	0.990(0.955,1.027)	0.981(0.945,1.019)
	Old	0.984(0.966,1.002)	0.976(0.948,1.005)	0.976(0.942,1.010)	0.981(0.945,1.018)	0.991(0.953,1.030)
**$PM_{2.5}$**	Male	0.996(0.984,1.008)	0.992(0.973,1.013)	0.991(0.966,1.016)	0.990(0.962,1.018)	0.989(0.958,1.020)
	Female	0.991(0.979,1.003)	0.983(0.964,1.004)	0.978(0.954,1.003)	0.975(0.947,1.003)	0.972(0.942,1.004)
	Young	1.000(0.988,1.013)	0.999(0.979,1.020)	0.997(0.972,1.022)	0.993(0.965,1.021)	0.988(0.957,1.019)
	Old	0.990(0.978,1.002)	0.984(0.965,1.004)	0.982(0.958,1.007)	0.983(0.956,1.011)	0.986(0.955,1.017)
**PM_{10}**	Male	0.984(0.968,1.000)	0.966(0.941,0.992)	0.947(0.916,0.979)	0.926(0.892,0.962)	0.906(0.868,0.945)
	Female	0.982(0.966,0.998)	0.963(0.937,0.989)	0.943(0.912,0.976)	0.923(0.888,0.960)	0.903(0.865,0.944)
	Young	0.993(0.976,1.009)	0.979(0.952,1.006)	0.960(0.927,0.993)	0.936(0.900,0.974)	0.911(0.872,0.952)
	Old	0.981(0.966,0.996)	0.964(0.940,0.989)	0.949(0.919,0.980)	0.935(0.901,0.971)	0.923(0.885,0.963)
**SO_2**	Male	1.000(0.985,1.015)	0.992(0.969,1.017)	0.978(0.950,1.006)	0.957(0.929,0.986)	0.934(0.905,0.963)
	Female	0.999(0.984,1.015)	0.990(0.966,1.015)	0.973(0.946,1.002)	0.952(0.923,0.981)	0.927(0.898,0.957)
	Young	0.995(0.981,1.010)	0.982(0.959,1.006)	0.962(0.936,0.989)	0.936(0.909,0.964)	0.907(0.881,0.935)
	Old	1.008(0.993,1.025)	1.008(0.983,1.034)	1.001(0.971,1.031)	0.987(0.957,1.018)	0.970(0.939,1.002)
**NO_2**	Male	0.997(0.971,1.022)	0.982(0.941,1.024)	0.957(0.909,1.008)	0.925(0.873,0.981)	0.890(0.835,0.949)
	Female	0.998(0.972,1.024)	0.983(0.942,1.026)	0.958(0.908,1.009)	0.924(0.872,0.981)	0.888(0.832,0.947)
	Young	1.003(0.977,1.030)	0.990(0.948,1.033)	0.963(0.913,1.015)	0.925(0.873,0.982)	0.883(0.828,0.942)
	Old	0.995(0.971,1.020)	0.980(0.941,1.021)	0.956(0.909,1.006)	0.926(0.975,0.980)	0.893(0.838,0.950)
**O_3**	Male	1.121(1.081,1.162)	1.259(1.186,1.337)	1.418(1.316,1.527)	1.597(1.471,1.735)	1.799(1.644,1.968)
	Female	1.091(1.051,1.133)	1.207(1.135,1.284)	1.352(1.253,1.459)	1.527(1.403,1.662)	1.734(1.581,1.901)
	Young	1.111(1.070,1.154)	1.236(1.161,1.315)	1.375(1.274,1.485)	1.531(1.406,1.666)	1.705(1.555,1.869)
	Old	1.081(1.043,1.121)	1.182(1.114,1.255)	1.306(1.214,1.405)	1.453(1.338,1.577)	1.620(1.481,1.773)

pollutant	group	Lag05	Lag06	Lag07	Lag08	Lag09
		RR 95% CI	RR 95% CI	RR 95% CI	RR 95% CI	RR 95% CI
**CO**	Male	1.013(0.973,1.054)	1.015(0.974,1.059)	1.015(0.974,1.058)	1.011(0.972,1.050)	1.002(0.965,1.040)
	Female	0.998(0.958,1.040)	1.002(0.960,1.046)	1.004(0.962,1.047)	1.003(0.964,1.043)	0.999(0.962,1.038)
	Young	0.970(0.933,1.010)	0.958(0.919,0.999)	0.945(0.907,0.984)	0.931(0.896,0.967)	0.916(0.883,0.951)
	Old	1.002(0.962,1.044)	1.014(0.971,1.058)	1.022(0.980,1.066)	1.027(0.988,1.068)	1.027(0.989,1.067)
**$PM_{2.5}$**	Male	0.988(0.954,1.023)	0.986(0.949,1.025)	0.983(0.944,1.024)	0.978(0.939,1.019)	0.972(0.931,1.014)
	Female	0.971(0.937,1.006)	0.970(0.933,1.008)	0.969(0.930,1.009)	0.968(0.928,1.009)	0.967(0.926,1.009)
	Young	0.982(0.948,1.017)	0.976(0.939,1.013)	0.969(0.931,1.009)	0.963(0.924,1.002)	0.956(0.917,0.997)
	Old	0.989(0.955,1.025)	0.993(0.955,1.032)	0.996(0.955,1.037)	0.996(0.955,1.039)	0.995(0.953,1.039)
**PM_{10}**	Male	0.885(0.843,0.928)	0.864(0.820,0.912)	0.845(0.799,0.894)	0.827(0.780,0.877)	0.810(0.762,0.861)
	Female	0.885(0.842,0.930)	0.868(0.822,0.917)	0.854(0.806,0.905)	0.843(0.794,0.895)	0.835(0.785,0.888)
	Young	0.885(0.843,0.930)	0.861(0.816,0.909)	0.841(0.794,0.890)	0.824(0.777,0.874)	0.812(0.764,0.863)
	Old	0.912(0.870,0.957)	0.901(0.855,0.950)	0.891(0.842,0.942)	0.880(0.831,0.933)	0.870(0.819,0.923)
**SO_2**	Male	0.910(0.881,0.940)	0.888(0.859,0.919)	0.871(0.842,0.901)	0.858(0.831,0.886)	0.850(0.824,0.877)
	Female	0.903(0.874,0.933)	0.883(0.853,0.914)	0.868(0.839,0.898)	0.859(0.832,0.887)	0.858(0.831,0.886)
	Young	0.879(0.852,0.907)	0.854(0.826,0.882)	0.833(0.806,0.861)	0.818(0.793,0.844)	0.809(0.785,0.834)
	Old	0.953(0.921,0.986)	0.938(0.905,0.972)	0.928(0.896,0.962)	0.924(0.894,0.956)	0.927(0.897,0.958)
**NO_2**	Male	0.855(0.797,0.918)	0.824(0.763,0.890)	0.799(0.736,0.866)	0.780(0.718,0.848)	0.770(0.707,0.838)
	Female	0.853(0.794,0.916)	0.823(0.761,0.890)	0.801(0.738,0.870)	0.789(0.726,0.858)	0.786(0.722,0.857)
	Young	0.842(0.784,0.904)	0.806(0.746,0.871)	0.779(0.718,0.845)	0.762(0.701,0.828)	0.755(0.694,0.822)
	Old	0.860(0.802,0.923)	0.832(0.771,0.898)	0.811(0.748,0.879)	0.797(0.734,0.865)	0.791(0.727,0.860)
**O_3**	Male	2.021(1.832,2.230)	2.262(2.033,2.515)	2.516(2.250,2.812)	2.779(2.480,3.115)	3.048(2.711,3.428)
	Female	1.965(1.777,2.174)	2.212(1.984,2.466)	2.460(2.194,2.757)	2.695(2.398,3.028)	2.905(2.576,3.275)
	Young	1.898(1.717,2.098)	2.113(1.897,2.354)	2.352(2.101,2.633)	2.617(2.333,2.934)	2.910(2.586,3.274)
	Old	1.803(1.634,1.990)	1.990(1.788,2.215)	2.170(1.939,2.429)	2.331(2.077,2.617)	2.464(2.186,2.777)

Supplementary Table 19 Subgroup analysis by gender and age of single-day lagged relative risks (RRs) and 95% confidence intervals (CIs) for the impact of extremely low air pollution concentrations on CHD hospitalizations in Henan Province, 2016–2021(the maximum lag period = 9)

pollutant	group	Lag0	Lag1	Lag2	Lag3	Lag4
		RR 95% CI	RR 95% CI	RR 95% CI	RR 95% CI	RR 95% CI
**CO**	Male	0.996(0.978,1.014)	0.999(0.988,1.011)	1.002(0.995,1.010)	1.004(0.996,1.013)	1.005(0.995,1.016)
	Female	0.992(0.974,1.011)	0.996(0.985,1.008)	0.999(0.992,1.007)	1.002(0.994,1.011)	1.004(0.993,1.015)
	Young	1.002(0.984,1.020)	0.999(0.988,1.010)	0.996(0.988,1.004)	0.993(0.985,1.002)	0.991(0.981,1.001)
	Old	0.984(0.966,1.002)	0.992(0.981,1.003)	0.999(0.992,1.007)	1.006(0.997,1.014)	1.010(0.999,1.021)
**$PM_{2.5}$**	Male	0.996(0.984,1.008)	0.997(0.989,1.005)	0.998(0.992,1.005)	0.999(0.992,1.006)	0.999(0.991,1.008)
	Female	0.991(0.979,1.003)	0.993(0.984,1.001)	0.995(0.988,1.001)	0.996(0.989,1.003)	0.998(0.989,1.006)
	Young	1.000(0.988,1.013)	0.999(0.990,1.007)	0.997(0.991,1.004)	0.996(0.989,1.003)	0.995(0.987,1.003)
	Old	0.990(0.978,1.002)	0.994(0.986,1.002)	0.998(0.991,1.004)	1.001(0.994,1.008)	1.003(0.995,1.011)
**PM_{10}**	Male	0.984(0.968,1.000)	0.982(0.971,0.993)	0.980(0.971,0.989)	0.979(0.969,0.988)	0.977(0.967,0.988)
	Female	0.982(0.966,0.998)	0.981(0.970,0.992)	0.979(0.971,0.988)	0.979(0.969,0.988)	0.979(0.968,0.990)
	Young	0.993(0.976,1.009)	0.986(0.975,0.998)	0.980(0.972,0.989)	0.976(0.966,0.985)	0.973(0.962,0.984)
	Old	0.981(0.966,0.996)	0.983(0.972,0.993)	0.984(0.976,0.993)	0.986(0.977,0.995)	0.987(0.976,0.998)
**SO_2**	Male	1.000(0.985,1.015)	0.992(0.983,1.002)	0.985(0.979,0.991)	0.979(0.972,0.984)	0.976(0.967,0.984)
	Female	0.999(0.984,1.015)	0.991(0.981,1.000)	0.983(0.977,0.990)	0.978(0.971,0.985)	0.974(0.966,0.983)
	Young	0.995(0.981,1.010)	0.987(0.978,0.996)	0.979(0.973,0.985)	0.973(0.966,0.980)	0.969(0.961,0.978)
	Old	1.008(0.993,1.024)	1.000(0.990,1.010)	0.992(0.986,0.999)	0.986(0.979,0.994)	0.983(0.974,0.992)
**NO_2**	Male	0.997(0.971,1.022)	0.985(0.968,1.002)	0.975(0.962,0.988)	0.967(0.953,0.981)	0.962(0.946,0.978)
	Female	0.998(0.972,1.024)	0.985(0.968,1.003)	0.974(0.961,0.987)	0.965(0.951,0.980)	0.961(0.944,0.977)
	Young	1.003(0.977,1.030)	0.987(0.970,1.004)	0.973(0.960,0.986)	0.961(0.947,0.975)	0.954(0.938,0.971)
	Old	0.995(0.971,1.020)	0.985(0.968,1.001)	0.976(0.963,0.988)	0.968(0.955,0.982)	0.964(0.948,0.980)
**O_3**	Male	1.121(1.081,1.162)	1.124(1.096,1.151)	1.126(1.105,1.146)	1.127(1.105,1.149)	1.126(1.101,1.152)
	Female	1.091(1.051,1.133)	1.106(1.079,1.134)	1.120(1.099,1.141)	1.130(1.108,1.153)	1.135(1.109,1.162)
	Young	1.111(1.070,1.154)	1.112(1.084,1.141)	1.113(1.092,1.134)	1.113(1.091,1.135)	1.113(1.088,1.139)
	Old	1.081(1.043,1.121)	1.094(1.067,1.120)	1.104(1.085,1.125)	1.112(1.091,1.134)	1.116(1.091,1.141)

pollutant	group	Lag5 RR 95% CI	Lag6 RR 95% CI	Lag7 RR 95% CI	Lag8 RR 95% CI	Lag9 RR 95% CI
**CO**	Male	1.005(0.994,1.016)	1.003(0.994,1.012)	1.000(0.992,1.007)	0.996(0.985,1.006)	0.991(0.975,1.008)
	Female	1.004(0.994,1.015)	1.004(0.995,1.013)	1.002(0.994,1.010)	0.999(0.988,1.010)	0.996(0.980,1.014)
	Young	0.989(0.979,0.999)	0.987(0.979,0.996)	0.986(0.979,0.994)	0.985(0.975,0.996)	0.984(0.968,1.001)
	Old	1.012(1.001,1.023)	1.011(1.002,1.020)	1.009(1.001,1.017)	1.005(0.994,1.016)	1.000(0.984,1.017)
**$PM_{2.5}$**	Male	0.999(0.991,1.007)	0.998(0.991,1.007)	0.997(0.991,1.003)	0.995(0.987,1.003)	0.993(0.982,1.005)
	Female	0.999(0.990,1.007)	0.999(0.992,1.006)	0.999(0.993,1.005)	0.999(0.991,1.007)	0.999(0.987,1.010)
	Young	0.994(0.986,1.002)	0.994(0.987,1.001)	0.993(0.987,1.000)	0.993(0.985,1.001)	0.993(0.982,1.005)
	Old	1.004(0.996,1.012)	1.004(0.996,1.011)	1.003(0.996,1.009)	1.001(0.993,1.009)	0.999(0.988,1.010)
**PM_{10}**	Male	0.977(0.966,0.988)	0.977(0.968,0.987)	0.978(0.969,0.986)	0.978(0.968,0.989)	0.979(0.964,0.995)
	Female	0.980(0.969,0.991)	0.981(0.972,0.991)	0.984(0.975,0.993)	0.987(0.976,0.998)	0.990(0.974,1.006)
	Young	0.972(0.961,0.983)	0.973(0.964,0.983)	0.976(0.968,0.985)	0.980(0.970,0.991)	0.985(0.969,1.001)
	Old	0.988(0.977,0.999)	0.988(0.979,0.998)	0.988(0.980,0.997)	0.988(0.978,0.999)	0.988(0.973,1.003)
**SO_2**	Male	0.975(0.966,0.983)	0.976(0.969,0.984)	0.980(0.974,0.986)	0.985(0.977,0.994)	0.991(0.977,1.005)
	Female	0.974(0.965,0.983)	0.977(0.970,0.985)	0.983(0.977,0.989)	0.990(0.981,0.999)	0.998(0.984,1.013)
	Young	0.969(0.960,0.977)	0.971(0.964,0.978)	0.976(0.970,0.982)	0.982(0.974,0.990)	0.989(0.976,1.003)
	Old	0.982(0.973,0.991)	0.985(0.977,0.992)	0.989(0.983,0.996)	0.996(0.987,1.005)	1.003(0.989,1.017)
**NO_2**	Male	0.961(0.944,0.977)	0.963(0.949,0.978)	0.969(0.957,0.982)	0.977(0.961,0.993)	0.986(0.963,1.010)
	Female	0.960(0.944,0.977)	0.965(0.951,0.980)	0.974(0.961,0.987)	0.985(0.968,1.001)	0.997(0.973,1.022)
	Young	0.953(0.937,0.970)	0.958(0.943,0.972)	0.966(0.953,0.979)	0.978(0.962,0.994)	0.991(0.967,1.016)
	Old	0.964(0.948,0.980)	0.967(0.953,0.982)	0.974(0.961,0.987)	0.983(0.967,0.999)	0.993(0.969,1.016)
**O_3**	Male	1.124(1.098,1.150)	1.119(1.096,1.142)	1.112(1.092,1.133)	1.105(1.081,1.129)	1.097(1.063,1.132)
	Female	1.134(1.107,1.161)	1.125(1.102,1.149)	1.112(1.091,1.133)	1.096(1.071,1.121)	1.078(1.043,1.114)
	Young	1.114(1.088,1.140)	1.113(1.091,1.137)	1.113(1.093,1.134)	1.113(1.088,1.138)	1.112(1.076,1.149)
	Old	1.113(1.088,1.138)	1.104(1.082,1.126)	1.090(1.071,1.110)	1.074(1.051,1.098)	1.057(1.024,1.090)

Supplementary Table 20 Subgroup analysis by gender and age of cumulative lagged relative risks (RRs) and 95% confidence intervals (CIs) for the impact of extremely high air pollution concentrations on CHD hospitalizations in Henan Province, 2016–2021(the maximum lag period = 9)

pollutant	group	Lag00	Lag01	Lag02	Lag03	Lag04
		RR 95% CI	RR 95% CI	RR 95% CI	RR 95% CI	RR 95% CI
**CO**	Male	1.018(0.937,1.107)	1.021(0.892,1.169)	1.010(0.860,1.187)	0.989(0.833,1.175)	0.965(0.806,1.154)
	Female	1.036(0.952,1.128)	1.056(0.920,1.211)	1.058(0.898,1.247)	1.047(0.879,1.248)	1.028(0.857,1.234)
	Young	0.991(0.912,1.076)	0.995(0.870,1.138)	1.014(0.864,1.190)	1.046(0.883,1.241)	1.092(0.915,1.304)
	Old	1.078(0.991,1.172)	1.119(0.997,1.282)	1.122(0.954,1.320)	1.093(0.919,1.301)	1.044(0.871,1.251)
**$PM_{2.5}$**	Male	1.026(0.956,1.102)	1.045(0.929,1.176)	1.057(0.914,1.223)	1.064(0.902,1.254)	1.068(0.889,1.283)
	Female	1.056(0.983,1.136)	1.103(0.978,1.243)	1.138(0.982,1.319)	1.163(0.984,1.374)	1.179(0.979,1.420)
	Young	0.998(0.927,1.073)	1.005(0.891,1.133)	1.020(0.880,1.184)	1.044(0.884,1.233)	1.076(0.895,1.293)
	Old	1.059(0.987,1.136)	1.097(0.976,1.232)	1.112(0.962,1.285)	1.107(0.939,1.305)	1.089(0.905,1.309)
**PM_{10}**	Male	1.060(1.001,1.122)	1.132(1.030,1.245)	1.217(1.081,1.370)	1.315(1.148,1.507)	1.427(1.224,1.664)
	Female	1.067(1.007,1.132)	1.145(1.039,1.262)	1.234(1.093,1.393)	1.333(1.160,1.532)	1.440(1.231,1.686)
	Young	1.027(0.967,1.091)	1.080(0.977,1.193)	1.159(1.024,1.313)	1.266(1.100,1.458)	1.399(1.195,1.637)
	Old	1.072(1.014,1.132)	1.141(1.041,1.251)	1.207(1.075,1.355)	1.270(1.112,1.451)	1.331(1.144,1.548)
**SO_2**	Male	0.998(0.896,1.113)	1.056(0.887,1.257)	1.177(0.959,1.446)	1.369(1.103,1.700)	1.637(1.310,2.045)
	Female	1.006(0.902,1.124)	1.076(0.901,1.284)	1.214(0.985,1.497)	1.430(1.147,1.783)	1.725(1.375,2.165)
	Young	1.034(0.931,1.149)	1.137(0.960,1.347)	1.323(1.084,1.616)	1.610(1.304,1.986)	2.013(1.621,2.500)
	Old	0.942(0.842,1.054)	0.943(0.787,1.129)	0.996(0.805,1.234)	1.099(0.877,1.377)	1.245(0.987,1.572)
**NO_2**	Male	1.008(0.950,1.070)	1.044(0.946,1.152)	1.107(0.981,1.250)	1.198(1.046,1.372)	1.312(1.130,1.523)
	Female	1.005(0.946,1.068)	1.041(0.942,1.150)	1.107(0.979,1.251)	1.201(1.047,1.378)	1.319(1.134,1.535)
	Young	0.993(0.934,1.056)	1.024(0.926,1.132)	1.093(0.966,1.236)	1.198(1.044,1.375)	1.336(1.148,1.554)
	Old	1.011(0.954,1.072)	1.048(0.952,1.154)	1.110(0.987,1.250)	1.197(1.048,1.367)	1.304(1.126,1.510)
**O_3**	Male	0.838(0.792,0.887)	0.700(0.638,0.769)	0.583(0.520,0.654)	0.485(0.427,0.551)	0.403(0.351,0.463)
	Female	0.874(0.824,0.926)	0.747(0.679,0.822)	0.627(0.558,0.706)	0.519(0.456,0.592)	0.427(0.370,0.492)
	Young	0.849(0.801,0.900)	0.721(0.654,0.793)	0.611(0.543,0.688)	0.518(0.454,0.590)	0.438(0.380,0.505)
	Old	0.886(0.838,0.937)	0.772(0.704,0.846)	0.662(0.591,0.741)	0.561(0.495,0.637)	0.474(0.413,0.545)

pollutant	group	Lag05		Lag06		Lag07		Lag08		Lag09	
		RR	95% CI	RR	95% CI	RR	95% CI	RR	95% CI	RR	95% CI
**CO**	Male	0.943(0.781,1.139)		0.931(0.765,1.132)		0.933(0.769,1.131)		0.952(0.795,1.140)		0.992(0.833,1.182)	
	Female	1.007(0.832,1.220)		0.991(0.812,1.208)		0.983(0.808,1.195)		0.987(0.822,1.184)		1.003(0.840,1.198)	
	Young	1.150(0.955,1.385)		1.220(1.007,1.480)		1.302(1.077,1.575)		1.396(1.169,1.667)		1.503(1.266,1.786)	
	Old	0.989(0.817,1.196)		0.939(0.770,1.145)		0.902(0.742,1.097)		0.882(0.735,1.060)		0.881(0.738,1.053)	
**$PM_{2.5}$**	Male	1.074(0.875,1.318)		1.086(0.867,1.359)		1.106(0.873,1.402)		1.138(0.894,1.449)		1.184(0.924,1.516)	
	Female	1.189(0.967,1.463)		1.197(0.954,1.501)		1.203(0.947,1.529)		1.211(0.948,1.546)		1.220(0.950,1.568)	
	Young	1.113(0.908,1.366)		1.156(0.925,1.444)		1.202(0.951,1.519)		1.251(0.986,1.587)		1.302(1.020,1.661)	
	Old	1.065(0.865,1.309)		1.042(0.830,1.309)		1.027(0.807,1.307)		1.021(0.798,1.308)		1.028(0.797,1.325)	
**PM_{10}**	Male	1.551(1.305,1.844)		1.686(1.393,2.040)		1.829(1.493,2.239)		1.977(1.604,2.437)		2.130(1.714,2.647)	
	Female	1.551(1.299,1.852)		1.659(1.365,2.017)		1.758(1.429,2.163)		1.844(1.489,2.282)		1.910(1.531,2.384)	
	Young	1.549(1.299,1.848)		1.708(1.407,2.072)		1.861(1.517,2.284)		1.998(1.618,2.467)		2.108(1.694,2.625)	
	Old	1.391(1.172,1.649)		1.451(1.202,1.752)		1.514(1.239,1.850)		1.580(1.285,1.943)		1.651(1.331,2.046)	
**SO_2**	Male	1.971(1.560,2.490)		2.344(1.836,2.991)		2.710(2.126,3.455)		3.018(2.400,3.796)		3.220(2.568,4.038)	
	Female	2.082(1.642,2.642)		2.456(1.917,3.146)		2.779(2.173,3.553)		2.981(2.364,3.759)		3.014(2.397,3.791)	
	Young	2.530(2.015,3.176)		3.125(2.465,3.961)		3.728(2.945,4.720)		4.249(3.402,5.307)		4.600(3.693,5.728)	
	Old	1.417(1.110,1.809)		1.584(1.227,2.044)		1.710(1.326,2.205)		1.764(1.386,2.246)		1.729(1.361,2.196)	
**NO_2**	Male	1.441(1.221,1.700)		1.572(1.312,1.882)		1.690(1.398,2.043)		1.784(1.471,2.163)		1.842(1.511,2.246)	
	Female	1.450(1.226,1.714)		1.575(1.312,1.890)		1.676(1.384,2.031)		1.739(1.431,2.113)		1.751(1.434,2.139)	
	Young	1.494(1.264,1.765)		1.653(1.379,1.982)		1.791(1.481,2.166)		1.886(1.555,2.287)		1.925(1.580,2.346)	
	Old	1.421(1.207,1.673)		1.535(1.284,1.835)		1.632(1.352,1.970)		1.700(1.403,2.059)		1.729(1.420,2.106)	
**O_3**	Male	0.337(0.289,0.392)		0.283(0.240,0.334)		0.240(0.202,0.285)		0.206(0.173,0.245)		0.178(0.149,0.214)	
	Female	0.352(0.301,0.411)		0.293(0.248,0.347)		0.249(0.208,0.297)		0.216(0.180,0.258)		0.192(0.160,0.231)	
	Young	0.371(0.318,0.433)		0.314(0.266,0.371)		0.266(0.224,0.317)		0.226(0.189,0.270)		0.192(0.160,0.230)	
	Old	0.402(0.345,0.468)		0.345(0.292,0.407)		0.302(0.253,0.359)		0.270(0.226,0.323)		0.248(0.206,0.298)	

Supplementary Table 21 Subgroup analysis by gender and age of single-day lagged relative risks (RRs) and 95% confidence intervals (CIs) for the impact of extremely high air pollution concentrations on CHD hospitalizations in Henan Province, 2016–2021(the maximum lag period = 9)

pollutant	group	Lag0	Lag1	Lag2	Lag3	Lag4
		RR 95% CI	RR 95% CI	RR 95% CI	RR 95% CI	RR 95% CI
**CO**	Male	1.018(0.937,1.107)	1.003(0.951,1.058)	0.989(0.954,1.026)	0.979(0.941,1.019)	0.975(0.929,1.024)
	Female	1.036(0.952,1.128)	1.019(0.965,1.075)	1.003(0.966,1.040)	0.990(0.950,1.031)	0.982(0.935,1.031)
	Young	0.991(0.912,1.076)	1.005(0.953,1.059)	1.019(0.983,1.056)	1.032(0.992,1.073)	1.044(0.995,1.095)
	Old	1.078(0.991,1.172)	1.038(0.984,1.095)	1.003(0.967,1.040)	0.974(0.936,1.014)	0.955(0.909,1.003)
**$PM_{2.5}$**	Male	1.026(0.956,1.102)	1.018(0.970,1.069)	1.011(0.974,1.050)	1.006(0.965,1.049)	1.004(0.957,1.053)
	Female	1.056(0.983,1.136)	1.044(0.994,1.096)	1.032(0.994,1.072)	1.022(0.980,1.066)	1.014(0.966,1.064)
	Young	0.998(0.927,1.073)	1.007(0.959,1.058)	1.016(0.978,1.055)	1.024(0.982,1.067)	1.030(0.982,1.081)
	Old	1.059(0.987,1.136)	1.035(0.987,1.086)	1.014(0.976,1.053)	0.996(0.955,1.038)	0.983(0.937,1.032)
**PM_{10}**	Male	1.060(1.001,1.122)	1.068(1.027,1.111)	1.075(1.042,1.109)	1.081(1.044,1.119)	1.085(1.043,1.129)
	Female	1.067(1.007,1.132)	1.073(1.031,1.117)	1.078(1.043,1.113)	1.080(1.043,1.119)	1.080(1.038,1.125)
	Young	1.027(0.967,1.091)	1.051(1.009,1.095)	1.074(1.040,1.109)	1.092(1.054,1.132)	1.104(1.061,1.150)
	Old	1.072(1.014,1.132)	1.065(1.025,1.106)	1.058(1.026,1.091)	1.052(1.017,1.089)	1.048(1.008,1.089)
**SO_2**	Male	0.998(0.896,1.113)	1.058(0.988,1.132)	1.115(1.066,1.166)	1.163(1.106,1.224)	1.195(1.123,1.273)
	Female	1.006(0.902,1.124)	1.069(0.998,1.145)	1.129(1.078,1.182)	1.177(1.118,1.240)	1.206(1.132,1.286)
	Young	1.034(0.931,1.149)	1.100(1.030,1.175)	1.163(1.113,1.215)	1.216(1.158,1.278)	1.250(1.176,1.330)
	Old	0.942(0.842,1.054)	1.001(0.933,1.074)	1.057(1.008,1.107)	1.103(1.047,1.163)	1.133(1.062,1.209)
**NO_2**	Male	1.008(0.950,1.070)	1.035(0.995,1.078)	1.061(1.029,1.094)	1.082(1.046,1.119)	1.095(1.053,1.139)
	Female	1.005(0.946,1.068)	1.035(0.994,1.078)	1.063(1.031,1.097)	1.086(1.049,1.123)	1.098(1.056,1.143)
	Young	0.993(0.934,1.056)	1.031(0.990,1.074)	1.067(1.035,1.100)	1.096(1.060,1.134)	1.115(1.071,1.160)
	Old	1.011(0.954,1.072)	1.036(0.997,1.078)	1.060(1.028,1.092)	1.078(1.043,1.114)	1.089(1.048,1.132)
**O_3**	Male	1.121(1.081,1.162)	1.124(1.096,1.151)	1.126(1.105,1.146)	1.127(1.105,1.149)	1.126(1.101,1.152)
	Female	1.091(1.051,1.133)	1.106(1.079,1.134)	1.120(1.099,1.141)	1.130(1.108,1.153)	1.135(1.109,1.162)
	Young	1.111(1.070,1.154)	1.112(1.084,1.141)	1.113(1.092,1.134)	1.113(1.091,1.135)	1.113(1.088,1.139)
	Old	1.081(1.043,1.121)	1.094(1.067,1.120)	1.104(1.085,1.125)	1.112(1.091,1.134)	1.116(1.091,1.141)

pollutant	group	Lag5 RR 95% CI	Lag6 RR 95% CI	Lag7 RR 95% CI	Lag8 RR 95% CI	Lag9 RR 95% CI
**CO**	Male	0.978(0.931,1.027)	0.987(0.947,1.029)	1.002(0.966,1.039)	1.021(0.971,1.074)	1.042(0.964,1.127)
	Female	0.980(0.932,1.030)	0.984(0.943,1.026)	0.992(0.956,1.029)	1.004(0.954,1.056)	1.017(0.939,1.101)
	Young	1.053(1.004,1.106)	1.061(1.019,1.105)	1.067(1.030,1.106)	1.072(1.020,1.126)	1.077(0.997,1.163)
	Old	0.947(0.901,0.995)	0.950(0.911,0.990)	0.961(0.926,0.997)	0.978(0.930,1.029)	0.999(0.923,1.081)
**$PM_{2.5}$**	Male	1.006(0.958,1.055)	1.011(0.969,1.054)	1.019(0.982,1.057)	1.029(0.983,1.077)	1.040(0.973,1.112)
	Female	1.009(0.961,1.059)	1.006(0.964,1.050)	1.005(0.968,1.044)	1.006(0.961,1.054)	1.008(0.942,1.078)
	Young	1.035(0.986,1.086)	1.038(0.995,1.083)	1.040(1.002,1.079)	1.041(0.994,1.090)	1.041(0.973,1.114)
	Old	0.978(0.932,1.026)	0.979(0.938,1.021)	0.985(0.949,1.023)	0.995(0.951,1.041)	1.006(0.942,1.075)
**PM_{10}**	Male	1.087(1.045,1.131)	1.087(1.050,1.125)	1.085(1.051,1.119)	1.081(1.040,1.124)	1.077(1.018,1.140)
	Female	1.077(1.034,1.121)	1.070(1.033,1.108)	1.060(1.027,1.094)	1.048(1.008,1.091)	1.036(0.978,1.098)
	Young	1.108(1.064,1.154)	1.102(1.064,1.142)	1.090(1.056,1.125)	1.074(1.031,1.117)	1.055(0.995,1.119)
	Old	1.045(1.005,1.086)	1.043(1.009,1.080)	1.043(1.012,1.076)	1.044(1.005,1.084)	1.045(0.989,1.104)
**SO_2**	Male	1.204(1.130,1.284)	1.189(1.127,1.254)	1.157(1.105,1.211)	1.114(1.046,1.186)	1.067(0.966,1.179)
	Female	1.207(1.131,1.288)	1.179(1.117,1.245)	1.132(1.080,1.186)	1.073(1.006,1.144)	1.011(0.913,1.119)
	Young	1.257(1.181,1.338)	1.235(1.172,1.301)	1.193(1.141,1.248)	1.140(1.071,1.213)	1.082(0.982,1.194)
	Old	1.138(1.065,1.215)	1.118(1.058,1.181)	1.080(1.030,1.133)	1.032(0.966,1.101)	0.980(0.884,1.086)
**NO_2**	Male	1.098(1.055,1.143)	1.091(1.054,1.129)	1.075(1.043,1.109)	1.055(1.016,1.096)	1.033(0.976,1.093)
	Female	1.099(1.055,1.144)	1.086(1.049,1.125)	1.065(1.032,1.098)	1.037(0.997,1.078)	1.007(0.951,1.067)
	Young	1.118(1.074,1.164)	1.106(1.068,1.146)	1.083(1.050,1.118)	1.053(1.013,1.095)	1.021(1.964,1.081)
	Old	1.090(1.048,1.133)	1.080(1.044,1.118)	1.063(1.031,1.096)	1.041(1.003,1.081)	1.018(0.963,1.075)
**O_3**	Male	1.124(1.098,1.150)	1.119(1.096,1.142)	1.112(1.092,1.133)	1.105(1.081,1.129)	1.097(1.063,1.132)
	Female	1.134(1.107,1.161)	1.125(1.102,1.149)	1.112(1.091,1.133)	1.096(1.071,1.121)	1.078(1.043,1.114)
	Young	1.114(1.088,1.140)	1.113(1.091,1.137)	1.113(1.093,1.134)	1.113(1.088,1.138)	1.112(1.076,1.149)
	Old	1.113(1.088,1.138)	1.104(1.082,1.126)	1.090(1.071,1.110)	1.074(1.051,1.098)	1.057(1.024,1.090)

Supplementary Table 22 Subgroup analysis by gender and age of cumulative lagged relative risks (RRs) and 95% confidence intervals (CIs) for the impact of extremely low air pollution concentrations on CHD hospitalizations in Henan Province, 2016–2021(df of temperature = 2)

pollutant	group	Lag00	Lag01	Lag02	Lag03	Lag04	Lag05	Lag06	Lag07
		RR 95% CI	RR 95% CI	RR 95% CI	RR 95% CI	RR 95% CI	RR 95% CI	RR 95% CI	RR 95% CI
**CO**	Male	0.996(0.978,1.014)	0.993(0.965,1.022)	0.992(0.960,1.025)	0.992(0.958,1.027)	0.993(0.957,1.031)	0.996(0.958,1.035)	0.999(0.963,1.037)	1.004(0.968,1.041)
	Female	0.989(0.970,1.007)	0.985(0.958,1.014)	0.989(0.957,1.023)	0.997(0.963,1.033)	1.006(0.968,1.045)	1.010(0.972,1.051)	1.008(0.971,1.047)	0.999(0.962,1.036)
	Young	1.001(0.983,1.020)	0.996(0.968,1.024)	0.984(0.953,1.017)	0.969(0.936,1.003)	0.953(0.918,0.989)	0.939(0.904,0.976)	0.929(0.895,0.964)	0.924(0.891,0.957)
	Old	0.981(0.963,0.999)	0.975(0.948,1.003)	0.980(0.948,1.012)	0.992(0.957,1.027)	1.007(0.969,1.046)	1.019(0.980,1.060)	1.026(0.988,1.065)	1.024(0.987,1.063)
**$PM_{2.5}$**	Male	0.995(0.983,1.008)	0.992(0.972,1.011)	0.988(0.964,1.012)	0.985(0.958,1.014)	0.983(0.951,1.016)	0.981(0.946,1.018)	0.981(0.943,1.019)	0.980(0.942,1.021)
	Female	0.990(0.977,1.002)	0.982(0.963,1.002)	0.978(0.954,1.003)	0.976(0.949,1.005)	0.975(0.943,1.008)	0.974(0.939,1.011)	0.972(0.935,1.011)	0.970(0.931,1.010)
	Young	1.000(0.988,1.013)	0.997(0.977,1.018)	0.991(0.967,1.016)	0.983(0.956,1.012)	0.975(0.944,1.008)	0.969(0.935,1.005)	0.966(0.930,1.003)	0.965(0.928,1.005)
	Old	0.989(0.978,1.002)	0.985(0.966,1.004)	0.984(0.961,1.009)	0.988(0.960,1.016)	0.992(0.960,1.026)	0.996(0.960,1.034)	0.998(0.960,1.038)	0.998(0.957,1.040)
**PM_{10}**	Male	0.983(0.968,0.999)	0.962(0.937,0.988)	0.938(0.908,0.969)	0.912(0.877,0.948)	0.887(0.848,0.928)	0.866(0.823,0.911)	0.848(0.804,0.896)	0.836(0.790,0.885)
	Female	0.980(0.964,0.996)	0.960(0.935,0.986)	0.941(0.910,0.973)	0.922(0.886,0.959)	0.903(0.862,0.946)	0.885(0.840,0.932)	0.866(0.819,0.915)	0.847(0.799,0.898)
	Young	0.993(0.976,1.010)	0.976(0.949,1.003)	0.950(0.919,0.983)	0.921(0.885,0.958)	0.891(0.850,0.933)	0.865(0.821,0.911)	0.845(0.800,0.893)	0.833(0.787,0.882)
	Old	0.979(0.964,0.994)	0.961(0.937,0.986)	0.947(0.917,0.977)	0.934(0.900,0.971)	0.923(0.883,0.965)	0.911(0.867,0.958)	0.898(0.852,0.948)	0.884(0.836,0.935)
**SO_2**	Male	1.004(0.988,1.019)	0.995(0.971,1.019)	0.976(0.949,1.004)	0.950(0.922,0.979)	0.921(0.893,0.951)	0.894(0.866,0.923)	0.871(0.845,0.898)	0.853(0.827,0.879)
	Female	1.000(0.984,1.016)	0.991(0.967,1.015)	0.974(0.947,1.002)	0.952(0.924,0.981)	0.927(0.898,0.957)	0.902(0.873,0.932)	0.879(0.852,0.907)	0.858(0.832,0.885)
	Young	0.999(0.984,1.014)	0.984(0.961,1.008)	0.959(0.933,0.985)	0.926(0.900,0.953)	0.892(0.865,0.920)	0.860(0.834,0.888)	0.834(0.810,0.859)	0.814(0.790,0.838)
	Old	1.009(0.993,1.025)	1.010(0.985,1.035)	1.004(0.975,1.033)	0.992(0.962,1.023)	0.976(0.944,1.008)	0.958(0.927,0.991)	0.941(0.911,0.972)	0.923(0.894,0.953)
**NO_2**	Male	0.996(0.971,1.022)	0.979(0.939,1.020)	0.951(0.904,1.000)	0.916(0.864,0.971)	0.878(0.821,0.938)	0.841(0.782,0.905)	0.809(0.748,0.874)	0.780(0.720,0.846)
	Female	0.994(0.968,1.020)	0.978(0.937,1.020)	0.954(0.906,1.004)	0.924(0.871,0.980)	0.891(0.833,0.953)	0.858(0.796,0.924)	0.825(0.763,0.892)	0.794(0.732,0.862)
	Young	1.002(0.976,1.029)	0.987(0.946,1.029)	0.957(0.909,1.007)	0.917(0.865,0.973)	0.874(0.817,0.935)	0.832(0.773,0.896)	0.795(0.736,0.859)	0.763(0.704,0.827)
	Old	0.991(0.967,1.016)	0.975(0.936,1.015)	0.951(0.905,0.999)	0.923(0.871,0.977)	0.892(0.835,0.953)	0.861(0.801,0.926)	0.831(0.770,0.898)	0.803(0.741,0.871)
**O_3**	Male	1.138(1.096,1.182)	1.301(1.225,1.381)	1.489(1.385,1.601)	1.703(1.569,1.848)	1.936(1.765,2.123)	2.179(1.969,2.411)	2.419(2.175,2.691)	2.646(2.367,2.958)
	Female	1.103(1.061,1.146)	1.241(1.167,1.320)	1.420(1.319,1.530)	1.639(1.507,1.783)	1.886(1.716,2.073)	2.138(1.928,2.372)	2.370(2.126,2.643)	2.559(2.283,2.867)
	Young	1.130(1.086,1.175)	1.282(1.204,1.364)	1.458(1.353,1.571)	1.658(1.525,1.802)	1.874(1.707,2.059)	2.097(1.893,2.323)	2.314(2.079,2.576)	2.514(2.247,2.812)
	Old	1.091(1.051,1.132)	1.207(1.138,1.280)	1.352(1.258,1.452)	1.523(1.403,1.652)	1.711(1.560,1.876)	1.900(1.716,2.103)	2.074(1.863,2.309)	2.219(1.982,2.483)

Supplementary Table 23 Subgroup analysis by gender and age of single-day lagged relative risks (RRs) and 95% confidence intervals (CIs) for the impact of extremely low air pollution concentrations on CHD hospitalizations in Henan Province, 2016–2021(df of temperature = 2)

pollutant	group	Lag0	Lag1	Lag2	Lag3	Lag4	Lag5	Lag6	Lag7
		RR 95% CI	RR 95% CI	RR 95% CI	RR 95% CI	RR 95% CI	RR 95% CI	RR 95% CI	RR 95% CI
**CO**	Male	0.996(0.978,1.014)	0.997(0.987,1.008)	0.999(0.990,1.007)	1.000(0.989,1.011)	1.001(0.990,1.012)	1.003(0.993,1.012)	1.004(0.993,1.014)	1.005(0.988,1.022)
	Female	0.989(0.970,1.007)	0.997(0.986,1.008)	1.004(0.995,1.013)	1.008(0.997,1.019)	1.008(0.997,1.020)	1.005(0.995,1.014)	0.998(0.988,1.008)	0.990(0.974,1.007)
	Young	1.001(0.983,1.020)	0.994(0.984,1.005)	0.988(0.980,0.997)	0.984(0.974,0.995)	0.983(0.973,0.994)	0.985(0.977,0.994)	0.989(0.979,0.999)	0.994(0.978,1.011)
	Old	0.981(0.963,0.999)	0.994(0.983,1.004)	1.005(0.996,1.014)	1.012(1.002,1.023)	1.015(1.004,1.026)	1.012(1.003,1.022)	1.006(0.996,1.017)	0.999(0.982,1.015)
**$PM_{2.5}$**	Male	0.995(0.983,1.008)	0.996(0.988,1.004)	0.997(0.989,1.004)	0.997(0.988,1.006)	0.998(0.989,1.007)	0.998(0.991,1.006)	0.999(0.991,1.007)	1.000(0.989,1.011)
	Female	0.990(0.977,1.002)	0.993(0.985,1.001)	0.996(0.988,1.003)	0.998(0.989,1.007)	0.999(0.990,1.008)	0.999(0.991,1.007)	0.998(0.990,1.006)	0.997(0.986,1.009)
	Young	1.000(0.988,1.013)	0.997(0.989,1.005)	0.994(0.987,1.001)	0.992(0.983,1.001)	0.992(0.983,1.001)	0.994(0.986,1.001)	0.996(0.989,1.004)	1.000(0.988,1.011)
	Old	0.989(0.978,1.002)	0.995(0.987,1.003)	1.000(0.992,1.007)	1.003(0.995,1.012)	1.005(0.996,1.014)	1.004(0.996,1.012)	1.002(0.994,1.010)	0.999(0.988,1.010)
**PM_{10}**	Male	0.983(0.968,0.999)	0.979(0.968,0.989)	0.975(0.965,0.985)	0.972(0.961,0.984)	0.973(0.961,0.985)	0.976(0.966,0.986)	0.980(0.970,0.991)	0.986(0.970,1.002)
	Female	0.980(0.964,0.996)	0.980(0.969,0.991)	0.980(0.970,0.990)	0.980(0.968,0.992)	0.980(0.968,0.992)	0.979(0.969,0.990)	0.979(0.968,0.990)	0.978(0.962,0.994)
	Young	0.993(0.976,1.010)	0.983(0.972,0.994)	0.974(0.964,0.984)	0.969(0.957,0.981)	0.968(0.956,0.980)	0.971(0.961,0.981)	0.977(0.967,0.989)	0.986(0.969,1.002)
	Old	0.979(0.964,0.994)	0.982(0.972,0.992)	0.985(0.975,0.995)	0.987(0.975,0.999)	0.988(0.976,1.000)	0.987(0.977,0.997)	0.986(0.975,0.996)	0.984(0.969,1.000)
**SO_2**	Male	1.004(0.988,1.019)	0.992(0.983,1.001)	0.981(0.974,0.988)	0.973(0.965,0.982)	0.970(0.961,0.979)	0.971(0.963,0.978)	0.974(0.965,0.983)	0.979(0.965,0.993)
	Female	1.000(0.984,1.016)	0.991(0.982,1.000)	0.983(0.976,0.990)	0.977(0.968,0.986)	0.974(0.965,0.983)	0.973(0.966,0.981)	0.974(0.966,0.983)	0.976(0.962,0.991)
	Young	0.999(0.984,1.014)	0.986(0.977,0.994)	0.974(0.967,0.981)	0.966(0.958,0.975)	0.963(0.954,0.972)	0.965(0.957,0.972)	0.969(0.961,0.978)	0.976(0.962,0.990)
	Old	1.009(0.993,1.025)	1.001(0.992,1.011)	0.994(0.986,1.001)	0.988(0.979,0.997)	0.984(0.975,0.993)	0.982(0.974,0.990)	0.981(0.973,0.990)	0.981(0.967,0.996)
**NO_2**	Male	0.996(0.971,1.022)	0.983(0.967,0.999)	0.971(0.957,0.986)	0.963(0.946,0.980)	0.959(0.941,0.976)	0.958(0.943,0.974)	0.961(0.945,0.977)	0.965(0.942,0.989)
	Female	0.994(0.968,1.020)	0.984(0.968,1.001)	0.976(0.961,0.991)	0.969(0.951,0.986)	0.964(0.947,0.982)	0.962(0.947,0.978)	0.962(0.946,0.978)	0.963(0.939,0.987)
	Young	1.002(0.976,1.029)	0.985(0.968,1.002)	0.970(0.955,0.985)	0.959(0.941,0.976)	0.953(0.935,0.971)	0.952(0.937,0.967)	0.955(0.939,0.971)	0.960(0.937,0.984)
	Old	0.991(0.967,1.016)	0.983(0.967,0.999)	0.976(0.962,0.991)	0.970(0.953,0.987)	0.967(0.950,0.984)	0.965(0.950,0.980)	0.965(0.950,0.981)	0.966(0.944,0.989)
**O_3**	Male	1.138(1.096,1.182)	1.143(1.116,1.170)	1.145(1.122,1.169)	1.144(1.116,1.172)	1.137(1.109,1.166)	1.125(1.101,1.150)	1.110(1.086,1.135)	1.094(1.059,1.130)
	Female	1.103(1.061,1.146)	1.126(1.098,1.153)	1.144(1.120,1.169)	1.154(1.126,1.183)	1.151(1.121,1.181)	1.134(1.109,1.159)	1.108(1.083,1.134)	1.079(1.044,1.117)
	Young	1.130(1.086,1.175)	1.135(1.107,1.163)	1.138(1.114,1.162)	1.137(1.109,1.166)	1.131(1.102,1.160)	1.119(1.094,1.144)	1.103(1.078,1.129)	1.086(1.050,1.124)
	Old	1.091(1.051,1.132)	1.107(1.081,1.133)	1.120(1.097,1.143)	1.127(1.100,1.154)	1.123(1.096,1.152)	1.111(1.087,1.135)	1.092(1.068,1.116)	1.070(1.036,1.105)

Supplementary Table 24 Subgroup analysis by gender and age of cumulative lagged relative risks (RRs) and 95% confidence intervals (CIs) for the impact of extremely high air pollution concentrations on CHD hospitalizations in Henan Province, 2016–2021(df of temperature = 2)

pollutant	group	Lag00	Lag01	Lag02	Lag03	Lag04	Lag05	Lag06	Lag07
		RR 95% CI	RR 95% CI	RR 95% CI	RR 95% CI	RR 95% CI	RR 95% CI	RR 95% CI	RR 95% CI
**CO**	Male	1.020(0.936,1.110)	1.033(0.905,1.178)	1.039(0.892,1.210)	1.039(0.882,1.224)	1.033(0.867,1.230)	1.021(0.852,1.222)	1.003(0.843,1.194)	0.981(0.827,1.164)
	Female	1.055(0.968,1.150)	1.071(0.936,1.224)	1.052(0.901,1.228)	1.012(0.858,1.195)	0.974(0.816,1.162)	0.953(0.794,1.144)	0.962(0.806,1.148)	1.007(0.846,1.197)
	Young	0.993(0.913,1.081)	1.020(0.894,1.163)	1.077(0.925,1.253)	1.158(0.985,1.362)	1.252(1.054,1.488)	1.341(1.123,1.603)	1.410(1.188,1.675)	1.449(1.225,1.715)
	Old	1.093(1.004,1.190)	1.126(0.987,1.285)	1.101(0.945,1.283)	1.039(0.881,1.225)	0.969(0.812,1.156)	0.915(0.762,1.098)	0.888(0.744,1.060)	0.894(0.751,1.064)
**$PM_{2.5}$**	Male	1.027(0.955,1.104)	1.051(0.936,1.180)	1.073(0.930,1.237)	1.091(0.924,1.288)	1.106(0.913,1.339)	1.116(0.902,1.381)	1.122(0.895,1.406)	1.123(0.886,1.422)
	Female	1.063(0.988,1.144)	1.109(0.986,1.247)	1.137(0.984,1.314)	1.152(0.974,1.362)	1.159(0.955,1.406)	1.166(0.940,1.446)	1.178(0.937,1.480)	1.197(0.942,1.520)
	Young	0.998(0.926,1.074)	1.016(0.903,1.144)	1.054(0.912,1.218)	1.105(0.935,1.305)	1.158(0.957,1.402)	1.202(0.973,1.485)	1.228(0.982,1.534)	1.229(0.973,1.551)
	Old	1.064(0.991,1.142)	1.096(0.977,1.228)	1.096(0.952,1.263)	1.075(0.910,1.270)	1.046(0.862,1.269)	1.021(0.823,1.268)	1.010(0.802,1.271)	1.014(0.797,1.292)
**PM_{10}**	Male	1.062(1.002,1.126)	1.148(1.045,1.261)	1.259(1.120,1.414)	1.391(1.211,1.598)	1.536(1.305,1.807)	1.678(1.399,2.014)	1.803(1.485,2.189)	1.899(1.549,2.329)
	Female	1.076(1.014,1.142)	1.158(1.052,1.274)	1.245(1.104,1.402)	1.338(1.161,1.542)	1.439(1.219,1.700)	1.551(1.287,1.869)	1.676(1.375,2.043)	1.815(1.474,2.236)
	Young	1.026(0.965,1.091)	1.092(0.989,1.205)	1.200(1.062,1.355)	1.346(1.166,1.553)	1.515(1.283,1.789)	1.684(1.399,2.026)	1.827(1.502,2.222)	1.924(1.566,2.364)
	Old	1.080(1.021,1.142)	1.152(1.052,1.261)	1.216(1.086,1.363)	1.275(1.113,1.461)	1.333(1.136,1.564)	1.396(1.166,1.671)	1.469(1.213,1.780)	1.556(1.271,1.904)
**SO_2**	Male	0.974(0.871,1.089)	1.036(0.871,1.231)	1.189(0.974,1.453)	1.445(1.168,1.788)	1.802(1.438,2.257)	2.235(1.775,2.814)	2.701(2.165,3.371)	3.149(2.529,3.922)
	Female	1.001(0.894,1.121)	1.068(0.896,1.273)	1.207(0.986,1.479)	1.426(1.149,1.770)	1.726(1.373,2.170)	2.099(1.662,2.652)	2.531(2.022,3.168)	3.005(2.407,3.752)
	Young	1.009(0.906,1.125)	1.121(0.947,1.327)	1.354(1.115,1.644)	1.735(1.411,2.134)	2.277(1.830,2.835)	2.953(2.361,3.695)	3.693(2.979,4.578)	4.401(3.559,5.443)
	Old	0.938(0.836,1.053)	0.932(0.779,1.114)	0.974(0.792,1.199)	1.063(0.851,1.327)	1.193(0.943,1.509)	1.358(1.067,1.728)	1.554(1.231,1.962)	1.779(1.411,2.243)
**NO_2**	Male	1.009(0.950,1.072)	1.051(0.954,1.157)	1.124(0.999,1.265)	1.228(1.072,1.407)	1.356(1.161,1.584)	1.497(1.261,1.777)	1.642(1.371,1.967)	1.783(1.477,2.153)
	Female	1.015(0.955,1.080)	1.054(0.956,1.163)	1.117(0.991,1.258)	1.202(1.048,1.380)	1.308(1.118,1.531)	1.431(1.203,1.702)	1.565(1.304,1.879)	1.711(1.414,2.070)
	Young	0.996(0.936,1.059)	1.032(0.935,1.139)	1.109(0.984,1.250)	1.224(1.066,1.404)	1.370(1.171,1.602)	1.536(1.292,1.825)	1.709(1.426,2.048)	1.880(1.557,2.270)
	Old	1.021(0.963,1.083)	1.062(0.967,1.167)	1.124(1.002,1.262)	1.206(1.055,1.379)	1.306(1.120,1.522)	1.418(1.196,1.680)	1.539(1.286,1.841)	1.667(1.382,2.010)
**O_3**	Male	0.818(0.772,0.867)	0.666(0.607,0.731)	0.540(0.483,0.604)	0.439(0.387,0.498)	0.360(0.312,0.415)	0.300(0.256,0.351)	0.255(0.216,0.301)	0.222(0.187,0.264)
	Female	0.860(0.810,0.913)	0.716(0.651,0.788)	0.581(0.518,0.652)	0.466(0.409,0.530)	0.375(0.324,0.434)	0.309(0.263,0.362)	0.263(0.222,0.312)	0.234(0.196,0.279)
	Young	0.828(0.780,0.880)	0.681(0.619,0.750)	0.558(0.497,0.626)	0.458(0.402,0.521)	0.378(0.327,0.438)	0.318(0.272,0.373)	0.273(0.231,0.323)	0.240(0.202,0.286)
	Old	0.875(0.826,0.926)	0.748(0.683,0.819)	0.628(0.562,0.701)	0.522(0.460,0.592)	0.436(0.378,0.503)	0.371(0.317,0.434)	0.324(0.274,0.382)	0.292(0.245,0.347)

Supplementary Table 25 Subgroup analysis by gender and age of single-day lagged relative risks (RRs) and 95% confidence intervals (CIs) for the impact of extremely high air pollution concentrations on CHD hospitalizations in Henan Province, 2016–2021(df of temperature = 2)

pollutant	group	Lag0	Lag1	Lag2	Lag3	Lag4	Lag5	Lag6	Lag7
		RR 95% CI	RR 95% CI	RR 95% CI	RR 95% CI	RR 95% CI	RR 95% CI	RR 95% CI	RR 95% CI
**CO**	Male	1.020(0.936,1.110)	1.013(0.963,1.065)	1.006(0.966,1.048)	1.000(0.951,1.051)	0.994(0.944,1.046)	0.988(0.947,1.031)	0.983(0.937,1.031)	0.978(0.905,1.057)
	Female	1.055(0.968,1.150)	1.015(0.964,1.068)	0.982(0.943,1.023)	0.963(0.915,1.013)	0.962(0.913,1.013)	0.979(0.937,1.022)	1.009(0.961,1.059)	1.047(0.968,1.132)
	Young	0.993(0.913,1.081)	1.027(0.977,1.079)	1.056(1.014,1.099)	1.076(1.024,1.130)	1.081(1.028,1.137)	1.071(1.027,1.117)	1.052(1.003,1.102)	1.028(0.952,1.110)
	Old	1.093(1.004,1.190)	1.030(0.979,1.083)	0.978(0.939,1.019)	0.944(0.897,0.993)	0.933(0.886,0.982)	0.944(0.904,0.985)	0.971(0.925,1.019)	1.006(0.931,1.088)
**$PM_{2.5}$**	Male	1.027(0.955,1.104)	1.024(0.977,1.073)	1.021(0.977,1.066)	1.017(0.967,1.070)	1.013(0.962,1.067)	1.009(0.965,1.056)	1.005(0.960,1.052)	1.001(0.936,1.069)
	Female	1.063(0.988,1.144)	1.043(0.994,1.094)	1.026(0.982,1.071)	1.013(0.962,1.066)	1.006(0.955,1.060)	1.006(0.961,1.053)	1.010(0.964,1.058)	1.016(0.950,1.087)
	Young	0.998(0.926,1.074)	1.019(0.971,1.069)	1.037(0.993,1.082)	1.048(0.996,1.103)	1.048(0.995,1.105)	1.038(0.992,1.086)	1.021(0.975,1.070)	1.001(0.935,1.071)
	Old	1.064(0.991,1.142)	1.030(0.982,1.079)	1.001(0.958,1.045)	0.981(0.932,1.032)	0.973(0.923,1.025)	0.977(0.933,1.022)	0.989(0.944,1.035)	1.005(0.941,1.073)
**PM_{10}**	Male	1.062(1.002,1.126)	1.081(1.040,1.124)	1.096(1.057,1.138)	1.105(1.058,1.155)	1.104(1.057,1.153)	1.093(1.053,1.134)	1.074(1.033,1.117)	1.053(0.994,1.117)
	Female	1.076(1.014,1.142)	1.076(1.034,1.119)	1.075(1.035,1.116)	1.075(1.028,1.124)	1.076(1.029,1.125)	1.078(1.038,1.120)	1.080(1.038,1.124)	1.083(1.020,1.150)
	Young	1.026(0.965,1.091)	1.065(1.022,1.108)	1.099(1.058,1.141)	1.121(1.073,1.173)	1.126(1.077,1.177)	1.111(1.070,1.155)	1.085(1.042,1.130)	1.053(0.992,1.119)
	Old	1.080(1.021,1.142)	1.067(1.027,1.108)	1.056(1.018,1.095)	1.048(1.004,1.094)	1.045(1.001,1.091)	1.047(1.010,1.086)	1.052(1.013,1.094)	1.059(1.000,1.121)
**SO_2**	Male	0.974(0.871,1.089)	1.063(0.996,1.135)	1.148(1.090,1.210)	1.215(1.140,1.295)	1.247(1.168,1.331)	1.240(1.174,1.311)	1.209(1.134,1.288)	1.166(1.052,1.293)
	Female	1.001(0.894,1.121)	1.067(0.998,1.141)	1.130(1.072,1.192)	1.181(1.107,1.260)	1.210(1.133,1.293)	1.216(1.150,1.286)	1.206(1.130,1.286)	1.187(1.069,1.318)
	Young	1.009(0.906,1.125)	1.111(1.042,1.184)	1.208(1.148,1.271)	1.282(1.204,1.364)	1.312(1.231,1.399)	1.297(1.229,1.369)	1.251(1.175,1.331)	1.192(1.077,1.319)
	Old	0.938(0.836,1.053)	0.993(0.927,1.063)	1.046(0.991,1.104)	1.091(1.022,1.165)	1.122(1.049,1.200)	1.139(1.075,1.206)	1.144(1.072,1.222)	1.145(1.030,1.273)
**NO_2**	Male	1.009(0.950,1.072)	1.041(1.001,1.082)	1.070(1.033,1.108)	1.092(1.048,1.139)	1.104(1.058,1.152)	1.104(1.064,1.145)	1.097(1.055,1.140)	1.086(1.026,1.149)
	Female	1.015(0.955,1.080)	1.038(0.998,1.080)	1.059(1.022,1.098)	1.077(1.032,1.123)	1.088(1.042,1.136)	1.093(1.053,1.135)	1.094(1.052,1.138)	1.093(1.032,1.158)
	Young	0.996(0.936,1.059)	1.036(0.996,1.078)	1.074(1.037,1.113)	1.104(1.058,1.151)	1.119(1.072,1.169)	1.121(1.080,1.163)	1.113(1.070,1.157)	1.100(1.038,1.165)
	Old	1.021(0.963,1.083)	1.040(1.001,1.081)	1.058(1.022,1.096)	1.073(1.030,1.118)	1.082(1.038,1.128)	1.086(1.047,1.126)	1.085(1.045,1.127)	1.083(1.025,1.145)
**O_3**	Male	0.818(0.772,0.867)	0.814(0.784,0.844)	0.811(0.786,0.837)	0.813(0.783,0.844)	0.820(0.789,0.852)	0.833(0.806,0.861)	0.851(0.822,0.880)	0.871(0.828,0.916)
	Female	0.860(0.810,0.913)	0.833(0.802,0.865)	0.812(0.786,0.839)	0.801(0.771,0.833)	0.805(0.774,0.838)	0.823(0.796,0.852)	0.853(0.823,0.884)	0.888(0.843,0.936)
	Young	0.828(0.780,0.880)	0.823(0.792,0.854)	0.819(0.793,0.846)	0.820(0.789,0.852)	0.827(0.795,0.860)	0.841(0.812,0.870)	0.859(0.829,0.890)	0.880(0.835,0.927)
	Old	0.875(0.826,0.926)	0.855(0.824,0.887)	0.839(0.813,0.866)	0.832(0.801,0.863)	0.835(0.804,0.868)	0.850(0.822,0.879)	0.873(0.844,0.904)	0.901(0.857,0.947)

Supplementary Table 26 Subgroup analysis by gender and age of cumulative lagged relative risks (RRs) and 95% confidence intervals (CIs) for the impact of extremely low air pollution concentrations on CHD hospitalizations in Henan Province, 2016–2021(df of temperature = 4)

pollutant	group	Lag00	Lag01	Lag02	Lag03	Lag04	Lag05	Lag06	Lag07
		RR 95% CI	RR 95% CI	RR 95% CI	RR 95% CI	RR 95% CI	RR 95% CI	RR 95% CI	RR 95% CI
**CO**	Male	0.995(0.977,1.014)	0.992(0.965,1.021)	0.991(0.959,1.024)	0.991(0.957,1.027)	0.993(0.957,1.031)	0.996(0.958,1.035)	1.000(0.963,1.038)	1.005(0.969,1.042)
	Female	0.989(0.970,1.007)	0.986(0.958,1.014)	0.989(0.957,1.023)	0.998(0.963,1.034)	1.006(0.968,1.045)	1.011(0.972,1.051)	1.009(0.971,1.048)	0.999(0.962,1.037)
	Young	1.001(0.983,1.020)	0.996(0.968,1.024)	0.984(0.953,1.017)	0.969(0.936,1.003)	0.953(0.918,0.989)	0.939(0.904,0.976)	0.929(0.895,0.964)	0.923(0.891,0.957)
	Old	0.981(0.963,0.999)	0.974(0.947,1.002)	0.979(0.947,1.012)	0.991(0.957,1.027)	1.007(0.969,1.045)	1.020(0.980,1.060)	1.026(0.988,1.066)	1.025(0.988,1.064)
**$PM_{2.5}$**	Male	0.995(0.983,1.007)	0.991(0.971,1.011)	0.987(0.964,1.012)	0.985(0.957,1.013)	0.982(0.951,1.015)	0.981(0.946,1.017)	0.980(0.943,1.019)	0.980(0.941,1.021)
	Female	0.990(0.977,1.002)	0.982(0.963,1.002)	0.978(0.954,1.003)	0.976(0.948,1.004)	0.975(0.943,1.008)	0.974(0.939,1.011)	0.972(0.935,1.011)	0.970(0.931,1.010)
	Young	1.000(0.988,1.013)	0.997(0.977,1.018)	0.991(0.967,1.016)	0.983(0.956,1.012)	0.975(0.944,1.008)	0.969(0.935,1.005)	0.966(0.929,1.003)	0.965(0.928,1.005)
	Old	0.989(0.977,1.001)	0.984(0.965,1.003)	0.984(0.960,1.008)	0.987(0.959,1.015)	0.992(0.960,1.025)	0.996(0.960,1.033)	0.998(0.960,1.038)	0.997(0.957,1.039)
**PM_{10}**	Male	0.983(0.968,0.999)	0.962(0.938,0.988)	0.938(0.908,0.969)	0.912(0.878,0.948)	0.887(0.848,0.929)	0.866(0.823,0.911)	0.848(0.804,0.896)	0.836(0.790,0.885)
	Female	0.980(0.963,0.996)	0.960(0.935,0.986)	0.941(0.910,0.973)	0.922(0.886,0.959)	0.903(0.862,0.946)	0.885(0.840,0.932)	0.866(0.819,0.915)	0.847(0.799,0.897)
	Young	0.993(0.976,1.010)	0.976(0.949,1.003)	0.950(0.919,0.983)	0.921(0.885,0.958)	0.891(0.850,0.933)	0.865(0.821,0.911)	0.845(0.800,0.893)	0.833(0.787,0.882)
	Old	0.979(0.964,0.994)	0.961(0.937,0.986)	0.947(0.917,0.977)	0.935(0.900,0.971)	0.923(0.883,0.965)	0.911(0.867,0.958)	0.898(0.852,0.948)	0.884(0.835,0.935)
**SO_2**	Male	1.004(0.988,1.019)	0.995(0.972,1.019)	0.976(0.950,1.004)	0.950(0.923,0.979)	0.922(0.893,0.951)	0.895(0.866,0.924)	0.871(0.845,0.899)	0.853(0.827,0.879)
	Female	1.000(0.984,1.016)	0.991(0.967,1.016)	0.974(0.947,1.002)	0.952(0.924,0.981)	0.927(0.898,0.957)	0.902(0.873,0.932)	0.879(0.852,0.907)	0.858(0.832,0.885)
	Young	0.999(0.984,1.014)	0.984(0.961,1.008)	0.959(0.933,0.985)	0.926(0.900,0.953)	0.892(0.865,0.919)	0.860(0.834,0.887)	0.834(0.809,0.859)	0.814(0.790,0.838)
	Old	1.009(0.993,1.025)	1.010(0.985,1.035)	1.004(0.975,1.033)	0.992(0.962,1.023)	0.976(0.945,1.009)	0.959(0.927,0.991)	0.941(0.911,0.972)	0.923(0.894,0.954)
**NO_2**	Male	0.991(0.966,1.016)	0.991(0.966,1.016)	0.991(0.966,1.016)	0.991(0.966,1.016)	0.991(0.966,1.016)	0.991(0.966,1.016)	0.991(0.966,1.016)	0.991(0.966,1.016)
	Female	0.974(0.935,1.014)	0.974(0.935,1.014)	0.974(0.935,1.014)	0.974(0.935,1.014)	0.974(0.935,1.014)	0.974(0.935,1.014)	0.974(0.935,1.014)	0.974(0.935,1.014)
	Young	0.951(0.905,0.999)	0.951(0.905,0.999)	0.951(0.905,0.999)	0.951(0.905,0.999)	0.951(0.905,0.999)	0.951(0.905,0.999)	0.951(0.905,0.999)	0.951(0.905,0.999)
	Old	0.923(0.871,0.977)	0.923(0.871,0.977)	0.923(0.871,0.977)	0.923(0.871,0.977)	0.923(0.871,0.977)	0.923(0.871,0.977)	0.923(0.871,0.977)	0.923(0.871,0.977)
**O_3**	Male	1.140(1.098,1.184)	1.303(1.227,1.384)	1.493(1.388,1.605)	1.707(1.573,1.853)	1.941(1.770,2.128)	2.183(1.973,2.416)	2.424(2.179,2.696)	2.651(2.371,2.963)
	Female	1.104(1.062,1.147)	1.243(1.169,1.322)	1.422(1.320,1.532)	1.641(1.509,1.785)	1.888(1.718,2.075)	2.140(1.929,2.374)	2.372(2.127,2.645)	2.561(2.285,2.870)
	Young	1.130(1.086,1.175)	1.282(1.205,1.364)	1.459(1.354,1.571)	1.658(1.525,1.803)	1.875(1.707,2.060)	2.098(1.894,2.324)	2.315(2.079,2.577)	2.515(2.248,2.813)
	Old	1.092(1.052,1.133)	1.210(1.140,1.283)	1.355(1.262,1.456)	1.527(1.407,1.657)	1.715(1.564,1.881)	1.905(1.720,2.109)	2.079(1.867,2.315)	2.224(1.987,2.490)

Supplementary Table 27 Subgroup analysis by gender and age of single-day lagged relative risks (RRs) and 95% confidence intervals (CIs) for the impact of extremely low air pollution concentrations on CHD hospitalizations in Henan Province, 2016–2021(df of temperature = 4)

pollutant	group	Lag0	Lag1	Lag2	Lag3	Lag4	Lag5	Lag6	Lag7
		RR 95% CI	RR 95% CI	RR 95% CI	RR 95% CI	RR 95% CI	RR 95% CI	RR 95% CI	RR 95% CI
**CO**	Male	0.995(0.977,1.014)	0.997(0.986,1.008)	0.999(0.990,1.007)	1.000(0.990,1.011)	1.002(0.991,1.013)	1.003(0.994,1.012)	1.004(0.994,1.014)	1.005(0.988,1.022)
	Female	0.989(0.970,1.007)	0.997(0.986,1.008)	1.004(0.995,1.013)	1.008(0.997,1.019)	1.008(0.997,1.020)	1.005(0.995,1.014)	0.998(0.988,1.008)	0.990(0.974,1.007)
	Young	1.001(0.983,1.020)	0.994(0.984,1.005)	0.988(0.980,0.997)	0.984(0.974,0.995)	0.983(0.973,0.994)	0.985(0.976,0.994)	0.989(0.979,0.999)	0.994(0.978,1.011)
	Old	0.981(0.963,0.999)	0.994(0.983,1.004)	1.005(0.996,1.014)	1.013(1.002,1.024)	1.015(1.004,1.027)	1.013(1.004,1.022)	1.007(0.996,1.017)	0.999(0.982,1.016)
**$PM_{2.5}$**	Male	0.995(0.983,1.007)	0.996(0.988,1.004)	0.996(0.989,1.004)	0.997(0.989,1.006)	0.998(0.989,1.007)	0.999(0.991,1.006)	0.999(0.991,1.007)	1.000(0.989,1.011)
	Female	0.990(0.977,1.002)	0.993(0.985,1.001)	0.996(0.988,1.003)	0.998(0.989,1.007)	0.999(0.990,1.008)	0.999(0.991,1.007)	0.998(0.990,1.006)	0.997(0.986,1.009)
	Young	1.000(0.988,1.013)	0.997(0.989,1.005)	0.994(0.987,1.001)	0.992(0.983,1.001)	0.992(0.983,1.001)	0.994(0.986,1.001)	0.996(0.989,1.004)	1.000(0.988,1.011)
	Old	0.989(0.977,1.001)	0.995(0.987,1.003)	1.000(0.992,1.007)	1.003(0.995,1.012)	1.005(0.996,1.014)	1.004(0.996,1.012)	1.002(0.994,1.010)	0.999(0.988,1.010)
**PM_{10}**	Male	0.983(0.968,0.999)	0.979(0.968,0.989)	0.975(0.965,0.985)	0.973(0.961,0.984)	0.973(0.961,0.985)	0.976(0.966,0.986)	0.980(0.969,0.991)	0.985(0.970,1.002)
	Female	0.980(0.963,0.996)	0.980(0.969,0.991)	0.980(0.970,0.990)	0.980(0.968,0.992)	0.980(0.968,0.992)	0.979(0.969,0.990)	0.979(0.968,0.990)	0.978(0.962,0.994)
	Young	0.993(0.976,1.010)	0.983(0.972,0.994)	0.974(0.964,0.984)	0.969(0.957,0.981)	0.968(0.956,0.980)	0.971(0.961,0.981)	0.977(0.967,0.989)	0.986(0.969,1.002)
	Old	0.979(0.964,0.994)	0.982(0.972,0.993)	0.985(0.975,0.995)	0.987(0.975,0.999)	0.988(0.976,1.000)	0.987(0.977,0.997)	0.986(0.975,0.996)	0.984(0.968,1.000)
**SO_2**	Male	1.004(0.988,1.019)	0.992(0.983,1.001)	0.981(0.974,0.988)	0.973(0.965,0.982)	0.970(0.961,0.979)	0.971(0.963,0.978)	0.974(0.965,0.983)	0.979(0.965,0.993)
	Female	1.000(0.984,1.016)	0.991(0.982,1.000)	0.983(0.976,0.990)	0.977(0.968,0.986)	0.974(0.965,0.983)	0.973(0.966,0.981)	0.974(0.966,0.983)	0.976(0.962,0.991)
	Young	0.999(0.984,1.014)	0.986(0.977,0.994)	0.974(0.967,0.981)	0.966(0.958,0.975)	0.963(0.954,0.972)	0.965(0.957,0.972)	0.969(0.961,0.978)	0.976(0.962,0.990)
	Old	1.009(0.993,1.025)	1.001(0.992,1.011)	0.994(0.986,1.001)	0.988(0.979,0.997)	0.984(0.975,0.993)	0.982(0.974,0.990)	0.981(0.973,0.990)	0.981(0.967,0.996)
**NO_2**	Male	0.995(0.970,1.022)	0.983(0.966,0.999)	0.972(0.957,0.986)	0.963(0.946,0.981)	0.959(0.942,0.977)	0.959(0.944,0.974)	0.962(0.946,0.978)	0.966(0.942,0.989)
	Female	0.994(0.968,1.020)	0.984(0.968,1.001)	0.976(0.961,0.991)	0.969(0.951,0.986)	0.964(0.947,0.982)	0.962(0.947,0.978)	0.962(0.946,0.978)	0.963(0.939,0.987)
	Young	1.002(0.976,1.029)	0.985(0.968,1.002)	0.970(0.955,0.985)	0.959(0.941,0.976)	0.953(0.935,0.970)	0.952(0.937,0.967)	0.955(0.939,0.971)	0.960(0.936,0.984)
	Old	0.991(0.966,1.016)	0.983(0.967,0.999)	0.976(0.962,0.991)	0.971(0.954,0.988)	0.967(0.950,0.985)	0.966(0.951,0.981)	0.966(0.950,0.982)	0.967(0.944,0.990)
**O_3**	Male	1.140(1.098,1.184)	1.143(1.116,1.171)	1.145(1.122,1.169)	1.144(1.116,1.172)	1.137(1.109,1.166)	1.125(1.101,1.150)	1.110(1.086,1.135)	1.094(1.059,1.130)
	Female	1.104(1.062,1.147)	1.126(1.099,1.154)	1.144(1.120,1.169)	1.154(1.125,1.183)	1.150(1.121,1.180)	1.134(1.109,1.159)	1.108(1.083,1.134)	1.080(1.044,1.117)
	Young	1.130(1.086,1.175)	1.135(1.107,1.163)	1.138(1.114,1.162)	1.137(1.109,1.166)	1.131(1.102,1.160)	1.119(1.094,1.144)	1.103(1.078,1.129)	1.086(1.050,1.124)
	Old	1.092(1.052,1.133)	1.108(1.082,1.134)	1.120(1.098,1.144)	1.127(1.100,1.154)	1.123(1.096,1.152)	1.110(1.087,1.135)	1.092(1.068,1.116)	1.070(1.036,1.105)

Supplementary Table 28 Subgroup analysis by gender and age of cumulative lagged relative risks (RRs) and 95% confidence intervals (CIs) for the impact of extremely high air pollution concentrations on CHD hospitalizations in Henan Province, 2016–2021(df of temperature = 4)

pollutant	group	Lag00	Lag01	Lag02	Lag03	Lag04	Lag05	Lag06	Lag07
		RR 95% CI	RR 95% CI	RR 95% CI	RR 95% CI	RR 95% CI	RR 95% CI	RR 95% CI	RR 95% CI
**CO**	Male	1.022(0.939,1.113)	1.037(0.908,1.183)	1.043(0.895,1.215)	1.041(0.884,1.227)	1.033(0.867,1.230)	1.019(0.851,1.221)	1.001(0.841,1.191)	0.978(0.824,1.160)
	Female	1.055(0.967,1.150)	1.070(0.936,1.224)	1.051(0.900,1.227)	1.012(0.857,1.194)	0.973(0.815,1.161)	0.952(0.793,1.144)	0.961(0.805,1.147)	1.006(0.846,1.196)
	Young	0.993(0.913,1.081)	1.020(0.894,1.163)	1.076(0.925,1.253)	1.158(0.985,1.362)	1.252(1.053,1.488)	1.341(1.123,1.603)	1.411(1.188,1.675)	1.450(1.225,1.716)
	Old	1.096(1.006,1.193)	1.130(0.990,1.290)	1.105(0.948,1.287)	1.041(0.883,1.228)	0.969(0.813,1.157)	0.913(0.761,1.097)	0.885(0.742,1.057)	0.890(0.748,1.060)
**$PM_{2.5}$**	Male	1.030(0.958,1.107)	1.056(0.940,1.186)	1.078(0.934,1.243)	1.096(0.928,1.294)	1.109(0.916,1.343)	1.118(0.904,1.384)	1.123(0.896,1.408)	1.124(0.887,1.424)
	Female	1.064(0.988,1.144)	1.110(0.986,1.248)	1.138(0.985,1.315)	1.152(0.974,1.363)	1.159(0.955,1.407)	1.166(0.940,1.446)	1.178(0.937,1.480)	1.197(0.942,1.520)
	Young	0.998(0.926,1.074)	1.016(0.903,1.144)	1.054(0.912,1.218)	1.105(0.935,1.305)	1.158(0.957,1.402)	1.202(0.973,1.485)	1.228(0.982,1.535)	1.229(0.973,1.551)
	Old	1.067(0.994,1.145)	1.100(0.981,1.234)	1.102(0.956,1.270)	1.080(0.914,1.276)	1.049(0.865,1.273)	1.024(0.825,1.271)	1.011(0.803,1.273)	1.016(0.798,1.294)
**PM_{10}**	Male	1.062(1.002,1.125)	1.148(1.045,1.260)	1.258(1.119,1.414)	1.390(1.210,1.597)	1.535(1.305,1.806)	1.677(1.398,2.013)	1.803(1.485,2.188)	1.900(1.549,2.330)
	Female	1.077(1.015,1.143)	1.158(1.052,1.275)	1.245(1.105,1.403)	1.338(1.161,1.542)	1.440(1.219,1.700)	1.551(1.288,1.870)	1.676(1.375,2.044)	1.816(1.474,2.237)
	Young	1.026(0.964,1.091)	1.092(0.989,1.205)	1.200(1.062,1.355)	1.346(1.166,1.553)	1.515(1.283,1.789)	1.684(1.399,2.026)	1.827(1.502,2.222)	1.924(1.566,2.364)
	Old	1.080(1.021,1.142)	1.152(1.052,1.261)	1.216(1.086,1.363)	1.275(1.113,1.460)	1.332(1.135,1.564)	1.396(1.166,1.671)	1.469(1.213,1.780)	1.557(1.273,1.906)
**SO_2**	Male	0.974(0.871,1.089)	1.035(0.871,1.231)	1.189(0.973,1.452)	1.444(1.167,1.787)	1.799(1.436,2.254)	2.230(1.771,2.808)	2.694(2.159,3.362)	3.139(2.521,3.910)
	Female	1.000(0.893,1.120)	1.066(0.895,1.271)	1.205(0.984,1.477)	1.424(1.147,1.768)	1.725(1.373,2.168)	2.099(1.662,2.652)	2.532(2.023,3.169)	3.007(2.408,3.755)
	Young	1.009(0.905,1.125)	1.121(0.947,1.327)	1.354(1.115,1.644)	1.736(1.412,2.135)	2.279(1.831,2.836)	2.955(2.362,3.698)	3.697(2.982,4.583)	4.406(3.562,5.449)
	Old	0.938(0.836,1.052)	0.931(0.778,1.113)	0.973(0.790,1.197)	1.061(0.850,1.324)	1.190(0.941,1.506)	1.355(1.064,1.724)	1.550(1.227,1.957)	1.774(1.407,2.237)
**NO_2**	Male	1.011(0.951,1.074)	1.053(0.956,1.159)	1.126(1.001,1.267)	1.229(1.073,1.408)	1.355(1.160,1.583)	1.495(1.259,1.774)	1.638(1.367,1.962)	1.777(1.472,2.146)
	Female	1.015(0.954,1.079)	1.053(0.955,1.162)	1.116(0.990,1.258)	1.202(1.047,1.379)	1.308(1.118,1.531)	1.431(1.203,1.702)	1.565(1.304,1.879)	1.710(1.414,2.069)
	Young	0.996(0.936,1.059)	1.032(0.935,1.139)	1.108(0.983,1.250)	1.224(1.066,1.404)	1.370(1.171,1.603)	1.536(1.293,1.826)	1.710(1.427,2.049)	1.881(1.558,2.272)
	Old	1.022(0.964,1.084)	1.064(0.968,1.169)	1.126(1.003,1.263)	1.207(1.056,1.380)	1.305(1.119,1.521)	1.415(1.194,1.678)	1.535(1.283,1.836)	1.661(1.377,2.004)
**O_3**	Male	0.817(0.770,0.866)	0.664(0.605,0.728)	0.538(0.481,0.602)	0.437(0.385,0.497)	0.359(0.311,0.414)	0.299(0.256,0.350)	0.254(0.216,0.300)	0.221(0.186,0.263)
	Female	0.858(0.808,0.912)	0.714(0.649,0.786)	0.580(0.517,0.651)	0.465(0.408,0.529)	0.374(0.323,0.433)	0.308(0.263,0.362)	0.263(0.222,0.311)	0.234(0.196,0.279)
	Young	0.828(0.779,0.880)	0.681(0.619,0.750)	0.558(0.497,0.626)	0.457(0.402,0.521)	0.378(0.327,0.437)	0.318(0.271,0.373)	0.273(0.231,0.322)	0.240(0.202,0.286)
	Old	0.873(0.824,0.924)	0.745(0.680,0.816)	0.625(0.559,0.698)	0.520(0.458,0.590)	0.434(0.376,0.501)	0.369(0.315,0.432)	0.322(0.273,0.381)	0.290(0.244,0.346)

Supplementary Table 29 Subgroup analysis by gender and age of single-day lagged relative risks (RRs) and 95% confidence intervals (CIs) for the impact of extremely high air pollution concentrations on CHD hospitalizations in Henan Province, 2016–2021(df of temperature = 4)

pollutant	group	Lag0	Lag1	Lag2	Lag3	Lag4	Lag5	Lag6	Lag7
		RR 95% CI	RR 95% CI	RR 95% CI	RR 95% CI	RR 95% CI	RR 95% CI	RR 95% CI	RR 95% CI
**CO**	Male	1.022(0.939,1.113)	1.014(0.964,1.066)	1.006(0.966,1.048)	0.999(0.950,1.050)	0.992(0.943,1.044)	0.987(0.945,1.030)	0.982(0.936,1.030)	0.977(0.904,1.056)
	Female	1.055(0.967,1.150)	1.015(0.964,1.068)	0.982(0.942,1.023)	0.963(0.915,1.013)	0.962(0.913,1.013)	0.979(0.937,1.022)	1.009(0.961,1.059)	1.047(0.967,1.132)
	Young	0.993(0.913,1.081)	1.026(0.977,1.079)	1.056(1.014,1.099)	1.076(1.024,1.130)	1.081(1.028,1.137)	1.071(1.027,1.117)	1.052(1.003,1.103)	1.028(0.952,1.110)
	Old	1.096(1.006,1.193)	1.031(0.980,1.084)	0.978(0.938,1.019)	0.943(0.896,0.991)	0.931(0.884,0.980)	0.942(0.903,0.984)	0.969(0.924,1.017)	1.006(0.930,1.087)
**$PM_{2.5}$**	Male	1.030(0.958,1.107)	1.025(0.978,1.075)	1.021(0.978,1.066)	1.017(0.966,1.070)	1.012(0.961,1.066)	1.008(0.964,1.055)	1.004(0.959,1.052)	1.000(0.936,1.069)
	Female	1.064(0.988,1.144)	1.043(0.995,1.094)	1.026(0.982,1.071)	1.013(0.962,1.066)	1.006(0.954,1.060)	1.006(0.961,1.053)	1.010(0.964,1.058)	1.016(0.950,1.087)
	Young	0.998(0.926,1.074)	1.019(0.971,1.069)	1.037(0.993,1.082)	1.048(0.996,1.103)	1.048(0.995,1.105)	1.038(0.992,1.086)	1.021(0.975,1.070)	1.001(0.935,1.071)
	Old	1.067(0.994,1.145)	1.031(0.984,1.081)	1.001(0.958,1.046)	0.980(0.931,1.032)	0.972(0.922,1.024)	0.976(0.932,1.021)	0.988(0.943,1.034)	1.005(0.941,1.073)
**PM_{10}**	Male	1.062(1.002,1.125)	1.081(1.040,1.123)	1.096(1.056,1.138)	1.105(1.058,1.154)	1.104(1.057,1.153)	1.093(1.053,1.134)	1.075(1.034,1.118)	1.054(0.994,1.117)
	Female	1.077(1.015,1.143)	1.076(1.034,1.119)	1.075(1.035,1.116)	1.075(1.028,1.124)	1.076(1.029,1.125)	1.078(1.037,1.120)	1.080(1.038,1.124)	1.083(1.021,1.150)
	Young	1.026(0.964,1.091)	1.065(1.022,1.108)	1.099(1.058,1.141)	1.121(1.073,1.173)	1.126(1.077,1.177)	1.111(1.070,1.155)	1.085(1.042,1.130)	1.053(0.992,1.119)
	Old	1.080(1.021,1.142)	1.067(1.027,1.108)	1.056(1.018,1.095)	1.048(1.004,1.094)	1.045(1.001,1.091)	1.047(1.010,1.086)	1.053(1.013,1.094)	1.060(1.001,1.122)
**SO_2**	Male	0.974(0.871,1.089)	1.063(0.995,1.135)	1.148(1.090,1.209)	1.214(1.140,1.294)	1.246(1.167,1.330)	1.240(1.173,1.310)	1.208(1.134,1.288)	1.165(1.051,1.292)
	Female	1.000(0.893,1.120)	1.067(0.998,1.140)	1.130(1.072,1.192)	1.182(1.108,1.261)	1.211(1.133,1.294)	1.217(1.151,1.287)	1.206(1.131,1.287)	1.188(1.069,1.319)
	Young	1.009(0.905,1.125)	1.111(1.042,1.184)	1.208(1.148,1.271)	1.282(1.204,1.365)	1.313(1.231,1.399)	1.297(1.229,1.369)	1.251(1.175,1.331)	1.192(1.077,1.319)
	Old	0.938(0.836,1.052)	0.992(0.927,1.063)	1.045(0.990,1.103)	1.090(1.021,1.164)	1.122(1.049,1.200)	1.138(1.075,1.205)	1.144(1.071,1.222)	1.144(1.030,1.272)
**NO_2**	Male	1.011(0.951,1.074)	1.042(1.002,1.083)	1.070(1.033,1.108)	1.091(1.047,1.138)	1.103(1.057,1.150)	1.103(1.063,1.144)	1.096(1.054,1.139)	1.085(1.025,1.148)
	Female	1.015(0.954,1.079)	1.038(0.998,1.080)	1.059(1.022,1.098)	1.077(1.032,1.123)	1.088(1.043,1.136)	1.093(1.053,1.135)	1.094(1.052,1.138)	1.093(1.032,1.157)
	Young	0.996(0.936,1.059)	1.036(0.996,1.078)	1.074(1.037,1.113)	1.104(1.058,1.151)	1.120(1.073,1.169)	1.121(1.080,1.164)	1.113(1.071,1.157)	1.100(1.039,1.165)
	Old	1.022(0.964,1.084)	1.041(1.002,1.081)	1.058(1.022,1.096)	1.072(1.029,1.117)	1.081(1.037,1.127)	1.085(1.046,1.125)	1.084(1.044,1.126)	1.082(1.024,1.144)
**O_3**	Male	0.817(0.770,0.866)	0.813(0.783,0.843)	0.811(0.785,0.837)	0.813(0.783,0.844)	0.820(0.789,0.852)	0.833(0.806,0.862)	0.851(0.822,0.881)	0.871(0.828,0.916)
	Female	0.858(0.808,0.912)	0.832(0.801,0.864)	0.812(0.786,0.839)	0.801(0.771,0.833)	0.805(0.774,0.838)	0.824(0.796,0.853)	0.853(0.823,0.884)	0.888(0.843,0.936)
	Young	0.828(0.779,0.880)	0.822(0.792,0.854)	0.819(0.793,0.846)	0.820(0.789,0.852)	0.827(0.795,0.860)	0.841(0.812,0.870)	0.859(0.829,0.890)	0.880(0.835,0.927)
	Old	0.873(0.824,0.924)	0.854(0.823,0.885)	0.839(0.813,0.866)	0.832(0.801,0.863)	0.835(0.804,0.868)	0.850(0.822,0.879)	0.873(0.844,0.904)	0.901(0.857,0.947)

Supplementary Table 30 Subgroup analysis by gender and age of cumulative lagged relative risks (RRs) and 95% confidence intervals (CIs) for the impact of extremely low air pollution concentrations on CHD hospitalizations in Henan Province, 2016–2021(df of relative humidity = 2)

pollutant	group	Lag00	Lag01	Lag02	Lag03	Lag04	Lag05	Lag06	Lag07
		RR 95% CI	RR 95% CI	RR 95% CI	RR 95% CI	RR 95% CI	RR 95% CI	RR 95% CI	RR 95% CI
**CO**	Male	0.996(0.978,1.014)	0.993(0.965,1.022)	0.992(0.960,1.025)	0.993(0.959,1.028)	0.995(0.958,1.033)	0.998(0.960,1.037)	1.001(0.965,1.039)	1.006(0.970,1.043)
	Female	0.989(0.971,1.007)	0.986(0.958,1.015)	0.990(0.958,1.023)	0.998(0.964,1.035)	1.007(0.970,1.046)	1.012(0.973,1.052)	1.010(0.973,1.049)	1.000(0.963,1.038)
	Young	1.002(0.984,1.020)	0.996(0.969,1.025)	0.985(0.954,1.018)	0.970(0.937,1.004)	0.954(0.920,0.990)	0.940(0.905,0.977)	0.930(0.897,0.965)	0.924(0.892,0.958)
	Old	0.981(0.963,0.999)	0.975(0.948,1.003)	0.980(0.948,1.013)	0.993(0.958,1.028)	1.008(0.971,1.047)	1.021(0.982,1.062)	1.028(0.990,1.068)	1.026(0.989,1.065)
**$PM_{2.5}$**	Male	0.995(0.983,1.007)	0.991(0.971,1.011)	0.988(0.964,1.012)	0.985(0.958,1.013)	0.983(0.952,1.016)	0.982(0.947,1.018)	0.981(0.944,1.020)	0.981(0.942,1.021)
	Female	0.989(0.977,1.002)	0.982(0.963,1.002)	0.978(0.954,1.003)	0.976(0.949,1.005)	0.976(0.944,1.008)	0.975(0.940,1.011)	0.973(0.936,1.012)	0.970(0.931,1.011)
	Young	1.000(0.988,1.013)	0.997(0.977,1.018)	0.991(0.967,1.016)	0.984(0.956,1.012)	0.976(0.945,1.008)	0.970(0.935,1.005)	0.966(0.930,1.004)	0.966(0.928,1.005)
	Old	0.989(0.977,1.001)	0.984(0.965,1.003)	0.984(0.960,1.008)	0.987(0.960,1.016)	0.992(0.960,1.026)	0.997(0.961,1.034)	0.999(0.960,1.039)	0.998(0.957,1.040)
**PM_{10}**	Male	0.984(0.968,1.000)	0.963(0.938,0.988)	0.939(0.909,0.970)	0.913(0.879,0.949)	0.889(0.849,0.930)	0.867(0.824,0.912)	0.850(0.805,0.897)	0.837(0.791,0.886)
	Female	0.980(0.964,0.996)	0.960(0.935,0.986)	0.942(0.911,0.973)	0.923(0.887,0.960)	0.905(0.864,0.947)	0.886(0.841,0.933)	0.867(0.820,0.916)	0.847(0.800,0.898)
	Young	0.993(0.976,1.010)	0.976(0.950,1.003)	0.951(0.919,0.984)	0.921(0.885,0.959)	0.892(0.851,0.934)	0.866(0.822,0.912)	0.846(0.801,0.894)	0.834(0.787,0.883)
	Old	0.979(0.964,0.994)	0.962(0.938,0.986)	0.948(0.918,0.978)	0.936(0.901,0.972)	0.924(0.884,0.966)	0.912(0.868,0.959)	0.899(0.853,0.949)	0.885(0.836,0.936)
**SO_2**	Male	1.005(0.989,1.020)	0.997(0.973,1.021)	0.978(0.951,1.006)	0.952(0.924,0.981)	0.923(0.895,0.952)	0.896(0.868,0.925)	0.872(0.846,0.900)	0.854(0.828,0.880)
	Female	1.001(0.985,1.017)	0.992(0.969,1.017)	0.976(0.949,1.004)	0.953(0.925,0.983)	0.928(0.899,0.958)	0.903(0.874,0.933)	0.880(0.853,0.908)	0.859(0.833,0.886)
	Young	0.999(0.985,1.015)	0.985(0.963,1.009)	0.960(0.935,0.986)	0.927(0.901,0.954)	0.893(0.866,0.920)	0.861(0.835,0.888)	0.835(0.810,0.860)	0.814(0.791,0.839)
	Old	1.010(0.994,1.026)	1.012(0.987,1.037)	1.006(0.977,1.035)	0.994(0.964,1.025)	0.978(0.946,1.010)	0.960(0.928,0.993)	0.942(0.912,0.973)	0.924(0.895,0.955)
**NO_2**	Male	0.997(0.972,1.023)	0.981(0.941,1.022)	0.953(0.906,1.002)	0.918(0.866,0.973)	0.880(0.823,0.940)	0.843(0.783,0.907)	0.810(0.750,0.875)	0.782(0.721,0.848)
	Female	0.995(0.969,1.021)	0.980(0.940,1.022)	0.956(0.909,1.006)	0.926(0.873,0.982)	0.893(0.835,0.955)	0.859(0.797,0.925)	0.826(0.764,0.893)	0.795(0.732,0.862)
	Young	1.003(0.977,1.030)	0.988(0.948,1.031)	0.959(0.911,1.009)	0.919(0.866,0.974)	0.875(0.818,0.936)	0.833(0.773,0.897)	0.795(0.736,0.859)	0.763(0.703,0.827)
	Old	0.992(0.968,1.017)	0.976(0.938,1.016)	0.953(0.907,1.001)	0.925(0.873,0.979)	0.894(0.837,0.955)	0.863(0.802,0.928)	0.833(0.771,0.899)	0.805(0.742,0.872)
**O_3**	Male	1.140(1.097,1.183)	1.303(1.227,1.384)	1.492(1.388,1.604)	1.707(1.573,1.853)	1.941(1.770,2.129)	2.184(1.974,2.416)	2.423(2.179,2.696)	2.649(2.370,2.961)
	Female	1.103(1.061,1.147)	1.242(1.168,1.321)	1.422(1.320,1.531)	1.641(1.509,1.785)	1.889(1.718,2.076)	2.141(1.930,2.375)	2.372(2.128,2.645)	2.559(2.283,2.867)
	Young	1.130(1.086,1.175)	1.282(1.205,1.364)	1.459(1.354,1.572)	1.659(1.526,1.804)	1.876(1.708,2.060)	2.099(1.894,2.325)	2.315(2.079,2.577)	2.513(2.247,2.812)
	Old	1.091(1.052,1.132)	1.209(1.140,1.282)	1.355(1.261,1.455)	1.527(1.407,1.656)	1.715(1.564,1.881)	1.905(1.721,2.109)	2.079(1.867,2.315)	2.222(1.985,2.488)

Supplementary Table 31 Subgroup analysis by gender and age of single-day lagged relative risks (RRs) and 95% confidence intervals (CIs) for the impact of extremely low air pollution concentrations on CHD hospitalizations in Henan Province, 2016–2021(df of relative humidity = 2)

pollutant	group	Lag0	Lag1	Lag2	Lag3	Lag4	Lag5	Lag6	Lag7
		RR 95% CI	RR 95% CI	RR 95% CI	RR 95% CI	RR 95% CI	RR 95% CI	RR 95% CI	RR 95% CI
**CO**	Male	0.996(0.978,1.014)	0.997(0.987,1.008)	0.999(0.990,1.008)	1.001(0.990,1.011)	1.002(0.991,1.013)	1.003(0.994,1.012)	1.004(0.994,1.014)	1.005(0.988,1.022)
	Female	0.989(0.971,1.007)	0.997(0.986,1.008)	1.004(0.995,1.013)	1.009(0.998,1.020)	1.009(0.998,1.020)	1.005(0.995,1.014)	0.998(0.988,1.008)	0.990(0.973,1.007)
	Young	1.002(0.984,1.020)	0.995(0.984,1.005)	0.989(0.980,0.997)	0.985(0.974,0.995)	0.984(0.973,0.994)	0.985(0.977,0.994)	0.989(0.979,0.999)	0.994(0.978,1.010)
	Old	0.981(0.963,0.999)	0.994(0.983,1.005)	1.005(0.996,1.014)	1.013(1.002,1.024)	1.016(1.005,1.027)	1.013(1.004,1.022)	1.007(0.996,1.017)	0.998(0.982,1.015)
**$PM_{2.5}$**	Male	0.995(0.983,1.007)	0.996(0.988,1.004)	0.997(0.989,1.004)	0.997(0.989,1.006)	0.998(0.989,1.007)	0.999(0.991,1.006)	0.999(0.991,1.007)	1.000(0.988,1.011)
	Female	0.989(0.977,1.002)	0.993(0.985,1.001)	0.996(0.988,1.003)	0.998(0.989,1.007)	0.999(0.990,1.008)	0.999(0.991,1.007)	0.998(0.990,1.006)	0.997(0.986,1.008)
	Young	1.000(0.988,1.013)	0.997(0.989,1.005)	0.994(0.987,1.001)	0.992(0.984,1.001)	0.992(0.983,1.001)	0.994(0.986,1.001)	0.996(0.988,1.004)	1.000(0.988,1.011)
	Old	0.989(0.977,1.001)	0.995(0.987,1.003)	1.000(0.993,1.007)	1.004(0.995,1.012)	1.005(0.996,1.014)	1.004(0.997,1.012)	1.002(0.994,1.010)	0.999(0.988,1.010)
**PM_{10}**	Male	0.984(0.968,1.000)	0.979(0.968,0.989)	0.975(0.965,0.985)	0.973(0.961,0.985)	0.973(0.961,0.985)	0.976(0.966,0.986)	0.980(0.969,0.991)	0.985(0.969,1.001)
	Female	0.980(0.964,0.996)	0.980(0.969,0.991)	0.980(0.970,0.991)	0.980(0.968,0.993)	0.980(0.968,0.992)	0.979(0.969,0.990)	0.979(0.968,0.989)	0.978(0.961,0.994)
	Young	0.993(0.976,1.010)	0.983(0.972,0.994)	0.974(0.964,0.985)	0.969(0.957,0.981)	0.968(0.956,0.980)	0.971(0.961,0.981)	0.977(0.966,0.988)	0.985(0.969,1.002)
	Old	0.979(0.964,0.994)	0.982(0.972,0.993)	0.985(0.975,0.995)	0.987(0.976,0.999)	0.988(0.976,1.000)	0.987(0.977,0.997)	0.986(0.975,0.996)	0.984(0.968,0.999)
**SO_2**	Male	1.005(0.989,1.020)	0.992(0.983,1.001)	0.981(0.974,0.988)	0.973(0.965,0.982)	0.970(0.961,0.979)	0.970(0.963,0.978)	0.974(0.965,0.983)	0.979(0.965,0.993)
	Female	1.001(0.985,1.017)	0.992(0.982,1.001)	0.983(0.976,0.991)	0.977(0.968,0.986)	0.974(0.965,0.983)	0.973(0.965,0.981)	0.974(0.965,0.983)	0.976(0.962,0.991)
	Young	0.999(0.985,1.015)	0.986(0.977,0.995)	0.974(0.967,0.981)	0.966(0.958,0.974)	0.963(0.954,0.971)	0.964(0.957,0.972)	0.969(0.961,0.978)	0.976(0.962,0.990)
	Old	1.010(0.994,1.026)	1.002(0.992,1.011)	0.994(0.987,1.002)	0.988(0.979,0.997)	0.984(0.975,0.993)	0.982(0.974,0.990)	0.981(0.972,0.990)	0.981(0.967,0.996)
**NO_2**	Male	0.997(0.972,1.023)	0.984(0.967,1.000)	0.972(0.957,0.987)	0.963(0.946,0.980)	0.958(0.941,0.976)	0.958(0.943,0.973)	0.961(0.945,0.977)	0.965(0.942,0.989)
	Female	0.995(0.969,1.021)	0.985(0.968,1.002)	0.976(0.961,0.991)	0.969(0.951,0.986)	0.964(0.946,0.982)	0.962(0.947,0.977)	0.962(0.946,0.978)	0.962(0.939,0.986)
	Young	1.003(0.977,1.030)	0.985(0.969,1.002)	0.970(0.955,0.985)	0.958(0.941,0.976)	0.952(0.935,0.970)	0.952(0.937,0.967)	0.955(0.939,0.971)	0.959(0.936,0.983)
	Old	0.992(0.968,1.017)	0.984(0.968,1.000)	0.976(0.962,0.991)	0.970(0.953,0.988)	0.967(0.949,0.984)	0.965(0.950,0.980)	0.965(0.950,0.981)	0.966(0.943,0.989)
**O_3**	Male	1.140(1.097,1.183)	1.143(1.116,1.171)	1.145(1.122,1.169)	1.144(1.116,1.172)	1.137(1.109,1.166)	1.125(1.101,1.150)	1.110(1.085,1.135)	1.093(1.058,1.129)
	Female	1.103(1.061,1.147)	1.126(1.099,1.154)	1.145(1.121,1.169)	1.154(1.126,1.184)	1.151(1.122,1.181)	1.134(1.109,1.159)	1.108(1.083,1.134)	1.079(1.043,1.116)
	Young	1.130(1.086,1.175)	1.135(1.107,1.163)	1.138(1.114,1.162)	1.137(1.109,1.166)	1.131(1.102,1.160)	1.119(1.094,1.144)	1.103(1.078,1.129)	1.086(1.049,1.123)
	Old	1.091(1.052,1.132)	1.108(1.082,1.134)	1.121(1.098,1.144)	1.127(1.100,1.155)	1.124(1.096,1.152)	1.111(1.087,1.135)	1.091(1.067,1.116)	1.069(1.035,1.104)

Supplementary Table 32 Subgroup analysis by gender and age of cumulative lagged relative risks (RRs) and 95% confidence intervals (CIs) for the impact of extremely high air pollution concentrations on CHD hospitalizations in Henan Province, 2016–2021(df of relative humidity = 2)

pollutant	group	Lag00	Lag01	Lag02	Lag03	Lag04	Lag05	Lag06	Lag07
		RR 95% CI	RR 95% CI	RR 95% CI	RR 95% CI	RR 95% CI	RR 95% CI	RR 95% CI	RR 95% CI
**CO**	Male	1.020(0.937,1.111)	1.033(0.905,1.179)	1.037(0.890,1.208)	1.034(0.878,1.218)	1.025(0.861,1.220)	1.011(0.845,1.211)	0.993(0.835,1.182)	0.972(0.820,1.153)
	Female	1.055(0.967,1.150)	1.069(0.935,1.222)	1.048(0.898,1.223)	1.007(0.853,1.189)	0.967(0.810,1.154)	0.946(0.788,1.135)	0.954(0.800,1.139)	1.000(0.841,1.190)
	Young	0.992(0.912,1.080)	1.017(0.892,1.160)	1.073(0.922,1.248)	1.152(0.980,1.355)	1.244(1.047,1.478)	1.332(1.115,1.591)	1.402(1.181,1.664)	1.443(1.219,1.707)
	Old	1.094(1.005,1.192)	1.126(0.987,1.286)	1.099(0.943,1.281)	1.035(0.878,1.220)	0.962(0.807,1.147)	0.906(0.755,1.087)	0.879(0.736,1.049)	0.885(0.744,1.053)
**$PM_{2.5}$**	Male	1.030(0.958,1.107)	1.055(0.940,1.185)	1.076(0.933,1.241)	1.092(0.925,1.289)	1.104(0.912,1.337)	1.113(0.899,1.377)	1.118(0.892,1.402)	1.120(0.884,1.419)
	Female	1.064(0.989,1.145)	1.110(0.987,1.249)	1.137(0.984,1.314)	1.150(0.972,1.360)	1.156(0.952,1.402)	1.162(0.937,1.441)	1.174(0.934,1.474)	1.195(0.941,1.517)
	Young	0.998(0.926,1.075)	1.016(0.903,1.144)	1.053(0.911,1.217)	1.102(0.933,1.302)	1.154(0.954,1.397)	1.198(0.970,1.480)	1.224(0.979,1.529)	1.226(0.971,1.548)
	Old	1.067(0.994,1.146)	1.100(0.981,1.234)	1.100(0.955,1.268)	1.077(0.912,1.272)	1.045(0.862,1.268)	1.019(0.821,1.265)	1.007(0.800,1.268)	1.013(0.796,1.290)
**PM_{10}**	Male	1.061(1.001,1.125)	1.146(1.043,1.258)	1.254(1.116,1.410)	1.385(1.206,1.591)	1.528(1.299,1.797)	1.669(1.391,2.002)	1.795(1.479,2.178)	1.894(1.544,2.322)
	Female	1.076(1.014,1.142)	1.156(1.050,1.272)	1.241(1.102,1.398)	1.333(1.157,1.536)	1.433(1.214,1.692)	1.544(1.282,1.861)	1.669(1.369,2.035)	1.811(1.470,2.230)
	Young	1.025(0.964,1.090)	1.091(0.988,1.204)	1.197(1.060,1.352)	1.342(1.163,1.548)	1.509(1.279,1.782)	1.677(1.394,2.018)	1.821(1.497,2.214)	1.920(1.563,2.359)
	Old	1.079(1.020,1.141)	1.150(1.050,1.259)	1.213(1.083,1.359)	1.270(1.109,1.454)	1.326(1.130,1.557)	1.389(1.160,1.663)	1.463(1.208,1.772)	1.553(1.269,1.900)
**SO_2**	Male	0.967(0.865,1.080)	1.023(0.861,1.216)	1.173(0.961,1.432)	1.425(1.152,1.763)	1.779(1.420,2.228)	2.210(1.755,2.782)	2.674(2.143,3.336)	3.118(2.504,3.882)
	Female	0.994(0.888,1.113)	1.056(0.886,1.259)	1.192(0.974,1.460)	1.409(1.135,1.749)	1.708(1.359,2.147)	2.081(1.648,2.629)	2.512(2.008,3.144)	2.985(2.391,3.726)
	Young	1.004(0.901,1.119)	1.112(0.940,1.315)	1.341(1.105,1.628)	1.720(1.399,2.115)	2.261(1.817,2.814)	2.937(2.347,3.674)	3.677(2.966,4.558)	4.383(3.544,5.419)
	Old	0.931(0.830,1.044)	0.920(0.770,1.100)	0.960(0.780,1.181)	1.047(0.839,1.306)	1.176(0.930,1.488)	1.341(1.054,1.707)	1.536(1.217,1.940)	1.760(1.396,2.219)
**NO_2**	Male	1.007(0.948,1.069)	1.046(0.950,1.152)	1.119(0.995,1.258)	1.222(1.067,1.399)	1.349(1.155,1.575)	1.490(1.255,1.769)	1.635(1.365,1.959)	1.777(1.471,2.146)
	Female	1.012(0.952,1.076)	1.048(0.951,1.156)	1.110(0.985,1.251)	1.196(1.042,1.372)	1.303(1.113,1.525)	1.426(1.199,1.697)	1.563(1.302,1.876)	1.710(1.414,2.069)
	Young	0.993(0.934,1.056)	1.028(0.932,1.134)	1.104(0.979,1.244)	1.219(1.062,1.398)	1.366(1.168,1.597)	1.533(1.290,1.822)	1.709(1.426,2.048)	1.882(1.558,2.272)
	Old	1.018(0.960,1.080)	1.058(0.963,1.161)	1.118(0.997,1.255)	1.200(1.050,1.371)	1.299(1.114,1.514)	1.411(1.190,1.672)	1.533(1.281,1.833)	1.661(1.377,2.004)
**O_3**	Male	0.817(0.771,0.866)	0.664(0.605,0.729)	0.538(0.481,0.602)	0.437(0.385,0.497)	0.359(0.311,0.414)	0.299(0.256,0.349)	0.254(0.216,0.300)	0.222(0.187,0.263)
	Female	0.859(0.809,0.912)	0.715(0.650,0.787)	0.580(0.517,0.651)	0.465(0.408,0.529)	0.374(0.323,0.433)	0.308(0.262,0.362)	0.263(0.222,0.311)	0.234(0.196,0.279)
	Young	0.828(0.780,0.880)	0.681(0.619,0.750)	0.558(0.497,0.626)	0.457(0.402,0.520)	0.378(0.327,0.437)	0.318(0.271,0.372)	0.273(0.231,0.322)	0.240(0.202,0.286)
	Old	0.873(0.825,0.925)	0.746(0.681,0.817)	0.625(0.560,0.699)	0.520(0.458,0.590)	0.434(0.376,0.501)	0.369(0.315,0.432)	0.322(0.273,0.381)	0.291(0.244,0.346)

Supplementary Table 33 Subgroup analysis by gender and age of single-day lagged relative risks (RRs) and 95% confidence intervals (CIs) for the impact of extremely high air pollution concentrations on CHD hospitalizations in Henan Province, 2016–2021(df of relative humidity = 2)

pollutant	group	Lag0	Lag1	Lag2	Lag3	Lag4	Lag5	Lag6	Lag7
		RR 95% CI	RR 95% CI	RR 95% CI	RR 95% CI	RR 95% CI	RR 95% CI	RR 95% CI	RR 95% CI
**CO**	Male	1.020(0.937,1.111)	1.012(0.963,1.064)	1.004(0.964,1.046)	0.997(0.949,1.048)	0.991(0.942,1.043)	0.986(0.945,1.029)	0.982(0.936,1.031)	0.979(0.905,1.058)
	Female	1.055(0.967,1.150)	1.014(0.963,1.066)	0.981(0.941,1.022)	0.961(0.913,1.011)	0.960(0.912,1.011)	0.978(0.937,1.021)	1.009(0.962,1.059)	1.048(0.969,1.134)
	Young	0.992(0.912,1.080)	1.025(0.975,1.078)	1.054(1.013,1.097)	1.074(1.023,1.128)	1.080(1.027,1.136)	1.071(1.027,1.117)	1.052(1.004,1.103)	1.029(0.953,1.111)
	Old	1.094(1.005,1.192)	1.029(0.979,1.082)	0.976(0.937,1.017)	0.941(0.895,0.990)	0.930(0.883,0.979)	0.942(0.902,0.983)	0.970(0.924,1.018)	1.007(0.932,1.089)
**$PM_{2.5}$**	Male	1.030(0.958,1.107)	1.025(0.977,1.074)	1.020(0.977,1.065)	1.015(0.965,1.068)	1.011(0.960,1.065)	1.008(0.963,1.054)	1.005(0.959,1.052)	1.002(0.938,1.070)
	Female	1.064(0.989,1.145)	1.043(0.994,1.094)	1.025(0.981,1.070)	1.011(0.960,1.065)	1.005(0.953,1.059)	1.005(0.960,1.052)	1.010(0.964,1.059)	1.018(0.952,1.088)
	Young	0.998(0.926,1.075)	1.018(0.971,1.068)	1.036(0.992,1.081)	1.047(0.995,1.102)	1.048(0.994,1.103)	1.038(0.992,1.086)	1.022(0.975,1.070)	1.002(0.937,1.072)
	Old	1.067(0.994,1.146)	1.031(0.984,1.081)	1.000(0.958,1.045)	0.979(0.930,1.030)	0.971(0.921,1.023)	0.975(0.932,1.020)	0.988(0.944,1.035)	1.006(0.942,1.074)
**PM_{10}**	Male	1.061(1.001,1.125)	1.080(1.039,1.122)	1.095(1.055,1.136)	1.104(1.057,1.153)	1.103(1.056,1.152)	1.092(1.053,1.134)	1.075(1.034,1.118)	1.055(0.995,1.119)
	Female	1.076(1.014,1.142)	1.075(1.033,1.118)	1.074(1.034,1.115)	1.074(1.027,1.123)	1.075(1.028,1.124)	1.078(1.037,1.119)	1.081(1.039,1.125)	1.085(1.022,1.151)
	Young	1.025(0.964,1.090)	1.064(1.022,1.108)	1.098(1.057,1.140)	1.121(1.072,1.171)	1.125(1.076,1.176)	1.111(1.070,1.154)	1.086(1.043,1.130)	1.055(0.993,1.120)
	Old	1.079(1.020,1.141)	1.066(1.026,1.107)	1.055(1.017,1.094)	1.047(1.003,1.093)	1.045(1.001,1.090)	1.047(1.010,1.086)	1.053(1.014,1.094)	1.061(1.002,1.123)
**SO_2**	Male	0.967(0.865,1.080)	1.058(0.991,1.130)	1.146(1.088,1.208)	1.215(1.140,1.295)	1.248(1.169,1.332)	1.242(1.175,1.313)	1.210(1.135,1.290)	1.166(1.052,1.293)
	Female	0.994(0.888,1.113)	1.063(0.994,1.136)	1.129(1.070,1.190)	1.182(1.108,1.261)	1.212(1.135,1.295)	1.218(1.152,1.288)	1.207(1.132,1.288)	1.188(1.070,1.319)
	Young	1.004(0.901,1.119)	1.107(1.039,1.180)	1.207(1.147,1.270)	1.283(1.205,1.365)	1.314(1.233,1.401)	1.299(1.231,1.371)	1.252(1.176,1.332)	1.192(1.077,1.319)
	Old	0.931(0.830,1.044)	0.988(0.923,1.058)	1.043(0.988,1.101)	1.091(1.021,1.165)	1.123(1.050,1.202)	1.140(1.077,1.207)	1.146(1.073,1.224)	1.146(1.031,1.273)
**NO_2**	Male	1.007(0.948,1.069)	1.039(1.000,1.081)	1.069(1.032,1.107)	1.092(1.048,1.138)	1.104(1.058,1.152)	1.105(1.065,1.146)	1.097(1.056,1.141)	1.087(1.027,1.150)
	Female	1.012(0.952,1.076)	1.036(0.996,1.078)	1.059(1.022,1.097)	1.077(1.033,1.124)	1.089(1.044,1.137)	1.095(1.055,1.136)	1.096(1.054,1.139)	1.094(1.033,1.159)
	Young	0.993(0.934,1.056)	1.035(0.995,1.077)	1.074(1.036,1.113)	1.104(1.059,1.152)	1.121(1.074,1.170)	1.123(1.082,1.165)	1.114(1.072,1.159)	1.101(1.040,1.167)
	Old	1.018(0.960,1.080)	1.039(1.000,1.079)	1.058(1.021,1.095)	1.073(1.030,1.118)	1.082(1.038,1.129)	1.086(1.048,1.126)	1.086(1.046,1.128)	1.084(1.026,1.146)
**O_3**	Male	0.817(0.771,0.866)	0.813(0.783,0.844)	0.811(0.785,0.837)	0.812(0.782,0.843)	0.820(0.789,0.852)	0.833(0.806,0.862)	0.851(0.822,0.881)	0.872(0.829,0.917)
	Female	0.859(0.809,0.912)	0.832(0.801,0.865)	0.811(0.785,0.838)	0.801(0.770,0.832)	0.805(0.773,0.837)	0.824(0.796,0.852)	0.853(0.823,0.884)	0.890(0.844,0.937)
	Young	0.828(0.780,0.880)	0.822(0.792,0.854)	0.819(0.793,0.846)	0.820(0.789,0.852)	0.827(0.795,0.860)	0.841(0.812,0.870)	0.859(0.829,0.890)	0.881(0.836,0.928)
	Old	0.873(0.825,0.925)	0.854(0.823,0.886)	0.839(0.812,0.866)	0.831(0.801,0.863)	0.835(0.804,0.868)	0.850(0.822,0.879)	0.874(0.844,0.904)	0.902(0.858,0.948)

Supplementary Table 34 Subgroup analysis by gender and age of cumulative lagged relative risks (RRs) and 95% confidence intervals (CIs) for the impact of extremely low air pollution concentrations on CHD hospitalizations in Henan Province, 2016–2021(df of relative humidity = 4)

pollutant	group	Lag00	Lag01	Lag02	Lag03	Lag04	Lag05	Lag06	Lag07
		RR 95% CI	RR 95% CI	RR 95% CI	RR 95% CI	RR 95% CI	RR 95% CI	RR 95% CI	RR 95% CI
**CO**	Male	0.995(0.977,1.014)	0.993(0.965,1.021)	0.991(0.959,1.024)	0.992(0.957,1.027)	0.993(0.957,1.031)	0.996(0.958,1.035)	1.000(0.963,1.038)	1.005(0.969,1.042)
	Female	0.989(0.970,1.007)	0.986(0.958,1.014)	0.989(0.957,1.023)	0.998(0.963,1.034)	1.006(0.968,1.045)	1.011(0.972,1.051)	1.009(0.971,1.048)	0.999(0.963,1.037)
	Young	1.002(0.984,1.020)	0.996(0.968,1.024)	0.985(0.953,1.017)	0.969(0.936,1.004)	0.953(0.919,0.989)	0.939(0.904,0.976)	0.929(0.896,0.964)	0.924(0.891,0.958)
	Old	0.981(0.963,0.999)	0.974(0.947,1.002)	0.979(0.947,1.012)	0.991(0.957,1.027)	1.007(0.969,1.046)	1.020(0.980,1.060)	1.026(0.988,1.066)	1.025(0.988,1.064)
**$PM_{2.5}$**	Male	0.995(0.983,1.007)	0.991(0.971,1.011)	0.987(0.964,1.012)	0.985(0.957,1.013)	0.983(0.951,1.015)	0.981(0.946,1.018)	0.981(0.943,1.019)	0.981(0.942,1.021)
	Female	0.990(0.977,1.002)	0.982(0.963,1.002)	0.978(0.954,1.003)	0.976(0.949,1.005)	0.975(0.944,1.008)	0.974(0.939,1.011)	0.973(0.936,1.011)	0.970(0.931,1.011)
	Young	1.000(0.988,1.013)	0.997(0.977,1.018)	0.991(0.967,1.016)	0.983(0.956,1.012)	0.976(0.944,1.008)	0.969(0.935,1.005)	0.966(0.930,1.003)	0.966(0.928,1.005)
	Old	0.989(0.977,1.001)	0.984(0.965,1.003)	0.984(0.960,1.008)	0.987(0.959,1.016)	0.992(0.960,1.025)	0.996(0.960,1.034)	0.998(0.960,1.038)	0.997(0.957,1.039)
**PM_{10}**	Male	0.983(0.968,0.999)	0.962(0.938,0.988)	0.938(0.908,0.969)	0.912(0.878,0.948)	0.888(0.848,0.929)	0.866(0.823,0.911)	0.849(0.804,0.896)	0.836(0.790,0.885)
	Female	0.980(0.964,0.996)	0.960(0.935,0.986)	0.941(0.910,0.973)	0.923(0.887,0.960)	0.904(0.863,0.947)	0.885(0.840,0.932)	0.866(0.820,0.916)	0.847(0.799,0.898)
	Young	0.993(0.976,1.010)	0.976(0.950,1.003)	0.951(0.919,0.984)	0.921(0.885,0.959)	0.891(0.851,0.933)	0.865(0.822,0.911)	0.846(0.801,0.893)	0.834(0.787,0.883)
	Old	0.979(0.964,0.994)	0.961(0.937,0.986)	0.947(0.917,0.977)	0.935(0.900,0.971)	0.923(0.883,0.965)	0.911(0.867,0.958)	0.898(0.852,0.948)	0.884(0.836,0.935)
**SO_2**	Male	1.004(0.988,1.019)	0.995(0.972,1.020)	0.976(0.950,1.004)	0.950(0.923,0.979)	0.922(0.893,0.951)	0.895(0.866,0.924)	0.871(0.845,0.899)	0.853(0.827,0.879)
	Female	1.000(0.984,1.016)	0.991(0.967,1.016)	0.974(0.947,1.002)	0.952(0.924,0.981)	0.927(0.898,0.957)	0.902(0.873,0.932)	0.879(0.852,0.907)	0.858(0.832,0.885)
	Young	0.999(0.984,1.014)	0.984(0.962,1.008)	0.959(0.933,0.985)	0.926(0.900,0.953)	0.892(0.865,0.919)	0.860(0.834,0.887)	0.834(0.809,0.859)	0.814(0.790,0.838)
	Old	1.009(0.993,1.025)	1.010(0.985,1.035)	1.004(0.975,1.033)	0.992(0.962,1.023)	0.976(0.945,1.008)	0.959(0.927,0.991)	0.941(0.911,0.972)	0.923(0.894,0.954)
**NO_2**	Male	0.996(0.970,1.022)	0.979(0.939,1.020)	0.951(0.904,1.000)	0.916(0.864,0.971)	0.878(0.822,0.939)	0.842(0.782,0.906)	0.810(0.749,0.875)	0.782(0.721,0.848)
	Female	0.994(0.968,1.020)	0.978(0.938,1.020)	0.954(0.907,1.004)	0.925(0.872,0.981)	0.892(0.834,0.954)	0.858(0.797,0.925)	0.826(0.764,0.893)	0.795(0.733,0.863)
	Young	1.002(0.976,1.029)	0.987(0.946,1.030)	0.957(0.910,1.008)	0.918(0.865,0.974)	0.874(0.818,0.935)	0.833(0.773,0.896)	0.795(0.736,0.859)	0.763(0.704,0.827)
	Old	0.991(0.966,1.016)	0.974(0.935,1.014)	0.951(0.905,0.999)	0.923(0.871,0.977)	0.892(0.836,0.953)	0.862(0.801,0.927)	0.833(0.771,0.899)	0.805(0.743,0.872)
**O_3**	Male	1.141(1.099,1.185)	1.305(1.229,1.386)	1.495(1.391,1.608)	1.710(1.575,1.856)	1.943(1.772,2.131)	2.186(1.975,2.418)	2.426(2.181,2.698)	2.654(2.374,2.966)
	Female	1.106(1.064,1.150)	1.246(1.172,1.326)	1.427(1.324,1.537)	1.646(1.514,1.791)	1.893(1.722,2.081)	2.145(1.934,2.379)	2.377(2.132,2.650)	2.566(2.289,2.875)
	Young	1.132(1.089,1.178)	1.287(1.209,1.369)	1.465(1.359,1.578)	1.665(1.531,1.810)	1.881(1.712,2.066)	2.103(1.898,2.330)	2.320(2.084,2.583)	2.520(2.253,2.819)
	Old	1.093(1.053,1.134)	1.211(1.141,1.284)	1.357(1.263,1.457)	1.528(1.409,1.658)	1.717(1.565,1.883)	1.906(1.722,2.111)	2.081(1.868,2.317)	2.226(1.988,2.492)

Supplementary Table 35 Subgroup analysis by gender and age of single-day lagged relative risks (RRs) and 95% confidence intervals (CIs) for the impact of extremely low air pollution concentrations on CHD hospitalizations in Henan Province, 2016–2021(df of relative humidity = 4)

pollutant	group	Lag0	Lag1	Lag2	Lag3	Lag4	Lag5	Lag6	Lag7
		RR 95% CI	RR 95% CI	RR 95% CI	RR 95% CI	RR 95% CI	RR 95% CI	RR 95% CI	RR 95% CI
**CO**	Male	0.995(0.977,1.014)	0.997(0.986,1.008)	0.999(0.990,1.007)	1.000(0.990,1.011)	1.002(0.991,1.013)	1.003(0.994,1.012)	1.004(0.994,1.014)	1.005(0.988,1.022)
	Female	0.989(0.970,1.007)	0.997(0.986,1.008)	1.004(0.995,1.013)	1.008(0.997,1.019)	1.008(0.997,1.020)	1.005(0.995,1.014)	0.998(0.988,1.009)	0.990(0.974,1.007)
	Young	1.002(0.984,1.020)	0.995(0.984,1.005)	0.988(0.980,0.997)	0.984(0.974,0.995)	0.983(0.973,0.994)	0.985(0.976,0.994)	0.989(0.979,0.999)	0.994(0.978,1.011)
	Old	0.981(0.963,0.999)	0.994(0.983,1.004)	1.005(0.996,1.014)	1.013(1.002,1.024)	1.015(1.004,1.027)	1.013(1.004,1.022)	1.007(0.996,1.017)	0.999(0.982,1.016)
**$PM_{2.5}$**	Male	0.995(0.983,1.007)	0.996(0.988,1.004)	0.997(0.989,1.004)	0.997(0.989,1.006)	0.998(0.989,1.007)	0.999(0.991,1.006)	0.999(0.991,1.007)	1.000(0.989,1.011)
	Female	0.990(0.977,1.002)	0.993(0.985,1.001)	0.996(0.988,1.003)	0.998(0.989,1.007)	0.999(0.990,1.008)	0.999(0.991,1.007)	0.998(0.990,1.006)	0.997(0.986,1.009)
	Young	1.000(0.988,1.013)	0.997(0.989,1.005)	0.994(0.987,1.001)	0.992(0.983,1.001)	0.992(0.983,1.001)	0.994(0.986,1.001)	0.996(0.989,1.004)	1.000(0.988,1.012)
	Old	0.989(0.977,1.001)	0.995(0.987,1.003)	1.000(0.992,1.007)	1.003(0.995,1.012)	1.005(0.996,1.014)	1.004(0.996,1.012)	1.002(0.994,1.010)	0.999(0.988,1.011)
**PM_{10}**	Male	0.983(0.968,0.999)	0.979(0.968,0.989)	0.975(0.965,0.985)	0.973(0.961,0.984)	0.973(0.961,0.985)	0.976(0.966,0.986)	0.980(0.969,0.991)	0.985(0.970,1.002)
	Female	0.980(0.964,0.996)	0.980(0.969,0.991)	0.980(0.970,0.991)	0.980(0.968,0.992)	0.980(0.968,0.992)	0.979(0.969,0.990)	0.979(0.968,0.990)	0.978(0.962,0.994)
	Young	0.993(0.976,1.010)	0.983(0.972,0.994)	0.974(0.964,0.984)	0.969(0.957,0.981)	0.968(0.956,0.980)	0.971(0.961,0.981)	0.978(0.967,0.989)	0.986(0.969,1.002)
	Old	0.979(0.964,0.994)	0.982(0.972,0.993)	0.985(0.975,0.995)	0.987(0.975,0.999)	0.988(0.976,1.000)	0.987(0.977,0.997)	0.986(0.975,0.996)	0.984(0.968,1.000)
**SO_2**	Male	1.004(0.988,1.019)	0.992(0.983,1.001)	0.981(0.974,0.988)	0.973(0.965,0.982)	0.970(0.961,0.979)	0.971(0.963,0.978)	0.974(0.965,0.983)	0.979(0.965,0.993)
	Female	1.000(0.984,1.016)	0.991(0.982,1.000)	0.983(0.976,0.990)	0.977(0.968,0.986)	0.974(0.965,0.983)	0.973(0.966,0.981)	0.974(0.966,0.983)	0.976(0.962,0.991)
	Young	0.999(0.984,1.014)	0.986(0.977,0.994)	0.974(0.967,0.981)	0.966(0.958,0.974)	0.963(0.954,0.971)	0.964(0.957,0.972)	0.969(0.961,0.978)	0.976(0.962,0.990)
	Old	1.009(0.993,1.025)	1.001(0.992,1.011)	0.994(0.986,1.001)	0.988(0.979,0.997)	0.984(0.975,0.993)	0.982(0.974,0.990)	0.981(0.973,0.990)	0.981(0.967,0.996)
**NO_2**	Male	0.996(0.970,1.022)	0.983(0.967,0.999)	0.972(0.957,0.986)	0.963(0.946,0.981)	0.959(0.942,0.977)	0.959(0.944,0.974)	0.962(0.946,0.978)	0.966(0.942,0.989)
	Female	0.994(0.968,1.020)	0.984(0.968,1.001)	0.976(0.961,0.991)	0.969(0.952,0.987)	0.965(0.947,0.983)	0.963(0.947,0.978)	0.962(0.946,0.978)	0.962(0.939,0.986)
	Young	1.002(0.976,1.029)	0.985(0.968,1.002)	0.970(0.955,0.985)	0.959(0.941,0.976)	0.953(0.935,0.970)	0.952(0.937,0.967)	0.955(0.939,0.971)	0.960(0.936,0.984)
	Old	0.991(0.966,1.016)	0.983(0.967,0.999)	0.976(0.962,0.991)	0.971(0.954,0.988)	0.967(0.950,0.985)	0.966(0.951,0.981)	0.966(0.950,0.982)	0.967(0.944,0.990)
**O_3**	Male	1.141(1.099,1.185)	1.144(1.117,1.172)	1.145(1.122,1.169)	1.143(1.116,1.171)	1.136(1.108,1.165)	1.125(1.101,1.149)	1.110(1.086,1.135)	1.094(1.059,1.130)
	Female	1.106(1.064,1.150)	1.127(1.100,1.155)	1.145(1.121,1.169)	1.154(1.125,1.183)	1.150(1.121,1.180)	1.133(1.108,1.159)	1.108(1.083,1.134)	1.079(1.044,1.116)
	Young	1.132(1.089,1.178)	1.136(1.109,1.165)	1.138(1.115,1.162)	1.137(1.109,1.165)	1.130(1.101,1.159)	1.118(1.094,1.143)	1.103(1.078,1.129)	1.086(1.050,1.124)
	Old	1.093(1.053,1.134)	1.108(1.082,1.135)	1.121(1.098,1.144)	1.127(1.100,1.154)	1.123(1.096,1.151)	1.110(1.087,1.135)	1.091(1.068,1.116)	1.070(1.036,1.105)

Supplementary Table 36 Subgroup analysis by gender and age of cumulative lagged relative risks (RRs) and 95% confidence intervals (CIs) for the impact of extremely high air pollution concentrations on CHD hospitalizations in Henan Province, 2016–2021(df of relative humidity = 4)

pollutant	group	Lag00	Lag01	Lag02	Lag03	Lag04	Lag05	Lag06	Lag07
		RR 95% CI	RR 95% CI	RR 95% CI	RR 95% CI	RR 95% CI	RR 95% CI	RR 95% CI	RR 95% CI
**CO**	Male	1.021(0.938,1.112)	1.035(0.907,1.181)	1.041(0.894,1.213)	1.040(0.883,1.225)	1.032(0.867,1.229)	1.018(0.850,1.220)	1.000(0.840,1.190)	0.977(0.824,1.159)
	Female	1.055(0.968,1.150)	1.070(0.936,1.224)	1.051(0.900,1.227)	1.012(0.857,1.194)	0.973(0.815,1.161)	0.952(0.793,1.143)	0.960(0.804,1.146)	1.005(0.845,1.195)
	Young	0.993(0.912,1.080)	1.019(0.893,1.161)	1.075(0.924,1.251)	1.157(0.983,1.360)	1.250(1.052,1.486)	1.340(1.121,1.601)	1.409(1.187,1.673)	1.448(1.224,1.714)
	Old	1.096(1.006,1.193)	1.129(0.990,1.289)	1.104(0.947,1.287)	1.041(0.883,1.227)	0.969(0.812,1.156)	0.913(0.761,1.096)	0.885(0.742,1.057)	0.890(0.748,1.060)
**$PM_{2.5}$**	Male	1.029(0.958,1.106)	1.055(0.940,1.185)	1.077(0.934,1.242)	1.095(0.927,1.292)	1.108(0.915,1.342)	1.117(0.903,1.383)	1.122(0.895,1.406)	1.122(0.886,1.422)
	Female	1.063(0.988,1.144)	1.109(0.986,1.248)	1.137(0.984,1.314)	1.151(0.973,1.362)	1.158(0.954,1.405)	1.164(0.939,1.444)	1.175(0.935,1.477)	1.194(0.940,1.516)
	Young	0.997(0.926,1.074)	1.016(0.902,1.143)	1.053(0.911,1.217)	1.103(0.933,1.303)	1.156(0.955,1.399)	1.200(0.971,1.482)	1.225(0.980,1.531)	1.225(0.971,1.547)
	Old	1.067(0.994,1.145)	1.100(0.981,1.234)	1.101(0.956,1.269)	1.079(0.914,1.275)	1.049(0.864,1.273)	1.023(0.824,1.270)	1.011(0.803,1.272)	1.015(0.797,1.292)
**PM_{10}**	Male	1.062(1.002,1.125)	1.147(1.045,1.260)	1.258(1.119,1.413)	1.390(1.210,1.596)	1.534(1.304,1.805)	1.676(1.397,2.011)	1.802(1.484,2.187)	1.899(1.548,2.329)
	Female	1.076(1.014,1.142)	1.156(1.051,1.273)	1.243(1.103,1.400)	1.335(1.159,1.539)	1.437(1.217,1.697)	1.548(1.285,1.866)	1.673(1.372,2.039)	1.811(1.470,2.232)
	Young	1.025(0.964,1.090)	1.091(0.988,1.204)	1.198(1.060,1.353)	1.343(1.164,1.550)	1.512(1.280,1.785)	1.680(1.396,2.022)	1.823(1.499,2.217)	1.920(1.563,2.359)
	Old	1.080(1.021,1.142)	1.152(1.052,1.261)	1.216(1.085,1.362)	1.274(1.112,1.460)	1.332(1.135,1.563)	1.395(1.165,1.670)	1.469(1.212,1.779)	1.556(1.272,1.905)
**SO_2**	Male	0.973(0.871,1.088)	1.034(0.870,1.230)	1.187(0.972,1.450)	1.442(1.166,1.785)	1.798(1.435,2.253)	2.230(1.771,2.808)	2.695(2.159,3.363)	3.141(2.522,3.912)
	Female	1.000(0.893,1.120)	1.067(0.895,1.272)	1.206(0.985,1.478)	1.426(1.149,1.770)	1.728(1.375,2.172)	2.103(1.664,2.657)	2.536(2.026,3.174)	3.011(2.411,3.759)
	Young	1.008(0.904,1.124)	1.119(0.946,1.325)	1.353(1.114,1.642)	1.735(1.411,2.134)	2.280(1.831,2.838)	2.959(2.365,3.702)	3.703(2.986,4.591)	4.412(3.568,5.457)
	Old	0.938(0.836,1.052)	0.931(0.778,1.114)	0.973(0.791,1.198)	1.061(0.850,1.325)	1.191(0.941,1.506)	1.355(1.065,1.725)	1.550(1.228,1.958)	1.774(1.407,2.237)
**NO_2**	Male	1.010(0.951,1.073)	1.052(0.955,1.158)	1.125(1.000,1.266)	1.228(1.072,1.407)	1.354(1.159,1.582)	1.493(1.258,1.773)	1.637(1.366,1.960)	1.776(1.471,2.145)
	Female	1.015(0.955,1.079)	1.053(0.955,1.162)	1.115(0.990,1.257)	1.201(1.046,1.378)	1.306(1.116,1.529)	1.428(1.200,1.699)	1.562(1.302,1.875)	1.708(1.412,2.067)
	Young	0.995(0.935,1.058)	1.031(0.934,1.137)	1.107(0.982,1.248)	1.221(1.065,1.401)	1.367(1.169,1.599)	1.533(1.290,1.822)	1.708(1.425,2.046)	1.880(1.557,2.270)
	Old	1.022(0.964,1.084)	1.064(0.968,1.169)	1.126(1.003,1.263)	1.207(1.056,1.379)	1.304(1.119,1.521)	1.414(1.193,1.677)	1.534(1.282,1.835)	1.660(1.376,2.003)
**O_3**	Male	0.815(0.769,0.864)	0.662(0.603,0.727)	0.537(0.480,0.601)	0.436(0.384,0.495)	0.358(0.310,0.413)	0.298(0.255,0.349)	0.254(0.215,0.299)	0.221(0.186,0.263)
	Female	0.856(0.806,0.909)	0.711(0.647,0.783)	0.577(0.514,0.648)	0.463(0.406,0.527)	0.373(0.322,0.431)	0.307(0.262,0.361)	0.262(0.222,0.310)	0.233(0.195,0.278)
	Young	0.825(0.776,0.877)	0.677(0.615,0.746)	0.554(0.494,0.622)	0.455(0.399,0.518)	0.376(0.326,0.435)	0.317(0.270,0.371)	0.272(0.231,0.321)	0.239(0.201,0.285)
	Old	0.872(0.823,0.923)	0.744(0.679,0.815)	0.624(0.558,0.697)	0.519(0.457,0.589)	0.434(0.376,0.500)	0.369(0.315,0.432)	0.322(0.273,0.380)	0.290(0.244,0.345)

Supplementary Table 37 Subgroup analysis by gender and age of single-day lagged relative risks (RRs) and 95% confidence intervals (CIs) for the impact of extremely high air pollution concentrations on CHD hospitalizations in Henan Province, 2016–2021(df of relative humidity = 4)

pollutant	group	Lag0	Lag1	Lag2	Lag3	Lag4	Lag5	Lag6	Lag7
		RR 95% CI	RR 95% CI	RR 95% CI	RR 95% CI	RR 95% CI	RR 95% CI	RR 95% CI	RR 95% CI
**CO**	Male	1.021(0.938,1.112)	1.013(0.964,1.066)	1.006(0.966,1.047)	0.999(0.950,1.050)	0.992(0.943,1.044)	0.987(0.946,1.030)	0.982(0.936,1.030)	0.977(0.904,1.056)
	Female	1.055(0.968,1.150)	1.015(0.964,1.068)	0.982(0.942,1.023)	0.962(0.915,1.013)	0.961(0.913,1.013)	0.979(0.937,1.022)	1.009(0.961,1.059)	1.047(0.967,1.132)
	Young	0.993(0.912,1.080)	1.026(0.976,1.079)	1.056(1.014,1.099)	1.076(1.024,1.130)	1.081(1.028,1.137)	1.071(1.027,1.118)	1.052(1.003,1.103)	1.028(0.952,1.110)
	Old	1.096(1.006,1.193)	1.031(0.980,1.084)	0.978(0.938,1.019)	0.943(0.896,0.991)	0.931(0.884,0.980)	0.942(0.903,0.984)	0.969(0.924,1.017)	1.005(0.930,1.087)
**$PM_{2.5}$**	Male	1.029(0.958,1.106)	1.025(0.978,1.075)	1.021(0.978,1.066)	1.016(0.966,1.070)	1.012(0.961,1.066)	1.008(0.964,1.055)	1.004(0.959,1.052)	1.000(0.936,1.069)
	Female	1.063(0.988,1.144)	1.043(0.994,1.094)	1.025(0.982,1.071)	1.012(0.961,1.066)	1.006(0.954,1.060)	1.005(0.961,1.052)	1.010(0.963,1.058)	1.016(0.950,1.086)
	Young	0.997(0.926,1.074)	1.018(0.971,1.068)	1.037(0.993,1.082)	1.048(0.996,1.102)	1.048(0.995,1.104)	1.038(0.992,1.086)	1.021(0.974,1.069)	1.000(0.935,1.070)
	Old	1.067(0.994,1.145)	1.031(0.984,1.081)	1.001(0.958,1.046)	0.980(0.931,1.032)	0.972(0.922,1.024)	0.976(0.932,1.021)	0.988(0.943,1.034)	1.004(0.941,1.072)
**PM_{10}**	Male	1.062(1.002,1.125)	1.080(1.039,1.123)	1.096(1.056,1.137)	1.105(1.058,1.154)	1.104(1.057,1.153)	1.093(1.053,1.134)	1.075(1.034,1.118)	1.054(0.994,1.117)
	Female	1.076(1.014,1.142)	1.075(1.033,1.118)	1.075(1.035,1.116)	1.075(1.028,1.124)	1.076(1.029,1.125)	1.078(1.037,1.120)	1.080(1.038,1.124)	1.083(1.020,1.150)
	Young	1.025(0.964,1.090)	1.064(1.022,1.108)	1.098(1.058,1.141)	1.121(1.072,1.172)	1.126(1.076,1.177)	1.111(1.070,1.154)	1.085(1.042,1.129)	1.053(0.991,1.119)
	Old	1.080(1.021,1.142)	1.067(1.027,1.108)	1.056(1.018,1.095)	1.048(1.004,1.094)	1.045(1.001,1.091)	1.047(1.010,1.087)	1.053(1.013,1.094)	1.060(1.001,1.122)
**SO_2**	Male	0.973(0.871,1.088)	1.063(0.995,1.135)	1.148(1.090,1.209)	1.215(1.140,1.295)	1.246(1.168,1.331)	1.240(1.174,1.311)	1.209(1.134,1.288)	1.165(1.051,1.292)
	Female	1.000(0.893,1.120)	1.067(0.998,1.141)	1.131(1.072,1.192)	1.182(1.108,1.261)	1.212(1.134,1.295)	1.217(1.151,1.287)	1.206(1.131,1.287)	1.187(1.069,1.318)
	Young	1.008(0.904,1.124)	1.110(1.042,1.184)	1.208(1.148,1.271)	1.283(1.205,1.366)	1.314(1.232,1.401)	1.298(1.230,1.370)	1.251(1.176,1.332)	1.192(1.077,1.319)
	Old	0.938(0.836,1.052)	0.993(0.927,1.063)	1.045(0.990,1.103)	1.090(1.021,1.164)	1.122(1.049,1.200)	1.138(1.075,1.205)	1.144(1.071,1.222)	1.144(1.030,1.272)
**NO_2**	Male	1.010(0.951,1.073)	1.041(1.001,1.083)	1.070(1.033,1.108)	1.091(1.047,1.138)	1.103(1.057,1.150)	1.103(1.063,1.144)	1.096(1.054,1.139)	1.085(1.025,1.148)
	Female	1.015(0.955,1.079)	1.038(0.997,1.080)	1.059(1.022,1.097)	1.076(1.032,1.123)	1.088(1.042,1.136)	1.093(1.053,1.135)	1.094(1.052,1.138)	1.093(1.032,1.158)
	Young	0.995(0.935,1.058)	1.036(0.996,1.078)	1.074(1.036,1.113)	1.104(1.058,1.151)	1.120(1.072,1.169)	1.121(1.081,1.164)	1.113(1.071,1.158)	1.101(1.039,1.166)
	Old	1.022(0.964,1.084)	1.041(1.002,1.081)	1.058(1.022,1.095)	1.072(1.029,1.117)	1.081(1.037,1.127)	1.084(1.046,1.125)	1.084(1.044,1.126)	1.082(1.024,1.144)
**O_3**	Male	0.815(0.769,0.864)	0.812(0.783,0.843)	0.811(0.785,0.837)	0.813(0.783,0.844)	0.820(0.789,0.853)	0.834(0.806,0.862)	0.851(0.822,0.881)	0.871(0.828,0.915)
	Female	0.856(0.806,0.909)	0.831(0.800,0.863)	0.811(0.785,0.838)	0.801(0.771,0.833)	0.806(0.774,0.838)	0.824(0.796,0.853)	0.853(0.823,0.884)	0.889(0.843,0.936)
	Young	0.825(0.776,0.877)	0.821(0.790,0.853)	0.819(0.792,0.846)	0.820(0.789,0.853)	0.828(0.796,0.861)	0.841(0.813,0.871)	0.859(0.829,0.890)	0.880(0.835,0.927)
	Old	0.872(0.823,0.923)	0.853(0.823,0.885)	0.839(0.812,0.866)	0.832(0.801,0.863)	0.836(0.804,0.868)	0.851(0.823,0.879)	0.873(0.844,0.904)	0.901(0.857,0.947)

Supplementary Table 38 Relative risks (RRs) and 95% confidence intervals (CIs) of coronary heart disease hospitalizations associated with extremely low concentrations of each pollutant in Henan Province from 2016 to 2021(the maximum lag period = 5)

Lag days	CO		$PM_{2.5}$		PM_{10}		SO_2		NO_2		O_3	
	RR	95% CI	RR	95% CI	RR	95% CI	RR	95% CI	RR	95% CI	RR	95% CI
lag0	0.988	(0.972,1.004)	0.993	(0.982,1.004)	0.982	(0.968,0.996)	0.999	(0.985,1.013)	0.986	(0.963,1.009)	1.135	(1.096,1.174)
lag1	1.000	(0.991,1.010)	0.998	(0.990,1.006)	0.979	(0.968,0.989)	0.981	(0.973,0.989)	0.972	(0.957,0.988)	1.140	(1.116,1.166)
lag2	1.008	(0.998,1.018)	1.001	(0.993,1.010)	0.976	(0.965,0.988)	0.969	(0.961,0.977)	0.962	(0.946,0.979)	1.143	(1.116,1.170)
lag3	1.007	(0.997,1.018)	1.001	(0.993,1.010)	0.977	(0.966,0.989)	0.966	(0.958,0.975)	0.960	(0.944,0.977)	1.138	(1.111,1.166)
lag4	0.998	(0.988,1.007)	0.999	(0.991,1.006)	0.981	(0.970,0.991)	0.974	(0.966,0.982)	0.966	(0.951,0.981)	1.127	(1.103,1.152)
lag5	0.984	(0.969,0.999)	0.995	(0.985,1.005)	0.985	(0.972,1.000)	0.986	(0.974,0.999)	0.975	(0.954,0.997)	1.113	(1.080,1.146)
Lag01	0.988	(0.964,1.012)	0.991	(0.974,1.009)	0.961	(0.939,0.984)	0.980	(0.960,1.000)	0.958	(0.924,0.994)	1.294	(1.227,1.364)
Lag02	0.996	(0.968,1.025)	0.992	(0.970,1.015)	0.938	(0.910,0.968)	0.949	(0.927,0.972)	0.922	(0.880,0.966)	1.479	(1.385,1.578)
Lag03	1.004	(0.973,1.035)	0.994	(0.967,1.021)	0.917	(0.883,0.952)	0.917	(0.894,0.941)	0.885	(0.837,0.936)	1.683	(1.559,1.817)
Lag04	1.001	(0.970,1.034)	0.993	(0.962,1.023)	0.899	(0.861,0.939)	0.893	(0.870,0.917)	0.855	(0.804,0.910)	1.897	(1.742,2.066)
Lag05	0.985	(0.954,1.017)	0.987	(0.955,1.020)	0.886	(0.846,0.928)	0.881	(0.858,0.905)	0.834	(0.781,0.890)	2.111	(1.926,2.314)

Supplementary Table 39 Relative risks (RRs) and 95% confidence intervals (CIs) of coronary heart disease hospitalizations associated with extremely high concentrations of each pollutant in Henan Province from 2016 to 2021(the maximum lag period = 5)

Lag days	CO		$PM_{2.5}$		PM_{10}		SO_2		NO_2		O_3	
	RR	95% CI	RR	95% CI	RR	95% CI	RR	95% CI	RR	95% CI	RR	95% CI
lag0	1.060	(0.983,1.142)	1.041	(0.976,1.110)	1.067	(1.013,1.124)	1.010	(0.914,1.116)	1.034	(0.980,1.091)	0.822	(0.780,0.867)
lag1	0.999	(0.956,1.045)	1.012	(0.968,1.059)	1.081	(1.041,1.122)	1.147	(1.084,1.215)	1.069	(1.030,1.109)	0.816	(0.789,0.844)
lag2	0.962	(0.919,1.007)	0.993	(0.946,1.043)	1.089	(1.044,1.136)	1.256	(1.183,1.334)	1.094	(1.051,1.139)	0.814	(0.785,0.844)
lag3	0.966	(0.922,1.012)	0.991	(0.944,1.042)	1.087	(1.042,1.134)	1.279	(1.203,1.360)	1.099	(1.056,1.144)	0.819	(0.789,0.850)
lag4	1.010	(0.967,1.056)	1.007	(0.963,1.053)	1.073	(1.033,1.114)	1.210	(1.143,1.281)	1.085	(1.046,1.125)	0.831	(0.804,0.859)
lag5	1.079	(1.007,1.157)	1.031	(0.971,1.095)	1.054	(1.002,1.109)	1.104	(1.005,1.212)	1.060	(1.007,1.117)	0.847	(0.809,0.887)
Lag01	1.059	(0.946,1.185)	1.054	(0.951,1.169)	1.153	(1.060,1.255)	1.158	(0.999,1.343)	1.105	(1.015,1.203)	0.671	(0.619,0.728)
Lag02	1.019	(0.893,1.162)	1.047	(0.917,1.196)	1.256	(1.125,1.403)	1.455	(1.228,1.725)	1.209	(1.084,1.348)	0.546	(0.494,0.604)
Lag03	0.984	(0.851,1.138)	1.038	(0.884,1.219)	1.365	(1.191,1.564)	1.861	(1.546,2.242)	1.329	(1.166,1.514)	0.447	(0.397,0.503)
Lag04	0.994	(0.857,1.153)	1.045	(0.873,1.251)	1.464	(1.255,1.708)	2.253	(1.866,2.720)	1.441	(1.248,1.665)	0.371	(0.326,0.424)
Lag05	1.073	(0.926,1.243)	1.078	(0.889,1.307)	1.543	(1.308,1.821)	2.486	(2.057,3.005)	1.529	(1.311,1.782)	0.315	(0.273,0.363)

Supplementary Table 40 Relative risks (RRs) and 95% confidence intervals (CIs) of coronary heart disease hospitalizations associated with extremely low concentrations of each pollutant in Henan Province from 2016 to 2021(the maximum lag period = 6)

Lag days	CO		$PM_{2.5}$		PM_{10}		SO_2		NO_2		O_3	
	RR	95% CI	RR	95% CI	RR	95% CI	RR	95% CI	RR	95% CI	RR	95% CI
lag0	0.989	(0.973,1.005)	0.994	(0.983,1.005)	0.984	(0.970,0.999)	1.002	(0.988,1.016)	0.991	(0.968,1.014)	1.119	(1.081,1.158)
lag1	0.997	(0.988,1.006)	0.996	(0.989,1.004)	0.981	(0.971,0.991)	0.989	(0.981,0.997)	0.977	(0.962,0.991)	1.137	(1.112,1.162)
lag2	1.003	(0.994,1.012)	0.999	(0.991,1.006)	0.978	(0.968,0.989)	0.978	(0.971,0.985)	0.965	(0.951,0.980)	1.148	(1.125,1.173)
lag3	1.005	(0.995,1.015)	1.000	(0.992,1.008)	0.977	(0.966,0.989)	0.971	(0.963,0.979)	0.959	(0.943,0.976)	1.148	(1.121,1.175)
lag4	1.003	(0.993,1.012)	1.000	(0.992,1.008)	0.978	(0.968,0.989)	0.971	(0.963,0.978)	0.960	(0.945,0.975)	1.130	(1.106,1.155)
lag5	0.996	(0.987,1.005)	0.998	(0.991,1.006)	0.981	(0.971,0.991)	0.975	(0.967,0.982)	0.967	(0.952,0.981)	1.100	(1.078,1.124)
Lag6	0.988	(0.973,1.003)	0.996	(0.986,1.007)	0.985	(0.970,0.999)	0.981	(0.968,0.994)	0.976	(0.955,0.997)	1.066	(1.035,1.098)
Lag01	0.986	(0.962,1.011)	0.990	(0.973,1.008)	0.965	(0.943,0.988)	0.991	(0.970,1.012)	0.968	(0.933,1.004)	1.272	(1.205,1.342)
Lag02	0.989	(0.961,1.018)	0.989	(0.967,1.011)	0.944	(0.916,0.973)	0.969	(0.945,0.993)	0.934	(0.893,0.977)	1.461	(1.368,1.560)
Lag03	0.994	(0.964,1.026)	0.989	(0.963,1.015)	0.923	(0.890,0.957)	0.941	(0.916,0.966)	0.896	(0.849,0.945)	1.676	(1.554,1.808)
Lag04	0.997	(0.964,1.030)	0.989	(0.959,1.019)	0.903	(0.865,0.942)	0.913	(0.888,0.939)	0.860	(0.809,0.914)	1.894	(1.739,2.063)
Lag05	0.993	(0.961,1.026)	0.987	(0.955,1.020)	0.886	(0.846,0.928)	0.890	(0.866,0.915)	0.832	(0.779,0.888)	2.084	(1.902,2.284)
Lag06	0.981	(0.950,1.013)	0.984	(0.950,1.018)	0.872	(0.831,0.916)	0.873	(0.850,0.897)	0.811	(0.757,0.870)	2.221	(2.018,2.446)

Supplementary Table 41 Relative risks (RRs) and 95% confidence intervals (CIs) of coronary heart disease hospitalizations associated with extremely high concentrations of each pollutant in Henan Province from 2016 to 2021(the maximum lag period = 6)

Lag days	CO		$PM_{2.5}$		PM_{10}		SO_2		NO_2		O_3	
	RR	95% CI	RR	95% CI	RR	95% CI	RR	95% CI	RR	95% CI	RR	95% CI
lag0	1.052	(0.975,1.135)	1.039	(0.974,1.108)	1.058	(1.004,1.115)	0.984	(0.890,1.089)	1.022	(0.968,1.078)	0.840	(0.797,0.886)
lag1	1.014	(0.970,1.060)	1.021	(0.978,1.066)	1.072	(1.034,1.111)	1.085	(1.024,1.150)	1.057	(1.020,1.094)	0.820	(0.793,0.848)
lag2	0.986	(0.947,1.027)	1.007	(0.964,1.051)	1.082	(1.042,1.124)	1.177	(1.117,1.240)	1.086	(1.049,1.125)	0.807	(0.782,0.834)
lag3	0.976	(0.931,1.023)	0.999	(0.952,1.050)	1.086	(1.041,1.133)	1.234	(1.161,1.312)	1.102	(1.059,1.147)	0.808	(0.779,0.838)
lag4	0.988	(0.947,1.031)	1.001	(0.957,1.047)	1.082	(1.042,1.123)	1.239	(1.173,1.309)	1.099	(1.060,1.140)	0.828	(0.800,0.856)
lag5	1.018	(0.975,1.064)	1.009	(0.967,1.054)	1.071	(1.033,1.110)	1.203	(1.136,1.274)	1.083	(1.045,1.121)	0.862	(0.835,0.891)
Lag6	1.059	(0.987,1.135)	1.021	(0.961,1.085)	1.057	(1.003,1.113)	1.149	(1.046,1.262)	1.059	(1.006,1.115)	0.906	(0.866,0.949)
Lag01	1.067	(0.950,1.198)	1.060	(0.956,1.176)	1.135	(1.043,1.235)	1.068	(0.917,1.245)	1.080	(0.992,1.175)	0.689	(0.634,0.749)
Lag02	1.052	(0.920,1.203)	1.068	(0.938,1.215)	1.228	(1.102,1.367)	1.257	(1.054,1.499)	1.173	(1.056,1.302)	0.556	(0.503,0.616)
Lag03	1.027	(0.887,1.189)	1.067	(0.914,1.246)	1.333	(1.169,1.521)	1.552	(1.283,1.877)	1.292	(1.141,1.464)	0.450	(0.400,0.506)
Lag04	1.015	(0.870,1.185)	1.068	(0.894,1.276)	1.442	(1.238,1.680)	1.922	(1.574,2.347)	1.421	(1.232,1.638)	0.372	(0.326,0.425)
Lag05	1.034	(0.887,1.204)	1.078	(0.889,1.307)	1.544	(1.309,1.821)	2.312	(1.902,2.812)	1.538	(1.319,1.792)	0.321	(0.279,0.370)
Lag06	1.094	(0.941,1.272)	1.101	(0.898,1.350)	1.632	(1.372,1.942)	2.657	(2.188,3.227)	1.629	(1.386,1.915)	0.291	(0.251,0.338)

Supplementary Table 42 Relative risks (RRs) and 95% confidence intervals (CIs) of coronary heart disease hospitalizations associated with extremely low concentrations of each pollutant in Henan Province from 2016 to 2021(the maximum lag period = 7)

Lag days	CO		$PM_{2.5}$		PM_{10}		SO_2		NO_2		O_3	
	RR	95% CI	RR	95% CI	RR	95% CI	RR	95% CI	RR	95% CI	RR	95% CI
lag0	0.991	(0.975,1.007)	0.994	(0.983,1.005)	0.985	(0.970,0.999)	1.004	(0.990,1.018)	0.996	(0.973,1.019)	1.111	(1.074,1.149)
lag1	0.995	(0.985,1.004)	0.996	(0.988,1.003)	0.982	(0.972,0.992)	0.993	(0.985,1.001)	0.984	(0.969,0.998)	1.123	(1.099,1.147)
lag2	0.998	(0.990,1.006)	0.997	(0.991,1.004)	0.980	(0.971,0.989)	0.984	(0.977,0.990)	0.973	(0.960,0.986)	1.131	(1.111,1.152)
lag3	1.000	(0.991,1.010)	0.998	(0.991,1.006)	0.978	(0.968,0.989)	0.977	(0.969,0.985)	0.965	(0.950,0.980)	1.135	(1.110,1.160)
lag4	1.001	(0.991,1.011)	0.999	(0.991,1.007)	0.978	(0.968,0.989)	0.973	(0.965,0.981)	0.960	(0.945,0.976)	1.130	(1.105,1.156)
lag5	1.000	(0.992,1.009)	1.000	(0.993,1.006)	0.980	(0.971,0.989)	0.973	(0.966,0.980)	0.959	(0.946,0.973)	1.117	(1.096,1.139)
Lag6	0.999	(0.990,1.008)	1.000	(0.993,1.007)	0.982	(0.972,0.991)	0.975	(0.968,0.983)	0.961	(0.947,0.975)	1.099	(1.077,1.121)
Lag7	0.997	(0.982,1.012)	0.999	(0.989,1.010)	0.984	(0.970,0.999)	0.979	(0.966,0.991)	0.963	(0.942,0.984)	1.079	(1.047,1.111)
Lag01	0.985	(0.961,1.011)	0.990	(0.972,1.007)	0.967	(0.944,0.990)	0.997	(0.975,1.018)	0.979	(0.944,1.016)	1.247	(1.182,1.316)
Lag02	0.983	(0.955,1.012)	0.987	(0.965,1.009)	0.947	(0.920,0.975)	0.980	(0.956,1.005)	0.953	(0.911,0.997)	1.411	(1.322,1.506)
Lag03	0.983	(0.953,1.015)	0.985	(0.960,1.011)	0.927	(0.895,0.960)	0.958	(0.932,0.984)	0.919	(0.873,0.968)	1.601	(1.487,1.723)
Lag04	0.984	(0.952,1.018)	0.984	(0.956,1.014)	0.907	(0.871,0.944)	0.932	(0.906,0.959)	0.883	(0.832,0.937)	1.809	(1.665,1.965)
Lag05	0.985	(0.951,1.019)	0.984	(0.952,1.017)	0.888	(0.849,0.930)	0.907	(0.881,0.934)	0.847	(0.793,0.904)	2.021	(1.845,2.213)
Lag06	0.984	(0.951,1.017)	0.984	(0.950,1.018)	0.872	(0.831,0.915)	0.885	(0.860,0.910)	0.813	(0.759,0.871)	2.221	(2.019,2.444)
Lag07	0.980	(0.949,1.013)	0.983	(0.948,1.019)	0.859	(0.816,0.903)	0.866	(0.842,0.890)	0.783	(0.729,0.842)	2.396	(2.168,2.649)

Supplementary Table 43 Relative risks (RRs) and 95% confidence intervals (CIs) of coronary heart disease hospitalizations associated with extremely high concentrations of each pollutant in Henan Province from 2016 to 2021(the maximum lag period = 7)

Lag days	CO		$PM_{2.5}$		PM_{10}		SO_2		NO_2		O_3	
	RR	95% CI	RR	95% CI	RR	95% CI	RR	95% CI	RR	95% CI	RR	95% CI
lag0	1.044	(0.968,1.127)	1.037	(0.971,1.106)	1.057	(1.003,1.114)	0.975	(0.882,1.078)	1.010	(0.958,1.066)	0.850	(0.806,0.895)
lag1	1.026	(0.981,1.073)	1.026	(0.984,1.071)	1.067	(1.031,1.105)	1.052	(0.991,1.116)	1.039	(1.004,1.076)	0.836	(0.809,0.865)
lag2	1.010	(0.974,1.048)	1.017	(0.978,1.057)	1.076	(1.041,1.112)	1.125	(1.074,1.179)	1.066	(1.033,1.100)	0.826	(0.803,0.850)
lag3	0.999	(0.956,1.045)	1.009	(0.964,1.057)	1.081	(1.040,1.124)	1.184	(1.118,1.254)	1.087	(1.047,1.128)	0.822	(0.795,0.851)
lag4	0.995	(0.951,1.042)	1.005	(0.959,1.053)	1.081	(1.040,1.125)	1.215	(1.146,1.289)	1.099	(1.058,1.142)	0.828	(0.800,0.857)
lag5	0.998	(0.961,1.037)	1.003	(0.963,1.044)	1.076	(1.041,1.113)	1.216	(1.157,1.278)	1.102	(1.066,1.139)	0.843	(0.818,0.868)
Lag6	1.005	(0.963,1.049)	1.002	(0.962,1.045)	1.068	(1.031,1.106)	1.197	(1.130,1.267)	1.098	(1.061,1.137)	0.864	(0.838,0.891)
Lag7	1.015	(0.947,1.088)	1.003	(0.945,1.065)	1.058	(1.004,1.115)	1.168	(1.065,1.282)	1.092	(1.038,1.148)	0.889	(0.850,0.931)
Lag01	1.071	(0.952,1.206)	1.064	(0.958,1.180)	1.129	(1.037,1.228)	1.025	(0.877,1.198)	1.050	(0.964,1.145)	0.711	(0.654,0.772)
Lag02	1.082	(0.944,1.241)	1.082	(0.951,1.229)	1.214	(1.093,1.349)	1.154	(0.964,1.381)	1.120	(1.008,1.243)	0.587	(0.531,0.649)
Lag03	1.082	(0.934,1.252)	1.092	(0.940,1.267)	1.313	(1.159,1.487)	1.366	(1.127,1.655)	1.217	(1.078,1.374)	0.483	(0.431,0.541)
Lag04	1.077	(0.921,1.259)	1.097	(0.924,1.302)	1.420	(1.227,1.643)	1.660	(1.354,2.034)	1.338	(1.165,1.536)	0.400	(0.352,0.454)
Lag05	1.075	(0.914,1.263)	1.100	(0.908,1.331)	1.528	(1.298,1.799)	2.018	(1.639,2.485)	1.474	(1.265,1.718)	0.337	(0.293,0.388)
Lag06	1.080	(0.924,1.263)	1.102	(0.900,1.350)	1.633	(1.373,1.941)	2.415	(1.977,2.951)	1.619	(1.379,1.902)	0.291	(0.251,0.337)
Lag07	1.097	(0.941,1.278)	1.106	(0.894,1.367)	1.728	(1.440,2.073)	2.821	(2.314,3.440)	1.768	(1.495,2.091)	0.259	(0.222,0.302)

Supplementary Table 44 Relative risks (RRs) and 95% confidence intervals (CIs) of coronary heart disease hospitalizations associated with extremely low concentrations of each pollutant in Henan Province from 2016 to 2021(the maximum lag period = 8)

Lag days	CO		$PM_{2.5}$		PM_{10}		SO_2		NO_2		O_3	
	RR	95% CI	RR	95% CI	RR	95% CI	RR	95% CI	RR	95% CI	RR	95% CI
lag0	0.993	(0.977,1.009)	0.995	(0.984,1.006)	0.986	(0.972,1.000)	1.004	(0.990,1.018)	0.999	(0.976,1.022)	1.105	(1.068,1.142)
lag1	0.995	(0.985,1.005)	0.996	(0.989,1.003)	0.983	(0.973,0.993)	0.992	(0.984,1.000)	0.984	(0.970,1.000)	1.111	(1.087,1.135)
lag2	0.997	(0.989,1.004)	0.997	(0.991,1.003)	0.980	(0.972,0.989)	0.981	(0.975,0.987)	0.972	(0.960,0.984)	1.116	(1.097,1.136)
lag3	0.998	(0.990,1.007)	0.998	(0.991,1.005)	0.979	(0.969,0.988)	0.974	(0.967,0.981)	0.963	(0.949,0.977)	1.120	(1.098,1.142)
lag4	0.999	(0.990,1.009)	0.999	(0.991,1.007)	0.978	(0.968,0.989)	0.970	(0.962,0.978)	0.958	(0.943,0.973)	1.120	(1.096,1.145)
lag5	1.000	(0.991,1.009)	0.999	(0.992,1.007)	0.979	(0.970,0.989)	0.972	(0.965,0.980)	0.959	(0.945,0.973)	1.117	(1.095,1.141)
Lag6	1.000	(0.992,1.007)	0.999	(0.993,1.005)	0.982	(0.974,0.990)	0.979	(0.973,0.985)	0.964	(0.952,0.976)	1.112	(1.092,1.132)
Lag7	1.000	(0.990,1.009)	0.999	(0.992,1.006)	0.985	(0.976,0.995)	0.988	(0.981,0.996)	0.972	(0.958,0.987)	1.104	(1.082,1.126)
Lag8	0.999	(0.985,1.015)	0.998	(0.988,1.008)	0.989	(0.975,1.003)	0.999	(0.987,1.012)	0.982	(0.961,1.004)	1.096	(1.064,1.128)
Lag01	0.988	(0.962,1.013)	0.990	(0.973,1.008)	0.969	(0.946,0.992)	0.996	(0.974,1.018)	0.983	(0.947,1.021)	1.227	(1.163,1.296)
Lag02	0.984	(0.955,1.015)	0.988	(0.966,1.010)	0.950	(0.922,0.979)	0.977	(0.953,1.002)	0.956	(0.913,1.001)	1.370	(1.283,1.463)
Lag03	0.982	(0.951,1.015)	0.986	(0.961,1.011)	0.930	(0.898,0.962)	0.951	(0.926,0.977)	0.920	(0.873,0.969)	1.534	(1.426,1.651)
Lag04	0.982	(0.948,1.016)	0.985	(0.957,1.014)	0.910	(0.874,0.946)	0.923	(0.897,0.950)	0.881	(0.831,0.934)	1.719	(1.585,1.865)
Lag05	0.981	(0.947,1.017)	0.984	(0.953,1.017)	0.891	(0.852,0.932)	0.898	(0.872,0.925)	0.844	(0.791,0.901)	1.921	(1.756,2.101)
Lag06	0.981	(0.946,1.018)	0.983	(0.950,1.018)	0.875	(0.833,0.918)	0.879	(0.853,0.905)	0.814	(0.759,0.873)	2.136	(1.940,2.351)
Lag07	0.981	(0.948,1.015)	0.982	(0.947,1.018)	0.862	(0.819,0.906)	0.869	(0.844,0.894)	0.792	(0.737,0.850)	2.358	(2.135,2.605)
Lag08	0.981	(0.949,1.014)	0.981	(0.945,1.018)	0.852	(0.809,0.898)	0.868	(0.844,0.893)	0.778	(0.722,0.837)	2.584	(2.331,2.865)

Supplementary Table 45 Relative risks (RRs) and 95% confidence intervals (CIs) of coronary heart disease hospitalizations associated with extremely high concentrations of each pollutant in Henan Province from 2016 to 2021(the maximum lag period = 8)

Lag days	CO		$PM_{2.5}$		PM_{10}		SO_2		NO_2		O_3	
	RR	95% CI	RR	95% CI	RR	95% CI	RR	95% CI	RR	95% CI	RR	95% CI
lag0	1.034	(0.959,1.116)	1.033	(0.968,1.102)	1.052	(0.999,1.108)	0.971	(0.880,1.073)	1.003	(0.951,1.058)	0.857	(0.814,0.903)
lag1	1.025	(0.978,1.074)	1.024	(0.981,1.069)	1.064	(1.027,1.102)	1.061	(0.999,1.127)	1.037	(1.001,1.075)	0.850	(0.822,0.879)
lag2	1.016	(0.982,1.051)	1.016	(0.980,1.054)	1.074	(1.042,1.107)	1.146	(1.098,1.197)	1.069	(1.038,1.100)	0.844	(0.822,0.866)
lag3	1.009	(0.969,1.050)	1.010	(0.969,1.053)	1.081	(1.043,1.119)	1.212	(1.152,1.276)	1.093	(1.057,1.131)	0.839	(0.815,0.865)
lag4	1.004	(0.959,1.050)	1.006	(0.961,1.054)	1.082	(1.041,1.124)	1.242	(1.171,1.317)	1.106	(1.065,1.148)	0.839	(0.811,0.868)
lag5	1.001	(0.960,1.043)	1.004	(0.963,1.048)	1.078	(1.040,1.116)	1.223	(1.159,1.290)	1.104	(1.066,1.142)	0.842	(0.816,0.869)
Lag6	1.000	(0.966,1.036)	1.005	(0.969,1.042)	1.068	(1.036,1.100)	1.166	(1.114,1.220)	1.089	(1.057,1.122)	0.849	(0.826,0.872)
Lag7	1.001	(0.958,1.046)	1.007	(0.967,1.048)	1.055	(1.019,1.092)	1.088	(1.027,1.152)	1.068	(1.032,1.105)	0.858	(0.832,0.885)
Lag8	1.002	(0.935,1.075)	1.010	(0.952,1.071)	1.041	(0.989,1.095)	1.004	(0.917,1.100)	1.042	(0.991,1.096)	0.868	(0.830,0.908)
Lag01	1.060	(0.940,1.196)	1.058	(0.952,1.175)	1.119	(1.028,1.219)	1.030	(0.881,1.206)	1.040	(0.953,1.135)	0.728	(0.670,0.792)
Lag02	1.077	(0.934,1.242)	1.075	(0.944,1.224)	1.202	(1.081,1.337)	1.181	(0.983,1.420)	1.112	(0.999,1.238)	0.614	(0.555,0.680)
Lag03	1.087	(0.933,1.265)	1.086	(0.936,1.260)	1.299	(1.148,1.469)	1.432	(1.179,1.739)	1.216	(1.076,1.373)	0.516	(0.460,0.578)
Lag04	1.091	(0.929,1.281)	1.092	(0.923,1.293)	1.405	(1.219,1.620)	1.779	(1.451,2.180)	1.344	(1.172,1.542)	0.433	(0.382,0.491)
Lag05	1.092	(0.923,1.292)	1.097	(0.908,1.327)	1.514	(1.289,1.779)	2.175	(1.757,2.691)	1.484	(1.274,1.728)	0.364	(0.317,0.419)
Lag06	1.092	(0.922,1.293)	1.103	(0.899,1.354)	1.617	(1.358,1.925)	2.535	(2.046,3.141)	1.616	(1.373,1.903)	0.309	(0.267,0.359)
Lag07	1.093	(0.932,1.282)	1.111	(0.899,1.373)	1.706	(1.424,2.044)	2.758	(2.249,3.381)	1.725	(1.459,2.040)	0.265	(0.228,0.309)
Lag08	1.096	(0.939,1.279)	1.122	(0.903,1.394)	1.775	(1.472,2.140)	2.770	(2.264,3.389)	1.798	(1.514,2.136)	0.230	(0.196,0.270)

Supplementary Table 46 Relative risks (RRs) and 95% confidence intervals (CIs) of coronary heart disease hospitalizations associated with extremely low concentrations of each pollutant in Henan Province from 2016 to 2021(the maximum lag period = 9)

Lag days	CO		$PM_{2.5}$		PM_{10}		SO_2		NO_2		O_3	
	RR	95% CI	RR	95% CI	RR	95% CI	RR	95% CI	RR	95% CI	RR	95% CI
lag0	0.993	(0.977,1.009)	0.995	(0.984,1.006)	0.986	(0.972,1.000)	1.002	(0.988,1.015)	0.999	(0.976,1.022)	1.097	(1.062,1.134)
lag1	0.996	(0.986,1.006)	0.996	(0.989,1.004)	0.984	(0.974,0.994)	0.993	(0.985,1.002)	0.986	(0.971,1.001)	1.104	(1.080,1.129)
lag2	0.999	(0.992,1.006)	0.998	(0.992,1.003)	0.982	(0.975,0.990)	0.986	(0.980,0.991)	0.974	(0.963,0.985)	1.111	(1.093,1.129)
lag3	1.001	(0.993,1.008)	0.999	(0.992,1.005)	0.981	(0.973,0.990)	0.980	(0.973,0.986)	0.965	(0.953,0.977)	1.116	(1.096,1.135)
lag4	1.002	(0.992,1.011)	0.999	(0.992,1.007)	0.980	(0.971,0.990)	0.976	(0.968,0.984)	0.959	(0.945,0.974)	1.118	(1.095,1.140)
lag5	1.002	(0.992,1.011)	1.000	(0.992,1.007)	0.980	(0.971,0.990)	0.975	(0.968,0.983)	0.959	(0.944,0.973)	1.116	(1.093,1.139)
Lag6	1.000	(0.992,1.008)	0.999	(0.993,1.006)	0.981	(0.973,0.990)	0.978	(0.971,0.984)	0.963	(0.950,0.975)	1.111	(1.091,1.131)
Lag7	0.998	(0.991,1.005)	0.998	(0.993,1.004)	0.983	(0.975,0.990)	0.983	(0.977,0.988)	0.970	(0.959,0.982)	1.103	(1.085,1.121)
Lag8	0.996	(0.986,1.005)	0.997	(0.990,1.004)	0.984	(0.975,0.994)	0.989	(0.981,0.997)	0.980	(0.966,0.995)	1.093	(1.072,1.115)
Lag9	0.993	(0.978,1.008)	0.996	(0.986,1.006)	0.986	(0.972,1.000)	0.996	(0.984,1.009)	0.991	(0.970,1.013)	1.083	(1.052,1.114)
Lag01	0.989	(0.964,1.015)	0.991	(0.973,1.009)	0.970	(0.947,0.993)	0.995	(0.973,1.017)	0.984	(0.948,1.022)	1.212	(1.148,1.279)
Lag02	0.988	(0.957,1.019)	0.988	(0.967,1.011)	0.953	(0.925,0.982)	0.980	(0.955,1.006)	0.959	(0.915,1.004)	1.346	(1.259,1.439)
Lag03	0.988	(0.956,1.021)	0.987	(0.963,1.012)	0.935	(0.903,0.967)	0.960	(0.934,0.987)	0.925	(0.879,0.974)	1.502	(1.394,1.617)
Lag04	0.990	(0.956,1.024)	0.987	(0.959,1.015)	0.916	(0.882,0.952)	0.937	(0.911,0.964)	0.888	(0.838,0.940)	1.678	(1.548,1.820)
Lag05	0.991	(0.956,1.028)	0.986	(0.956,1.018)	0.899	(0.861,0.938)	0.914	(0.887,0.941)	0.851	(0.799,0.906)	1.873	(1.715,2.046)
Lag06	0.992	(0.955,1.029)	0.985	(0.952,1.020)	0.882	(0.841,0.925)	0.893	(0.867,0.921)	0.819	(0.765,0.878)	2.081	(1.891,2.289)
Lag07	0.990	(0.954,1.027)	0.984	(0.949,1.020)	0.866	(0.824,0.911)	0.878	(0.852,0.905)	0.795	(0.739,0.854)	2.294	(2.075,2.537)
Lag08	0.985	(0.952,1.020)	0.981	(0.945,1.018)	0.853	(0.809,0.898)	0.868	(0.844,0.893)	0.779	(0.724,0.838)	2.508	(2.264,2.779)
Lag09	0.978	(0.946,1.011)	0.977	(0.941,1.015)	0.841	(0.796,0.887)	0.865	(0.841,0.890)	0.772	(0.716,0.833)	2.715	(2.443,3.018)

Supplementary Table 47 Relative risks (RRs) and 95% confidence intervals (CIs) of coronary heart disease hospitalizations associated with extremely high concentrations of each pollutant in Henan Province from 2016 to 2021(the maximum lag period = 9)

Lag days	CO		$PM_{2.5}$		PM_{10}		SO_2		NO_2		O_3	
	RR	95% CI	RR	95% CI	RR	95% CI	RR	95% CI	RR	95% CI	RR	95% CI
lag0	1.033	(0.959,1.113)	1.032	(0.968,1.100)	1.053	(1.000,1.109)	0.989	(0.897,1.090)	1.003	(0.951,1.058)	0.867	(0.824,0.912)
lag1	1.019	(0.972,1.069)	1.023	(0.979,1.068)	1.060	(1.023,1.098)	1.051	(0.989,1.117)	1.034	(0.998,1.072)	0.858	(0.829,0.887)
lag2	1.007	(0.975,1.040)	1.014	(0.981,1.049)	1.066	(1.036,1.097)	1.111	(1.067,1.157)	1.063	(1.035,1.093)	0.850	(0.829,0.872)
lag3	0.998	(0.963,1.034)	1.008	(0.971,1.046)	1.071	(1.038,1.104)	1.161	(1.109,1.215)	1.087	(1.055,1.120)	0.844	(0.822,0.867)
lag4	0.992	(0.950,1.037)	1.004	(0.961,1.048)	1.073	(1.036,1.112)	1.192	(1.127,1.262)	1.101	(1.063,1.141)	0.842	(0.816,0.869)
lag5	0.993	(0.950,1.037)	1.003	(0.960,1.047)	1.073	(1.036,1.112)	1.198	(1.131,1.268)	1.103	(1.065,1.143)	0.844	(0.817,0.871)
Lag6	0.998	(0.962,1.036)	1.005	(0.968,1.044)	1.070	(1.038,1.104)	1.176	(1.121,1.234)	1.093	(1.060,1.127)	0.850	(0.826,0.874)
Lag7	1.008	(0.976,1.042)	1.010	(0.977,1.044)	1.065	(1.036,1.096)	1.135	(1.089,1.183)	1.073	(1.044,1.103)	0.860	(0.838,0.882)
Lag8	1.021	(0.976,1.068)	1.016	(0.976,1.059)	1.059	(1.023,1.096)	1.084	(1.023,1.147)	1.048	(1.013,1.084)	0.871	(0.845,0.898)
Lag9	1.035	(0.965,1.111)	1.024	(0.964,1.087)	1.052	(1.000,1.107)	1.028	(0.940,1.125)	1.020	(0.970,1.073)	0.885	(0.846,0.924)
Lag01	1.053	(0.933,1.188)	1.055	(0.949,1.173)	1.116	(1.025,1.216)	1.039	(0.888,1.215)	1.038	(0.950,1.133)	0.743	(0.684,0.808)
Lag02	1.060	(0.918,1.225)	1.070	(0.939,1.220)	1.190	(1.069,1.324)	1.154	(0.959,1.389)	1.103	(0.990,1.229)	0.632	(0.570,0.700)
Lag03	1.058	(0.907,1.234)	1.078	(0.930,1.250)	1.274	(1.127,1.440)	1.340	(1.102,1.629)	1.199	(1.063,1.353)	0.533	(0.475,0.598)
Lag04	1.050	(0.894,1.233)	1.082	(0.918,1.276)	1.367	(1.191,1.569)	1.598	(1.306,1.954)	1.321	(1.156,1.509)	0.449	(0.396,0.509)
Lag05	1.042	(0.880,1.234)	1.085	(0.903,1.304)	1.468	(1.257,1.713)	1.913	(1.549,2.363)	1.457	(1.258,1.689)	0.379	(0.330,0.434)
Lag06	1.040	(0.873,1.240)	1.091	(0.892,1.334)	1.571	(1.325,1.863)	2.250	(1.806,2.804)	1.593	(1.356,1.871)	0.322	(0.278,0.373)
Lag07	1.049	(0.883,1.247)	1.101	(0.890,1.362)	1.673	(1.396,2.005)	2.555	(2.052,3.180)	1.710	(1.444,2.024)	0.277	(0.237,0.323)
Lag08	1.071	(0.912,1.259)	1.119	(0.901,1.391)	1.772	(1.470,2.135)	2.768	(2.250,3.405)	1.792	(1.509,2.127)	0.241	(0.206,0.283)
Lag09	1.109	(0.948,1.297)	1.146	(0.917,1.431)	1.864	(1.535,2.262)	2.846	(2.319,3.493)	1.828	(1.532,2.180)	0.213	(0.181,0.251)

Supplementary Table 48 Relative risks (RRs) and 95% confidence intervals (CIs) of coronary heart disease hospitalizations associated with extremely low concentrations of each pollutant in Henan Province from 2016 to 2021(df of relative humidity = 2)

Lag days	CO		$PM_{2.5}$		PM_{10}		SO_2		NO_2		O_3	
	RR	95% CI	RR	95% CI	RR	95% CI	RR	95% CI	RR	95% CI	RR	95% CI
lag0	0.991	(0.975,1.007)	0.994	(0.983,1.005)	0.985	(0.970,0.999)	1.005	(0.991,1.019)	0.997	(0.974,1.020)	1.111	(1.074,1.149)
lag1	0.995	(0.985,1.004)	0.996	(0.988,1.003)	0.982	(0.973,0.992)	0.994	(0.985,1.002)	0.984	(0.970,0.999)	1.123	(1.099,1.147)
lag2	0.998	(0.990,1.006)	0.997	(0.991,1.004)	0.980	(0.971,0.989)	0.984	(0.978,0.990)	0.973	(0.960,0.986)	1.132	(1.111,1.153)
lag3	1.000	(0.991,1.010)	0.999	(0.991,1.006)	0.979	(0.968,0.989)	0.977	(0.969,0.985)	0.965	(0.949,0.980)	1.135	(1.110,1.160)
lag4	1.001	(0.991,1.011)	0.999	(0.991,1.007)	0.979	(0.968,0.989)	0.973	(0.965,0.981)	0.960	(0.944,0.976)	1.130	(1.105,1.156)
lag5	1.001	(0.992,1.009)	1.000	(0.993,1.007)	0.980	(0.971,0.989)	0.973	(0.966,0.980)	0.959	(0.945,0.972)	1.117	(1.096,1.139)
Lag6	0.999	(0.990,1.008)	1.000	(0.992,1.007)	0.982	(0.972,0.991)	0.975	(0.967,0.983)	0.960	(0.946,0.974)	1.099	(1.077,1.121)
Lag7	0.996	(0.982,1.011)	0.999	(0.989,1.009)	0.984	(0.970,0.999)	0.979	(0.966,0.991)	0.963	(0.942,0.984)	1.078	(1.047,1.110)
Lag01	0.986	(0.961,1.011)	0.990	(0.972,1.007)	0.967	(0.945,0.990)	0.998	(0.977,1.020)	0.981	(0.946,1.018)	1.247	(1.181,1.316)
Lag02	0.984	(0.956,1.013)	0.987	(0.966,1.009)	0.948	(0.921,0.976)	0.982	(0.958,1.007)	0.955	(0.913,0.999)	1.411	(1.322,1.506)
Lag03	0.985	(0.954,1.016)	0.986	(0.961,1.011)	0.928	(0.896,0.960)	0.959	(0.934,0.985)	0.921	(0.875,0.970)	1.602	(1.488,1.724)
Lag04	0.986	(0.953,1.019)	0.985	(0.956,1.014)	0.908	(0.872,0.946)	0.933	(0.907,0.960)	0.884	(0.833,0.938)	1.810	(1.666,1.967)
Lag05	0.986	(0.953,1.021)	0.985	(0.953,1.017)	0.890	(0.850,0.931)	0.908	(0.882,0.935)	0.848	(0.794,0.905)	2.022	(1.846,2.214)
Lag06	0.985	(0.953,1.019)	0.984	(0.951,1.019)	0.873	(0.832,0.916)	0.886	(0.861,0.911)	0.814	(0.760,0.872)	2.221	(2.019,2.444)
Lag07	0.982	(0.950,1.014)	0.983	(0.948,1.020)	0.859	(0.817,0.904)	0.867	(0.843,0.891)	0.783	(0.729,0.842)	2.394	(2.166,2.647)

Supplementary Table 49 Relative risks (RRs) and 95% confidence intervals (CIs) of coronary heart disease hospitalizations associated with extremely high concentrations of each pollutant in Henan Province from 2016 to 2021(df of relative humidity = 2)

Lag days	CO		$PM_{2.5}$		PM_{10}		SO_2		NO_2		O_3	
	RR	95% CI	RR	95% CI	RR	95% CI	RR	95% CI	RR	95% CI	RR	95% CI
lag0	1.043	(0.967,1.126)	1.037	(0.972,1.106)	1.057	(1.003,1.113)	0.968	(0.876,1.070)	1.007	(0.955,1.063)	0.850	(0.807,0.896)
lag1	1.024	(0.979,1.072)	1.026	(0.983,1.070)	1.067	(1.030,1.104)	1.048	(0.987,1.111)	1.037	(1.002,1.074)	0.836	(0.809,0.865)
lag2	1.009	(0.973,1.046)	1.016	(0.977,1.056)	1.075	(1.040,1.111)	1.124	(1.072,1.178)	1.065	(1.033,1.099)	0.826	(0.803,0.850)
lag3	0.998	(0.954,1.043)	1.008	(0.963,1.055)	1.080	(1.039,1.123)	1.184	(1.118,1.254)	1.088	(1.048,1.129)	0.822	(0.795,0.850)
lag4	0.994	(0.950,1.041)	1.004	(0.958,1.051)	1.081	(1.039,1.124)	1.217	(1.147,1.290)	1.100	(1.060,1.143)	0.828	(0.799,0.857)
lag5	0.998	(0.960,1.036)	1.002	(0.962,1.044)	1.076	(1.041,1.113)	1.218	(1.159,1.280)	1.104	(1.068,1.140)	0.843	(0.818,0.868)
Lag6	1.006	(0.964,1.050)	1.003	(0.962,1.045)	1.069	(1.032,1.107)	1.198	(1.132,1.269)	1.100	(1.063,1.138)	0.865	(0.838,0.892)
Lag7	1.017	(0.948,1.090)	1.005	(0.947,1.066)	1.060	(1.005,1.117)	1.169	(1.065,1.282)	1.093	(1.040,1.150)	0.890	(0.851,0.932)
Lag01	1.069	(0.950,1.203)	1.063	(0.958,1.180)	1.127	(1.036,1.226)	1.014	(0.868,1.185)	1.045	(0.959,1.138)	0.711	(0.654,0.773)
Lag02	1.078	(0.940,1.235)	1.080	(0.950,1.228)	1.211	(1.091,1.345)	1.140	(0.952,1.364)	1.113	(1.002,1.236)	0.587	(0.531,0.649)
Lag03	1.075	(0.929,1.245)	1.089	(0.938,1.264)	1.309	(1.156,1.482)	1.350	(1.114,1.635)	1.211	(1.073,1.366)	0.483	(0.431,0.541)
Lag04	1.069	(0.915,1.250)	1.093	(0.920,1.298)	1.414	(1.223,1.636)	1.642	(1.341,2.012)	1.332	(1.160,1.530)	0.399	(0.351,0.454)
Lag05	1.066	(0.908,1.253)	1.095	(0.905,1.326)	1.522	(1.293,1.791)	2.001	(1.625,2.463)	1.470	(1.262,1.713)	0.337	(0.292,0.387)
Lag06	1.073	(0.918,1.254)	1.098	(0.897,1.345)	1.626	(1.368,1.934)	2.398	(1.963,2.929)	1.617	(1.377,1.899)	0.291	(0.251,0.337)
Lag07	1.090	(0.936,1.271)	1.104	(0.892,1.364)	1.723	(1.436,2.067)	2.803	(2.299,3.417)	1.768	(1.495,2.091)	0.259	(0.222,0.303)

Supplementary Table 50 Relative risks (RRs) and 95% confidence intervals (CIs) of coronary heart disease hospitalizations associated with extremely low concentrations of each pollutant in Henan Province from 2016 to 2021(df of relative humidity = 4)

Lag days	CO		$PM_{2.5}$		PM_{10}		SO_2		NO_2		O_3	
	RR	95% CI	RR	95% CI	RR	95% CI	RR	95% CI	RR	95% CI	RR	95% CI
lag0	0.991	(0.975,1.007)	0.994	(0.983,1.005)	0.985	(0.970,0.999)	1.004	(0.990,1.018)	0.996	(0.973,1.019)	1.113	(1.076,1.151)
lag1	0.995	(0.985,1.004)	0.996	(0.988,1.003)	0.982	(0.973,0.992)	0.993	(0.985,1.001)	0.984	(0.969,0.998)	1.124	(1.100,1.148)
lag2	0.998	(0.990,1.006)	0.997	(0.991,1.004)	0.980	(0.971,0.989)	0.984	(0.977,0.990)	0.973	(0.960,0.986)	1.132	(1.111,1.153)
lag3	1.000	(0.991,1.010)	0.998	(0.991,1.006)	0.978	(0.968,0.989)	0.977	(0.969,0.985)	0.965	(0.950,0.980)	1.135	(1.110,1.160)
lag4	1.001	(0.991,1.011)	0.999	(0.991,1.007)	0.978	(0.968,0.989)	0.973	(0.965,0.981)	0.960	(0.945,0.976)	1.129	(1.104,1.155)
lag5	1.000	(0.992,1.009)	1.000	(0.993,1.007)	0.980	(0.971,0.989)	0.973	(0.966,0.980)	0.959	(0.946,0.973)	1.117	(1.095,1.139)
Lag6	0.999	(0.990,1.008)	1.000	(0.993,1.007)	0.982	(0.972,0.991)	0.975	(0.968,0.983)	0.960	(0.946,0.975)	1.099	(1.077,1.121)
Lag7	0.997	(0.982,1.012)	1.000	(0.989,1.010)	0.984	(0.970,0.999)	0.979	(0.966,0.991)	0.963	(0.942,0.984)	1.079	(1.047,1.111)
Lag01	0.985	(0.961,1.011)	0.990	(0.972,1.007)	0.967	(0.945,0.990)	0.997	(0.975,1.018)	0.979	(0.944,1.016)	1.250	(1.184,1.320)
Lag02	0.983	(0.955,1.013)	0.987	(0.965,1.009)	0.948	(0.920,0.976)	0.980	(0.956,1.005)	0.953	(0.911,0.997)	1.415	(1.326,1.510)
Lag03	0.983	(0.953,1.015)	0.985	(0.961,1.011)	0.927	(0.896,0.960)	0.958	(0.932,0.984)	0.920	(0.873,0.969)	1.605	(1.491,1.728)
Lag04	0.984	(0.952,1.018)	0.985	(0.956,1.014)	0.907	(0.871,0.945)	0.932	(0.906,0.959)	0.883	(0.832,0.937)	1.813	(1.669,1.970)
Lag05	0.985	(0.951,1.020)	0.984	(0.952,1.017)	0.889	(0.849,0.930)	0.907	(0.881,0.934)	0.847	(0.793,0.905)	2.025	(1.848,2.217)
Lag06	0.984	(0.951,1.017)	0.984	(0.950,1.018)	0.873	(0.831,0.916)	0.885	(0.860,0.910)	0.814	(0.760,0.872)	2.225	(2.022,2.448)
Lag07	0.981	(0.949,1.013)	0.983	(0.948,1.020)	0.859	(0.816,0.904)	0.866	(0.842,0.890)	0.784	(0.729,0.842)	2.400	(2.171,2.653)

Supplementary Table 51 Relative risks (RRs) and 95% confidence intervals (CIs) of coronary heart disease hospitalizations associated with extremely high concentrations of each pollutant in Henan Province from 2016 to 2021(df of relative humidity = 4)

Lag days	CO		$PM_{2.5}$		PM_{10}		SO_2		NO_2		O_3	
	RR	95% CI	RR	95% CI	RR	95% CI	RR	95% CI	RR	95% CI	RR	95% CI
lag0	1.044	(0.967,1.127)	1.036	(0.971,1.106)	1.057	(1.003,1.114)	0.974	(0.881,1.077)	1.010	(0.957,1.066)	0.848	(0.804,0.893)
lag1	1.026	(0.981,1.073)	1.026	(0.983,1.070)	1.067	(1.031,1.105)	1.052	(0.991,1.116)	1.039	(1.004,1.076)	0.835	(0.808,0.863)
lag2	1.010	(0.974,1.048)	1.017	(0.978,1.057)	1.076	(1.041,1.112)	1.126	(1.074,1.180)	1.066	(1.033,1.100)	0.826	(0.803,0.850)
lag3	0.999	(0.956,1.045)	1.009	(0.964,1.056)	1.081	(1.040,1.124)	1.184	(1.118,1.254)	1.087	(1.047,1.128)	0.823	(0.795,0.851)
lag4	0.995	(0.951,1.042)	1.005	(0.959,1.052)	1.081	(1.040,1.124)	1.216	(1.146,1.289)	1.099	(1.058,1.141)	0.828	(0.800,0.858)
lag5	0.998	(0.961,1.037)	1.002	(0.963,1.044)	1.076	(1.041,1.113)	1.217	(1.158,1.279)	1.102	(1.066,1.139)	0.843	(0.818,0.869)
Lag6	1.005	(0.963,1.049)	1.002	(0.962,1.045)	1.068	(1.031,1.106)	1.197	(1.130,1.268)	1.099	(1.062,1.137)	0.864	(0.838,0.892)
Lag7	1.015	(0.947,1.088)	1.003	(0.945,1.064)	1.058	(1.004,1.115)	1.168	(1.065,1.282)	1.092	(1.038,1.149)	0.889	(0.850,0.931)
Lag01	1.071	(0.951,1.205)	1.063	(0.958,1.180)	1.128	(1.037,1.227)	1.024	(0.877,1.197)	1.050	(0.963,1.144)	0.708	(0.651,0.770)
Lag02	1.082	(0.943,1.240)	1.081	(0.951,1.229)	1.213	(1.092,1.348)	1.153	(0.963,1.380)	1.119	(1.007,1.242)	0.585	(0.529,0.647)
Lag03	1.081	(0.934,1.251)	1.091	(0.940,1.266)	1.312	(1.158,1.486)	1.366	(1.127,1.655)	1.216	(1.077,1.372)	0.481	(0.429,0.539)
Lag04	1.076	(0.920,1.258)	1.096	(0.923,1.301)	1.418	(1.226,1.641)	1.660	(1.355,2.034)	1.336	(1.163,1.534)	0.398	(0.350,0.453)
Lag05	1.074	(0.914,1.262)	1.098	(0.907,1.330)	1.527	(1.297,1.797)	2.020	(1.640,2.487)	1.472	(1.263,1.716)	0.336	(0.292,0.387)
Lag06	1.080	(0.924,1.262)	1.101	(0.899,1.348)	1.631	(1.371,1.939)	2.418	(1.979,2.954)	1.617	(1.377,1.900)	0.290	(0.250,0.337)
Lag07	1.096	(0.940,1.277)	1.104	(0.893,1.365)	1.725	(1.438,2.070)	2.824	(2.316,3.444)	1.766	(1.493,2.090)	0.258	(0.221,0.302)

Supplementary Table 52 Relative risks (RRs) and 95% confidence intervals (CIs) of coronary heart disease hospitalizations associated with extremely low concentrations of each pollutant in Henan Province from 2016 to 2021(df of relative temperature = 2)

Lag days	CO		$PM_{2.5}$		PM_{10}		SO_2		NO_2		O_3	
	RR	95% CI	RR	95% CI	RR	95% CI	RR	95% CI	RR	95% CI	RR	95% CI
lag0	0.991	(0.975,1.007)	0.994	(0.983,1.005)	0.985	(0.970,0.999)	1.004	(0.990,1.018)	0.996	(0.973,1.019)	1.110	(1.073,1.149)
lag1	0.995	(0.985,1.004)	0.996	(0.989,1.003)	0.982	(0.972,0.992)	0.993	(0.985,1.001)	0.984	(0.969,0.998)	1.122	(1.098,1.146)
lag2	0.998	(0.990,1.006)	0.997	(0.991,1.004)	0.980	(0.971,0.989)	0.984	(0.977,0.990)	0.973	(0.960,0.986)	1.131	(1.110,1.152)
lag3	1.000	(0.990,1.010)	0.998	(0.991,1.006)	0.978	(0.968,0.989)	0.977	(0.969,0.985)	0.965	(0.949,0.980)	1.135	(1.110,1.160)
lag4	1.001	(0.991,1.011)	0.999	(0.991,1.007)	0.978	(0.968,0.989)	0.973	(0.965,0.981)	0.960	(0.945,0.976)	1.130	(1.105,1.156)
lag5	1.000	(0.992,1.008)	0.999	(0.993,1.006)	0.980	(0.971,0.989)	0.973	(0.966,0.980)	0.959	(0.946,0.973)	1.117	(1.096,1.139)
Lag6	0.999	(0.990,1.008)	1.000	(0.992,1.007)	0.982	(0.972,0.991)	0.975	(0.968,0.983)	0.960	(0.946,0.975)	1.099	(1.077,1.121)
Lag7	0.997	(0.982,1.012)	0.999	(0.989,1.010)	0.984	(0.970,0.999)	0.979	(0.966,0.991)	0.963	(0.942,0.984)	1.079	(1.048,1.111)
Lag01	0.986	(0.961,1.011)	0.990	(0.972,1.008)	0.967	(0.944,0.990)	0.996	(0.975,1.018)	0.979	(0.944,1.016)	1.246	(1.180,1.315)
Lag02	0.983	(0.955,1.013)	0.987	(0.966,1.009)	0.947	(0.920,0.975)	0.980	(0.956,1.005)	0.953	(0.911,0.997)	1.409	(1.320,1.504)
Lag03	0.983	(0.953,1.015)	0.986	(0.961,1.011)	0.927	(0.895,0.960)	0.957	(0.932,0.983)	0.919	(0.873,0.968)	1.599	(1.485,1.721)
Lag04	0.984	(0.952,1.018)	0.985	(0.956,1.014)	0.907	(0.871,0.944)	0.932	(0.906,0.959)	0.883	(0.832,0.937)	1.807	(1.663,1.963)
Lag05	0.985	(0.951,1.019)	0.984	(0.952,1.017)	0.888	(0.849,0.930)	0.907	(0.881,0.934)	0.847	(0.793,0.904)	2.018	(1.843,2.211)
Lag06	0.983	(0.951,1.017)	0.984	(0.950,1.018)	0.872	(0.831,0.915)	0.885	(0.860,0.910)	0.813	(0.759,0.871)	2.219	(2.016,2.441)
Lag07	0.980	(0.948,1.013)	0.983	(0.948,1.019)	0.859	(0.816,0.903)	0.866	(0.842,0.890)	0.783	(0.728,0.841)	2.393	(2.165,2.645)

Supplementary Table 53 Relative risks (RRs) and 95% confidence intervals (CIs) of coronary heart disease hospitalizations associated with extremely high concentrations of each pollutant in Henan Province from 2016 to 2021(df of relative temperature = 2)

Lag days	CO		$PM_{2.5}$		PM_{10}		SO_2		NO_2		O_3	
	RR	95% CI	RR	95% CI	RR	95% CI	RR	95% CI	RR	95% CI	RR	95% CI
lag0	1.043	(0.967,1.126)	1.035	(0.970,1.104)	1.057	(1.003,1.114)	0.975	(0.882,1.078)	1.010	(0.957,1.066)	0.851	(0.807,0.896)
lag1	1.026	(0.980,1.073)	1.025	(0.983,1.070)	1.067	(1.031,1.105)	1.052	(0.992,1.116)	1.039	(1.004,1.076)	0.837	(0.810,0.865)
lag2	1.010	(0.974,1.048)	1.017	(0.978,1.057)	1.076	(1.041,1.112)	1.126	(1.074,1.180)	1.066	(1.033,1.100)	0.827	(0.803,0.851)
lag3	1.000	(0.956,1.046)	1.010	(0.965,1.057)	1.081	(1.040,1.124)	1.184	(1.118,1.254)	1.087	(1.048,1.129)	0.823	(0.795,0.851)
lag4	0.996	(0.952,1.043)	1.005	(0.959,1.053)	1.081	(1.040,1.125)	1.215	(1.146,1.289)	1.100	(1.059,1.142)	0.828	(0.800,0.857)
lag5	0.999	(0.961,1.038)	1.003	(0.963,1.045)	1.076	(1.041,1.113)	1.216	(1.157,1.278)	1.102	(1.067,1.139)	0.842	(0.817,0.868)
Lag6	1.006	(0.964,1.050)	1.003	(0.962,1.045)	1.068	(1.031,1.106)	1.197	(1.130,1.268)	1.099	(1.062,1.137)	0.864	(0.838,0.891)
Lag7	1.016	(0.947,1.089)	1.003	(0.945,1.065)	1.058	(1.004,1.115)	1.168	(1.065,1.282)	1.092	(1.039,1.149)	0.889	(0.850,0.931)
Lag01	1.070	(0.951,1.204)	1.061	(0.956,1.178)	1.129	(1.037,1.228)	1.026	(0.878,1.199)	1.050	(0.963,1.144)	0.712	(0.655,0.774)
Lag02	1.081	(0.943,1.239)	1.079	(0.949,1.226)	1.215	(1.093,1.349)	1.155	(0.964,1.382)	1.119	(1.008,1.243)	0.588	(0.532,0.651)
Lag03	1.081	(0.934,1.251)	1.089	(0.938,1.264)	1.313	(1.160,1.487)	1.367	(1.128,1.657)	1.217	(1.078,1.373)	0.484	(0.432,0.542)
Lag04	1.077	(0.921,1.259)	1.095	(0.922,1.300)	1.420	(1.228,1.643)	1.661	(1.356,2.036)	1.338	(1.165,1.537)	0.401	(0.352,0.455)
Lag05	1.075	(0.915,1.264)	1.099	(0.907,1.330)	1.529	(1.299,1.799)	2.021	(1.641,2.488)	1.475	(1.266,1.719)	0.338	(0.293,0.389)
Lag06	1.082	(0.926,1.265)	1.102	(0.899,1.349)	1.633	(1.373,1.941)	2.419	(1.980,2.955)	1.621	(1.380,1.904)	0.292	(0.252,0.338)
Lag07	1.099	(0.943,1.280)	1.105	(0.894,1.367)	1.727	(1.440,2.072)	2.825	(2.317,3.445)	1.771	(1.497,2.094)	0.259	(0.222,0.303)

Supplementary Table 54 Relative risks (RRs) and 95% confidence intervals (CIs) of coronary heart disease hospitalizations associated with extremely low concentrations of each pollutant in Henan Province from 2016 to 2021(df of relative temperature = 4)

Lag days	CO		$PM_{2.5}$		PM_{10}		SO_2		NO_2		O_3	
	RR	95% CI	RR	95% CI	RR	95% CI	RR	95% CI	RR	95% CI	RR	95% CI
lag0	0.991	(0.975,1.007)	0.994	(0.983,1.005)	0.985	(0.970,0.999)	1.004	(0.990,1.018)	0.996	(0.973,1.019)	1.111	(1.074,1.150)
lag1	0.995	(0.985,1.004)	0.996	(0.988,1.003)	0.982	(0.972,0.992)	0.993	(0.985,1.001)	0.984	(0.969,0.998)	1.123	(1.099,1.147)
lag2	0.998	(0.990,1.006)	0.997	(0.991,1.004)	0.980	(0.971,0.989)	0.984	(0.977,0.990)	0.973	(0.960,0.986)	1.131	(1.111,1.152)
lag3	1.000	(0.991,1.010)	0.998	(0.991,1.006)	0.978	(0.968,0.989)	0.977	(0.969,0.985)	0.965	(0.950,0.980)	1.135	(1.110,1.160)
lag4	1.001	(0.991,1.011)	0.999	(0.991,1.007)	0.978	(0.968,0.989)	0.973	(0.965,0.981)	0.960	(0.945,0.976)	1.130	(1.105,1.156)
lag5	1.000	(0.992,1.009)	1.000	(0.993,1.006)	0.980	(0.971,0.989)	0.973	(0.966,0.980)	0.959	(0.946,0.973)	1.117	(1.095,1.139)
Lag6	0.999	(0.990,1.008)	1.000	(0.993,1.007)	0.982	(0.972,0.991)	0.975	(0.968,0.983)	0.961	(0.947,0.975)	1.099	(1.077,1.121)
Lag7	0.997	(0.982,1.012)	0.999	(0.989,1.010)	0.984	(0.970,0.999)	0.979	(0.966,0.991)	0.963	(0.942,0.984)	1.079	(1.047,1.111)
Lag01	0.985	(0.961,1.011)	0.990	(0.972,1.007)	0.967	(0.944,0.990)	0.997	(0.975,1.018)	0.979	(0.944,1.016)	1.248	(1.182,1.317)
Lag02	0.983	(0.955,1.012)	0.987	(0.965,1.009)	0.947	(0.920,0.975)	0.980	(0.956,1.005)	0.953	(0.911,0.997)	1.411	(1.323,1.506)
Lag03	0.983	(0.953,1.015)	0.985	(0.960,1.011)	0.927	(0.895,0.960)	0.958	(0.932,0.984)	0.919	(0.873,0.968)	1.602	(1.488,1.724)
Lag04	0.984	(0.952,1.018)	0.984	(0.956,1.014)	0.907	(0.871,0.944)	0.932	(0.906,0.959)	0.883	(0.832,0.937)	1.809	(1.665,1.966)
Lag05	0.985	(0.951,1.019)	0.984	(0.952,1.017)	0.888	(0.849,0.930)	0.907	(0.881,0.934)	0.847	(0.793,0.904)	2.021	(1.846,2.214)
Lag06	0.984	(0.951,1.017)	0.983	(0.950,1.018)	0.872	(0.831,0.915)	0.885	(0.860,0.910)	0.813	(0.759,0.871)	2.221	(2.019,2.445)
Lag07	0.980	(0.949,1.013)	0.983	(0.948,1.019)	0.859	(0.816,0.903)	0.866	(0.842,0.890)	0.783	(0.729,0.842)	2.396	(2.168,2.649)

Supplementary Table 55 Relative risks (RRs) and 95% confidence intervals (CIs) of coronary heart disease hospitalizations associated with extremely high concentrations of each pollutant in Henan Province from 2016 to 2021(df of relative temperature = 4)

Lag days	CO		$PM_{2.5}$		PM_{10}		SO_2		NO_2		O_3	
	RR	95% CI	RR	95% CI	RR	95% CI	RR	95% CI	RR	95% CI	RR	95%CI
lag0	1.044	(0.968,1.127)	1.037	(0.971,1.106)	1.057	(1.003,1.114)	0.975	(0.882,1.077)	1.010	(0.957,1.066)	0.849	(0.806,0.895)
lag1	1.026	(0.981,1.073)	1.026	(0.984,1.071)	1.067	(1.031,1.105)	1.052	(0.991,1.116)	1.039	(1.004,1.076)	0.836	(0.809,0.864)
lag2	1.010	(0.974,1.048)	1.017	(0.978,1.057)	1.076	(1.041,1.112)	1.125	(1.074,1.179)	1.066	(1.033,1.100)	0.826	(0.803,0.850)
lag3	0.999	(0.956,1.045)	1.009	(0.964,1.057)	1.081	(1.040,1.124)	1.184	(1.118,1.254)	1.087	(1.047,1.128)	0.823	(0.795,0.851)
lag4	0.995	(0.951,1.042)	1.005	(0.959,1.053)	1.081	(1.040,1.125)	1.215	(1.146,1.289)	1.099	(1.058,1.142)	0.828	(0.800,0.857)
lag5	0.998	(0.961,1.037)	1.003	(0.963,1.044)	1.076	(1.041,1.113)	1.216	(1.157,1.278)	1.102	(1.066,1.139)	0.843	(0.818,0.868)
Lag6	1.005	(0.963,1.049)	1.003	(0.962,1.045)	1.068	(1.031,1.106)	1.197	(1.130,1.268)	1.098	(1.061,1.137)	0.864	(0.838,0.891)
Lag7	1.015	(0.947,1.088)	1.003	(0.945,1.065)	1.058	(1.004,1.115)	1.168	(1.065,1.282)	1.092	(1.038,1.148)	0.889	(0.850,0.931)
Lag01	1.071	(0.952,1.206)	1.064	(0.958,1.180)	1.129	(1.037,1.228)	1.025	(0.877,1.198)	1.050	(0.964,1.144)	0.710	(0.653,0.772)
Lag02	1.082	(0.944,1.241)	1.082	(0.951,1.229)	1.215	(1.093,1.349)	1.153	(0.963,1.381)	1.119	(1.008,1.243)	0.587	(0.531,0.649)
Lag03	1.082	(0.934,1.252)	1.092	(0.940,1.267)	1.313	(1.159,1.487)	1.366	(1.127,1.655)	1.217	(1.078,1.374)	0.483	(0.431,0.541)
Lag04	1.077	(0.921,1.259)	1.097	(0.924,1.303)	1.420	(1.227,1.643)	1.659	(1.354,2.034)	1.338	(1.165,1.536)	0.400	(0.352,0.454)
Lag05	1.075	(0.914,1.263)	1.100	(0.908,1.332)	1.528	(1.298,1.799)	2.018	(1.639,2.485)	1.474	(1.265,1.718)	0.337	(0.293,0.388)
Lag06	1.080	(0.924,1.263)	1.102	(0.900,1.350)	1.633	(1.373,1.941)	2.416	(1.977,2.951)	1.619	(1.379,1.902)	0.291	(0.251,0.337)
Lag07	1.097	(0.941,1.278)	1.106	(0.894,1.368)	1.728	(1.440,2.073)	2.822	(2.314,3.441)	1.768	(1.495,2.091)	0.259	(0.222,0.302)

Supplementary Table 56 Interaction analysis between various air pollutants on coronary heart disease hospitalizations in Henan Province from 2016 to 2021(the maximum lag period = 5)

pollutant	CO	NO_2	SO_2	O_3	PM_{10}	$PM_{2.5}$
CO		**IRR:1.113(1.051,1.175)***<br>**RERI:0.094(0.048,0.141)***	**IRR:1.245(1.176,1.314)***<br>**RERI:0.196(0.153,0.239)***	IRR:0.960(0.910,1.010)<br>RERI:-0.037(-0.089,0.016)	**IRR:1.065(1.007,1.123)***<br>**RERI:0.060(0.019,0.101)***	IRR:1.006(0.949,1.063)<br>RERI:0.004(-0.037,0.045)
NO_2	**IRR:1.113(1.051,1.174)***<br>**RERI:0.091(0.047,0.135)***		IRR:1.026(0.969,1.083)<br>RERI:0.015(-0.029,0.059)	IRR:0.983(0.930,1.036)<br>RERI:-0.017(-0.069,0.035)	IRR:1.034(0.975,1.093)<br>RERI:0.031(-0.010,0.072)	IRR:1.034(0.974,1.094)<br>RERI:0.032(-0.008,0.073)
SO_2	**IRR:1.243(1.173,1.312)***<br>**RERI:0.196(0.151,0.241)***	IRR:1.037(0.979,1.094)<br>RERI:0.028(-0.019,0.075)		IRR:0.996(0.943,1.049)<br>RERI:-0.001(-0.055,0.052)	IRR:1.049(0.994,1.103)<br>**RERI:0.047(0.003,0.092)***	IRR:1.023(0.971,1.075)<br>RERI:0.021(-0.024,0.066)
O_3	**IRR:0.939(0.889,0.989)***<br>RERI:-0.044(-0.096,0.008)	IRR:0.954(0.902,1.006)<br>RERI:-0.046(-0.100,0.007)	IRR:0.975(0.923,1.027)<br>RERI:-0.009(-0.060,0.043)		**IRR:0.934(0.885,0.983)***<br>**RERI:-0.061(-0.111,-0.012)***	IRR:0.951(0.901,1.002)<br>RERI:-0.047(-0.097,0.002)
PM_{10}	**IRR:1.060(1.002,1.118)***<br>**RERI:0.054(0.012,0.095)***	IRR:1.025(0.966,1.083)<br>RERI:0.023(-0.021,0.067)	IRR:1.041(0.987,1.095)<br>RERI:0.037(-0.006,0.080)	IRR:0.954(0.904,1.003)<br>RERI:-0.046(-0.097,0.004)		IRR:1.068(0.994,1.143)<br>**RERI:0.064(0.028,0.100)***
$PM_{2.5}$	IRR:1.004(0.948,1.061)<br>RERI:0.002(-0.040,0.043)	IRR:1.027(0.967,1.086)<br>RERI:0.026(-0.018,0.070)	IRR:1.021(0.969,1.073)<br>RERI:0.017(-0.028,0.062)	IRR:0.977(0.925,1.028)<br>RERI:-0.023(-0.075,0.028)	IRR:1.072(0.997,1.146)<br>**RERI:0.066(0.029,0.103)***	

Supplementary Table 57 Interaction analysis between various air pollutants on coronary heart disease hospitalizations in Henan Province from 2016 to 2021(the maximum lag period = 6)

pollutant	CO	NO_2	SO_2	O_3	PM_{10}	$PM_{2.5}$
CO		**IRR:1.114(1.053,1.176)*** **RERI:0.095(0.050,0.141)***	**IRR:1.246(1.177,1.315)*** **RERI:0.197(0.154,0.240)***	IRR:0.961(0.910,1.011) RERI:-0.035(-0.087,0.017)	**IRR:1.065(1.007,1.124)*** **RERI:0.060(0.020,0.101)***	IRR:1.006(0.949,1.063) RERI:0.004(-0.037,0.045)
NO_2	**IRR:1.115(1.053,1.176)*** **RERI:0.092(0.049,0.136)***		IRR:1.025(0.968,1.082) RERI:0.014(-0.029,0.057)	IRR:0.982(0.929,1.035) RERI:-0.018(-0.070,0.034)	IRR:1.035(0.976,1.094) RERI:0.032(-0.009,0.072)	IRR:1.034(0.974,1.094) RERI:0.032(-0.008,0.073)
SO_2	**IRR:1.244(1.175,1.314)*** **RERI:0.197(0.153,0.242)***	IRR:1.038(0.980,1.096) RERI:0.029(-0.017,0.075)		IRR:0.997(0.944,1.049) RERI:0.001(-0.052,0.054)	IRR:1.050(0.996,1.104) **RERI:0.049(0.005,0.092)***	IRR:1.023(0.971,1.075) RERI:0.021(-0.024,0.066)
O_3	**IRR:0.939(0.889,0.989)*** RERI:-0.045(-0.096,0.007)	IRR:0.953(0.902,1.005) RERI:-0.047(-0.100,0.006)	IRR:0.975(0.923,1.026) RERI:-0.009(-0.061,0.042)		**IRR:0.934(0.885,0.983)*** **RERI:-0.061(-0.111,-0.012)***	IRR:0.951(0.901,1.002) RERI:-0.047(-0.097,0.002)
PM_{10}	**IRR:1.062(1.004,1.120)*** **RERI:0.055(0.013,0.096)***	IRR:1.027(0.968,1.086) RERI:0.026(-0.017,0.069)	IRR:1.041(0.987,1.094) RERI:0.036(-0.006,0.079)	IRR:0.953(0.904,1.003) RERI:-0.046(-0.096,0.004)		IRR:1.068(0.994,1.143) **RERI:0.064(0.028,0.100)***
$PM_{2.5}$	IRR:1.006(0.950,1.063) RERI:0.003(-0.039,0.045)	IRR:1.029(0.969,1.088) RERI:0.028(-0.015,0.071)	IRR:1.021(0.969,1.073) RERI:0.017(-0.028,0.061)	IRR:0.973(0.922,1.025) RERI:-0.027(-0.078,0.024)	IRR:1.072(0.997,1.146) **RERI:0.066(0.029,0.103)***	

Supplementary Table 58 Interaction analysis between various air pollutants on coronary heart disease hospitalizations in Henan Province from 2016 to 2021(the maximum lag period = 7)

pollutant	CO	NO_2	SO_2	O_3	PM_{10}	$PM_{2.5}$
CO		**IRR:1.115(1.053,1.177)*** **RERI:0.096(0.051,0.141)***	**IRR:1.247(1.178,1.317)*** **RERI:0.198(0.155,0.241)***	IRR:0.962(0.912,1.012) RERI:-0.033(-0.085,0.019)	**IRR:1.065(1.007,1.123)*** **RERI:0.060(0.019,0.100)***	IRR:1.007(0.950,1.063) RERI:0.005(-0.036,0.046)
NO_2	**IRR:1.116(1.054,1.178)*** **RERI:0.093(0.050,0.137)***		IRR:1.024(0.967,1.081) RERI:0.013(-0.030,0.056)	IRR:0.985(0.932,1.038) RERI:-0.016(-0.067,0.036)	IRR:1.032(0.973,1.091) RERI:0.029(-0.011,0.069)	IRR:1.034(0.974,1.094) RERI:0.033(-0.008,0.073)
SO_2	**IRR:1.243(1.174,1.312)*** **RERI:0.196(0.152,0.241)***	IRR:1.037(0.979,1.094) RERI:0.028(-0.018,0.074)		IRR:0.998(0.946,1.051) RERI:0.002(-0.050,0.055)	IRR:1.051(0.996,1.105) **RERI:0.049(0.005,0.093)***	IRR:1.023(0.971,1.075) RERI:0.021(-0.024,0.066)
O_3	**IRR:0.938(0.888,0.987)*** RERI:-0.046(-0.098,0.006)	IRR:0.956(0.904,1.007) RERI:-0.045(-0.098,0.008)	IRR:0.976(0.924,1.028) RERI:-0.008(-0.059,0.043)		**IRR:0.934(0.885,0.983)*** **RERI:-0.062(-0.111,-0.012)***	IRR:0.950(0.900,1.001) RERI:-0.048(-0.097,0.002)
PM_{10}	**IRR:1.063(1.005,1.121)*** **RERI:0.055(0.014,0.097)***	IRR:1.024(0.966,1.083) RERI:0.023(-0.020,0.066)	IRR:1.040(0.987,1.094) RERI:0.036(-0.006,0.079)	IRR:0.955(0.905,1.004) RERI:-0.045(-0.095,0.005)		IRR:1.068(0.994,1.142) **RERI:0.063(0.027,0.099)***
$PM_{2.5}$	IRR:1.008(0.951,1.065) RERI:0.004(-0.037,0.046)	IRR:1.028(0.968,1.087) RERI:0.027(-0.016,0.070)	IRR:1.021(0.969,1.073) RERI:0.017(-0.028,0.061)	IRR:0.972(0.921,1.023) RERI:-0.028(-0.078,0.023)	IRR:1.072(0.998,1.147) **RERI:0.067(0.030,0.103)***	

Supplementary Table 59 Interaction analysis between various air pollutants on coronary heart disease hospitalizations in Henan Province from 2016 to 2021(the maximum lag period = 8)

pollutant	CO	NO_2	SO_2	O_3	PM_{10}	$PM_{2.5}$
CO		**IRR:1.114(1.053,1.176)*** **RERI:0.095(0.050,0.140)***	**IRR:1.247(1.178,1.316)*** **RERI:0.198(0.155,0.240)***	IRR:0.965(0.915,1.016) RERI:-0.031(-0.082,0.021)	**IRR:1.066(1.008,1.124)*** **RERI:0.061(0.020,0.101)***	IRR:1.007(0.951,1.064) RERI:0.005(-0.035,0.046)
NO_2	**IRR:1.117(1.055,1.179)*** **RERI:0.094(0.051,0.137)***		IRR:1.025(0.968,1.082) RERI:0.014(-0.029,0.057)	IRR:0.987(0.934,1.040) RERI:-0.013(-0.065,0.038)	IRR:1.032(0.974,1.091) RERI:0.030(-0.010,0.070)	IRR:1.034(0.974,1.094) RERI:0.033(-0.008,0.073)
SO_2	**IRR:1.243(1.174,1.312)*** **RERI:0.196(0.152,0.241)***	IRR:1.038(0.980,1.095) RERI:0.029(-0.017,0.074)		IRR:1.004(0.951,1.057) RERI:0.007(-0.046,0.060)	IRR:1.051(0.997,1.106) **RERI:0.049(0.006,0.093)***	IRR:1.023(0.971,1.076) RERI:0.021(-0.023,0.066)
O_3	**IRR:0.937(0.887,0.986)*** RERI:-0.047(-0.099,0.005)	IRR:0.955(0.903,1.006) RERI:-0.046(-0.099,0.007)	IRR:0.976(0.924,1.027) RERI:-0.008(-0.060,0.043)		**IRR:0.933(0.884,0.982)*** **RERI:-0.063(-0.112,-0.014)***	IRR:0.950(0.900,1.001) RERI:-0.048(-0.098,0.001)
PM_{10}	**IRR:1.065(1.007,1.123)*** **RERI:0.057(0.016,0.098)***	IRR:1.025(0.966,1.084) RERI:0.024(-0.019,0.066)	IRR:1.041(0.987,1.095) RERI:0.037(-0.006,0.079)	IRR:0.960(0.910,1.010) RERI:-0.039(-0.089,0.011)		IRR:1.068(0.994,1.142) **RERI:0.064(0.028,0.099)***
$PM_{2.5}$	IRR:1.010(0.953,1.066) RERI:0.006(-0.036,0.047)	IRR:1.027(0.967,1.086) RERI:0.026(-0.017,0.068)	IRR:1.022(0.969,1.074) RERI:0.017(-0.027,0.061)	IRR:0.978(0.926,1.029) RERI:-0.022(-0.072,0.029)	IRR:1.073(0.998,1.147) **RERI:0.067(0.031,0.103)***	

Supplementary Table 60 Interaction analysis between various air pollutants on coronary heart disease hospitalizations in Henan Province from 2016 to 2021(the maximum lag period = 9)

pollutant	CO	NO_2	SO_2	O_3	PM_{10}	$PM_{2.5}$
CO		**IRR:1.115(1.053,1.177)***	**IRR:1.246(1.177,1.315)***	IRR:0.964(0.914,1.014)	**IRR:1.066(1.008,1.124)***	IRR:1.008(0.951,1.065)
		**RERI:0.096(0.051,0.140)***	**RERI:0.197(0.154,0.240)***	RERI:-0.032(-0.083,0.020)	**RERI:0.061(0.020,0.101)***	RERI:0.006(-0.035,0.046)
NO_2	**IRR:1.118(1.056,1.180)***		IRR:1.025(0.968,1.081)	IRR:0.982(0.930,1.035)	IRR:1.033(0.974,1.092)	IRR:1.034(0.974,1.094)
	**RERI:0.095(0.051,0.138)***		RERI:0.013(-0.030,0.057)	RERI:-0.018(-0.070,0.033)	RERI:0.030(-0.010,0.070)	RERI:0.033(-0.007,0.073)
SO_2	**IRR:1.243(1.174,1.312)***	IRR:1.038(0.980,1.096)		IRR:1.002(0.950,1.055)	IRR:1.051(0.996,1.105)	IRR:1.024(0.971,1.076)
	**RERI:0.196(0.152,0.240)***	RERI:0.029(-0.017,0.074)		RERI:0.006(-0.046,0.059)	**RERI:0.049(0.006,0.092)***	RERI:0.022(-0.023,0.066)
O_3	**IRR:0.935(0.886,0.985)***	IRR:0.955(0.903,1.006)	IRR:0.976(0.924,1.027)		**IRR:0.932(0.883,0.981)***	IRR:0.950(0.899,1.000)
	RERI:-0.048(-0.100,0.003)	RERI:-0.046(-0.098,0.007)	RERI:-0.008(-0.060,0.043)		**RERI:-0.064(-0.113,-0.015)***	RERI:-0.049(-0.098,0.000)
PM_{10}	**IRR:1.066(1.008,1.124)***	IRR:1.026(0.967,1.084)	IRR:1.040(0.987,1.094)	IRR:0.954(0.904,1.003)		IRR:1.069(0.994,1.143)
	**RERI:0.058(0.017,0.099)***	RERI:0.024(-0.018,0.066)	RERI:0.036(-0.007,0.078)	RERI:-0.046(-0.095,0.004)		**RERI:0.064(0.029,0.100)***
$PM_{2.5}$	IRR:1.011(0.954,1.068)	IRR:1.027(0.967,1.086)	IRR:1.021(0.969,1.074)	IRR:0.970(0.919,1.021)	IRR:1.074(0.999,1.148)	
	RERI:0.007(-0.035,0.048)	RERI:0.026(-0.016,0.068)	RERI:0.017(-0.027,0.061)	RERI:-0.030(-0.080,0.020)	**RERI:0.068(0.032,0.104)***	

Supplementary Table 61 Interaction analysis between various air pollutants on coronary heart disease hospitalizations in Henan Province from 2016 to 2021(df of temperature = 2)

pollutant	CO	NO_2	SO_2	O_3	PM_{10}	$PM_{2.5}$
CO		**IRR:1.114(1.052,1.175)*** **RERI:0.095(0.050,0.140)***	**IRR:1.245(1.176,1.314)*** **RERI:0.197(0.154,0.239)***	IRR:0.963(0.913,1.014) RERI:-0.033(-0.084,0.018)	**IRR:1.063(1.005,1.121)*** **RERI:0.058(0.018,0.099)***	IRR:1.006(0.949,1.063) RERI:0.004(-0.037,0.045)
NO_2	**IRR:1.114(1.053,1.176)*** **RERI:0.092(0.048,0.135)***		IRR:1.025(0.968,1.082) RERI:0.014(-0.029,0.057)	IRR:0.988(0.935,1.041) RERI:-0.013(-0.064,0.039)	IRR:1.031(0.972,1.090) RERI:0.028(-0.012,0.069)	IRR:1.033(0.973,1.093) RERI:0.032(-0.008,0.072)
SO_2	**IRR:1.241(1.172,1.310)*** **RERI:0.195(0.150,0.239)***	IRR:1.038(0.980,1.095) RERI:0.029(-0.017,0.075)		IRR:0.999(0.947,1.052) RERI:0.002(-0.050,0.054)	IRR:1.050(0.996,1.105) **RERI:0.048(0.005,0.092)***	IRR:1.023(0.971,1.076) RERI:0.021(-0.023,0.066)
O_3	**IRR:0.939(0.889,0.988)*** RERI:-0.046(-0.097,0.005)	IRR:0.958(0.907,1.010) RERI:-0.042(-0.095,0.010)	IRR:0.977(0.925,1.028) RERI:-0.009(-0.059,0.042)		**IRR:0.937(0.888,0.986)*** **RERI:-0.059(-0.107,-0.010)***	IRR:0.953(0.902,1.004) RERI:-0.046(-0.094,0.003)
PM_{10}	**IRR:1.061(1.003,1.119)*** **RERI:0.054(0.013,0.095)***	IRR:1.024(0.965,1.082) RERI:0.023(-0.020,0.065)	IRR:1.040(0.986,1.094) RERI:0.036(-0.007,0.078)	IRR:0.958(0.908,1.007) RERI:-0.042(-0.091,0.007)		IRR:1.068(0.994,1.142) **RERI:0.063(0.028,0.099)***
$PM_{2.5}$	IRR:1.007(0.951,1.064) RERI:0.004(-0.038,0.045)	IRR:1.027(0.968,1.087) RERI:0.026(-0.017,0.069)	IRR:1.021(0.969,1.073) RERI:0.017(-0.028,0.061)	IRR:0.975(0.923,1.026) RERI:-0.025(-0.075,0.025)	IRR:1.072(0.998,1.147) **RERI:0.067(0.030,0.103)***	

Supplementary Table 62 Interaction analysis between various air pollutants on coronary heart disease hospitalizations in Henan Province from 2016 to 2021(df of temperature = 4)

pollutant	CO	NO_2	SO_2	O_3	PM_{10}	$PM_{2.5}$
CO		**IRR:1.115(1.053,1.177)*** **RERI:0.096(0.051,0.141)***	**IRR:1.247(1.178,1.317)*** **RERI:0.198(0.155,0.241)***	IRR:0.962(0.912,1.012) RERI:-0.034(-0.086,0.019)	**IRR:1.064(1.006,1.122)*** **RERI:0.059(0.019,0.100)***	IRR:1.006(0.950,1.063) RERI:0.005(-0.036,0.046)
NO_2	**IRR:1.116(1.054,1.178)*** **RERI:0.093(0.050,0.137)***		IRR:1.024(0.968,1.081) RERI:0.013(-0.030,0.057)	IRR:0.985(0.932,1.038) RERI:-0.015(-0.067,0.036)	IRR:1.032(0.973,1.091) RERI:0.029(-0.011,0.070)	IRR:1.034(0.974,1.094) RERI:0.033(-0.008,0.073)
SO_2	**IRR:1.243(1.174,1.312)*** **RERI:0.196(0.152,0.241)***	IRR:1.037(0.979,1.094) RERI:0.028(-0.018,0.074)		IRR:0.998(0.946,1.051) RERI:0.002(-0.050,0.055)	IRR:1.051(0.996,1.105) **RERI:0.049(0.005,0.093)***	IRR:1.023(0.971,1.076) RERI:0.021(-0.024,0.066)
O_3	**IRR:0.938(0.888,0.987)*** RERI:-0.046(-0.098,0.006)	IRR:0.956(0.904,1.007) RERI:-0.045(-0.098,0.008)	IRR:0.976(0.924,1.028) RERI:-0.008(-0.059,0.043)		**IRR:0.934(0.885,0.983)*** **RERI:-0.062(-0.111,-0.012)***	IRR:0.950(0.900,1.001) RERI:-0.048(-0.098,0.001)
PM_{10}	**IRR:1.062(1.004,1.120)*** **RERI:0.055(0.014,0.096)***	IRR:1.024(0.966,1.083) RERI:0.023(-0.019,0.066)	IRR:1.041(0.987,1.094) RERI:0.036(-0.006,0.079)	IRR:0.955(0.905,1.004) RERI:-0.045(-0.095,0.005)		IRR:1.068(0.994,1.143) **RERI:0.064(0.028,0.099)***
$PM_{2.5}$	IRR:1.008(0.951,1.065) RERI:0.004(-0.038,0.046)	IRR:1.028(0.968,1.087) RERI:0.027(-0.016,0.070)	IRR:1.021(0.969,1.073) RERI:0.017(-0.027,0.061)	IRR:0.972(0.921,1.023) RERI:-0.028(-0.078,0.023)	IRR:1.073(0.998,1.147) **RERI:0.067(0.030,0.104)***	

Supplementary Table 63 Interaction analysis between various air pollutants on coronary heart disease hospitalizations in Henan Province from 2016 to 2021(df of relative humidity = 2)

pollutant	CO	NO_2	SO_2	O_3	PM_{10}	$PM_{2.5}$
CO		**IRR:1.115(1.053,1.176)***	**IRR:1.248(1.178,1.317)***	IRR:0.963(0.913,1.013)	**IRR:1.067(1.009,1.125)***	IRR:1.007(0.951,1.064)
		**RERI:0.096(0.051,0.141)***	**RERI:0.198(0.155,0.241)***	RERI:-0.033(-0.085,0.019)	**RERI:0.061(0.021,0.102)***	RERI:0.005(-0.036,0.046)
NO_2	**IRR:1.115(1.053,1.177)***		IRR:1.023(0.967,1.080)	IRR:0.980(0.928,1.033)	IRR:1.030(0.971,1.089)	IRR:1.031(0.971,1.091)
	**RERI:0.093(0.049,0.136)***		RERI:0.012(-0.031,0.056)	RERI:-0.020(-0.072,0.032)	RERI:0.028(-0.013,0.068)	RERI:0.030(-0.011,0.070)
SO_2	**IRR:1.243(1.174,1.312)***	IRR:1.036(0.979,1.093)		IRR:0.996(0.944,1.049)	IRR:1.049(0.995,1.104)	IRR:1.022(0.970,1.075)
	**RERI:0.196(0.152,0.241)***	RERI:0.027(-0.019,0.073)		RERI:0.000(-0.053,0.053)	**RERI:0.048(0.004,0.091)***	RERI:0.021(-0.024,0.065)
O_3	**IRR:0.939(0.889,0.989)***	IRR:0.952(0.901,1.003)	IRR:0.973(0.922,1.025)		**IRR:0.931(0.882,0.979)***	IRR:0.949(0.899,0.999)
	RERI:-0.045(-0.097,0.007)	RERI:-0.049(-0.102,0.004)	RERI:-0.010(-0.062,0.041)		**RERI:-0.065(-0.114,-0.016)***	RERI:-0.049(-0.099,0.000)
PM_{10}	**IRR:1.065(1.007,1.123)***	IRR:1.023(0.964,1.082)	IRR:1.039(0.985,1.092)	IRR:0.951(0.902,1.001)		IRR:1.066(0.992,1.141)
	**RERI:0.057(0.016,0.098)***	RERI:0.022(-0.021,0.064)	RERI:0.035(-0.008,0.077)	RERI:-0.048(-0.098,0.002)		**RERI:0.062(0.026,0.098)***
$PM_{2.5}$	IRR:1.009(0.952,1.066)	IRR:1.025(0.966,1.085)	IRR:1.020(0.968,1.072)	IRR:0.971(0.920,1.022)	IRR:1.071(0.996,1.145)	
	RERI:0.005(-0.036,0.047)	RERI:0.025(-0.018,0.068)	RERI:0.016(-0.028,0.060)	RERI:-0.029(-0.080,0.021)	**RERI:0.065(0.029,0.102)***	

Supplementary Table 64 Interaction analysis between various air pollutants on coronary heart disease hospitalizations in Henan Province from 2016 to 2021(df of relative humidity = 4)

pollutant	CO	NO_2	SO_2	O_3	PM_{10}	$PM_{2.5}$
CO		**IRR:1.115(1.054,1.177)*** **RERI:0.096(0.051,0.141)***	**IRR:1.246(1.177,1.316)*** **RERI:0.197(0.155,0.240)***	IRR:0.961(0.911,1.011) RERI:0.034(-0.087,0.018)	**IRR:1.065(1.006,1.123)*** **RERI:0.059(0.019,0.100)***	IRR:1.007(0.950,1.064) RERI:0.005(-0.036,0.046)
NO_2	**IRR:1.116(1.054,1.178)*** **RERI:0.093(0.050,0.137)***		IRR:1.025(0.968,1.082) RERI:0.014(-0.029,0.057)	IRR:0.983(0.930,1.036) RERI:-0.018(-0.070,0.034)	IRR:1.032(0.973,1.091) RERI:0.029(-0.011,0.070)	IRR:1.034(0.974,1.094) RERI:0.033(-0.008,0.073)
SO_2	**IRR:1.242(1.173,1.312)*** **RERI:0.196(0.151,0.240)***	IRR:1.038(0.980,1.095) RERI:0.029(-0.017,0.075)		IRR:0.996(0.944,1.049) RERI:0.000(-0.052,0.053)	IRR:1.050(0.996,1.105) **RERI:0.049(0.005,0.092)***	IRR:1.022(0.970,1.074) RERI:0.020(-0.025,0.065)
O_3	**IRR:0.937(0.888,0.987)*** RERI:-0.047(-0.098,0.005)	IRR:0.954(0.903,1.006) RERI:-0.046(-0.099,0.007)	IRR:0.974(0.922,1.026) RERI:-0.010(-0.061,0.042)		**IRR:0.933(0.884,0.982)*** **RERI:-0.062(-0.112,-0.013)***	IRR:0.949(0.898,1.000) RERI:-0.049(-0.099,0.000)
PM_{10}	**IRR:1.062(1.004,1.120)*** **RERI:0.055(0.014,0.096)***	IRR:1.025(0.966,1.083) RERI:0.023(-0.019,0.066)	IRR:1.040(0.986,1.094) RERI:0.035(-0.007,0.078)	IRR:0.953(0.904,1.003) RERI:-0.046(-0.096,0.004)		IRR:1.067(0.993,1.142) **RERI:0.063(0.027,0.099)***
$PM_{2.5}$	IRR:1.008(0.951,1.065) RERI:0.004(-0.038,0.046)	IRR:1.028(0.968,1.087) RERI:0.027(-0.016,0.070)	IRR:1.020(0.968,1.072) RERI:0.015(-0.029,0.060)	IRR:0.970(0.919,1.021) RERI:-0.030(-0.081,0.021)	IRR:1.072(0.997,1.146) **RERI:0.066(0.030,0.103)***	
